# Protein Phosphatase 2A Improves Cardiac Functional Response to Ischemia and Sepsis

**DOI:** 10.3390/ijms23094688

**Published:** 2022-04-23

**Authors:** Ulrich Gergs, Tina Jahn, Nico Schulz, Claudia Großmann, Uwe Rueckschloss, Uta Demus, Igor B. Buchwalow, Joachim Neumann

**Affiliations:** 1 Institut für Pharmakologie und Toxikologie, Medizinische Fakultät, Martin-Luther-Universität Halle-Wittenberg, D-06097 Halle, Germany; tina.jahn-1986@web.de (T.J.); nicoschulz@yahoo.de (N.S.); joachim.neumann@medizin.uni-halle.de (J.N.); 2 Julius-Bernstein-Institut für Physiologie, Medizinische Fakultät, Martin-Luther-Universität Halle-Wittenberg, D-06097 Halle, Germany; claudia.grossmann@medizin.uni-halle.de; 3 Institut für Anatomie und Zellbiologie, Julius-Maximilians-Universität Würzburg, D-97070 Würzburg, Germany; uwe.rueckschloss@uni-wuerzburg.de; 4 Gesellschaft zur Förderung von Medizin-, Bio-und Umwelttechnologien e. V., D-06120 Halle, Germany; demus@gmbu.de; 5 Institut für Hämatopathologie, D-22547 Hamburg, Germany; buchwalow@pathologie-hh.de; 6 Scientific and Educational Resource Center for Molecular Morphology, Peoples’ Friendship University of Russia, Moscow 117198, Russia

**Keywords:** protein phosphorylation, PP2A, transgenic mice, heart, LPS, sepsis, ischemia

## Abstract

Reversible protein phosphorylation is a posttranslational modification of regulatory proteins involved in cardiac signaling pathways. Here, we focus on the role of protein phosphatase 2A (PP2A) for cardiac gene expression and stress response using a transgenic mouse model with cardiac myocyte-specific overexpression of the catalytic subunit of PP2A (PP2A-TG). Gene and protein expression were assessed under basal conditions by gene chip analysis and Western blotting. Some cardiac genes related to the cell metabolism and to protein phosphorylation such as kinases and phosphatases were altered in PP2A-TG compared to wild type mice (WT). As cardiac stressors, a lipopolysaccharide (LPS)-induced sepsis in vivo and a global cardiac ischemia in vitro (stop-flow isolated perfused heart model) were examined. Whereas the basal cardiac function was reduced in PP2A-TG as studied by echocardiography or as studied in the isolated work-performing heart, the acute LPS- or ischemia-induced cardiac dysfunction deteriorated less in PP2A-TG compared to WT. From the data, we conclude that increased PP2A activity may influence the acute stress tolerance of cardiac myocytes.

## 1. Introduction

Reversible phosphorylation of proteins belongs to the essential mechanisms by which the function of the mammalian heart is regulated. The serine/threonine protein phosphatases type 1 (PP1) and type 2A (PP2A) are responsible for more than 90% of the cardiac serine/threonine phosphatase activity in the human heart. They control the dephosphorylation of multiple regulatory proteins involved in cardiac contractility, electrophysiology, gene expression, cell metabolism, and receptor signaling (reviewed in, e.g., [1,2]). The multiple functions of PP2A, at least in part, might be due to the highly variable structure of the PP2A holoenzyme. The trimeric PP2A holoenzyme is usually composed of a catalytic C-subunit, a structural A-subunit, and a regulatory B-subunit [3]. Whereas only two genes code for both the C- or A-subunits, at least 15 genes with additional splice variants for the B-subunit are known. Moreover, at least seven genes are coding for endogenous proteins that may act as PP2A inhibitors [3]. This variable composition of the PP2A-holoenzyme may form the basis of the substrate specificity and may contribute to the precise fine tuning of signaling pathways in the cell, though many aspects need more research. PP2A is generally thought to play an important role not only under physiological but also under pathophysiological conditions [1,4].

The heart belongs to the most frequently affected organs in patients suffering from sepsis. Sepsis-induced cardiomyopathy is accompanied by ventricular dysfunction, metabolic dysfunction, and elevated biomarkers [5,6,7]. Sepsis is caused most frequently by bacterial infections. Bacteria contain many molecules on their outer cell membrane and intracellular molecules that are released after bacterial lysis and that are recognized by specific receptors in the heart of the patient leading to an inflammatory cardiac response. One important bacterial surface molecule is the endotoxin lipopolysaccharide (LPS) that acts in the heart via the toll-like receptor 4 (TLR4) signaling cascade leading to activation of the nuclear factor kappa B (NF-κB) (review [8,9]). Finally, proinflammatory cytokines such as tumor necrosis factor alpha (TNFα) and interleukin 1 (IL-1) as well as high amounts of nitric oxide (NO) are released contributing to the organ dysfunctions in sepsis [8,9]. This pathway will be examined here also because it was shown that PP2A is involved in the regulation of the TLR4-mediated signaling [10].

Moreover, the role of PP2A-dependent protein phosphorylation in the heart was demonstrated using genetically modified mouse models. Cardiac overexpression as well as knock out of the catalytic subunit of PP2A led to cardiac hypertrophy and cardiac dysfunction [11,12].

As another clinically relevant stressor, we studied in the present work also the ischemia-reperfusion injury. As a consequence of myocardial infarction, the ischemia-reperfusion injury greatly contributes to morbidity as well as to mortality of the patients [13]. It might appear to be a paradox that the reperfusion of an ischemic heart leads to its damage. A main reason for this negative effect was identified as the generation of reactive oxygen species (ROS) [14,15]. By this way, the oxidation of nearly all cellular molecules such as lipids, proteins, and nucleic acids by free radicals changes the structure and function of these molecules and can lead to cell damage and cell death [13,15]. In this respect, it was particularly important for us that in nearly all signaling pathways that are affected by ischemia and reperfusion, protein phosphorylation is involved. These signaling pathways include, for example, the transcriptional control of gene expression, the inflammatory response, and the regulation of oxidative stress. Importantly, PP2A appears to be involved in all of these pathways [16].

Thus, we hypothesized that PP2A is involved in the response of the heart to sepsis or ischemia. We started with a comparison of the cardiac gene expression profiles in wild type (WT) and PP2A-transgenic (PP2A-TG) mice under physiological (control) conditions. Thereafter, we analyzed the cardiac response of WT and PP2A-TG mice in lipopolysaccharide-induced sepsis and in a separate experimental setup, we analyzed the response to a global ischemia in vitro.

## 2. Results

### 2.1. Differential Gene Expression under Basal Conditions in PP2A-TG Hearts

In previous work, we had never the opportunity to analyze the differential gene expression profile in PP2A-TG hearts. Such knowledge might be helpful in understanding the strength of our model better. To that end, total cardiac RNA was prepared from quickly frozen WT and PP2A-TG hearts under basal conditions, that is, untreated conditions. Thereafter, a mouse genome chip was used to get a complete overview of expressed genes. For further analyses, only genes with 1.5-fold up- or downregulation were taken into consideration (supplementary Table S1 and S2). Under these restrictions, 170 genes were established to be upregulated in PP2A-TG (supplementary Table S1) and 365 genes were downregulated in PP2A-TG (supplementary Table S2). In addition, the analysis of genes expressed in both WT and PP2A-TG and adjusted by a variance filter is shown in the supplementary Table S3. Further graphical presentations of the microarray data including principal component analyses, corresponding analyses, and t-Test results are shown in the supplementary Figures S1 to S4.

**Figure 1:**
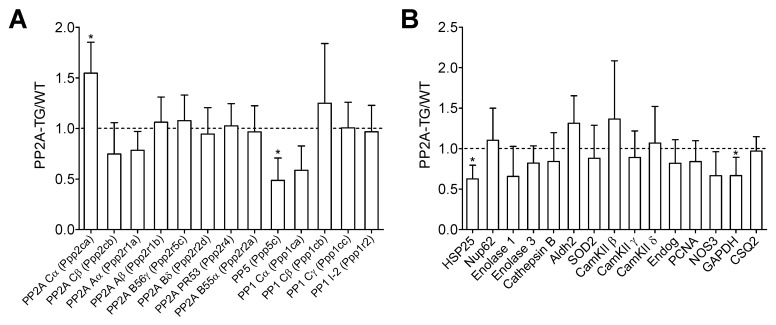
Differential mRNA expression. The basal cardiac gene expression profile of PP2A overexpressing (PP2A-TG) and littermate wild type (WT) mouse hearts was analyzed by a mouse genome gene chip. Data are presented as ratio of the mean PP2A-TG signal divided by the mean WT signal ± SD. More detailed data can be found in the supplementary Tables S1–S3 and supplementary Figures S1–S4. (**A**) Several subunits of different protein phosphatases are summarized. (**B**) A selection of genes with various functions is shown. The abbreviations are: aldehyde dehydrogenase 2 (Aldh2), Ca^2+^ calmodulin kinase II (CamKII), cardiac calsequestrin (CSQ2), endonuclease G (Endog), glycerin aldehyde phosphate dehydrogenase (GAPDH), heat shock protein 25 (HSP25), nitric oxide synthase 3 (NOS3), nucleoporin 62kDa (Nup62), proliferating cell nuclear antigen (PCNA), superoxide dismutase 2 (SOD2). Three RNA samples from each genotype (n = 3) were studied. * *p* < 0.05 vs. WT (by comparison of individual data sets).

**Figure 2:**
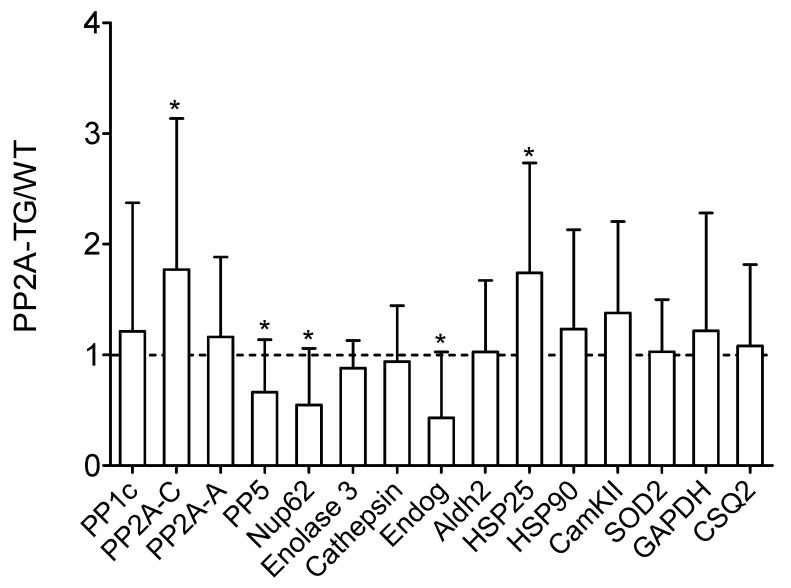
Basal cardiac protein expression. Basal cardiac protein expression of a selection of genes with various functions analyzed by Western blotting of wild type (WT) and PP2A overexpressing (PP2A-TG) mouse hearts. Original Western blots are shown in the supplementary Figures S5 and S6. Quantification data of Western blots are presented as ratio of the mean PP2A-TG signal divided by the mean WT signal ± SD. The abbreviations are: aldehyde dehydrogenase 2 (Aldh2), cardiac calsequestrin (CSQ2), Ca^2+^ calmodulin kinase II (CamKII), endonuclease G (Endog), glycerin aldehyde phosphate dehydrogenase (GAPDH), heat shock proteins 25 and 90 (HSP25, HSP90), nucleoporin 62kDa (Nup62), catalytic alpha subunit of protein phosphatase 1 (PP1c), structural A-subunit and catalytic C-subunit of protein phosphatase 2A (PP2A-A, PP2A-C), protein phosphatase 5 (PP5), superoxide dismutase 2 (SOD2). * *p* < 0.05 vs. WT.

Currently, the differentially expressed genes give no hint to a signaling pathway possibly disturbed in PP2A-TG hearts that could be an explanation for the functional cardiac restrictions found in these mice. We hypothesized that some genes related to the energy/cell metabolism and to protein phosphorylation (kinases, phosphatases) were altered in PP2A-TG hearts compared to WT (Figure 1). Therefore, we decided to test the protein expression of several genes, that were changed in the gene chip analysis and some genes accepted to be particularly relevant for the cardiac function in our mouse model but that were, surprisingly, not changed in the gene chip analyses on the mRNA level (Figure 1). The overexpression of the catalytic subunit of PP2A could be confirmed by both, gene chip analysis and Western blotting in PP2A-TG, as expected and a control of the system (Figure 1 and Figure 2). Furthermore, by Western blotting, we detected an upregulation of heat shock protein 25 (HSP25) and a downregulation of endonuclease G (Endog), nucleoporin 62 (Nup62), and protein phosphatase 5 (PP5) on protein level (Figure 2). Interestingly, HSP25 was downregulated in the gene chip. Likewise, other genes that were altered in the gene chip, e.g., protein phosphatase 1c alpha (PP1cα) or calcium calmodulin kinase II beta (CamKIIβ) were unchanged in Western blotting (compare Figure 1 and Figure 2). These findings cast doubt on the predictive power of a gene chip, at least under our experimental conditions. Similar discrepancies have been reported before (review: [17]). Original Western blots are presented in supplementary .

### 2.2. Role of PP2A in Sepsis

Protein phosphorylation is important for the regulation of inflammatory signaling [18,19]. We did not detect marked differences in inflammatory cytokine expression in our microarray. Nevertheless, because PP2A is a well-established regulator of inflammatory signaling in non-cardiac cell types [20,21], we hypothesized that the expression of pro-inflammatory genes may be altered in hearts of PP2A-TG mice.

#### 2.2.1. LPS and Echocardiography

**Figure 3:**
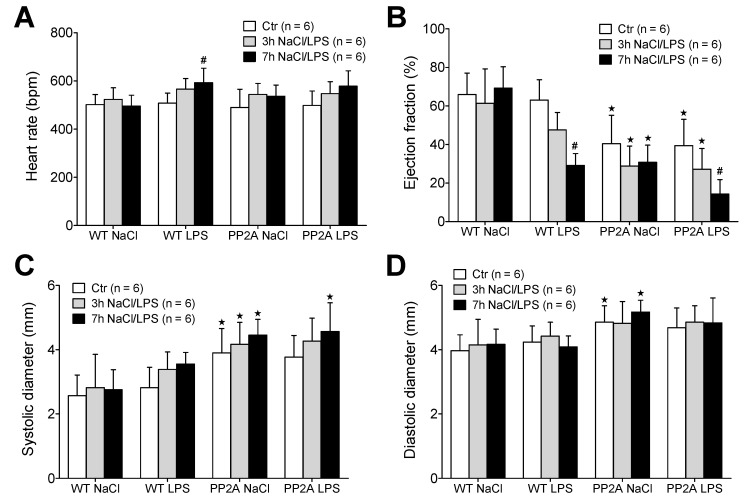
Echocardiography after LPS. Measurement of echocardiographic parameters of wild type (WT) and PP2A overexpressing mice before (Ctr) and 3 and 7 h after intraperitoneal application of LPS (25 mg/kg) or NaCl solution as solvent control. (**A**) Heart rate, (**B**) ejection fraction, (**C**) maximal systolic cardiac diameter of the left ventricle, (**D**) maximal diastolic cardiac diameter of the left ventricle of the heart. First bars always indicate basal conditions (before application of LPS), second bars indicate three hours after application of LPS or solvent control (NaCl). Third bars indicate seven hours after application of LPS or solvent control (NaCl). Numbers in brackets indicate the number of experiments. ^★^ *p* < 0.05 vs. WT; # *p* < 0.05 vs. NaCl.

First, WT and PP2A-TG mice were divided further into four groups with subsequent LPS-injection or with NaCl-(solvent control)-injection (Figure 3). The basal cardiac function of each individual mouse was assessed by echocardiography. Thereafter, a single intraperitoneal injection of LPS (25 mg/kg in 0.9% NaCl) or isotonic 0.9 % NaCl solution in water (as solvent control) was administered. The development of a possible cardiac dysfunction was monitored three hours and seven hours after LPS-or NaCl-injection in each single mouse (Figure 3). This was done because sepsis is known to develop over time. We used two time points of measurement because this was expected to facilitate our understanding of putative underlying mechanisms of the sepsis. The heart rate was not different between WT and PP2A-TG under basal conditions, but was increased by LPS in WT seven hours after LPS-injection, whereas in PP2A-TG mice no significant increase was noted (Figure 3A). However, the contractility of the PP2A-TG hearts was impaired already under control (basal) conditions as demonstrated by a reduced left ventricular ejection fraction and increased ventricular diameters (Figure 3B–D). Furthermore, the application of LPS led to a significant reduction in the systolic cardiac function, demonstrated by a time-dependent fall of the left ventricular ejection fraction in both WT and PP2A-TG mice (Figure 3B). After seven hours, the narcotized mice were euthanized and the hearts were removed to perform an analysis of the mRNA expression.

#### 2.2.2. LPS and Working Heart

**Figure 4:**
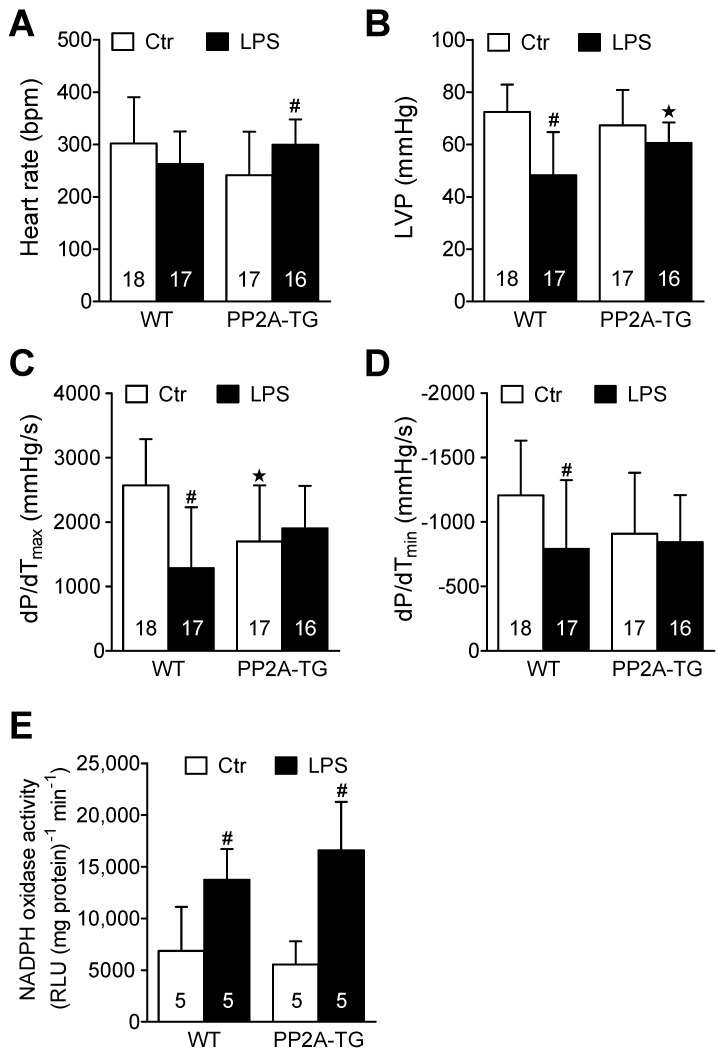
Isolated perfused hearts after LPS. Working heart preparations of wild type (WT) and PP2A overexpressing mouse hearts 3 days after intraperitoneal application of LPS (25 mg/kg) or NaCl solution as control. (**A**) Heart rate, (**B**) maximum left ventricular pressure (LVP), (**C**) maximum rate of left ventricular pressure development (+dP/dt), (**D**) maximum rate of left ventricular pressure decline (−dP/dt), (**E**) activity of NADPH oxidase in cardiac tissue from WT or PP2A-TG. Numbers in brackets indicate the number of experiments ^★^ *p* < 0.05 vs. WT; # *p* < 0.05 vs. control (Ctr).

In a complementary experimental setup, we analyzed the cardiac function in vitro three days after LPS- or NaCl-injection using the isolated work-performing heart preparation to measure the cardiac contractility independent of any neuronal or humoral influences that are operational in living mice. Under control conditions, heart rate and maximum left ventricular pressure were, at first glance, not different between WT and PP2A-TG hearts in vitro (Figure 4A,B). However, when looking at the maxima of the derivative of pressure with respect to time (dP/dt), as a measure of left ventricular performance, the basal contractility of PP2A-TG hearts was reduced compared to WT (Figure 4C,D) which is in line with our echocardiographic data (Figure 3B). Under septic conditions, three days after LPS application, the contractility of WT hearts was impaired compared to control without any changes in heart rate (Figure 4). Interestingly, the opposite was found for PP2A-TG hearts: after LPS-injection, we measured in PP2A-TG hearts an increased heart rate but preserved cardiac contractility compared to control (Figure 4). This may be different from the situation in the living mouse, where, for instance, the sympathetic nerve system would control to a high extent the heart rate. One can easily reconcile the altered heart rate, reduced function in vitro, and preserved function in the living animal. The mouse ventricle displays a positive “treppe” phenomenon. In other words, in the isolated mouse heart (as in the human heart: [22]) an increase in the heart rate alone is sufficient to raise force of contraction [23]. We suggest this to have happened here. At the end of the experiments, the hearts were freeze-clamped and homogenized to analyze the mRNA expression of selected genes (4.2.3.). In addition, the activity of NADPH oxidase was estimated in these cardiac homogenates in order to assess oxidative stress (Figure 4E). In the LPS groups, the NADPH oxidase activity was increased 2-fold compared to the control hearts. There was no difference in this regard between WT and PP2A-TG hearts neither under control conditions nor after LPS (Figure 4E).

#### 2.2.3. LPS and Gene Expression

**Figure 5:**
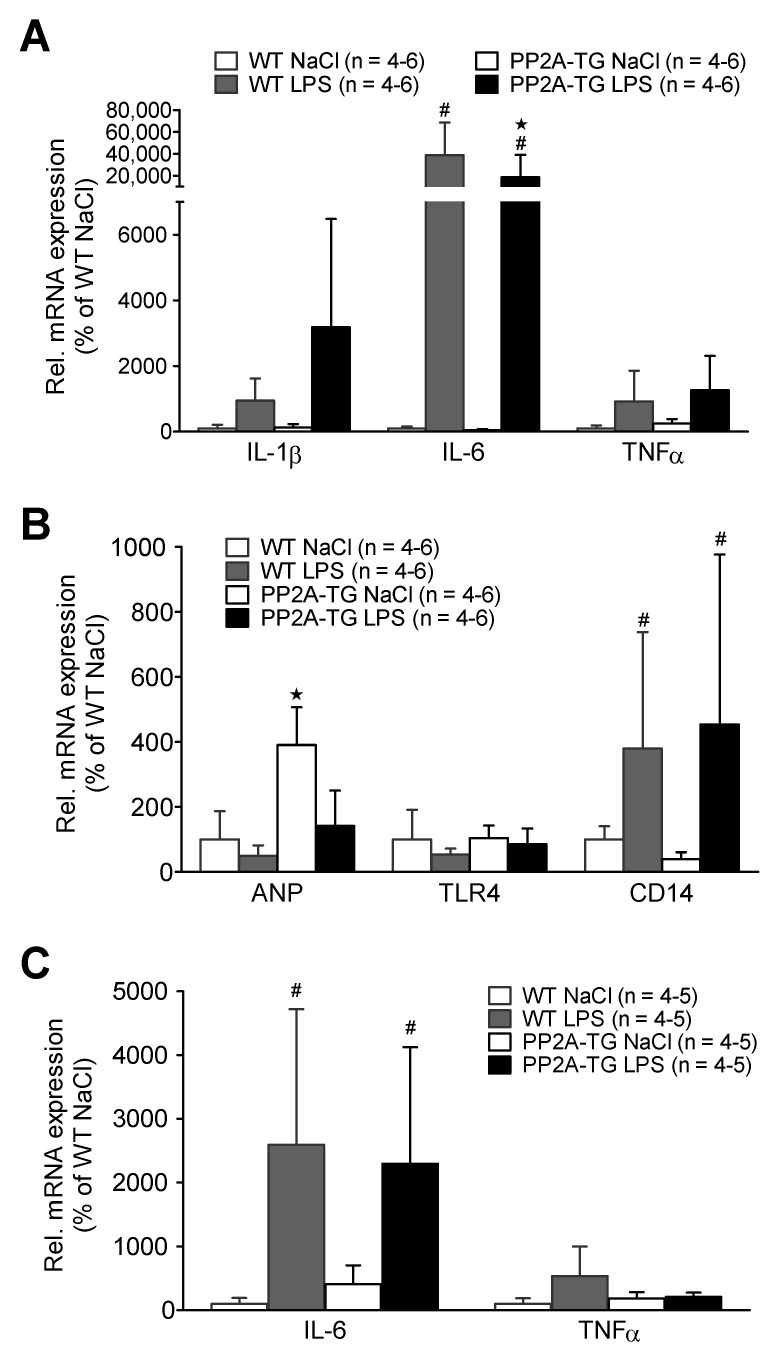
LPS-mediated gene expression. Cardiac gene expression analyzed by qPCR of wild type (WT) and PP2A overexpressing (PP2A-TG) mouse hearts after intraperitoneal application of LPS or NaCl solution as control (Ctr) after 7 h. Ordinates display relative mRNA expression in percentage of control, that is, WT hearts from mice treated with isotonic sodium chloride solutions alone (solvent control). (**A**) displays the mRNA for interleukin 1 beta (IL-1β), for interleukin 6 (IL-6) and for tumor necrosis factor alpha (TNFα). (**B**) displays the mRNA for atrial natriuretic peptide (ANP), for toll like receptor 4 (TLR4) and for cluster of differentiation 14 protein (CD14). Three days after LPS injection in (**C**) mRNA for interleukin 6 (IL-6) and tumor necrosis factor alpha (TNFα) as depicted in the ordinate. Note that after three days, expression of cytokine mRNAs nearly reached basal values. Data were normalized to GAPDH (**A,B**) or 18S RNA (**C**) mRNA expression and WT by the 2^−ΔΔCT^ method. ^★^ *p* < 0.05 vs. Ctr, # *p* < 0.05 vs. WT.

After functional measurements (Figure 4), the expression of cardiac genes involved in the LPS-activated inflammatory pathway was analyzed by real time RT-PCR. After seven hours of LPS treatment (experimental setup in 4.2.1), some key genes of the TLR4 signaling pathway were analyzed. The mRNAs encoding for the cytokines IL-1β, IL-6, and TNFα were increased seven hours after LPS in WT as well as in PP2A-TG (Figure 5A). Additionally, the mRNA for CD14 that is part of the LPS receptor complex was increased by LPS but the mRNA of TLR4, the LPS receptor itself, remained unchanged in all groups (Figure 5B). Interestingly, the expression the mRNA for ANP, a marker for cardiac hypertrophy, that was noted to be elevated in PP2A-TG controls, as expected in a mouse model with cardiac hypertrophy [11], decreased to WT levels after LPS (Figure 5B). Three days after LPS-treatment, the mRNA for IL-6 was still augmented in WT as well as in PP2A-TG. However, the mRNA for TNFα was slightly raised only in WT and completely reduced to the control level in PP2A-TG (Figure 5C).

### 2.3. Role of PP2A in Ischemia/Reperfusion

#### 2.3.1. Cardiac Function In Vitro

**Figure 6:**
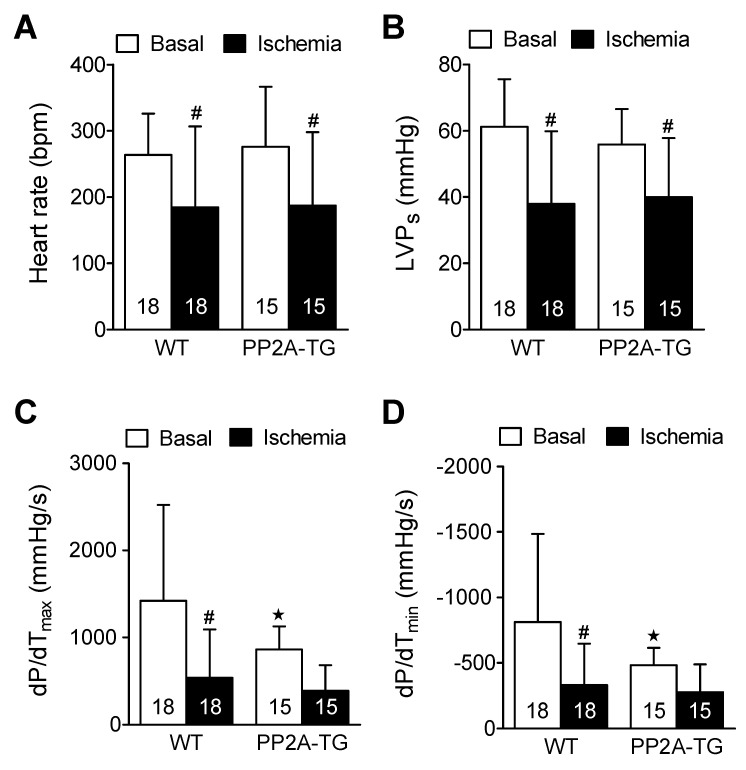
Ischemic cardiac function in isolated perfused hearts from wild type hearts (WT) and heart with cardiac specific overexpression of the catalytic subunit of protein phosphatase 2A (PP2A-TG). Contraction parameters were assessed under basal conditions and 120 min after ischemia. Heart rate is depicted as beats per minute (BPM) under (**A**), maximum left ventricular pressure as milli meter mercury column (mm Hg) (**B**) as LVPs, maximum first derivative of pressure in left ventricle in millimeter mercury column per second (mmHg/s) in the ordinate of (**C**), minimum first derivative of pressure in left ventricle in millimeter mercury column per second (mmHg/s) in the ordinate of (**D**), open bars indicated basal perfusion conditions and filled bars indicate values at the end of the ischemia. Numbers in bars depict the numbers of experiments in hearts from WT or PP2A-TG. ^★^ *p* < 0.05 vs. WT, # *p* < 0.05 vs. basal conditions.

In a previous study, where we investigated myocardial infarction (by occluding in vivo the left coronary artery) in PP2A-TG, we observed a protective role of PP2A [24]. This motivated us to find out whether PP2A affects cardiac performance in a model of ischemia and reperfusion where any influence of the blood and the nervous system does not confound the interpretation of the experimental results. Thus, we employed the working heart set-up. In this model, we induced a global cardiac ischemia by ceasing the perfusion of the isolated heart for 120 min, a time period that was found in pilot studies (data not shown) to result in an irreversible functional impairment of the WT and PP2A-TG myocardium (Figure 6). The main findings were that cardiac contractility and heart rate were reduced after ischemia. Indeed, the reductions were similar in WT and PP2A-TG hearts. Interestingly, the maximum and minimum rates of left ventricular pressure development (dP/dtmax and dP/dtmin) were smaller under basal conditions in PP2A-TG. Therefore, the relative decline in maximum and minimum rate of left ventricular pressure development under ischemia was less in PP2A-TG compared to WT (Figure 6). This observation is consistent with the data recorded by the working heart set-up three days after LPS-treatment where the pressure development in PP2A-TG was decreased even under basal conditions but no further under septic conditions (Figure 4).

#### 2.3.2. NADPH Oxidase and Nitric Oxide Synthases

**Figure 7:**
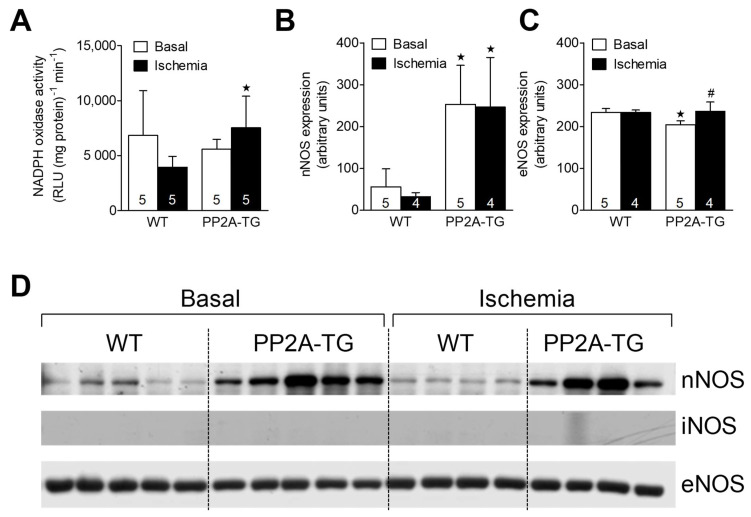
The cardiac activity of NADPH oxidase is plotted in (**A**). Western blot data for the expression of neuronal (**B**) and endothelial (**C**) nitric oxidase synthase (nNOS and eNOS) in the hearts from WT and PP2A-TG are plotted. The open bars indicate basal perfusion conditions and filled bars indicate values at the end of the ischemia. Original nitrocellulose membranes are shown in (**D**) for expression of nNOS (upper tracing) or eNOS (lower tracing), before ischemia (basal, left-hand side) and after 120 min of ischemia (right-hand side). The inducible NOS (iNOS) was not detectable by Western blotting (middle tracing). In the supplementary Figure S7, the corresponding fast green stained membranes are shown to demonstrate equal protein loading. The data for nNOS and eNOS are summarized as means and SD for 4–5 hearts and are presented in bar diagrams in (**B**,**C**). ^★^ *p* < 0.05 vs. WT, # *p* < 0.05 vs. basal conditions.

**Figure 8:**
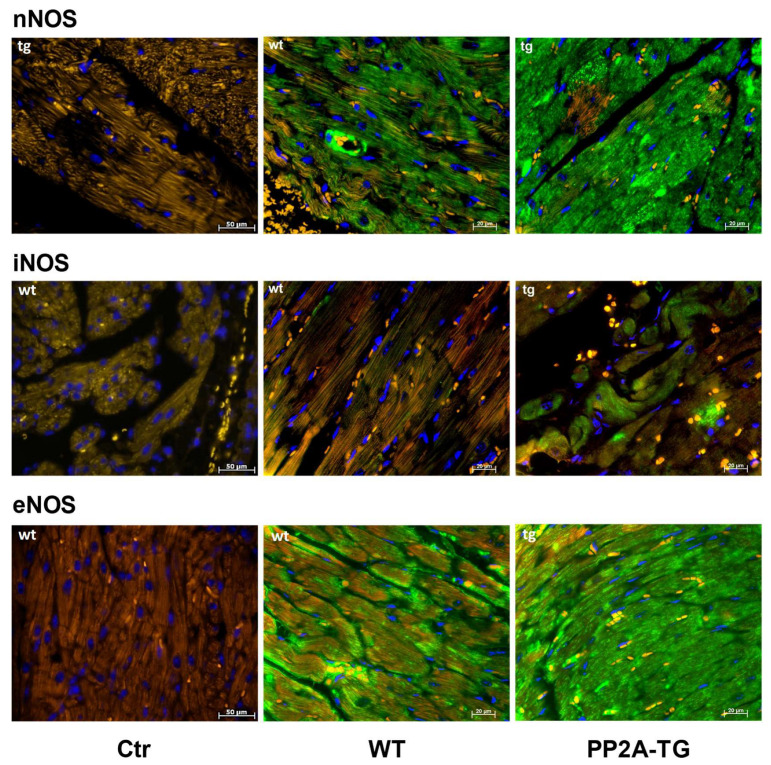
Immunohistochemical detection of NOS1 (nNOS), NOS2 (iNOS), and NOS3 (eNOS) in ventricular tissue sections from wild type (WT) and PP2A transgenic mice (TG). Controls (Ctr) are samples with omission of the first antibody. Bars in the lower right angle of the photographs indicate the length markers in micrometers (µm). Nitric oxide synthase (NOS) isoforms are stained in green. Nuclei are counterstained in blue with DAPI (4′,6-diamidino-2-phenylindole). Red blood cells appear yellow colored due to autofluorescence. Note the differences in NOS expression between WT and TG. This set of hearts is representative of two other experiments.

As reported above for LPS samples, the activity of NADPH oxidase was measured as a source of reactive oxygen species (ROS) and was found to be increased after 120 min of ischemia in PP2A-TG hearts compared to WT (Figure 7). As NO is suggested to be involved in oxidative processes in cardiac ischemia [25], the expression of the main nitric oxide synthase (NOS) isoforms was analyzed by Western blotting (Figure 7 and supplementary Figure S7) and then by immunohistochemistry (Figure 8). The NOS3, also known as endothelial or eNOS, was readily detectable in both WT and PP2A-TG hearts and was not different between genotypes (Figure 7 and Figure 8). The expression of NOS1, also known as neuronal or nNOS, was noted in both WT and PP2A-TG but was interestingly higher in PP2A-TG than in WT (Figure 7 and Figure 8). The so-called inducible isoform NOS2 or iNOS was not detectable by Western blotting in the examined set of samples, neither in WT nor in PP2A-TG (Figure 7). However, conceivably because the reaction of the antibody with the antigenic epitope of iNOS is better under these conditions, in immunohistochemistry, a slight expression of iNOS was noted, but confined to small regions of mouse ventricular slices and thus of doubtful relevance in our context (Figure 8).

## 3. Discussion

The main new findings of the present work are evidence that PP2A can be protective against cardiac sepsis and cardiac global ischemia.

Protein phosphorylation is an essential regulatory mechanism in nearly all aspects of cardiovascular function. In diseased human hearts, for example, an elevated protein phosphatase activity was found [26]. A role of PP2A in cardiac pathophysiology was demonstrated in a mouse model with PP2A overexpression [11,27]. Moreover, Mishra et al. (2020) highlighted the importance of protein phosphorylation for the cellular protein quality control and listed examples for the relation between cardiac diseases and inadequate protein quality control mechanisms [28]. Nevertheless, it remains unclear under what conditions a booster in cardiac protein phosphatase activity, in general, is detrimental for inotropy. PP2A overexpression in mouse cardiomyocytes led to cardiac hypertrophy and cardiac dysfunction [11] and by co-overexpression of PP5, the detrimental effects were further worsened [27]. Moreover, overexpression of PP2A was found to be protective in the case of myocardial infarction [24] or β-adrenergically induced cardiac hypertrophy [29]. To get further insight into this issue, we studied two additional cardiac stressors in these transgenic mice: either LPS-induced sepsis or ischemia in the isolated spontaneously beating heart.

The broad range of functions altered by PP2A, at least in cell culture or in isolated proteins, motivated us to analyze the gene expression profile in PP2A-TG hearts compared to WT hearts. Especially, we had recently demonstrated by a proteomic approach that the overall protein expression profile is different between WT and PP2A-TG [30]: we noted a differential protein profile but could not identify the differentially expressed cardiac proteins between WT and PP2A-TG, because of the limitation of that method. Therefore, we performed the gene chip experiments where all mRNAs are probably measurable. Several differentially expressed genes were noted, but the variability was high and no clear hint to a special signaling pathway altered in our model could be derived from the data. Moreover, the verification by Western blotting revealed paradoxical findings for some genes. This may be explained at least in part by the age and gender of the experimental mice. For gene expression analysis, five-month-old male mice were used whereas for Western blotting, the mice were about six months of age and both sexes were used. Even if we did not find any differences in the protein expression between male and female PP2A-TG at least for the studied proteins so far, this is a limitation of the study.

Nevertheless, based on previous studies [24,29], we expected a protective influence of PP2A also in relation to cardiac stressors which we had not tried before in this model. Our choice of stressors was driven by their clinical relevance: beyond any reasonable doubt, a clinically important stressor is the infection-related cardiac dysfunction in sepsis because it confers a high risk to lead to a sepsis-induced cardiomyopathy associated with a high incidence of death [31]. In animal experimental models, the bacterial endotoxin LPS is widely used as inductor of a sepsis. Additionally, we ourselves have used LPS before with success and had developed the necessary treatment protocols in mouse hearts [18,32]. In the heart, LPS binds to the receptor protein TLR4 that is involved in heart dysfunction [33]. In this context, the transcription factor NF-κB plays an important role in inflammation by translating the LPS-TLR4 signaling to the transcription of pro-inflammatory genes [34]. Moreover, PP2A expression and activity were reduced in a mouse model with LPS-induced sepsis, leading to an elevated phosphorylation state of the inhibitory subunit of troponin and reduced contractility of isolated cardiomyocytes [35]. Moreover, PP2A is involved in the regulation of the NF-κB pathway [36]. The binding of LPS to TLR4 initiates a signaling cascade eventually leading to NF-κB and c-Jun activation and subsequently resulting in an increased expression of some related pro-inflammatory genes such as IL-1β or TNFα [10]. This signaling cascade is mainly maintained by phosphorylation and PP2A was found to act as important regulator at various points of the signal chain. For example, PP2A is targeted to the IκBα kinase (IKK) complex as well as to phospho-IκBα (α inhibitor of NF-κB), dephosphorylates and inactivates both IKKα/β and IκBα, and thereby prevents sustained activation of NF-κB [10]. By this mechanism, PP2A may offer protection against LPS-induced inflammation. This assumption is supported, for example, by a study on endotoxin tolerance using human monocytes: in LPS-treated cells, the authors established a higher phosphatase activity and an enhanced PP2A expression controlling TLR4 signaling and thereby endotoxin tolerance [37]. Here, we noted a possible endotoxin tolerance in the mechanical function of PP2A-TG mice but not on the level of cytokine expression. Possibly, the overexpressed catalytic subunits of PP2A (“superfluous PP2Ac”) could not bind to the related phospho-proteins because of missing regulatory subunits responsible for the targeting of the holoenzyme. This is plausible because the gene expression analysis of the PP2A-TG hearts (but also previous Western blots: [11]) indicated unchanged expression of PP2A A- and B-subunits including the B56γ-subunit that is suggested to act as a targeting subunit to the NF-κB pathway together with the scaffolding A-subunits [19].

We have long-standing experience in using global ischemia in isolated perfused mouse hearts to assess the role of overexpressed genes in transgenic mouse hearts. For instance, using this model, we noted cardiac protection against ischemia in the heart of transgenic mice that overexpressed the H_2_-histamine receptor, the 5-HT_4_-serotonin receptor, and the A_2A_–adenosine receptor [32,38,39,40,41]. These receptors are pertinent in the present context because their activation decreases protein phosphatases activity [18]. As concerns enhanced activity of cardiac phosphatases, it is noteworthy that the present model of ischemia and reperfusion can differentiate between important and probably unimportant cardiac phosphatases for ischemia and reperfusion: for instance, using this set up we did not observe alterations in cardiac function between WT and mice that overexpressed cardiac PP2C [42]. In contrast, we noted cardioprotective effects in this set up for mice with cardiac overexpression of PP5 (PP5-TG) alone or in combination (PP2A-TG crossbred with PP5-TG) [18,27]. Ischemia and reperfusion injuries have been studied for decades. In our view, all these studies basically agree on a crucial role of cytosolic calcium (review: [43]). Hence, we hypothesize here that PP2A alters the phosphorylation state and thence the function of proteins that regulate the calcium homeostasis. In previous work, we have shown repeatedly that cardiac regulatory proteins such as phospholamban are substrates for protein phosphatases namely PP2A (review: [44]). More specifically, reduced phosphorylation of phospholamban has been reported by us in PP2A overexpressing mice [11]. Our group was not the first to look for a role of PP2A in cardiac ischemia. Using fostriecin, a drug developed as a cancer therapy because it inhibited topoisomerase II, colleagues could present evidence in isolated perfused rabbit hearts that PP2A exerts detrimental cardiac effects in ischemia and reperfusion [45]. Specifically, they could show that fostriecin given 15 min before no flow ischemia or 30 min into reperfusion protects the cardiac tissue against ischemic damage [45]. These data convincingly show that fostriecin can protect the heart [45]. However, these results were obtained in acute application of fostriecin (with the perfusion buffer) with genetically unaltered animals (“wild type rabbits”). In our model, PP2A has led to numerous alterations in gene expression (see gene chip data) and in addition, PP2A already had dephosphorylated, for instance, phospholamban, a protein involved in calcium homeostasis [11] before the start of the experiment. Hence, the apparent contradictory findings between Weinbrenner et al. ([45]: detrimental role of PP2A) and our data might be due to the duration of altered PP2A activity in both model (minutes versus months). This is in line with our previous study where PP2A had a detrimental role under basal conditions in PP2A-TG, in a similar way as represented here, but improved survival after myocardial infarction [24]. Alternatively, fostriecin might not be so specific for PP2A as initially thought: we now know that fostriecin also potently inhibits (in the nanomolar range) the activity of a protein phosphatase called PP4 [46] (our lab is now overexpressing PP4 in the heart for that reason) and, of course, species differences (mouse versus rabbit) could well play a role. On a positive note, our findings and theirs concur on a role of PP2A in ischemia and reperfusion and more work is expected to resolve this interesting contradiction in the literature.

Finally, we felt it important to clarify how free radical formation including NO production might play a role in our functional findings in sepsis and ischemia in PP2A-TG. In rodent models of cardiac pressure overload or myocardial infarction, the three NOS isoforms (NOS1, NOS2, and NOS3) may play neutral, protective, or even detrimental roles in myocardial remodeling, depending on the NOS activity, the cellular and even subcellular location of each NOS and their regulators [25,47]. NOS can be protective in human ischemia/reperfusion: an increase in NOS can be associated with augmented calcium levels activating the activity of NOS and may result in increased S-nitrosylation of the L-type calcium channel, less calcium entry, and therefore reduced potentially damaging calcium levels in the cytosol during ischemia [48].

In a complementary experiment, after myocardial infarction, in mice with genetic deletion of NOS1 (which is increased in PP2A-TG, Figure 7) pronounced cardiomyocyte hypertrophy and left ventricular dilation was noted: this suggested to the authors a protective role of NOS1 [49]. Taken together, the findings suggest that upregulation of myocardial NOS1 in infarcted hearts may be an important adaptive mechanism [49]. Fittingly, PP2A-TG expressed much more NOS1 protein than WT. In agreement with this, we noticed a higher NADPH oxidase activity in PP2A-TG than in WT. One can speculate that this higher basal activity of NOS and elevated production of free radicals might contribute to better protection of the hearts from PP2A-TG compared to WT with respect to both ischemia and sepsis. Moreover, one might conclude that increased expression of NOS1 in PP2A-TG might, in part, explain the lower initial mechanical performance of isolated perfused hearts from PP2A-TG compared to WT. Indeed, high levels of NO produced in the heart can reduce cardiac inotropy [50], in part by inhibiting the Ca^2+^ release through the ryanodine receptor in the sarcoplasmic reticulum [51].

In summary, we have extended our previous studies on cardiac roles of protein phosphatases [44,52] and specifically of PP2A. Here, we present experimental data that PP2A may be able to weaken the decrease in cardiac function during cardiac sepsis and cardiac ischemia. However, the detailed biochemical pathways involved still need to be meticulously studied in detail. At this stage, we nevertheless speculate that PP2A might be a druggable target for cardiac therapy of sepsis and ischemia.

## 4. Materials and Methods

### 4.1. Transgenic Mice

Here, transgenic mice with cardiomyocyte-specific overexpression of the catalytic subunit of PP2A were used [11]. Transgene-positive mice (CD1 background) were routinely identified by PCR assay of tail genomic DNA. Hearts from PP2A-TG mice showed a 2.5-fold overexpression of the catalytic subunit of PP2A on protein level. The phenotype of this mouse model has been described repeatedly [11,24,53,54]. Briefly, life span and fertility of these mice were unchanged compared to WT. However, age-dependently, PP2A-TG mice develop a cardiac hypertrophy, decreased cardiac function, and diminished response to β-adrenergic stimulation. On the other hand, PP2A-TG mice showed improved survival after myocardial infarction compared to WT mice [24]. For microarray experiments, 5-month-old male littermates were used. For all other experiments, 6–8-month-old mice of each gender were used. The investigation conforms to the Guide for the Care and Use of Laboratory Animals published by the National Research Council (US) 2011 [55]. The animal experiments were approved by the local ethics committee of the state Sachsen-Anhalt (Permit Number: 42502-02-691 MLU). For euthanasia of mice, intraperitoneal injection of 80 mg pentobarbital per kg body weight was used. All efforts were made to minimize suffering.

### 4.2. RNA Extraction and cDNA Synthesis

For analysis of cardiac mRNAs, the total RNA was isolated from the whole hearts with the TRIzol™ reagent (Invitrogen, by Fisher Scientific, Schwerte, Germany) according to the manufacturer’s protocol. For reverse transcription, the Maxima First Strand cDNA Synthesis Kit combined with a DNase I digestion to get rid of contaminating DNA was used as described by the manufacturer (Fisher Scientific, Schwerte, Germany). Briefly, about 5 µg total RNA and a mixture of oligo(dT)18 and random hexamer primers were used for reverse transcription. Finally, the cDNA was diluted with nuclease free water to a volume corresponding to 0.1 µg of originally added RNA per µL. A NRT (no reverse transcription) control was prepared for all RNA samples by omission of the enzyme.

### 4.3. Gene Chip Analysis

The gene expression analysis was performed with slight modifications as described elsewhere [56]. Total RNA was extracted from 50–100 mg of heart tissue as described above. The RNA concentration was assayed photometrically at 260 nm (BioPhotometer, Eppendorf, Hamburg, Germany) and the quality of the RNA samples was assessed by the integrity of the ribosomal 18S and 28S RNAs using a denaturing agarose gel. All further steps including hybridization and gene chip scanning were performed in the Core Unit DNA Technologies at the Medical Faculty of the University of Leipzig. The GeneChip Mouse Genome 430 2.0 Array (Affymetrix, Santa Clara, CA, USA) was used and the data were processed with the corresponding microarray analysis software (Affymetrix, Santa Clara, CA, USA) according to the manufacturer’s manual. After data processing, the subsequent analysis was performed only with genes expressed in both WT and PP2A-TG. Data analysis was performed with the stand-alone client of the TM4 application MeV (https://mev.tm4.org (last accessed on 7 April 2022) [57].

### 4.4. Real Time PCR

The cDNA samples were analyzed by real time PCR using a BioRad CFX Connect™ system using together with the iTaq SYBR Green kit (Bio-Rad Laboratories, Munich, Germany). As controls, the NRT samples and for all primer pairs no template controls (NTCs) were amplified. At the end of each PCR protocol, a melting curve of the PCR products was performed. Finally, the signal of the 18S or GAPDH amplification was used to normalize the PCR data and to calculate the relative expression of the genes of interest according to the 2^−ΔΔCT^ method [58]. The following primer sequences were used:

18S RNA:

Sense:   5′-GTTGGTGGAGCGATTTGTCTGG-3′

Antisense: 5′-AGGGCAGGGACTTAATCAACGC-3′

Atrial natriuretic peptide (ANP):

Sense:   5′-GTGCGGTGCCAACACAGAT-3′

Antisense: 5′-GCTTCCTCAGTCTGCTCACTCA-3′

CD14:

Sense:   5′-GGCGCTCCGAGTTGTGACT-3′

Antisense: 5′-TACCTGCTTCAGCCCAGTGA-3′

Glyceraldehyde-3-phosphate dehydrogenase (GAPDH):

Sense:   5′-CCAGCCTCGTCCCGTAGAC-3′

Antisense: 5′-ATGGCAACAATCTCCACTTTGC-3′

Interleukin 1 beta (IL-1β):

Sense:   5′-TCGTGCTGTCGGACCCATAT-3′

Antisense: 5′-GTCGTTGCTTGGTTCTCCTTGT-3′

Interleukin 6 (IL-6):

Sense:   5′-CCGGAGAGGAGACTTCACAG-3′

Antisense: 5′-TTCTGCAAGTGCATCATCGT-3′

Toll like receptor 4 (TLR4):

Sense:   5′-CTCTGCCTTCACTACAGAGAC-3′

Antisense: 5′-TGGATGATGTTGGCAGCAATG-3′

Tumor necrosis factor alpha (TNFα):

Sense:   5′-CACACTCAGATCATCTTCTCAAAA-3′

Antisense: 5′-GTAGACAAGGTACAACCCATCG-3′

### 4.5. Western Blot Analysis

The Western blot analysis of ventricular homogenates was performed as described previously [11,32,59]. Briefly, ventricular samples were pulverized under liquid nitrogen and 100 µL of homogenization buffer containing 10 mM NaHCO_3_ and 5% sodium dodecyl sulfate was added to about 10 mg tissue sample. The extracts were homogenized with an ultrasonic homogenizer (Sonopuls, 3× 30 s; Bandelin, Berlin, Germany), incubated at 25 °C for 30 min and then centrifuged to remove debris. The protein concentration was estimated by the Lowry method and aliquots of 100 µg protein were used for Western blotting. For detection of protein bands, the enhanced chemifluorescence together with alkaline phosphatase-conjugated secondary antibodies (Sigma-Aldrich, Munich, Germany) was used and the signals were recorded with a Typhoon 9410 Variable Mode Imager (GE Healthcare, Freiburg, Germany) and quantified with the ImageQuant TL software (GE Healthcare, Freiburg, Germany). The following primary antibodies were used: calsequestrin (CSQ: rabbit polyclonal [#SP5340P], Acris Antibodies, Herford, Germany [now available from abcam #ab3516]); glyceraldehyde-3-phosphate dehydrogenase (GAPDH; mouse monoclonal [#ab9484], abcam, Cambridge, MA, USA); super oxide dismutase 2 (SOD2: rabbit polyclonal [#SPC-118C/D], StressMarq Biosciences, Victoria, Canada); Ca2+ calmodulin kinase II (CamKII: rabbit monoclonal [#2048-1], Epitomics, Burlingame, CA, U.S.A.); heat shock protein 25 (HSP25: rabbit polyclonal [#ADI-SPA-801], Enzo Life Science, Lörrach, Germany); heat shock protein 90 (HSP90: rat monoclonal [#ADI-SPA-845], Enzo Life Science, Lörrach, Germany); aldehyde dehydrogenase 2 (Aldh2; goat polyclonal [#ABIN571181], antibodies-online, Aachen, Germany); endonuclease G (Endog; goat polyclonal [sc-26923], Santa Cruz Biotechnology, Heidelberg, Germany); cathepsin B (rabbit polyclonal [#ABIN1002042], antibodies-online, Aachen, Germany); enolase3 (=enolase beta; rabbit polyclonal [ABIN310998], antibodies-online, Aachen, Germany); nucleoporin 62kDa (Nup62; rabbit polyclonal [#ABIN1013745], antibodies-online, Aachen, Germany); protein phosphatase 5 (PP5: mouse monoclonal [#611021], BD Transduction Laboratories, Heidelberg, Germany); regulatory A-subunit of protein phosphatase 2A (PP2A-A: goat polyclonal [#sc-6113], Santa Cruz Biotechnology, Heidelberg, Germany); catalytic subunit of protein phosphatase 2A (PP2A-C: rabbit monoclonal [#ab32141], abcam, Berlin, Germany); catalytic subunit alpha of protein phosphatase 1 (PP1c; mouse monoclonal [#sc-7482], Santa Cruz Biotechnology, Heidelberg, Germany); nitric oxide synthase 1 to 3 (NOS 1; NOS 2; NOS 3; rabbit polyclonal [#610310; #610333; #610299], BD Transduction Laboratories, Heidelberg, Germany).

### 4.6. Echocardiography

For transthoracic echocardiography, a Vevo 2100 system with a MS550D transducer (Visual Sonics, Toronto, Canada) was used as described previously [18,59]. Briefly, the mice were anesthetized with 1.5% isoflurane (the spontaneous breathing was maintained) and fixed on a heated examination pad with integrated ECG electrodes. The cardiac dimensions were measured using two-dimensional images and M-mode tracings and as functional parameter, the ejection fraction of the hearts was calculated as described previously [18,59].

### 4.7. Work-Performing Heart Preparations

The technique of the isolated, perfused work-performing heart was performed as described previously [59]. Briefly, the mice were anesthetized with pentobarbital (80 mg kg^−1^) and treated with heparin (1.5 units). The hearts were prepared, attached by the aorta to a 20-gauge cannula, and fixed on a vertical Langendorff apparatus. The perfusion was performed with an oxygenized (carbogen gas = 95% O_2_, 5% CO_2_) Krebs Henseleit buffer (37.4 °C) containing (mM): NaCl 118, NaHCO_3_ 25, Na-EDTA 0.5, KCI 4.7, KH_2_PO_4_, 1.2, MgSO_4_, 1.2, CaCl_2_ 2.5, and glucose 11. Heart rate, aortic pressure, left intraventricular pressure, and left atrial pressure were recorded continuously using a PowerLab system (ADInstruments, Spechbach, Germany). From these data, the maxima of the first derivative of left intraventricular pressure (+dP/dt and −dP/dt) were calculated with the Chart software (ADInstruments, Spechbach, Germany).

### 4.8. NADPH Oxidase Activity

NADPH oxidase activity was measured in cardiac homogenates as previously described [60].

### 4.9. Immunohistochemistry

For immunohistological analysis of NOS expression, paraffin sections of hearts of mice were used as previously described [11]. The following antibodies were used: nitric oxide synthase 1 to 3 (NOS1; NOS2; NOS3; rabbit polyclonal [#610310; #610333; #610299], BD Transduction Laboratories, Heidelberg, Germany).

### 4.10. Reagents

LPS (E. coli O55:B5, #L2880) was purchased from Sigma-Aldrich (Munich, Germany). All chemicals used were of the highest purity grade commercially available and demineralized water was used throughout the experiments.

### 4.11. Statistics

The data are presented as means ± S.D. Statistical analyses were performed by the analysis of variance (ANOVA) followed by Bonferroni’s posttest or by the Student’s t-test if appropriate. As statistical significance level, a *p*-value < 0.05 was set. Statistical calculations and graphical presentations were carried out with GraphPad Prism 5.0 (GraphPad Software, San Diego, California, USA). Microarray data analysis was performed with the TM4 application MeV (https://mev.tm4.org (last accessed on 7 April 2022)) [57].

## Data Availability

The data presented in this study are available on request from the corresponding author.

## Supplementary Materials

The following supporting information can be downloaded at: https://www.mdpi.com/article/10.3390/ijms23094688/s1.
