# Supplementary material for: Protein Phosphatase 2A Improves Cardiac Functional Response to Ischemia and Sepsis
Source: Int J Mol Sci. 2022 Apr 23;23(9):4688. doi: 10.3390/ijms23094688 (PMC9101092; doi:10.3390/ijms23094688)
Supplement: Supplementary file 1 [file ijms-23-04688-s001.zip › Supplementary figures S1-S7.pdf]

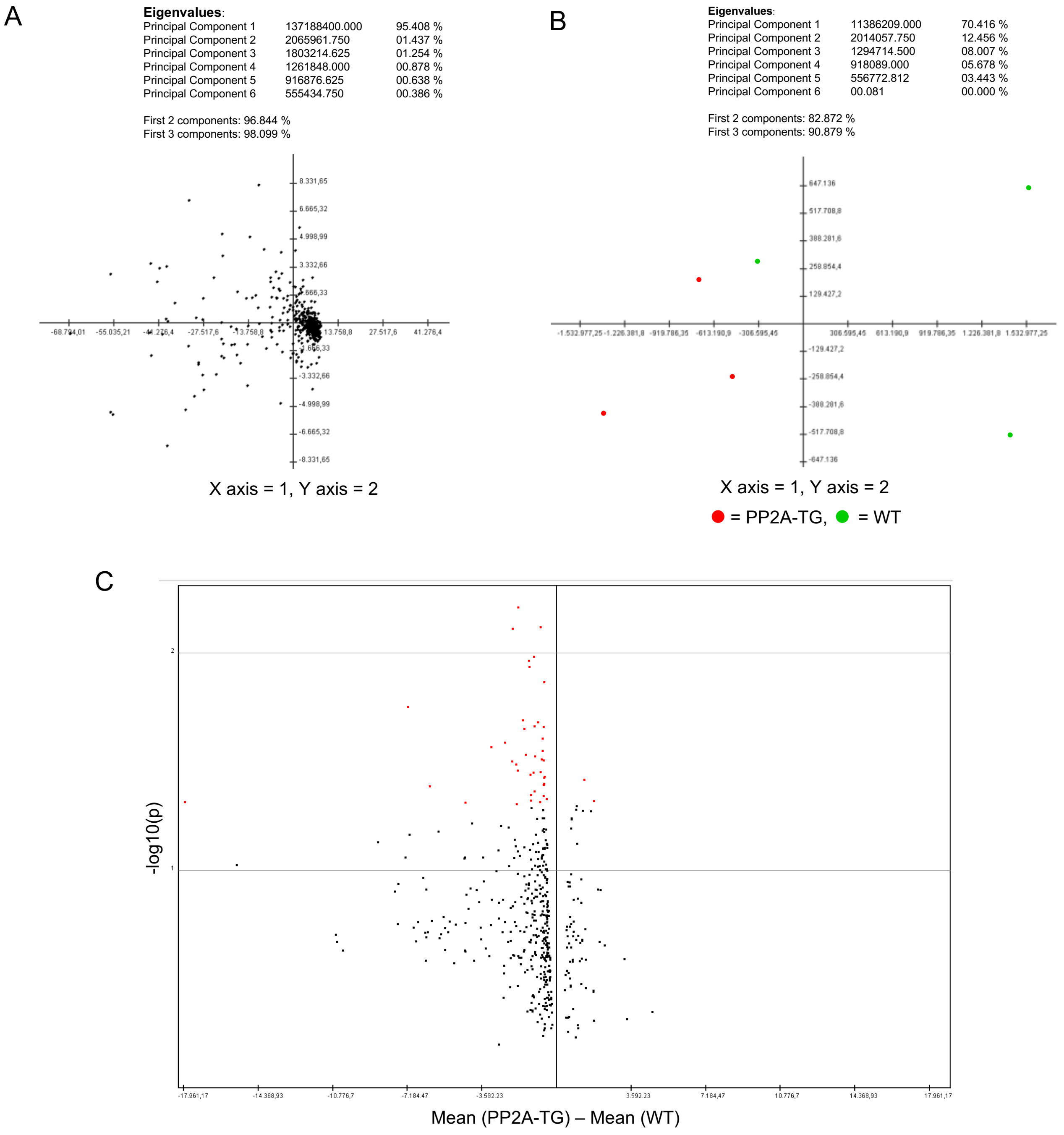

Supplementary Figure S1. A GeneChip Mouse Genome 430 2.0 Array (Affymetrix, Santa Clara, CA, United States) was used to analyze the basal mRNA expression in hearts from PP2A-TG (n=3) and WT (n=3) mice. For analysis, the TM4 application MeV was used (Saeed et al., 2003). Here, analysis was performed with genes that were expressed in both PP2A-TG and WT. First, data were adjusted by a variance filter (percent mode; value 50) leading to 454 relevant genes that were further analyzed. Secondly, a principal component analysis (PCA) was performed with the parameters: cluster genes (**A**) or cluster samples (**B**) for sample detection and mean for centering mode. Finally, WT and PP2A-TG were compared by a t-Test (Welch approximation; alpha (overall threshold p-value): 0.05; P-values based on t-distribution). An overview is given as volcano plot (**C**). Significant genes are marked by red color. More details are given in supplementary Table 1.

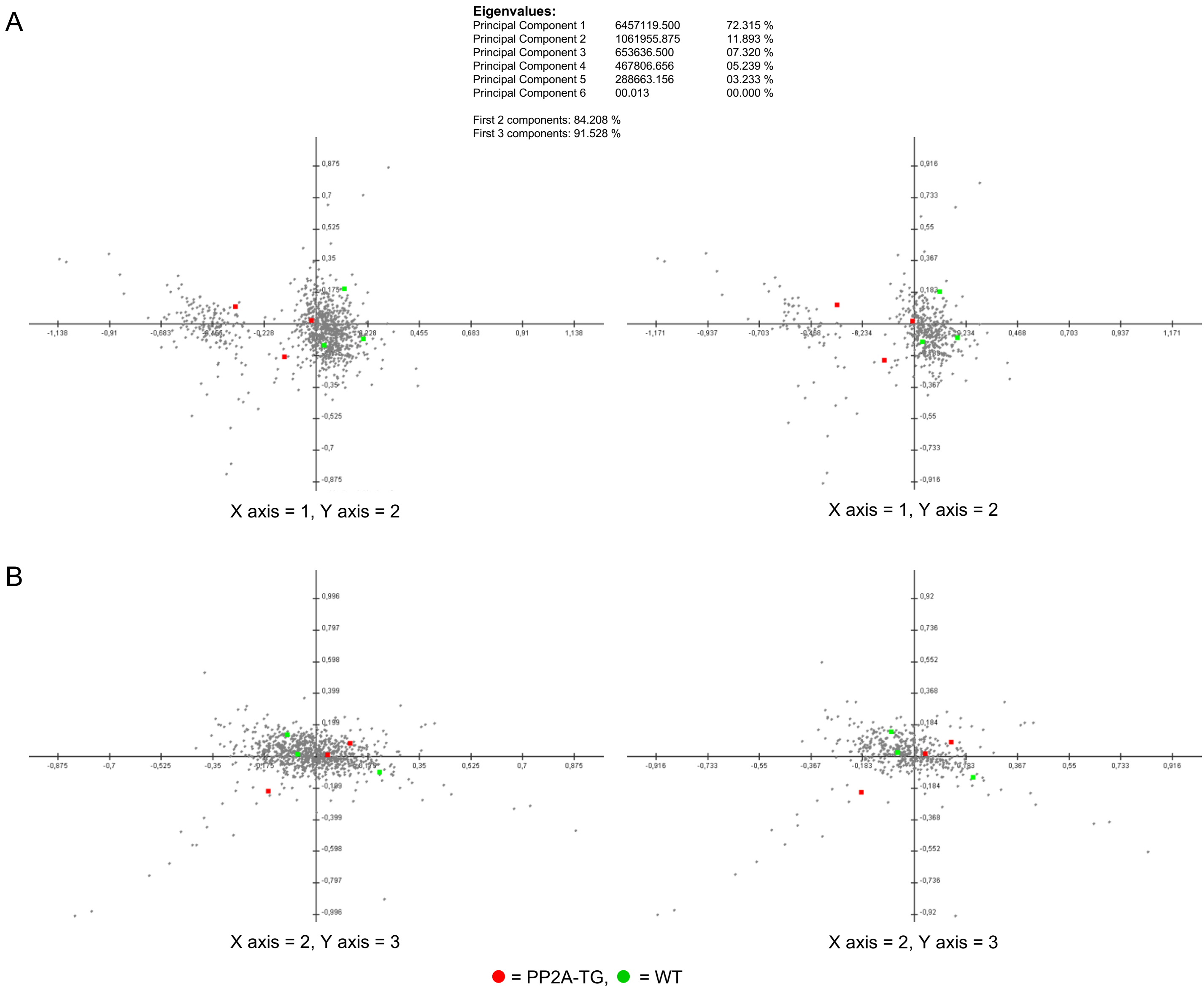

Supplementary Figure S2. A GeneChip Mouse Genome 430 2.0 Array (Affymetrix, Santa Clara, CA, United States) was used to analyze the basal mRNA expression in hearts from PP2A-TG (n=3) and WT (n=3) mice. For analysis, the TM4 application MeV was used (Saeed et al., 2003). Here, analysis was performed with genes that were expressed in both PP2A-TG and WT. The data were analyzed either without adjustment (**left side**) or they were adjusted by a variance filter (percent mode; value 50) leading to 454 relevant genes (**right side**) that were further analyzed. Than, a principal component analysis (PCA) was performed with the parameters: cluster samples for sample detection and mean for centering mode and finally, a correspondence analysis (COA) was performed. In (**A**), the COA for components 1 and 2 is plotted. In (**B**), the COA for components 2 and 3 is plotted.

A

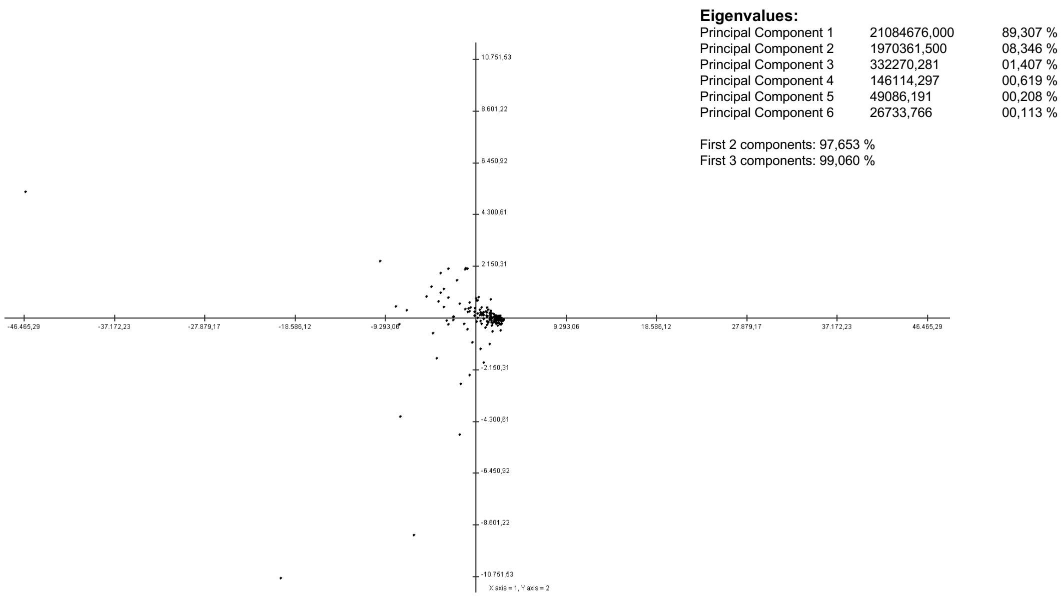

X axis = 1, Y axis = 2

B

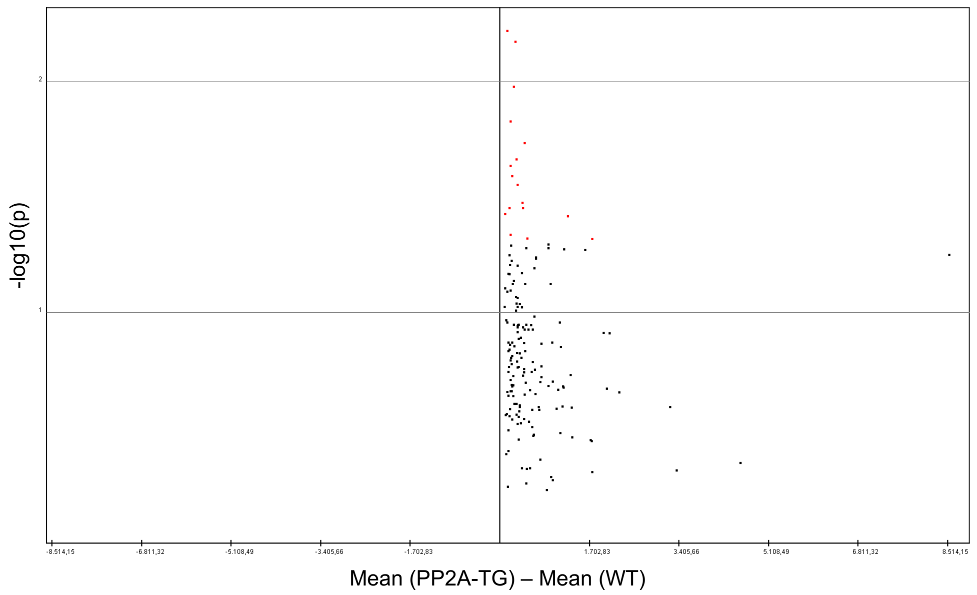

Supplementary Figure S3. A GeneChip Mouse Genome 430 2.0 Array (Affymetrix, Santa Clara, CA, United States) was used to analyze the basal mRNA expression in hearts from PP2A-TG (n=3) and WT (n=3) mice. For analysis, the TM4 application MeV was used (Saeed et al., 2003). Here, analysis was performed with **1.5-fold upregulated** genes that were expressed in both PP2A-TG and WT **without** adjustment. A principal component analysis (PCA) was performed (**A**) and WT and PP2A-TG were compared by a t-Test (Welch approximation; alpha (overall threshold p-value): 0.05; P-values based on t-distribution). An overview is given as volcano plot (**B**). Significant genes are marked by red color. More details are given in supplementary Table 1.

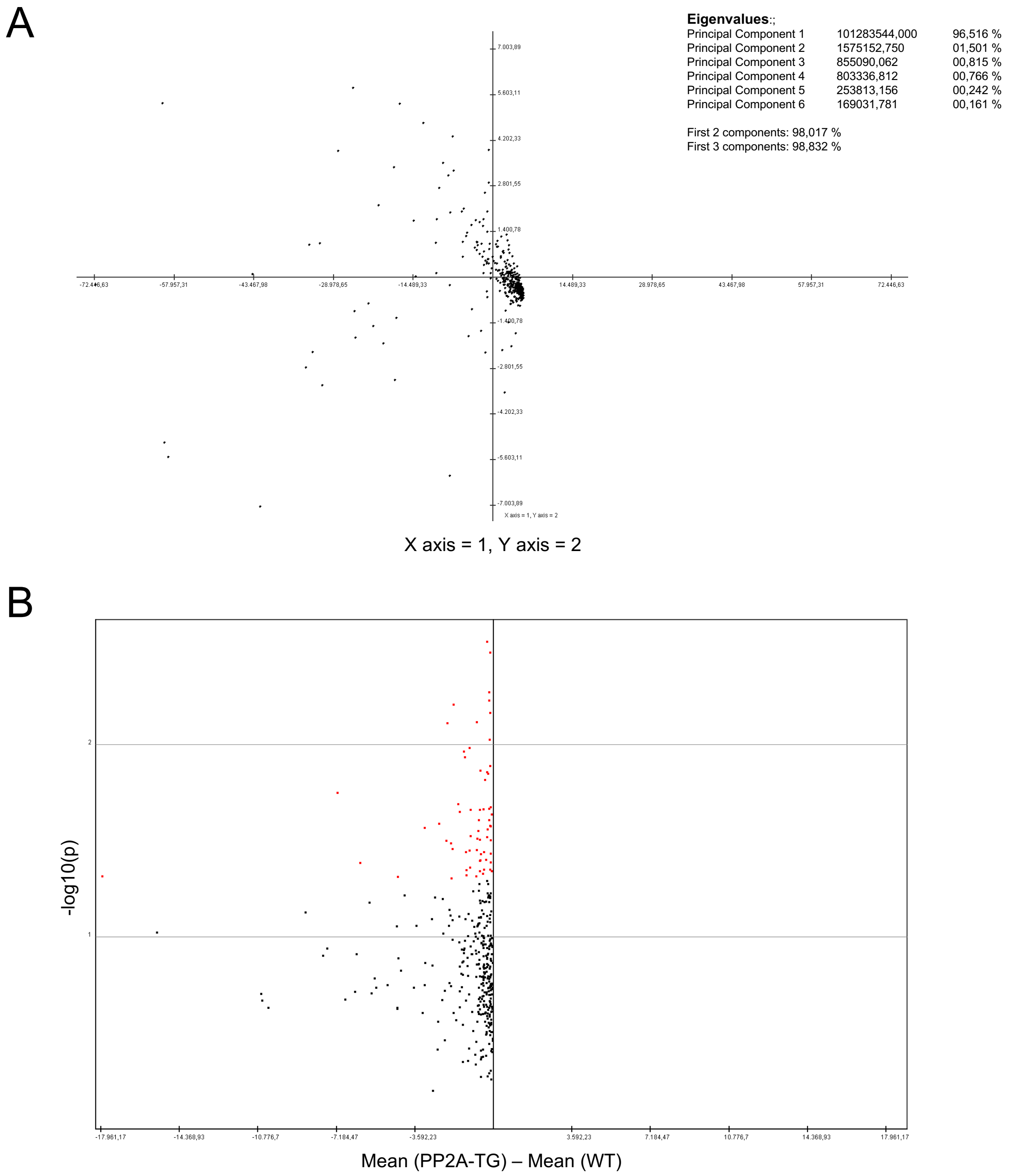

Supplementary Figure S4. A GeneChip Mouse Genome 430 2.0 Array (Affymetrix, Santa Clara, CA, United States) was used to analyze the basal mRNA expression in hearts from PP2A-TG (n=3) and WT (n=3) mice. For analysis, the TM4 application MeV was used (Saeed et al., 2003). Here, analysis was performed with **1.5-fold downregulated** genes that were expressed in both PP2A-TG and WT **without** adjustment. A principal component analysis (PCA) was performed (**A**) and WT and PP2A-TG were compared by a t-Test (Welch approximation; alpha (overall threshold p-value): 0.05; P-values based on t-distribution). An overview is given as volcano plot (**B**). Significant genes are marked by red color. More details are given in supplementary Table 2.

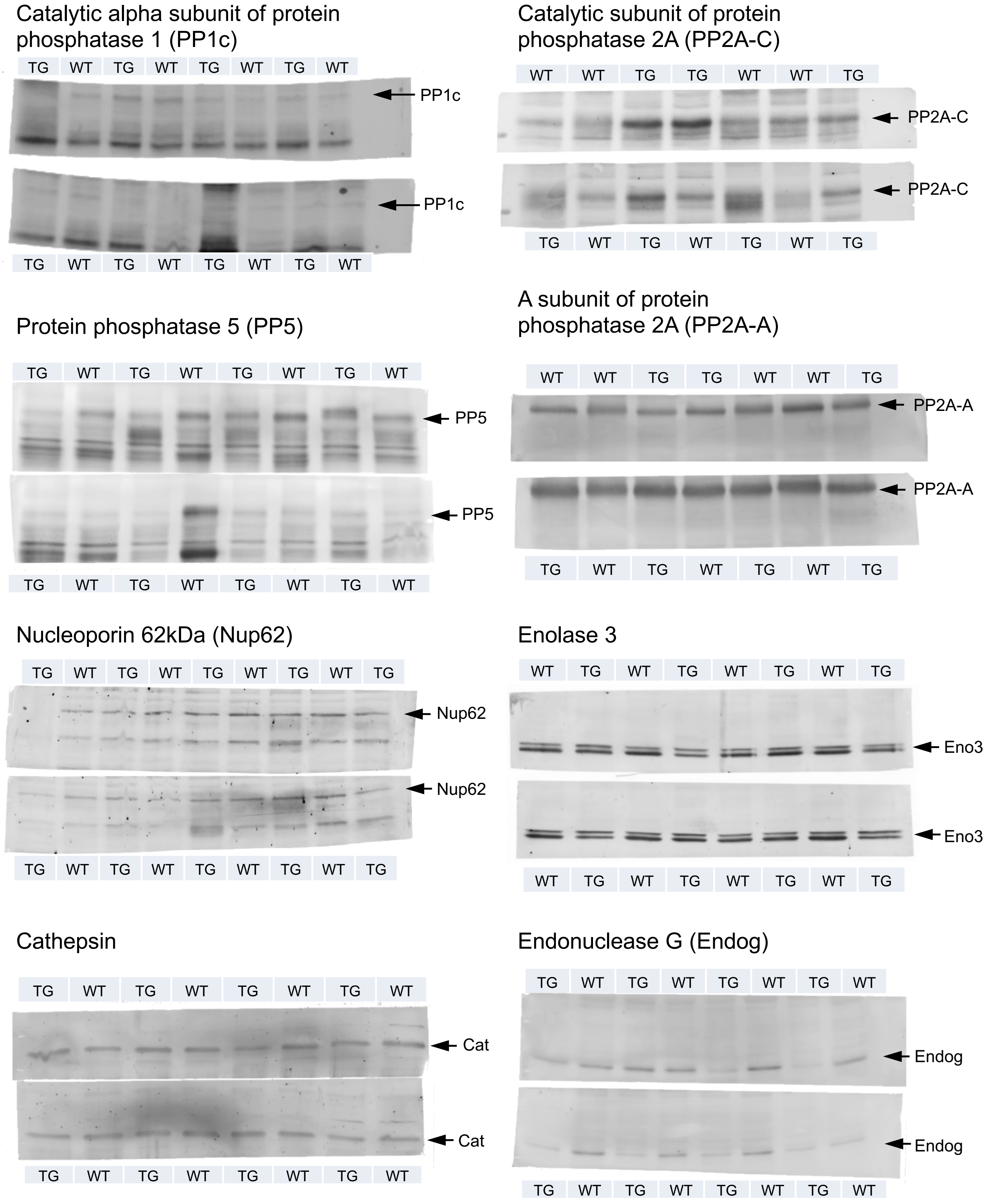

Supplementary Figure S5. Original Western blots (the region of interest was cut out before blotting) corresponding to Figure 2 are shown. WT, wild type samples; TG, PP2A-transgenic samples.

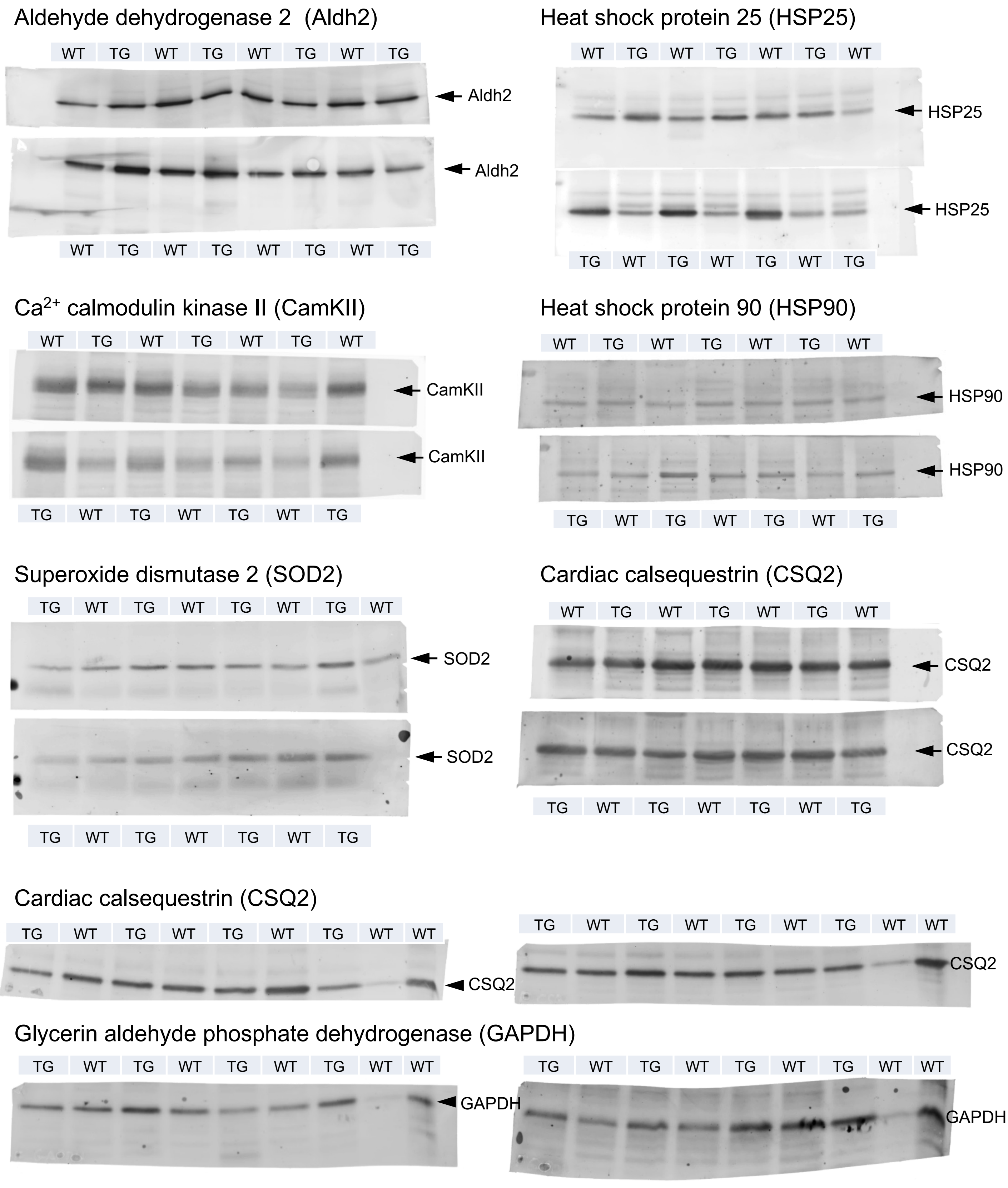

Supplementary Figure S6. Original Western blots (the region of interest was cut out before blotting) corresponding to Figure 2 are shown. WT, wild type samples; TG, PP2A-transgenic samples.

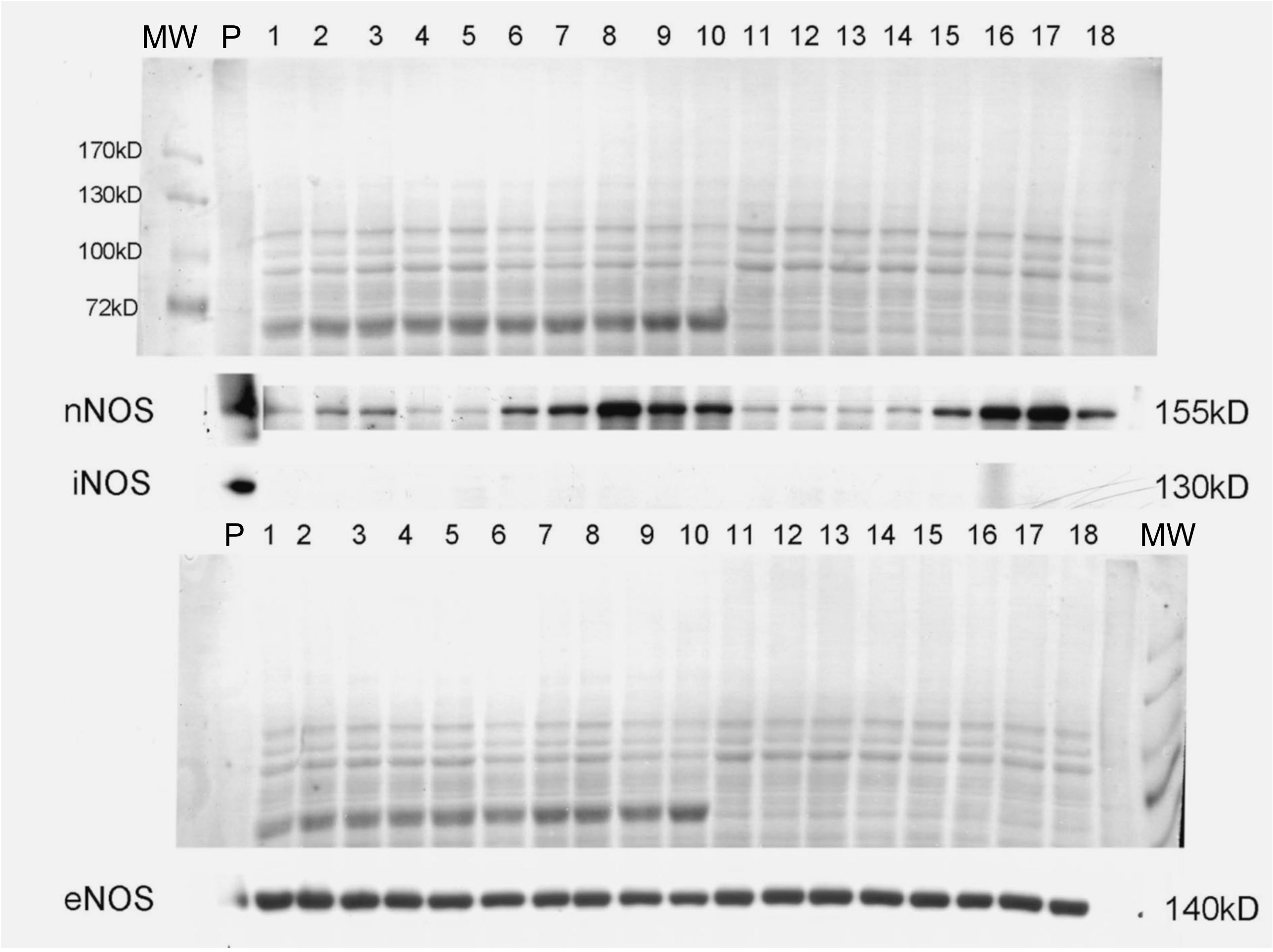

Supplementary Figure S7. The original nitrocellulose membranes (fast green staining) and Western blots (the region of interest was cut out before blotting) are shown for the expression of the neuronal, inducible and endothelial nitric oxidase synthase (nNOS, iNOS and eNOS) before ischemia (basal, left hand side: 1-10) and after 120 minutes of ischemia (right hand side, 11-18). The inducible NOS (iNOS) was not detectable in cardiac samples by Western blotting. Sample numbers 1-5 and 11-14 are wild type (WT) samples. Sample numbers 6-10 and 15-18 are PP2A-TG samples. P, positive control; MW, molecular weight marker.
