# Supplementary material for: Protein Phosphatase 2A Improves Cardiac Functional Response to Ischemia and Sepsis
Source: Int J Mol Sci. 2022 Apr 23;23(9):4688. doi: 10.3390/ijms23094688 (PMC9101092; doi:10.3390/ijms23094688)
Supplement: Supplementary file 1 [file ijms-23-04688-s001.zip › Supplementary Table S1.pdf]

Supplementary Table S1. Cardiac gene expression. A GeneChip Mouse Genome 430 2.0 Array (Affymetrix, Santa Clara, CA, United States) was used to analyze the basal mRNA expression in hearts from PP2A-TG (n=3) and WT (n=3) mice. Here, analysis was performed with genes that were expressed in both PP2A-TG and WT and with a 1.5-fold or higher up-regulation in PP2A-TG. For analysis, the TM4 application MeV was used (Saeed et al., 2003) and analyzed by a t-Test (Welch approximation; alpha (overall threshold p-value): 0.05; P-values based on t-distribution). Data are sorted by p value.

Gene descriptions	WT mean	WT SD	PP2A-TG mean	PP2A-TG SD	Absolute t value	Degrees of freedom	Adj p value	False discovery rate	PP2A-TG / WT
gb:AF071068.1 /DB_XREF=gi:3288844 /FEA=FLmRNA /CNT=74 /TID=Mm.12906.1 /TIER=FL+Stack /STK=59 /UG=Mm.12906 /LL=13195 /UG_GENE=Ddc /DEF=Mus musculus aromatic-L-amino-acid decarboxylase mRNA, complete cds. /PROD=aromatic-L-amino-acid decarboxylase /FL=gb:AF071068.1	177.93333	22.69303	295.16666	17.955597	7.0169954	3.0	0.005944862	1.0106266	1.66
gb:AK008813.1 /DB_XREF=gi:12843233 /FEA=mRNA /CNT=62 /TID=Mm.41393.1 /TIER=Stack /STK=32 /UG=Mm.41393 /LL=67098 /UG_GENE=2210403K04Rik /UG_TITLE=RIKEN cDNA 2210403K04 gene /DEF=Mus musculus adult male stomach cDNA, RIKEN full-length enriched library, clone:2210403K04:unclassifiable transcript, full insert sequence.	538.1	45.03732	806.39996	52.048145	6.7516923	3.0	0.006636761	0.5641247	1.50
gb:NM_133349.1 /DB_XREF=gi:19111157 /GEN=AA407930 /FEA=FLmRNA /CNT=202 /TID=Mm.24521.1 /TIER=FL+Stack /STK=164 /UG=Mm.24521 /LL=100494 /DEF=Mus musculus expressed sequence AA407930 (AA407930), mRNA. /PROD=arsenite inducible RNA associated protein /FL=gb:NM_133349.1 gb:AF224494.1	348.03333	37.002747	589.0	62.21969	5.765428	3.0	0.010371278	0.58770573	1.69
gb:NM_009697.1 /DB_XREF=gi:6753103 /GEN=Nr2f2 /FEA=FLmRNA /CNT=177 /TID=Mm.16519.1 /TIER=FL+Stack /STK=120 /UG=Mm.16519 /LL=11819 /DEF=Mus musculus nuclear receptor subfamily 2, group F, member 2 (Nr2f2), mRNA. /PROD=nuclear receptor subfamily 2, group F, member 2 /FL=gb:NM_009697.1 gb:U07635.1	273.56665	48.7658	451.30002	35.80963	5.0881906	3.0	0.014671077	0.62352073	1.65
gb:AK011810.1 /DB_XREF=gi:12848169 /FEA=FLmRNA /CNT=259 /TID=Mm.22676.1 /TIER=Stack /STK=13 /UG=Mm.22676 /LL=68263 /UG_GENE=Pdhb /UG_TITLE=pyruvate dehydrogenase (lipoamide) beta /DEF=Mus musculus 10 days embryo whole body cDNA, RIKEN full-length enriched library, clone:2610103L06:homolog to human PYRUVATE DEHYDROGENASE E1 COMPONENT BETA SUBUNIT, MITOCHONDRIAL PRECURSOR (EC 1.2.4.1) (PDHE1-B), full insert sequence. /FL=gb:BC019512.1 gb:NM_024221.1	428.06665	41.133972	867.96674	95.92052	7.3003874	2.0	0.018251156	0.6205393	2.03
gb:AK012898.1 /DB_XREF=gi:12849943 /FEA=mRNA /CNT=13 /TID=Mm.100168.1 /TIER=ConsEnd /STK=7 /UG=Mm.100168 /LL=72690 /UG_GENE=2810043G13Rik /UG_TITLE=RIKEN cDNA 2810043G13 gene /DEF=Mus musculus 10, 11 days embryo whole body cDNA, RIKEN full-length enriched library, clone:2810043G13:homolog to CDNA FLJ14050 FIS, CLONE HEMBA1006796, full insert sequence.	303.4	62.907635	591.0334	93.44459	4.4226418	3.0	0.021466253	0.6082105	1.95
gb:BB482460 /DB_XREF=gi:16440924 /DB_XREF=BB482460 /CLONE=D430013B11 /FEA=FLmRNA /CNT=77 /TID=Mm.35911.1 /TIER=Stack /STK=27 /UG=Mm.35911 /UG_TITLE=Mus musculus, Similar to hypothetical protein FLJ11896, clone MGC:27917 IMAGE:3581964, mRNA, complete cds /FL=gb:BC018458.1	279.56665	40.961243	458.83334	58.96186	4.324883	3.0	0.022787318	0.55340624	1.64
gb:NM_016777.2 /DB_XREF=gi:13384597 /GEN=Nasp /FEA=FLmRNA /CNT=221 /TID=Mm.7516.1 /TIER=FL+Stack /STK=139 /UG=Mm.7516 /LL=50927 /DEF=Mus musculus nuclear autoantigenic sperm protein (histone-binding) (Nasp), mRNA. /PROD=nuclear autoantigenic sperm protein(histone-binding) /FL=gb:NM_016777.2 gb:AF034610.2	313.0	39.067	515.39996	74.75647	4.1561475	3.0	0.02532544	0.53816557	1.65
gb:BC016565.1 /DB_XREF=gi:16741500 /FEA=FLmRNA /CNT=61 /TID=Mm.196517.1 /TIER=FL+Stack /STK=51 /UG=Mm.196517 /LL=66101 /UG_GENE=1100001J08Rik /DEF=Mus musculus, peptidyl prolyl isomerase H (cyclophilin H), clone MGC:29014 IMAGE:3157034, mRNA, complete cds. /PROD=peptidyl prolyl isomerase H (cyclophilin H) /FL=gb:BC016565.1	402.53333	31.306913	710.4666	84.90044	5.894173	2.0	0.027598174	0.5212988	1.76
gb:AB019577.1 /DB_XREF=gi:4760560 /GEN=ULK2 /FEA=FLmRNA /CNT=127 /TID=Mm.27664.1 /TIER=FL+Stack /STK=35 /UG=Mm.27664 /LL=29869 /DEF=Mus musculus mRNA for UNC-51-like kinase (ULK) 2, complete cds. /PROD=UNC-51-like kinase (ULK) 2 /FL=gb:AB019577.1 gb:NM_013881.1 gb:AF145922.1	765.1667	145.31139	1164.5665	112.95101	3.7587163	3.0	0.032918982	0.5596227	1.52
gb:W59564 /DB_XREF=gi:1366268 /DB_XREF=md72g04.r1 /CLONE=IMAGE:373974 /FEA=FLmRNA /CNT=62 /TID=Mm.30866.1 /TIER=ConsEnd /STK=0 /UG=Mm.30866 /UG_TITLE=Mus musculus, clone MGC:7403 IMAGE:3488364, mRNA, complete cds /FL=gb:BC003965.1	231.76666	8.855131	389.43335	51.549328	5.2211075	2.0	0.034781262	0.5375286	1.68
gb:BB252881 /DB_XREF=gi:16355965 /DB_XREF=BB252881 /CLONE=A730054K08 /FEA=EST /CNT=41 /TID=Mm.21114.2 /TIER=Stack /STK=20 /UG=Mm.21114 /UG_TITLE=Mus musculus, clone MGC:11980 IMAGE:3601364, mRNA, complete cds	765.3	164.06667	1179.9333	106.19165	3.6747198	3.0	0.034885198	0.49420694	1.54
gb:AF184112.1 /DB_XREF=gi:6007770 /GEN=Kid2 /FEA=FLmRNA /CNT=14 /TID=Mm.74670.1 /TIER=FL /STK=2 /UG=Mm.74670 /LL=27274 /DEF=Mus musculus KID2 (Kid2) mRNA, complete cds. /PROD=KID2 /FL=gb:NM_013744.1 gb:AF184112.1	126.73334	18.473314	201.73334	31.087677	3.592251	3.0	0.036964178	0.4833777	1.59
gb:NM_024225.1 /DB_XREF=gi:18034768 /GEN=Snx5 /FEA=FLmRNA /CNT=386 /TID=Mm.20847.1 /TIER=FL+Stack /STK=39 /UG=Mm.20847 /LL=69178 /DEF=Mus musculus sorting nexin 5 (Snx5), mRNA. /PROD=sorting nexin 5 /FL=gb:NM_024225.1 gb:BC002242.1	1845.6333	502.8171	3112.2	355.3908	3.562837	3.0	0.037743855	0.45831823	1.69
gb:AK004794.1 /DB_XREF=gi:12836243 /FEA=mRNA /CNT=30 /TID=Mm.50841.1 /TIER=Stack /STK=20 /UG=Mm.50841 /LL=71738 /UG_GENE=1200015L10Rik /UG_TITLE=RIKEN cDNA 1200015L10 gene /DEF=Mus musculus adult male lung cDNA, RIKEN full-length enriched library, clone:1200015L10:related to THYROID HORMONE-INDUCED PROTEIN B PRECURSOR, full insert sequence.	351.6	30.619608	529.36664	60.74538	4.526211	2.0	0.045506377	0.5157389	1.51
gb:AK007178.1 /DB_XREF=gi:12840577 /FEA=mRNA /CNT=31 /TID=Mm.33084.1 /TIER=Stack /STK=15 /UG=Mm.33084 /LL=66061 /UG_GENE=0610012D17Rik /UG_TITLE=RIKEN cDNA 0610012D17 gene /DEF=Mus musculus adult male testis cDNA, RIKEN full-length enriched library, clone:1700112I01:related to MR19,000 OUTER ARM DYNEIN LIGHT CHAIN, full insert sequence.	849.1667	184.6573	1347.8667	189.91486	3.2608945	3.0	0.047099724	0.5004346	1.59
gb:BC015254.1 /DB_XREF=gi:15929639 /FEA=FLmRNA /CNT=72 /TID=Mm.6522.1 /TIER=FL+Stack /STK=40 /UG=Mm.6522 /LL=12778 /UG_GENE=Cmkor1 /DEF=Mus musculus, chemokine orphan receptor 1, clone MGC:18378 IMAGE:4242244, mRNA, complete cds. /PROD=chemokine orphan receptor 1 /FL=gb:BC015254.1 gb:NM_007722.1 gb:AF000236.1	3317.8333	177.26184	5048.1665	652.2934	4.433797	2.0	0.04728927	0.4728927	1.52
gb:AK012776.1 /DB_XREF=gi:12849742 /FEA=mRNA /CNT=22 /TID=Mm.154145.2 /TIER=ConsEnd /STK=6 /UG=Mm.154145 /LL=66376 /UG_GENE=2310011F05Rik /UG_TITLE=RIKEN cDNA 2310011F05 gene /DEF=Mus musculus 10, 11 days embryo whole body cDNA, RIKEN full-length enriched library, clone:2810021G02:Zinc finger, C2H2 type containing protein, full insert sequence.	550.86664	266.142	1449.6334	410.57803	3.1815608	3.0	0.05003401	0.47254342	2.63
gb:AK013448.1 /DB_XREF=gi:12850809 /FEA=mRNA /CNT=4 /TID=Mm.45223.1 /TIER=ConsEnd /STK=3 /UG=Mm.45223 /LL=72819 /UG_GENE=2810487F15Rik /UG_TITLE=RIKEN cDNA 2810487F15 gene /DEF=Mus musculus 10, 11 days embryo whole body cDNA, RIKEN full-length enriched library, clone:2810487F15:homolog to SEROLOGICALLY DEFINED BREAST CANCER ANTIGEN NY-BR-81 (FRAGMENT), full insert sequence.	331.9	55.448353	518.8	85.94398	3.165087	3.0	0.05067204	0.4533814	1.56
gb:NM_024190.1 /DB_XREF=gi:13195617 /GEN=2810405I11Rik /FEA=FLmRNA /CNT=142 /TID=Mm.73777.1 /TIER=FL+Stack /STK=17 /UG=Mm.73777 /LL=67064 /DEF=Mus musculus RIKEN cDNA 2810405I11 gene (2810405I11Rik), mRNA. /PROD=RIKEN cDNA 2810405I11 /FL=gb:NM_024190.1 gb:BC002229.1	480.4	188.76564	948.6333	177.22948	3.1321821	3.0	0.051977485	0.4418086	1.97
gb:NM_013562.1 /DB_XREF=gi:7305178 /GEN=Ifrd1 /FEA=FLmRNA /CNT=225 /TID=Mm.168.1 /TIER=FL+Stack /STK=134 /UG=Mm.168 /LL=15982 /DEF=Mus musculus interferon-related developmental regulator 1 (Ifrd1), mRNA. /PROD=interferon-related developmental regulator 1 /FL=gb:NM_013562.1	1775.8002	335.17688	2668.5	362.82657	3.1302788	3.0	0.052054286	0.42139184	1.50
gb:NM_026252.1 /DB_XREF=gi:13385759 /GEN=4930447D24Rik /FEA=FLmRNA /CNT=161 /TID=Mm.23178.1 /TIER=ConsEnd /STK=0 /UG=Mm.23178 /LL=67579 /DEF=Mus musculus RIKEN cDNA 4930447D24 gene (4930447D24Rik), mRNA. /PROD=RIKEN cDNA 4930447D24 /FL=gb:NM_026252.1	2246.3333	522.5565	3440.5332	407.56915	3.12117	3.0	0.052423827	0.4050932	1.53
gb:U20344.1 /DB_XREF=gi:912487 /FEA=FLmRNA /CNT=140 /TID=Mm.4325.1 /TIER=FL+Stack /STK=46 /UG=Mm.4325 /LL=16600 /UG_GENE=Klf4 /DEF=Mus musculus gut-enriched Kruppel-like factor GKLF mRNA, complete cds. /PROD=gut-enriched Kruppel-like factor /FL=gb:BC010301.1 gb:U20344.1 gb:U70662.1 gb:NM_010637.1	2218.3	658.10254	3810.5332	593.3005	3.1124587	3.0	0.05278034	0.39011556	1.72
gb:BC003856.1 /DB_XREF=gi:13277983 /FEA=FLmRNA /CNT=288 /TID=Mm.11711.1 /TIER=FL+Stack /STK=46 /UG=Mm.11711 /LL=19052 /UG_GENE=Ppp2ca /DEF=Mus musculus, Protein phosphatase 2 (formerly 2A), catalytic subunit, alpha isoform, clone MGC:6583 IMAGE:3484084, mRNA, complete cds. /PROD=Protein phosphatase 2 (formerly 2A), catalyticsubunit, alpha isoform /FL=gb:NM_019411.1 gb:BC003856.1 gb:AF076192.1	15551.75	1277.25	24065.9	3395.3887	4.065121	2.0	0.05552183	0.3932796	1.55
gb:AK012533.1 /DB_XREF=gi:12849338 /FEA=mRNA /CNT=27 /TID=Mm.180363.2 /TIER=ConsEnd /STK=6 /UG=Mm.180363 /LL=66578 /UG_GENE=2610039C10Rik /UG_TITLE=RIKEN cDNA 2610039C10 gene /DEF=Mus musculus 11 days embryo whole body cDNA, RIKEN full-length enriched library, clone:2700078I24:homolog to PUTATIVE PROTEIN C21ORF45, full insert sequence.	201.76666	19.207373	359.70004	64.7058	4.0527883	2.0	0.055832442	0.37966058	1.78
gb:NM_016715.1 /DB_XREF=gi:7710051 /GEN=Tslpr /FEA=FLmRNA /CNT=43 /TID=Mm.35771.1 /TIER=FL+Stack /STK=30 /UG=Mm.35771 /LL=57914 /DEF=Mus musculus thymic stromal-derived lymphopoietin, receptor (Tslpr), mRNA. /PROD=thymic stromal-derived lymphopoietin, receptor /FL=gb:AF232936.1 gb:AB031333.1 gb:AB039945.1 gb:NM_016715.1	912.63336	302.9743	1570.6333	225.301	3.0185394	3.0	0.05682495	0.37154773	1.72
gb:BC019459.1 /DB_XREF=gi:18043385 /FEA=mRNA /CNT=128 /TID=Mm.31025.1 /TIER=Stack /STK=66 /UG=Mm.31025 /LL=76437 /UG_GENE=2310015K15Rik /UG_TITLE=RIKEN cDNA 2310015K15 gene /DEF=Mus musculus, Similar to RIKEN cDNA 2310015K15 gene, clone IMAGE:4035590, mRNA.	682.73334	297.8792	1340.9333	235.16748	3.003885	3.0	0.057490736	0.3619787	1.96
gb:AK013519.1 /DB_XREF=gi:12850916 /FEA=mRNA /CNT=51 /TID=Mm.38442.1 /TIER=Stack /STK=13 /UG=Mm.38442 /LL=72910 /UG_GENE=2900010L19Rik /UG_TITLE=RIKEN cDNA 2900010L19 gene /DEF=Mus musculus adult male hippocampus cDNA, RIKEN full-length enriched library, clone:2900010L19:unclassifiable, full insert sequence.	270.96667	80.71471	465.7667	28.976597	3.9343488	2.0	0.058948655	0.35790253	1.72
gb:NM_011857.2 /DB_XREF=gi:7657416 /GEN=Odz3 /FEA=FLmRNA /CNT=38 /TID=Mm.42191.1 /TIER=FL+Stack /STK=15 /UG=Mm.42191 /LL=23965 /DEF=Mus musculus odd Ozten-m homolog 3 (Drosophila) (Odz3), mRNA. /PROD=odd Ozten-m homolog 3 (Drosophila) /FL=gb:AB025412.1 gb:NM_011857.2	287.53333	60.616524	455.2667	78.964325	2.9184296	3.0	0.06157439	0.3609533	1.58
gb:BB559624 /DB_XREF=gi:9645990 /DB_XREF=BB559624 /CLONE=E330039L20 /FEA=EST /CNT=48 /TID=Mm.68427.2 /TIER=Stack /STK=48 /UG=Mm.68427 /UG_TITLE=Mus musculus, Similar to nucleoporin 133kD, clone MGC:25929 IMAGE:4235408, mRNA, complete cds	505.0	162.59068	816.03326	87.627	2.9167533	3.0	0.06165807	0.34939572	1.62
gb:NM_013692.1 /DB_XREF=gi:7305570 /GEN=Tieg /FEA=FLmRNA /CNT=184 /TID=Mm.4292.1 /TIER=FL+Stack /STK=141 /UG=Mm.4292 /LL=21847 /DEF=Mus musculus TGFB inducible early growth response (Tieg), mRNA. /PROD=TGFB inducible early growth response /FL=gb:AF064088.1 gb:BC003316.1 gb:NM_013692.1 gb:AF049879.1	528.2	266.4009	1155.1333	107.51492	3.7798882	2.0	0.06340678	0.3477146	2.19
gb:BC024718.1 /DB_XREF=gi:19354355 /FEA=FLmRNA /CNT=185 /TID=Mm.21534.1 /TIER=FL+Stack /STK=32 /UG=Mm.21534 /LL=67781 /UG_GENE=Ilf2 /DEF=Mus musculus, Similar to interleukin enhancer binding factor 2, 45kD, clone MGC:30414 IMAGE:5012365, mRNA, complete cds. /PROD=Similar to interleukin enhancer binding factor2, 45kD /FL=gb:BC024718.1 gb:NM_026374.1	446.19998	54.47163	836.1667	175.51302	3.6754448	2.0	0.066703826	0.35436407	1.87
gb:NM_138758.1 /DB_XREF=gi:20270296 /GEN=Tmlh /FEA=FLmRNA /CNT=8 /TID=Mm.139078.1 /TIER=FL /STK=3 /UG=Mm.139078 /LL=192289 /DEF=Mus musculus epsilon-trimethyllysine 2-oxoglutarate dioxygenase (Tmlh), mRNA. /PROD=epsilon-trimethyllysine 2-oxoglutaratedioxygenase /FL=gb:NM_138758.1 gb:AY033513.1 gb:BC010495.1	184.23334	58.77707	321.13333	60.226597	2.8176465	3.0	0.06686834	0.34447327	1.74
gb:AK013414.1 /DB_XREF=gi:12850758 /GEN=Sip1 /FEA=FLmRNA /CNT=43 /TID=Mm.35353.1 /TIER=Stack /STK=9 /UG=Mm.35353 /LL=66603 /UG_TITLE=survivor of motor neuron protein interacting protein 1 /DEF=Mus musculus 10, 11 days embryo whole body cDNA, RIKEN full-length enriched library, clone:2810477M04:survivor of motor neuron protein interacting protein 1, full insert sequence. /FL=gb:NM_025656.1	197.16667	70.14387	348.16666	61.384792	2.805894	3.0	0.067522064	0.3376103	1.77
gb:NM_026139.1 /DB_XREF=gi:13385647 /GEN=3230401N03Rik /FEA=FLmRNA /CNT=415 /TID=Mm.27714.1 /TIER=FL+Stack /STK=348 /UG=Mm.27714 /LL=67416 /DEF=Mus musculus RIKEN cDNA 3230401N03 gene (3230401N03Rik), mRNA. /PROD=RIKEN cDNA 3230401N03 /FL=gb:NM_026139.1	373.7333	115.7666	615.1667	100.210815	2.7311215	3.0	0.07187284	0.34909663	1.65
gb:NM_011240.1 /DB_XREF=gi:19882198 /GEN=Ranbp2 /FEA=FLmRNA /CNT=126 /TID=Mm.142730.1 /TIER=FL+Stack /STK=25 /UG=Mm.142730 /LL=19386 /DEF=Mus musculus RAN binding protein 2 (Ranbp2), mRNA. /PROD=RAN binding protein 2 /FL=gb:NM_011240.1	853.6667	134.80788	1304.4332	256.6447	2.6932094	3.0	0.07421185	0.35044485	1.53
gb:NM_008316.1 /DB_XREF=gi:6680330 /GEN=Hus1 /FEA=FLmRNA /CNT=52 /TID=Mm.42201.1 /TIER=FL+Stack /STK=26 /UG=Mm.42201 /LL=15574 /DEF=Mus musculus Hus1 homolog (S. pombe) (Hus1), mRNA. /PROD=Hus1 homolog (S. pombe) /FL=gb:NM_008316.1	360.73337	95.325775	571.63336	96.54104	2.692425	3.0	0.07426123	0.34120026	1.58
gb:NM_133721.1 /DB_XREF=gi:19526903 /GEN=Itga9 /FEA=FLmRNA /CNT=110 /TID=Mm.23386.1 /TIER=FL /STK=2 /UG=Mm.23386 /LL=70107 /DEF=Mus musculus integrin alpha 9 (Itga9), mRNA. /PROD=integrin alpha 9 /FL=gb:NM_133721.1	1774.6666	433.60614	2705.9	413.57755	2.691753	3.0	0.074303575	0.33241072	1.52
gb:BC003834.1 /DB_XREF=gi:13277929 /FEA=FLmRNA /CNT=83 /TID=Mm.4465.2 /TIER=FL+Stack /STK=12 /UG=Mm.4465 /LL=30050 /UG_GENE=Fbxw2 /DEF=Mus musculus, f-box and WD-40 domain protein 2, clone MGC:6266 IMAGE:3601733, mRNA, complete cds. /PROD=f-box and WD-40 domain protein 2 /FL=gb:BC003834.1	122.23334	39.00543	191.73334	23.556812	2.641773	3.0	0.07753805	0.33798638	1.57
gb:AV301675 /DB_XREF=gi:16393417 /DB_XREF=AV301675 /CLONE=5730495D10 /FEA=EST /CNT=21 /TID=Mm.16925.2 /TIER=Stack /STK=10 /UG=Mm.16925 /UG_TITLE=Mus musculus, Similar to HSPC144 protein, clone MGC:27728 IMAGE:2631627, mRNA, complete cds	278.83334	86.362976	455.83334	31.404675	3.3360977	2.0	0.079309046	0.33706343	1.63
gb:BC027185.1 /DB_XREF=gi:20072611 /FEA=FLmRNA /CNT=22 /TID=Mm.26580.1 /TIER=FL+Stack /STK=10 /UG=Mm.26580 /DEF=Mus musculus, clone MGC:28542 IMAGE:4194872, mRNA, complete cds. /PROD=Unknown (protein for MGC:28542) /FL=gb:BC027185.1	211.13335	57.66648	321.83334	2.3028996	3.3222992	2.0	0.07989219	0.3312603	1.52
gb:NM_019666.1 /DB_XREF=gi:13242327 /GEN=Nsap1l-pending /FEA=FLmRNA /CNT=67 /TID=Mm.196461.1 /TIER=FL+Stack /STK=13 /UG=Mm.196461 /LL=56393 /DEF=Mus musculus NS1-associated protein 1-like (Nsap1l-pending), mRNA. /PROD=NS1-associated protein 1-like /FL=gb:NM_019666.1 gb:AF093821.1	368.80002	103.69938	641.4667	154.10506	2.5425587	3.0	0.08449029	0.3419845	1.74
gb:BC003267.1 /DB_XREF=gi:13096942 /FEA=FLmRNA /CNT=17 /TID=Mm.102799.1 /TIER=FL /STK=7 /UG=Mm.102799 /DEF=Mus musculus, Similar to zinc finger protein 14 (KOX 6), clone MGC:7772 IMAGE:3499094, mRNA, complete cds. /PROD=Similar to zinc finger protein 14 (KOX 6) /FL=gb:BC003267.1	292.43332	50.514977	598.26666	157.72609	3.19844	2.0	0.08541378	0.33768237	2.05
gb:NM_028680.1 /DB_XREF=gi:20127154 /GEN=4833420A15Rik /FEA=FLmRNA /CNT=39 /TID=Mm.31355.1 /TIER=FL+Stack /STK=16 /UG=Mm.31355 /LL=73916 /DEF=Mus musculus RIKEN cDNA 4833420A15 gene (4833420A15Rik), mRNA. /PROD=huntingtin-interacting protein-1 proteininteractor /FL=gb:NM_028680.1 gb:AF274590.1	437.80002	60.285404	730.39996	151.9152	3.100824	2.0	0.09015908	0.34834188	1.67
gb:NM_010247.1 /DB_XREF=gi:6753927 /GEN=G22p1 /FEA=FLmRNA /CNT=76 /TID=Mm.16147.1 /TIER=FL+Stack /STK=45 /UG=Mm.16147 /LL=14375 /DEF=Mus musculus thyroid autoantigen 70 kDa (G22p1), mRNA. /PROD=thyroid autoantigen 70 kDa /FL=gb:AF483501.1 gb:AF483500.1 gb:NM_010247.1 gb:AB010282.1	648.2	195.2047	997.9333	149.58192	2.4631624	3.0	0.09061262	0.34231433	1.54
gb:BB702602 /DB_XREF=gi:16051426 /DB_XREF=BB702602 /CLONE=7420442K05 /FEA=mRNA /CNT=74 /TID=Mm.220922.1 /TIER=Stack /STK=16 /UG=Mm.220922 /UG_TITLE=Mus musculus, c-myc binding protein, clone MGC:13820 IMAGE:3985166, mRNA, complete cds	564.4	163.77605	871.0	60.70198	3.0403998	2.0	0.09328734	0.34475756	1.54
gb:NM_007825.1 /DB_XREF=gi:6681126 /GEN=Cyp7b1 /FEA=FLmRNA /CNT=26 /TID=Mm.4781.1 /TIER=FL /STK=2 /UG=Mm.4781 /LL=13123 /DEF=Mus musculus cytochrome P450, 7b1 (Cyp7b1), mRNA. /PROD=cytochrome P450, 7b1 /FL=gb:NM_007825.1 gb:U36993.1	131.56667	37.06067	196.73334	2.2678947	3.039914	2.0	0.09331312	0.33751553	1.50
gb:AK013419.1 /DB_XREF=gi:12850767 /FEA=mRNA /CNT=1 /TID=Mm.42154.3 /TIER=ConsEnd /STK=0 /UG=Mm.42154 /LL=56515 /UG_GENE=Trif-pending /UG_TITLE=Trif gene /DEF=Mus musculus 10, 11 days embryo whole body cDNA, RIKEN full-length enriched library, clone:2810480D20:Trif gene, full insert sequence.	636.0	222.4028	1026.1666	167.50089	2.4271975	3.0	0.093564205	0.3313732	1.61
gb:BC021511.1 /DB_XREF=gi:18204504 /FEA=mRNA /CNT=42 /TID=Mm.34850.1 /TIER=Stack /STK=13 /UG=Mm.34850 /DEF=Mus musculus, clone IMAGE:5347423, mRNA, partial cds. /PROD=Unknown (protein for IMAGE:5347423)	396.6	143.11713	673.0	73.22752	2.9779143	2.0	0.096687734	0.33544722	1.70
gb:AK004745.1 /DB_XREF=gi:12836150 /FEA=mRNA /CNT=188 /TID=Mm.26696.1 /TIER=Stack /STK=47 /UG=Mm.26696 /LL=74132 /UG_GENE=1200013I08Rik /UG_TITLE=RIKEN cDNA 1200013I08 gene /DEF=Mus musculus adult male lung cDNA, RIKEN full-length enriched library, clone:1200013I08:homolog to RING FINGER PROTEIN 6, full insert sequence.	1038.2334	374.87088	1668.3667	59.046204	2.8760056	2.0	0.10262341	0.34891957	1.61
gb:AK006234.1 /DB_XREF=gi:12839219 /FEA=mRNA /CNT=24 /TID=Mm.41534.1 /TIER=Stack /STK=12 /UG=Mm.41534 /LL=66336 /UG_GENE=1700022C02Rik /UG_TITLE=RIKEN cDNA 1700022C02 gene /DEF=Mus musculus adult male testis cDNA, RIKEN full-length enriched library, clone:1700022C02:hypothetical protein, full insert sequence.	173.7	10.578748	265.43335	55.55415	2.8095508	2.0	0.10677628	0.35592094	1.53
gb:NM_130889.1 /DB_XREF=gi:18700031 /GEN=Anp32b /FEA=FLmRNA /CNT=202 /TID=Mm.154985.1 /TIER=FL+Stack /STK=57 /UG=Mm.154985 /LL=67628 /DEF=Mus musculus acidic nuclear phosphoprotein 32 family, member B (Anp32b), mRNA. /PROD=acidic nuclear phosphoprotein 32 family, memberB /FL=gb:BC005628.1 gb:BC003489.1 gb:NM_130889.1 gb:AB025582.1	1617.6666	659.1917	2732.2334	218.32278	2.7800574	2.0	0.10869584	0.35535178	1.69
gb:NM_008990.1 /DB_XREF=gi:6679574 /GEN=Pvs /FEA=FLmRNA /CNT=59 /TID=Mm.4341.1 /TIER=FL+Stack /STK=38 /UG=Mm.4341 /LL=19294 /DEF=Mus musculus poliovirus sensitivity (Pvs), mRNA. /PROD=poliovirus sensitivity /FL=gb:NM_008990.1 gb:D26107.1	181.46667	20.528595	294.36667	67.33983	2.7777016	2.0	0.10885127	0.34914556	1.62
gb:BC005646.1 /DB_XREF=gi:13542905 /FEA=FLmRNA /CNT=157 /TID=Mm.30177.1 /TIER=FL+Stack /STK=21 /UG=Mm.30177 /LL=67191 /UG_GENE=2700047N05Rik /DEF=Mus musculus, RIKEN cDNA 2700047N05 gene, clone MGC:12088 IMAGE:3708975, mRNA, complete cds. /PROD=RIKEN cDNA 2700047N05 gene /FL=gb:NM_026023.1 gb:BC005646.1	248.93335	101.647934	488.73337	155.30946	2.2376606	3.0	0.11120266	0.35008243	1.96
gb:NM_025860.1 /DB_XREF=gi:13385341 /GEN=2310005B10Rik /FEA=FLmRNA /CNT=213 /TID=Mm.44219.1 /TIER=FL+Stack /STK=133 /UG=Mm.44219 /LL=66942 /DEF=Mus musculus RIKEN cDNA 2310005B10 gene (2310005B10Rik), mRNA. /PROD=RIKEN cDNA 2310005B10 /FL=gb:NM_025860.1	628.06665	225.23944	957.5334	120.43054	2.234228	3.0	0.111557566	0.34481427	1.52
gb:BC003916.1 /DB_XREF=gi:13278141 /FEA=FLmRNA /CNT=88 /TID=Mm.28679.1 /TIER=FL+Stack /STK=10 /UG=Mm.28679 /LL=67669 /UG_GENE=0610007P06Rik /DEF=Mus musculus, RIKEN cDNA 0610007P06 gene, clone MGC:7275 IMAGE:3485003, mRNA, complete cds. /PROD=RIKEN cDNA 0610007P06 gene /FL=gb:NM_026304.1 gb:BC003916.1	797.39996	238.46638	1268.1666	276.37323	2.2337542	3.0	0.11160665	0.33880588	1.59
gb:AK017551.1 /DB_XREF=gi:12856844 /FEA=mRNA /CNT=61 /TID=Mm.12648.1 /TIER=Stack /STK=16 /UG=Mm.12648 /LL=70492 /UG_GENE=5730412B09Rik /UG_TITLE=RIKEN cDNA 5730412B09 gene /DEF=Mus musculus 8 days embryo whole body cDNA, RIKEN full-length enriched library, clone:5730412B09:homolog to CDNA: FLJ21133 FIS, CLONE CAS07019, full insert sequence.	458.6333	142.55289	772.7666	197.81895	2.2314405	3.0	0.11184675	0.33357802	1.68

gb:NM_013469.1 /DB_XREF=gi:7304884 /GEN=Anxa11 /FEA=FLmRNA /CNT=81 /TID=Mm.1427.1 /TIER=FL+Stack /STK=23 /UG=Mm.1427 /LL=11744 /DEF=Mus musculus annexin A11 (Anxa11), mRNA. /PROD=annexin A11 /FL=gb:U65986.1 gb:BC012875.1 gb:NM_013469.1	1036.0	315.00784	1605.0668	310.22586	2.2293785	3.0	0.11206127	0.32845545	1.55
gb:NM_007945.1 /DB_XREF=gi:6679672 /GEN=Eps8 /FEA=FLmRNA /CNT=85 /TID=Mm.2012.1 /TIER=FL+Stack /STK=10 /UG=Mm.2012 /LL=13860 /DEF=Mus musculus epidermal growth factor receptor pathway substrate 8 (Eps8), mRNA. /PROD=epidermal growth factor receptor pathwaysubstrate 8 /FL=gb:L21671.1 gb:NM_007945.1	587.9667	173.123	892.56665	87.60487	2.71913	2.0	0.11281872	0.3250709	1.52
gb:NM_011997.1 /DB_XREF=gi:6753287 /GEN=Casp8ap2 /FEA=FLmRNA /CNT=64 /TID=Mm.22279.1 /TIER=FL+Stack /STK=18 /UG=Mm.22279 /LL=26885 /DEF=Mus musculus caspase 8 associated protein 2 (Casp8ap2), mRNA. /PROD=caspase 8 associated protein 2 /FL=gb:NM_011997.1 gb:AF132726.1	546.56665	199.0324	850.76666	129.13873	2.2207594	3.0	0.1129633	0.32006267	1.56
gb:NM_130449.1 /DB_XREF=gi:18485493 /GEN=Colec12 /FEA=FLmRNA /CNT=33 /TID=Mm.218571.1 /TIER=FL /STK=7 /UG=Mm.218571 /LL=140792 /DEF=Mus musculus collectin sub-family member 12 (Colec12), mRNA. /PROD=collectin sub-family member 12 /FL=gb:NM_130449.1 gb:AB038519.1	417.4	243.04816	826.4666	100.272255	2.6948278	2.0	0.114524886	0.3191677	1.98
gb:NM_007960.1 /DB_XREF=gi:6679700 /GEN=Etv1 /FEA=FLmRNA /CNT=157 /TID=Mm.4866.1 /TIER=FL+Stack /STK=27 /UG=Mm.4866 /LL=14009 /DEF=Mus musculus ets variant gene 1 (Etv1), mRNA. /PROD=ets variant gene 1 /FL=gb:BC005645.1 gb:L10426.1 gb:NM_007960.1	450.6	188.40437	759.4333	152.95242	2.2042534	3.0	0.11471522	0.31454173	1.69
gb:NM_008379.1 /DB_XREF=gi:6680448 /GEN=Impnb /FEA=FLmRNA /CNT=313 /TID=Mm.16710.1 /TIER=FL+Stack /STK=42 /UG=Mm.16710 /LL=16211 /DEF=Mus musculus importin beta (Impnb), mRNA. /PROD=importin beta /FL=gb:D45836.1 gb:NM_008379.1 gb:D67015.1	976.60004	305.5066	1489.2334	132.98216	2.6648316	2.0	0.116681345	0.3148544	1.52
gb:NM_007964.1 /DB_XREF=gi:6679706 /GEN=Evi5 /FEA=FLmRNA /CNT=132 /TID=Mm.35796.1 /TIER=FL+Stack /STK=43 /UG=Mm.35796 /LL=14020 /DEF=Mus musculus ecotropic viral integration site 5 (Evi5), mRNA. /PROD=ecotropic viral integration site 5 /FL=gb:U53586.1 gb:NM_007964.1	792.3667	377.52588	1390.3002	92.49189	2.6644602	2.0	0.11670841	0.3100067	1.75
gb:NM_009037.1 /DB_XREF=gi:6677690 /GEN=Rcn /FEA=FLmRNA /CNT=95 /TID=Mm.4876.1 /TIER=FL+Stack /STK=57 /UG=Mm.4876 /LL=19672 /DEF=Mus musculus reticulocalbin (Rcn), mRNA. /PROD=reticulocalbin /FL=gb:NM_009037.1 gb:D13003.1	772.5334	284.04846	1217.5333	209.04294	2.1854506	3.0	0.116750866	0.30534843	1.58
gb:AK014605.1 /DB_XREF=gi:12852564 /FEA=FLmRNA /CNT=129 /TID=Mm.28337.1 /TIER=ConsEnd /STK=5 /UG=Mm.28337 /LL=67426 /UG_GENE=4632432J16Rik /UG_TITLE=RIKEN cDNA 4632432J16 gene /DEF=Mus musculus 0 day neonate skin cDNA, RIKEN full-length enriched library, clone:4632432J16:HYPOTHETICAL HEART PROTEIN (FRAGMENT), full insert sequence. /FL=gb:NM_023341.1	281.7667	43.582718	593.36664	201.18909	2.6217752	2.0	0.11987785	0.3087763	2.11
gb:U25633.1 /DB_XREF=gi:1628496 /GEN=TMP /FEA=FLmRNA /CNT=216 /TID=Mm.30024.1 /TIER=FL+Stack /STK=83 /UG=Mm.30024 /LL=13730 /DEF=Mus musculus tumor-associated membrane protein (TMP) mRNA, complete cds. /PROD=tumor-associated membrane protein /FL=gb:U25633.1 gb:NM_010128.1	3414.7334	885.31415	5353.6997	1285.9819	2.1510766	3.0	0.120585434	0.30596304	1.57
gb:AV225808 /DB_XREF=gi:6177123 /DB_XREF=AV225808 /CLONE=3830432A03 /FEA=EST /CNT=20 /TID=Mm.219675.2 /TIER=Stack /STK=13 /UG=Mm.219675 /UG_TITLE=Mus musculus, Similar to elongation factor G2, clone MGC:28160 IMAGE:3984129, mRNA, complete cds	2209.2	1237.3901	4263.6665	585.0482	2.599816	2.0	0.1215556	0.30388898	1.93
gb:AK011543.1 /DB_XREF=gi:12847733 /FEA=mRNA /CNT=140 /TID=Mm.26893.1 /TIER=Stack /STK=104 /UG=Mm.26893 /LL=74197 /UG_GENE=Gtf2e1 /UG_TITLE=general transcription factor II E, polypeptide 1 (alpha subunit, 56kDa) /DEF=Mus musculus 10 days embryo whole body cDNA, RIKEN full-length enriched library, clone:2610024P03:homolog to GENERAL TRANSCRIPTION FACTOR IIE 56 KDA SUBUNIT, full insert sequence.	710.60004	8.685039	1085.6	256.12283	2.53451	2.0	0.12674415	0.3122682	1.53
gb:BC004048.1 /DB_XREF=gi:13278500 /FEA=FLmRNA /CNT=3 /TID=Mm.32831.2 /TIER=FL /STK=2 /UG=Mm.32831 /LL=24117 /UG_GENE=Wif1 /DEF=Mus musculus, clone MGC:7686 IMAGE:3497155, mRNA, complete cds. /PROD=Unknown (protein for MGC:7686) /FL=gb:BC004048.1	614.9	94.52243	944.5	206.26459	2.5161142	2.0	0.12826157	0.31149238	1.54
gb:AK014486.1 /DB_XREF=gi:12852374 /FEA=FLmRNA /CNT=216 /TID=Mm.147529.1 /TIER=Stack /STK=18 /UG=Mm.147529 /LL=67420 /UG_GENE=3732409C05Rik /UG_TITLE=RIKEN cDNA 3732409C05 gene /DEF=Mus musculus 14 days embryo liver cDNA, RIKEN full-length enriched library, clone:4432411H12:related to MALE STERILITY PROTEIN 2-LIKE PROTEIN, full insert sequence. /FL=gb:BC007178.1	404.23337	152.5175	607.5667	79.77861	2.0461202	3.0	0.13325994	0.31907308	1.50
gb:BC028256.1 /DB_XREF=gi:20380524 /FEA=mRNA /CNT=19 /TID=Mm.45357.2 /TIER=ConsEnd /STK=0 /UG=Mm.45357 /LL=59079 /UG_GENE=Erbb2ip /DEF=Mus musculus, Similar to erbb2 interacting protein, clone IMAGE:4988135, mRNA, partial cds. /PROD=Similar to erbb2 interacting protein	1193.3667	589.4717	2155.9001	562.5233	2.0460775	3.0	0.1332654	0.31465444	1.81
gb:BC025078.1 /DB_XREF=gi:19264014 /FEA=FLmRNA /CNT=61 /TID=Mm.28502.1 /TIER=FL+Stack /STK=17 /UG=Mm.28502 /LL=67966 /UG_GENE=2410141K03Rik /DEF=Mus musculus, RIKEN cDNA 2410141K03 gene, clone MGC:35654 IMAGE:4504126, mRNA, complete cds. /PROD=RIKEN cDNA 2410141K03 gene /FL=gb:BC025078.1 gb:NM_026479.1	192.53333	58.08876	323.46664	94.498375	2.044482	3.0	0.13346998	0.31082052	1.68
gb:NM_025307.1 /DB_XREF=gi:13384665 /GEN=Nrbf2 /FEA=FLmRNA /CNT=73 /TID=Mm.21209.1 /TIER=FL+Stack /STK=42 /UG=Mm.21209 /LL=50777 /DEF=Mus musculus nuclear receptor binding factor 2 (Nrbf2), mRNA. /PROD=nuclear receptor binding factor 2 /FL=gb:NM_025307.1 gb:BC019448.1	817.1667	287.53842	1247.9667	100.39329	2.4499803	2.0	0.13393125	0.3076799	1.53
gb:NM_008896.1 /DB_XREF=gi:6679416 /GEN=Pon2 /FEA=FLmRNA /CNT=70 /TID=Mm.22681.1 /TIER=FL+Stack /STK=27 /UG=Mm.22681 /LL=18980 /DEF=Mus musculus paraoxonase 2 (Pon2), mRNA. /PROD=paraoxonase 2 /FL=gb:L48514.1 gb:NM_008896.1	1238.8	514.217	1998.3667	390.5902	2.0373657	3.0	0.13438699	0.3046105	1.61
gb:NM_019458.1 /DB_XREF=gi:9506638 /GEN=5730511K23Rik /FEA=FLmRNA /CNT=103 /TID=Mm.7916.1 /TIER=FL+Stack /STK=66 /UG=Mm.7916 /LL=54624 /DEF=Mus musculus RIKEN cDNA 5730511K23 gene (5730511K23Rik), mRNA. /PROD=5730511K23Rik /FL=gb:NM_019458.1	319.76666	47.946877	484.93335	107.54671	2.4295177	2.0	0.13575621	0.3036652	1.52
gb:AF166486.1 /DB_XREF=gi:5733687 /GEN=Ku80 /FEA=FLmRNA /CNT=188 /TID=Mm.2627.1 /TIER=FL+Stack /STK=110 /UG=Mm.2627 /LL=22596 /DEF=Mus musculus Ku autoantigen (Ku80) mRNA, complete cds. /PROD=Ku autoantigen /FL=gb:AF166486.1	425.29996	169.33365	667.6667	44.300026	2.398363	2.0	0.13860177	0.3060039	1.57
gb:BC025450.1 /DB_XREF=gi:19343774 /FEA=mRNA /CNT=171 /TID=Mm.24463.1 /TIER=Stack /STK=124 /UG=Mm.24463 /LL=70049 /UG_GENE=2610044B22Rik /UG_TITLE=RIKEN cDNA 2610044B22 gene /DEF=Mus musculus, clone IMAGE:5345597, mRNA.	2023.8334	822.1557	3157.9336	534.31946	2.0033257	3.0	0.1388778	0.30268237	1.56
gb:NM_009364.2 /DB_XREF=gi:20070368 /GEN=Tfpi2 /FEA=FLmRNA /CNT=28 /TID=Mm.25612.1 /TIER=FL+Stack /STK=22 /UG=Mm.25612 /LL=21789 /DEF=Mus musculus tissue factor pathway inhibitor 2 (Tfpi2), mRNA. /PROD=tissue factor pathway inhibitor 2 /FL=gb:NM_009364.2 gb:D50586.1 gb:BC021639.1	220.2	47.49621	369.33334	98.90968	2.3541813	2.0	0.14278106	0.30725038	1.68
gb:BC015285.1 /DB_XREF=gi:15929723 /FEA=FLmRNA /CNT=61 /TID=Mm.35564.1 /TIER=FL+Stack /STK=23 /UG=Mm.35564 /DEF=Mus musculus, Similar to hypothetical protein, clone MGC:18890 IMAGE:4239390, mRNA, complete cds. /PROD=Similar to hypothetical protein /FL=gb:BC015285.1	736.60004	65.22938	1187.5333	328.61948	2.3312466	2.0	0.14501956	0.30816656	1.61
gb:BC020067.1 /DB_XREF=gi:18043640 /FEA=FLmRNA /CNT=50 /TID=Mm.35170.1 /TIER=FL+Stack /STK=24 /UG=Mm.35170 /LL=67770 /UG_GENE=5830433M19Rik /DEF=Mus musculus, Similar to hypothetical protein FLJ13657, clone MGC:28232 IMAGE:3991510, mRNA, complete cds. /PROD=Similar to hypothetical protein FLJ13657 /FL=gb:BC020067.1	269.6	84.23011	407.6	88.35162	1.9581054	3.0	0.14512028	0.30457342	1.51
gb:AK003758.1 /DB_XREF=gi:12834620 /FEA=mRNA /CNT=75 /TID=Mm.24411.1 /TIER=Stack /STK=47 /UG=Mm.24411 /LL=74780 /UG_GENE=1110017N23Rik /UG_TITLE=RIKEN cDNA 1110017N23 gene /DEF=Mus musculus 18 days embryo whole body cDNA, RIKEN full-length enriched library, clone:1110017N23:unclassifiable transcript, full insert sequence.	555.73334	164.02248	855.6667	211.4845	1.9410679	3.0	0.1475574	0.30591166	1.54
gb:BC027396.1 /DB_XREF=gi:20072075 /FEA=FLmRNA /CNT=242 /TID=Mm.29405.1 /TIER=FL+Stack /STK=44 /UG=Mm.29405 /LL=56438 /UG_GENE=Rbx1 /DEF=Mus musculus, ring-box 1, clone MGC:35907 IMAGE:4952242, mRNA, complete cds. /PROD=ring-box 1 /FL=gb:BC027396.1 gb:AF140599.1 gb:NM_019712.1	630.3	267.998	983.8	168.20787	1.9350696	3.0	0.14842682	0.30400673	1.56
gb:BG066552 /DB_XREF=gi:12549209 /DB_XREF=H3045C07-3 /CLONE=H3045C07 /FEA=mRNA /CNT=207 /TID=Mm.28483.1 /TIER=Stack /STK=75 /UG=Mm.28483 /UG_TITLE=Mus musculus, Similar to hypothetical protein FLJ22479, clone IMAGE:4487274, mRNA, partial cds	373.13333	65.45879	574.3667	139.45905	2.2624454	2.0	0.15203288	0.30768558	1.54
gb:NM_016912.1 /DB_XREF=gi:8393079 /GEN=Cdkl2 /FEA=FLmRNA /CNT=42 /TID=Mm.18133.1 /TIER=FL+Stack /STK=18 /UG=Mm.18133 /LL=53886 /DEF=Mus musculus cyclin-dependent kinase-like 2 (CDC2-related kinase) (Cdkl2), mRNA. /PROD=cyclin-dependent kinase-like 2 (CDC2-relatedkinase) /FL=gb:NM_016912.1 gb:AB029065.1	279.6	150.44058	468.13333	84.269585	1.893756	3.0	0.15458041	0.30916083	1.67
gb:BC006770.1 /DB_XREF=gi:13879576 /FEA=FLmRNA /CNT=1 /TID=Mm.220150.1 /TIER=FL /STK=1 /UG=Mm.220150 /DEF=Mus musculus, clone MGC:8099 IMAGE:3588650, mRNA, complete cds. /PROD=Unknown (protein for MGC:8099) /FL=gb:BC006770.1	346.1	44.936962	725.5334	290.35315	2.236813	2.0	0.15476531	0.30593142	2.10
gb:AK017225.1 /DB_XREF=gi:12856359 /FEA=mRNA /CNT=21 /TID=Mm.17858.1 /TIER=Stack /STK=14 /UG=Mm.17858 /LL=71407 /UG_GENE=5230400C17Rik /UG_TITLE=RIKEN cDNA 5230400C17 gene /DEF=Mus musculus adult male xiphoid cartilage cDNA, RIKEN full-length enriched library, clone:5230400C17:unclassifiable transcript, full insert sequence.	313.4	129.14647	485.4	93.75675	1.8667319	3.0	0.1587667	0.31023377	1.55
gb:BC026859.1 /DB_XREF=gi:20071539 /FEA=FLmRNA /CNT=59 /TID=Mm.24865.1 /TIER=FL+Stack /STK=44 /UG=Mm.24865 /DEF=Mus musculus, Similar to SEC15 (S. cerevisiae)-like, clone MGC:30428 IMAGE:2631201, mRNA, complete cds. /PROD=Similar to SEC15 (S. cerevisiae)-like /FL=gb:BC026859.1	568.13336	74.09219	868.0	226.2156	2.181918	2.0	0.16084833	0.3107297	1.53
gb:NM_133739.1 /DB_XREF=gi:19526937 /GEN=2310075C12Rik /FEA=FLmRNA /CNT=150 /TID=Mm.146516.1 /TIER=FL+Stack /STK=52 /UG=Mm.146516 /LL=71929 /DEF=Mus musculus RIKEN cDNA 2310075C12 gene (2310075C12Rik), mRNA. /PROD=RIKEN cDNA 2310075C12 /FL=gb:BC010292.1 gb:NM_133739.1	797.3333	489.41528	1390.7999	263.78073	1.8488518	3.0	0.16160896	0.3086913	1.74
gb:BC017537.1 /DB_XREF=gi:17028436 /FEA=FLmRNA /CNT=71 /TID=Mm.25333.1 /TIER=FL+Stack /STK=50 /UG=Mm.25333 /LL=75669 /UG_GENE=Pik3r4 /DEF=Mus musculus, clone MGC:28823 IMAGE:4505099, mRNA, complete cds. /PROD=Unknown (protein for MGC:28823) /FL=gb:BC017537.1	245.83331	89.719574	436.30002	157.05818	1.8238696	3.0	0.16567954	0.31295022	1.77
gb:BC027196.1 /DB_XREF=gi:20071133 /FEA=FLmRNA /CNT=203 /TID=Mm.34411.1 /TIER=FL+Stack /STK=128 /UG=Mm.34411 /DEF=Mus musculus, Similar to pyruvate dehydrogenase kinase, isoenzyme 1, clone MGC:28719 IMAGE:4458562, mRNA, complete cds. /PROD=Similar to pyruvate dehydrogenase kinase,isoenzyme 1 /FL=gb:BC027196.1	813.5	142.77159	1572.3334	606.1789	2.110487	2.0	0.16926411	0.31620768	1.93
gb:AK009885.1 /DB_XREF=gi:12844954 /FEA=mRNA /CNT=47 /TID=Mm.31827.1 /TIER=Stack /STK=30 /UG=Mm.31827 /LL=76466 /UG_GENE=2310047L21Rik /UG_TITLE=RIKEN cDNA 2310047L21 gene /DEF=Mus musculus adult male tongue cDNA, RIKEN full-length enriched library, clone:2310047L21:homolog to WUGSC:H_NH0438H20.1 PROTEIN (FRAGMENT), full insert sequence.	498.56665	42.196716	826.2	265.82748	2.1083615	2.0	0.16952364	0.31325018	1.66
gb:AK009725.1 /DB_XREF=gi:12844697 /FEA=mRNA /CNT=105 /TID=Mm.32254.1 /TIER=Stack /STK=84 /UG=Mm.32254 /LL=76451 /UG_GENE=2310040I01Rik /UG_TITLE=RIKEN cDNA 2310040I01 gene /DEF=Mus musculus adult male tongue cDNA, RIKEN full-length enriched library, clone:2310040I01:unclassifiable transcript, full insert sequence.	237.43333	92.97453	376.46667	96.53032	1.796792	3.0	0.17022601	0.3111658	1.59
gb:AK010060.1 /DB_XREF=gi:12845241 /GEN=Araf /FEA=mRNA /CNT=199 /TID=Mm.220946.1 /TIER=Stack /STK=29 /UG=Mm.220946 /UG_TITLE=Mus musculus adult male lung cDNA, RIKEN full-length enriched library, clone:1200013E08:raf-related oncogene, full insert sequence /DEF=Mus musculus adult male tongue cDNA, RIKEN full-length enriched library, clone:2310066D16:raf-related oncogene, full insert sequence.	300.30002	228.72858	612.93335	196.4921	1.7957757	3.0	0.17039944	0.3081692	2.04
gb:BC012262.1 /DB_XREF=gi:15126666 /FEA=FLmRNA /CNT=63 /TID=Mm.34514.1 /TIER=FL+Stack /STK=43 /UG=Mm.34514 /LL=71704 /UG_GENE=Arhgef3 /DEF=Mus musculus, clone MGC:19066 IMAGE:4192378, mRNA, complete cds. /PROD=Unknown (protein for MGC:19066) /FL=gb:BC012262.1 gb:BC005517.1	847.4666	87.602066	1277.0667	347.1345	2.07836	2.0	0.17324533	0.31001794	1.51
gb:BB504983 /DB_XREF=gi:9493777 /DB_XREF=BB504983 /CLONE=D630050N19 /FEA=EST /CNT=35 /TID=Mm.30245.4 /TIER=Stack /STK=34 /UG=Mm.30245 /UG_TITLE=Mus musculus, Similar to phosphatidylserine decarboxylase, clone MGC:7133 IMAGE:3158145, mRNA, complete cds	1228.9	481.8121	1871.4332	403.02145	1.7717181	3.0	0.17456463	0.30912486	1.52
gb:BC016105.1 /DB_XREF=gi:16359288 /FEA=FLmRNA /CNT=89 /TID=Mm.6724.1 /TIER=FL+Stack /STK=56 /UG=Mm.6724 /DEF=Mus musculus, clone MGC:27690 IMAGE:4921222, mRNA, complete cds. /PROD=Unknown (protein for MGC:27690) /FL=gb:BC016105.1	263.69998	24.313162	398.56665	111.872986	2.0404158	2.0	0.17811294	0.3121567	1.51
gb:BB433489 /DB_XREF=gi:9273216 /DB_XREF=BB433489 /CLONE=C820001M03 /FEA=EST /CNT=10 /TID=Mm.217102.3 /TIER=Stack /STK=8 /UG=Mm.217102 /UG_TITLE=Mus musculus, clone MGC:25836 IMAGE:4190175, mRNA, complete cds	1077.0333	378.58313	1656.7334	431.17288	1.7498903	3.0	0.17844518	0.30954775	1.54
gb:BM225280 /DB_XREF=gi:17786807 /DB_XREF=K0209C02-3 /CLONE=K0209C02 /FEA=FLmRNA /CNT=29 /TID=Mm.65655.1 /TIER=Stack /STK=13 /UG=Mm.65655 /UG_TITLE=Mus musculus, clone MGC:35978 IMAGE:5353641, mRNA, complete cds /FL=gb:BC021527.1	651.3333	209.51527	1085.6	375.84653	1.7480214	3.0	0.178782	0.3069994	1.67
gb:BB311034 /DB_XREF=gi:16402667 /DB_XREF=BB311034 /CLONE=B230321B18 /FEA=FLmRNA /CNT=290 /TID=Mm.194045.1 /TIER=Stack /STK=161 /UG=Mm.194045 /UG_TITLE=Mus musculus, clone MGC:36474 IMAGE:5361238, mRNA, complete cds /FL=gb:BC025804.1 gb:BC027056.1	2448.6667	768.1256	3768.6335	1084.3678	1.7204585	3.0	0.18383482	0.3125192	1.54
gb:NM_026341.1 /DB_XREF=gi:13385843 /GEN=4933433B15Rik /FEA=FLmRNA /CNT=6 /TID=Mm.41966.1 /TIER=FL /STK=1 /UG=Mm.41966 /LL=67725 /DEF=Mus musculus RIKEN cDNA 4933433B15 gene (4933433B15Rik), mRNA. /PROD=RIKEN cDNA 4933433B15 /FL=gb:NM_026341.1	754.6667	307.8523	1166.3334	280.70532	1.7114762	3.0	0.1855165	0.3122555	1.55
gb:BC026627.1 /DB_XREF=gi:20071839 /FEA=mRNA /CNT=37 /TID=Mm.33546.1 /TIER=Stack /STK=21 /UG=Mm.33546 /DEF=Mus musculus, Similar to DKFZP586L2024 protein, clone IMAGE:4982180, mRNA, partial cds. /PROD=Similar to DKFZP586L2024 protein	375.06665	175.38116	597.9666	84.79719	1.9818455	2.0	0.18599652	0.3099942	1.59
gb:NM_015751.1 /DB_XREF=gi:7657517 /GEN=Abce1 /FEA=FLmRNA /CNT=245 /TID=Mm.5831.1 /TIER=FL+Stack /STK=145 /UG=Mm.5831 /LL=24015 /DEF=Mus musculus ATP-binding cassette, sub-family E (OABP), member 1 (Abce1), mRNA. /PROD=ATP-binding cassette, subfamily E, member 1 /FL=gb:NM_015751.1 gb:U90446.1 gb:BC005422.1	1349.0668	637.7267	2110.8	209.44952	1.9655546	2.0	0.18827267	0.3107413	1.56
gb:AK011776.1 /DB_XREF=gi:12848114 /FEA=mRNA /CNT=40 /TID=Mm.30923.1 /TIER=Stack /STK=25 /UG=Mm.30923 /LL=72139 /UG_GENE=2610044O15Rik /UG_TITLE=RIKEN cDNA 2610044O15 gene /DEF=Mus musculus 10 days embryo whole body cDNA, RIKEN full-length enriched library, clone:2610044O15:similar to KRUPPEL-RELATED ZINC FINGER PROTEIN F80-S, full insert sequence.	321.33334	145.92743	492.1	99.984215	1.6720506	3.0	0.19310763	0.3156567	1.53
gb:NM_009840.1 /DB_XREF=gi:6753327 /GEN=Cct8 /FEA=FLmRNA /CNT=482 /TID=Mm.17989.1 /TIER=FL+Stack /STK=281 /UG=Mm.17989 /LL=12469 /DEF=Mus musculus chaperonin subunit 8 (theta) (Cct8), mRNA. /PROD=chaperonin subunit 8 (theta) /FL=gb:NM_009840.1 gb:BC009007.1	1954.2334	813.74225	2928.2002	348.0841	1.9060308	2.0	0.1969144	0.3188138	1.50
gb:BM117243 /DB_XREF=gi:17080261 /DB_XREF=L0848C02-3 /CLONE=L0848C02 /FEA=mRNA /CNT=252 /TID=Mm.29308.1 /TIER=ConsEnd /STK=0 /UG=Mm.29308 /UG_TITLE=Mus musculus, Similar to chromosome 20 open reading frame 36, clone IMAGE:5356821, mRNA, partial cds	1115.3667	606.996	1858.0	489.62155	1.6493825	3.0	0.1976312	0.3169557	1.67
gb:NM_023423.1 /DB_XREF=gi:12963672 /GEN=6330407G11Rik /FEA=FLmRNA /CNT=142 /TID=Mm.27903.1 /TIER=FL+Stack /STK=64 /UG=Mm.27903 /LL=68050 /DEF=Mus musculus RIKEN cDNA 6330407G11 gene (6330407G11Rik), mRNA. /PROD=RIKEN cDNA 6330407G11 /FL=gb:BC003291.1 gb:NM_023423.1	767.13336	60.56274	1230.6333	419.40765	1.8944916	2.0	0.1986508	0.31561345	1.60
gb:BC006953.1 /DB_XREF=gi:13905310 /FEA=FLmRNA /CNT=147 /TID=Mm.43671.1 /TIER=FL+Stack /STK=73 /UG=Mm.43671 /LL=66646 /UG_GENE=5730518J08Rik /DEF=Mus musculus, clone MGC:6936 IMAGE:2811899, mRNA, complete cds. /PROD=Unknown (protein for MGC:6936) /FL=gb:NM_025683.1 gb:BC006953.1 gb:BC019126.1	332.86667	48.727432	529.0333	175.3655	1.8667752	2.0	0.20290579	0.31938872	1.59
gb:BC018507.1 /DB_XREF=gi:17391194 /FEA=FLmRNA /CNT=100 /TID=Mm.139738.1 /TIER=FL+Stack /STK=57 /UG=Mm.139738 /DEF=Mus musculus, clone MGC:27742 IMAGE:2649340, mRNA, complete cds. /PROD=Unknown (protein for MGC:27742) /FL=gb:BC018507.1	372.8667	197.8616	602.2	80.845604	1.858403	2.0	0.20421492	0.31850034	1.62
gb:AK017277.1 /DB_XREF=gi:12856436 /FEA=mRNA /CNT=21 /TID=Mm.103495.1 /TIER=Stack /STK=10 /UG=Mm.103495 /LL=71324 /UG_GENE=5430405N12Rik /UG_TITLE=RIKEN cDNA 5430405N12 gene /DEF=Mus musculus 6 days neonate head cDNA, RIKEN full-length enriched library, clone:5430405N12:hypothetical protein, full insert sequence.	1315.7	752.4374	2206.2	358.13565	1.8508978	2.0	0.20539802	0.3174333	1.68
gb:NM_011668.1 /DB_XREF=gi:7106440 /GEN=Ube3a /FEA=FLmRNA /CNT=174 /TID=Mm.9002.1 /TIER=FL+Stack /STK=74 /UG=Mm.9002 /LL=22215 /DEF=Mus musculus ubiquitin protein ligase E3A (Ube3a), mRNA. /PROD=ubiquitin protein ligase E3A /FL=gb:U82122.1 gb:U96636.1 gb:NM_011668.1	2141.9333	729.3072	3311.8	1026.2908	1.6093861	3.0	0.20590682	0.3153528	1.55
gb:AK002371.1 /DB_XREF=gi:12832303 /FEA=mRNA /CNT=120 /TID=Mm.29606.1 /TIER=Stack /STK=64 /UG=Mm.29606 /LL=66585 /UG_GENE=0610009C03Rik /UG_TITLE=RIKEN cDNA 0610009C03 gene /DEF=Mus musculus adult male kidney cDNA, RIKEN full-length enriched library, clone:0610009C03:homolog to U5 SNRNP-SPECIFIC 40 KDA PROTEIN, full insert sequence.	414.70004	150.9388	621.5334	163.65213	1.6091467	3.0	0.2059575	0.31261405	1.50
gb:BC019131.1 /DB_XREF=gi:17512319 /FEA=FLmRNA /CNT=89 /TID=Mm.76694.1 /TIER=FL+Stack /STK=38 /UG=Mm.76694 /LL=68864 /UG_GENE=1110067D22Rik /DEF=Mus musculus, clone MGC:29165 IMAGE:5036749, mRNA, complete cds. /PROD=Unknown (protein for MGC:29165) /FL=gb:BC019131.1	1780.6666	942.588	2963.0667	868.2394	1.5980754	3.0	0.20831712	0.31339744	1.66
gb:AJ002387.1 /DB_XREF=gi:2598561 /FEA=mRNA /CNT=14 /TID=Mm.918.2 /TIER=ConsEnd /STK=0 /UG=Mm.918 /LL=14828 /UG_GENE=Hspa5 /DEF=Mus musculus mRNA for BiP. /PROD=BiP	3538.3337	1849.5023	5539.8667	464.41464	1.8179885	2.0	0.2106946	0.3141937	1.57
gb:BM235840 /DB_XREF=gi:17871110 /DB_XREF=K0426C06-3 /CLONE=K0426C06 /FEA=mRNA /CNT=47 /TID=Mm.159813.1 /TIER=Stack /STK=11 /UG=Mm.159813 /UG_TITLE=Mus musculus, clone IMAGE:5102112, mRNA, partial cds	1349.6	955.2317	2434.4333	712.7231	1.5765651	3.0	0.21298838	0.3148524	1.80
gb:AK017822.1 /DB_XREF=gi:12857270 /GEN=Ssb /FEA=mRNA /CNT=2 /TID=Mm.10508.2 /TIER=ConsEnd /STK=1 /UG=Mm.10508 /LL=20823 /UG_TITLE=Sjogren syndrome antigen B /DEF=Mus musculus 8 days embryo whole body cDNA, RIKEN full-length enriched library, clone:5730546E07:Sjogren syndrome antigen B, full insert sequence.	938.3333	519.42316	1485.9	91.67742	1.798105	2.0	0.21398263	0.3135952	1.58
gb:NM_007683.1 /DB_XREF=gi:6671739 /GEN=Cenpc /FEA=FLmRNA /CNT=12 /TID=Mm.4649.1 /TIER=FL /STK=5 /UG=Mm.4649 /LL=12617 /DEF=Mus musculus centromere autoantigen C (Cenpc), mRNA. /PROD=centromere autoantigen C /FL=gb:U03113.1 gb:NM_007683.1	380.56665	61.065964	572.93335	176.53152	1.7837127	2.0	0.21640497	0.3144346	1.51
gb:AK018685.1 /DB_XREF=gi:12858516 /FEA=FLmRNA /CNT=55 /TID=Mm.1622.1 /TIER=Stack /STK=9 /UG=Mm.1622 /LL=66819 /UG_GENE=9130422G05Rik /UG_TITLE=RIKEN cDNA 9130422G05 gene /DEF=Mus musculus adult male cecum cDNA, RIKEN full-length enriched library, clone:9130422G05:unclassifiable, full insert sequence. /FL=gb:BC020085.1	339.66666	163.15001	516.0	108.10833	1.560506	3.0	0.21655197	0.31198165	1.52
gb:AK008084.1 /DB_XREF=gi:12842047 /FEA=mRNA /CNT=305 /TID=Mm.28189.1 /TIER=Stack /STK=29 /UG=Mm.28189 /LL=57912 /UG_GENE=Spec1-pending /UG_TITLE=small protein effector 1 of Cdc42 /DEF=Mus musculus adult male small intestine cDNA, RIKEN full-length enriched library, clone:2010004C01:small protein effector 1 of Cdc42, full insert sequence.	203.63335	73.43094	317.33334	103.37283	1.553118	3.0	0.2182136	0.31173372	1.56
gb:BC026821.1 /DB_XREF=gi:20070721 /FEA=FLmRNA /CNT=85 /TID=Mm.87443.1 /TIER=FL+Stack /STK=32 /UG=Mm.87443 /LL=16649 /UG_GENE=Kpna4 /DEF=Mus musculus, karyopherin (importin) alpha 4, clone MGC:19244 IMAGE:3672708, mRNA, complete cds. /PROD=karyopherin (importin) alpha 4 /FL=gb:AF020771.1 gb:NM_008467.1 gb:BC026821.1	4096.9004	875.8401	6336.9	2006.1893	1.7723727	2.0	0.21833913	0.30931377	1.55
gb:NM_133748.1 /DB_XREF=gi:19526953 /GEN=2900053I11Rik /FEA=FLmRNA /CNT=102 /TID=Mm.27136.1 /TIER=FL+Stack /STK=32 /UG=Mm.27136 /LL=72999 /DEF=Mus musculus RIKEN cDNA 2900053I11 gene (2900053I11Rik), mRNA. /PROD=RIKEN cDNA 2900053I11 /FL=gb:BC024411.1 gb:BC015288.1 gb:NM_133748.1 gb:BC023067.1	1265.4667	550.9264	1911.3334	479.13797	1.5321524	3.0	0.2230064	0.31331477	1.51
gb:AK018466.1 /DB_XREF=gi:12858179 /FEA=mRNA /CNT=65 /TID=Mm.99916.1 /TIER=Stack /STK=48 /UG=Mm.99916 /LL=71506 /UG_GENE=8430436O14Rik /UG_TITLE=RIKEN cDNA 8430436O14 gene /DEF=Mus musculus 16 days embryo lung cDNA, RIKEN full-length enriched library, clone:8430436O14:unclassifiable transcript, full insert sequence.	838.56665	393.75806	1282.5333	198.18613	1.7444108	2.0	0.22320645	0.31102538	1.53

gb:NM_020010.1 /DB_XREF=gi:9910171 /GEN=Cyp51 /FEA=FLmRNA /CNT=202 /TID=Mm.140158.1 /TIER=FL+Stack /STK=42 /UG=Mm.140158 /LL=13121 /DEF=Mus musculus cytochrome P450, 51 (Cyp51), mRNA. /PROD=cytochrome P450, 51 /FL=gb:NM_020010.1 gb:AF166266.1	249.43335	21.021502	382.69998	132.06241	1.7261136	2.0	0.2264687	0.31300554	1.53
gb:NM_008748.1 /DB_XREF=gi:6679155 /GEN=Nttp1 /FEA=FLmRNA /CNT=37 /TID=Mm.39725.1 /TIER=FL+Stack /STK=19 /UG=Mm.39725 /LL=18218 /DEF=Mus musculus neuronal tyrosinethreonine phosphatase 1 (Nttp1), mRNA. /PROD=neuronal tyrosinethreonine phosphatase 1 /FL=gb:NM_008748.1	348.2333	198.05476	573.56665	165.27673	1.5129963	3.0	0.22748737	0.31187785	1.65
gb:AK016542.1 /DB_XREF=gi:12855327 /FEA=mRNA /CNT=4 /TID=Mm.27207.2 /TIER=ConsEnd /STK=1 /UG=Mm.27207 /LL=74386 /UG_GENE=4932432N11Rik /UG_TITLE=RIKEN cDNA 4932432N11 gene /DEF=Mus musculus adult male testis cDNA, RIKEN full-length enriched library, clone:4932432N11:homolog to CDNA FLJ12888 FIS, CLONE NT2RP2004081, full insert sequence.	429.73337	246.08824	673.56665	83.53874	1.6250961	2.0	0.24564427	0.33407623	1.57
gb:NM_019812.1 /DB_XREF=gi:9790228 /GEN=Sirt1 /FEA=FLmRNA /CNT=97 /TID=Mm.12604.1 /TIER=FL+Stack /STK=20 /UG=Mm.12604 /LL=93759 /DEF=Mus musculus sirtuin 1 ((silent mating type information regulation 2, homolog) 1 (S. cerevisiae) (Sirt1), mRNA. /PROD=sirtuin 1 ((silent mating type informationregulation 2, homolog) 1 (S. cerevisiae) /FL=gb:NM_019812.1 gb:AF214646.1	559.0	300.70676	850.0334	76.42424	1.6246833	2.0	0.24572687	0.33153623	1.52
gb:BC006828.1 /DB_XREF=gi:13905085 /FEA=FLmRNA /CNT=63 /TID=Mm.30501.1 /TIER=FL+Stack /STK=18 /UG=Mm.30501 /DEF=Mus musculus, Similar to microfibrillar-associated protein 3, clone MGC:11870 IMAGE:3597878, mRNA, complete cds. /PROD=Similar to microfibrillar-associated protein 3 /FL=gb:BC006828.1	539.26666	346.3587	886.5	140.9129	1.6084077	2.0	0.24901208	0.33332327	1.64
gb:BC027196.1 /DB_XREF=gi:20071133 /FEA=FLmRNA /CNT=203 /TID=Mm.34411.1 /TIER=FL+Stack /STK=128 /UG=Mm.34411 /DEF=Mus musculus, Similar to pyruvate dehydrogenase kinase, isoenzyme 1, clone MGC:28719 IMAGE:4458562, mRNA, complete cds. /PROD=Similar to pyruvate dehydrogenase kinase,isoenzyme 1 /FL=gb:BC027196.1	1481.2001	424.4901	2639.0334	1182.8103	1.5958194	2.0	0.25159192	0.33414552	1.78
gb:NM_020043.1 /DB_XREF=gi:9910463 /GEN=Nope /FEA=FLmRNA /CNT=107 /TID=Mm.143741.1 /TIER=FL+Stack /STK=87 /UG=Mm.143741 /LL=56741 /DEF=Mus musculus neighbor of Punc E11 (Nope), mRNA. /PROD=neighbor of Punc e11 protein /FL=gb:AF176694.1 gb:AB052621.1 gb:AB052620.1 gb:NM_020043.1	650.63336	374.27472	1000.5667	210.0922	1.4121382	3.0	0.25276536	0.33310163	1.54
gb:BC013496.1 /DB_XREF=gi:15488700 /FEA=mRNA /CNT=2 /TID=Mm.104747.19 /TIER=ConsEnd /STK=0 /UG=Mm.104747 /LL=16114 /UG_GENE=Igk-V28 /DEF=Mus musculus, clone IMAGE:4219618, mRNA, partial cds. /PROD=Unknown (protein for IMAGE:4219618)	2536.7	1346.0999	5748.767	3229.9329	1.5899211	2.0	0.2528125	0.33060095	2.27
gb:BC013777.1 /DB_XREF=gi:15489363 /FEA=FLmRNA /CNT=96 /TID=Mm.821.1 /TIER=FL+Stack /STK=47 /UG=Mm.821 /LL=57806 /UG_GENE=Snrpa1 /DEF=Mus musculus, clone MGC:6298 IMAGE:2651704, mRNA, complete cds. /PROD=Unknown (protein for MGC:6298) /FL=gb:AF230356.1 gb:BC013777.1 gb:NM_021336.1	1188.9333	536.97046	1902.2999	693.9675	1.4081479	3.0	0.2538262	0.3293928	1.60
gb:BC004096.1 /DB_XREF=gi:13278617 /FEA=FLmRNA /CNT=1 /TID=Mm.16710.2 /TIER=FL /STK=1 /UG=Mm.16710 /LL=16211 /UG_GENE=Impnb /DEF=Mus musculus, clone MGC:8315 IMAGE:3594201, mRNA, complete cds. /PROD=Unknown (protein for MGC:8315) /FL=gb:BC004096.1	2197.7332	580.041	3536.7668	1344.1012	1.5842928	2.0	0.2539843	0.327101	1.61
gb:NM_025706.1 /DB_XREF=gi:13385163 /GEN=4432405K22Rik /FEA=FLmRNA /CNT=232 /TID=Mm.22252.1 /TIER=FL+Stack /STK=137 /UG=Mm.22252 /LL=66687 /DEF=Mus musculus RIKEN cDNA 4432405K22 gene (4432405K22Rik), mRNA. /PROD=RIKEN cDNA 4432405K22 /FL=gb:NM_025706.1	2074.0334	925.5119	3127.4668	926.4323	1.3933324	3.0	0.25780717	0.32952797	1.51
gb:NM_007639.1 /DB_XREF=gi:6671705 /GEN=Cd1d1 /FEA=FLmRNA /CNT=52 /TID=Mm.1894.1 /TIER=FL+Stack /STK=22 /UG=Mm.1894 /LL=12479 /DEF=Mus musculus CD1d1 antigen (Cd1d1), mRNA. /PROD=CD1d1 antigen /FL=gb:NM_007639.1 gb:M63695.1	295.33334	122.077934	463.53336	170.89987	1.3871365	3.0	0.25949186	0.32920608	1.57
gb:AK004661.1 /DB_XREF=gi:12835991 /FEA=FLmRNA /CNT=211 /TID=Mm.22179.1 /TIER=Stack /STK=28 /UG=Mm.22179 /LL=67456 /UG_GENE=1200009B18Rik /UG_TITLE=RIKEN cDNA 1200009B18 gene /DEF=Mus musculus adult male lung cDNA, RIKEN full-length enriched library, clone:1200009B18:homolog to CDA14, full insert sequence. /FL=gb:BC006895.1 gb:BC018188.1 gb:BC026558.1	963.0	467.03268	1549.3334	564.1123	1.3867055	3.0	0.25960946	0.32691562	1.61
gb:BC023060.1 /DB_XREF=gi:18606201 /FEA=mRNA /CNT=19 /TID=Mm.44176.1 /TIER=Stack /STK=11 /UG=Mm.44176 /DEF=Mus musculus, Similar to EGF-containing fibulin-like extracellular matrix protein 1, clone IMAGE:5357328, mRNA, partial cds. /PROD=Similar to EGF-containing fibulin-likeextracellular matrix protein 1	1247.9333	767.05914	1964.3334	217.40591	1.5563558	2.0	0.25990483	0.32488105	1.57
gb:NM_138756.1 /DB_XREF=gi:20270292 /GEN=BC008171 /FEA=FLmRNA /CNT=117 /TID=Mm.135837.1 /TIER=FL /STK=7 /UG=Mm.135837 /LL=192287 /DEF=Mus musculus hypothetical protein BC008171 (BC008171), mRNA. /PROD=hypothetical protein BC008171 /FL=gb:NM_138756.1 gb:BC008171.1	509.66666	159.7224	845.7	344.3217	1.5334098	2.0	0.26489997	0.328708	1.66
gb:BB787946 /DB_XREF=gi:16956442 /DB_XREF=BB787946 /CLONE=G430120E06 /FEA=mRNA /CNT=81 /TID=Mm.93759.1 /TIER=Stack /STK=52 /UG=Mm.93759 /UG_TITLE=Mus musculus, clone IMAGE:3489582, mRNA, partial cds	190.93333	87.15917	290.1	93.40991	1.3444291	3.0	0.27142897	0.33436903	1.52
gb:BB557441 /DB_XREF=gi:9643807 /DB_XREF=BB557441 /CLONE=E330028F09 /FEA=EST /CNT=18 /TID=Mm.58104.2 /TIER=Stack /STK=18 /UG=Mm.58104 /UG_TITLE=Mus musculus, clone MGC:27940 IMAGE:3587176, mRNA, complete cds	417.86667	174.88164	709.2	334.79416	1.3359292	3.0	0.27387375	0.33495352	1.70
gb:AK011939.1 /DB_XREF=gi:12848371 /FEA=mRNA /CNT=47 /TID=Mm.27121.1 /TIER=Stack /STK=22 /UG=Mm.27121 /LL=70451 /UG_GENE=2610209N15Rik /UG_TITLE=RIKEN cDNA 2610209N15 gene /DEF=Mus musculus 10 days embryo whole body cDNA, RIKEN full-length enriched library, clone:2610209N15:homolog to ANDROGEN-REGULATED SHORT-CHAIN DEHYDROGENASEREDUCTASE 1, full insert sequence.	173.5	96.886734	260.03336	27.103933	1.4897667	2.0	0.27474177	0.333615	1.50
gb:NM_009140.1 /DB_XREF=gi:6677884 /GEN=Scyb2 /FEA=FLmRNA /CNT=4 /TID=Mm.4979.1 /TIER=FL /STK=3 /UG=Mm.4979 /LL=20310 /DEF=Mus musculus small inducible cytokine subfamily, member 2 (Scyb2), mRNA. /PROD=small inducible cytokine subfamily, member 2 /FL=gb:NM_009140.1	214.16667	104.578705	363.33334	165.20993	1.32137	3.0	0.27811554	0.33531663	1.70
gb:NM_030612.1 /DB_XREF=gi:13447397 /GEN=Mail-pending /FEA=FLmRNA /CNT=111 /TID=Mm.3732.1 /TIER=FL+Stack /STK=44 /UG=Mm.3732 /LL=80859 /DEF=Mus musculus molecule possessing ankyrin-repeats induced by lipopolysaccharide (Mail-pending), mRNA. /PROD=molecule possessing ankyrin-repeats induced bylipopolysaccharide /FL=gb:NM_030612.1 gb:AB026551.2 gb:AB020974.1	536.0333	98.96349	869.8333	379.71127	1.4734068	2.0	0.27854982	0.33347514	1.62
gb:BC020068.1 /DB_XREF=gi:18043363 /FEA=FLmRNA /CNT=31 /TID=Mm.172352.1 /TIER=FL /STK=6 /UG=Mm.172352 /DEF=Mus musculus, Similar to RIKEN cDNA 9530053H05 gene, clone MGC:28371 IMAGE:4020866, mRNA, complete cds. /PROD=Similar to RIKEN cDNA 9530053H05 gene /FL=gb:BC020068.1	610.4333	222.68494	1039.9	463.5047	1.4465678	2.0	0.28494188	0.3387421	1.70
gb:BC012405.1 /DB_XREF=gi:15214561 /FEA=FLmRNA /CNT=22 /TID=Mm.45217.1 /TIER=FL+Stack /STK=13 /UG=Mm.45217 /DEF=Mus musculus, clone MGC:12134 IMAGE:3710576, mRNA, complete cds. /PROD=Unknown (protein for MGC:12134) /FL=gb:BC012405.1	386.70004	56.796032	588.3	236.07414	1.4380835	2.0	0.28700063	0.3388202	1.52
gb:AK017269.1 /DB_XREF=gi:12856421 /FEA=mRNA /CNT=42 /TID=Mm.23242.1 /TIER=Stack /STK=21 /UG=Mm.23242 /LL=71408 /UG_GENE=5430405D20Rik /UG_TITLE=RIKEN cDNA 5430405D20 gene /DEF=Mus musculus 6 days neonate head cDNA, RIKEN full-length enriched library, clone:5430405D20:unclassifiable transcript, full insert sequence.	851.36664	218.27292	1371.7666	598.14606	1.4156123	2.0	0.29254377	0.34298235	1.61
gb:NM_010050.1 /DB_XREF=gi:6753637 /GEN=Dio2 /FEA=FLmRNA /CNT=27 /TID=Mm.21389.1 /TIER=FL+Stack /STK=16 /UG=Mm.21389 /LL=13371 /DEF=Mus musculus deiodinase, iodothyronine, type II (Dio2), mRNA. /PROD=deiodinase, iodothyronine, type II /FL=gb:AF177196.1 gb:NM_010050.1 gb:AF093137.1 gb:AF096875.1	400.40002	116.28194	767.9666	442.2855	1.3921314	2.0	0.2984789	0.34754393	1.92
gb:BC021443.1 /DB_XREF=gi:18204053 /FEA=FLmRNA /CNT=4 /TID=Mm.196130.1 /TIER=FL /STK=3 /UG=Mm.196130 /LL=68788 /UG_GENE=1110050F08Rik /DEF=Mus musculus, Similar to RIKEN cDNA 6530403A03 gene, clone MGC:29356 IMAGE:5038605, mRNA, complete cds. /PROD=Similar to RIKEN cDNA 6530403A03 gene /FL=gb:BC021443.1	509.43335	135.68286	814.93335	355.3257	1.3911959	2.0	0.29871845	0.34545672	1.60
gb:BC026055.1 /DB_XREF=gi:19913455 /FEA=FLmRNA /CNT=113 /TID=Mm.3379.2 /TIER=FL /STK=4 /UG=Mm.3379 /LL=20425 /UG_GENE=Shmt1 /DEF=Mus musculus, serine hydroxymethyl transferase 1 (soluble), clone MGC:14007 IMAGE:4160579, mRNA, complete cds. /PROD=serine hydroxymethyl transferase 1 (soluble) /FL=gb:AF237702.1 gb:BC026055.1	459.9	176.8502	1045.8334	731.91003	1.3478124	2.0	0.31009194	0.3561867	2.27
gb:NM_134130.1 /DB_XREF=gi:19527359 /GEN=Labh3-pending /FEA=FLmRNA /CNT=43 /TID=Mm.7100.1 /TIER=FL+Stack /STK=33 /UG=Mm.7100 /LL=106861 /DEF=Mus musculus lung alphabeta hydrolase 3 (Labh3-pending), mRNA. /PROD=lung alphabeta hydrolase 3 /FL=gb:AF429302.1 gb:NM_134130.1 gb:BC026770.1	176.59999	29.163845	311.63333	175.77275	1.3126625	2.0	0.31969804	0.36475617	1.76
gb:NM_025959.1 /DB_XREF=gi:13385449 /GEN=2300001E01Rik /FEA=FLmRNA /CNT=160 /TID=Mm.18472.1 /TIER=FL+Stack /STK=36 /UG=Mm.18472 /LL=67089 /DEF=Mus musculus RIKEN cDNA 2300001E01 gene (2300001E01Rik), mRNA. /PROD=RIKEN cDNA 2300001E01 /FL=gb:NM_025959.1	2173.4001	478.3655	3294.8665	1436.6143	1.2828438	2.0	0.32813188	0.3718828	1.52
gb:AA798895 /DB_XREF=gi:2861850 /DB_XREF=vw94e07.r1 /CLONE=IMAGE:1230084 /FEA=EST /CNT=14 /TID=Mm.220953.2 /TIER=Stack /STK=9 /UG=Mm.220953 /UG_TITLE=Mus musculus, clone IMAGE:4206769, mRNA	840.73334	59.36046	1457.7001	844.5047	1.2622637	2.0	0.33410957	0.37614986	1.73
gb:NM_008540.1 /DB_XREF=gi:6678771 /GEN=Madh4 /FEA=FLmRNA /CNT=200 /TID=Mm.100399.1 /TIER=FL+Stack /STK=73 /UG=Mm.100399 /LL=17128 /DEF=Mus musculus MAD homolog 4 (Drosophila) (Madh4), mRNA. /PROD=MAD homolog 4 (Drosophila) /FL=gb:U79748.1 gb:NM_008540.1	1135.6666	320.7318	1741.6333	773.355	1.2536227	2.0	0.33665836	0.3765258	1.53
gb:BC006026.1 /DB_XREF=gi:13543747 /FEA=FLmRNA /CNT=129 /TID=Mm.1192.1 /TIER=FL+Stack /STK=38 /UG=Mm.1192 /LL=16069 /UG_GENE=Igj /DEF=Mus musculus, Similar to immunoglobulin joining chain, clone MGC:6626 IMAGE:3491646, mRNA, complete cds. /PROD=Similar to immunoglobulin joining chain /FL=gb:M90766.1 gb:BC006026.1	742.43335	501.58264	2092.5	1830.8022	1.2318512	2.0	0.34318385	0.38131538	2.82
gb:BC004674.1 /DB_XREF=gi:13435593 /FEA=FLmRNA /CNT=195 /TID=Mm.28028.1 /TIER=FL+Stack /STK=130 /UG=Mm.28028 /DEF=Mus musculus, Similar to RNA binding motif protein 10, clone MGC:7826 IMAGE:3500403, mRNA, complete cds. /PROD=Similar to RNA binding motif protein 10 /FL=gb:BC004674.1	659.73334	137.62909	991.30005	453.94376	1.2106925	2.0	0.3496703	0.38599968	1.50
gb:BC007159.1 /DB_XREF=gi:13938088 /FEA=FLmRNA /CNT=1 /TID=Mm.212861.1 /TIER=FL /STK=1 /UG=Mm.212861 /DEF=Mus musculus, Similar to L1 repeat, Tf subfamily, member 30, clone MGC:7372 IMAGE:3487559, mRNA, complete cds. /PROD=Similar to L1 repeat, Tf subfamily, member 30 /FL=gb:BC007159.1	744.03326	325.80676	2445.2	2433.6653	1.2000223	2.0	0.35299635	0.3871573	3.29
gb:BC007159.1 /DB_XREF=gi:13938088 /FEA=FLmRNA /CNT=1 /TID=Mm.212861.1 /TIER=FL /STK=1 /UG=Mm.212861 /DEF=Mus musculus, Similar to L1 repeat, Tf subfamily, member 30, clone MGC:7372 IMAGE:3487559, mRNA, complete cds. /PROD=Similar to L1 repeat, Tf subfamily, member 30 /FL=gb:BC007159.1	705.5	321.25174	2421.8333	2482.401	1.1876372	2.0	0.35690385	0.3889337	3.43
gb:BC016603.1 /DB_XREF=gi:16741597 /FEA=FLmRNA /CNT=33 /TID=Mm.58104.1 /TIER=FL+Stack /STK=24 /UG=Mm.58104 /DEF=Mus musculus, clone MGC:27940 IMAGE:3587176, mRNA, complete cds. /PROD=Unknown (protein for MGC:27940) /FL=gb:BC016603.1	231.76666	63.020744	363.66666	201.21635	1.0834839	2.0	0.39183238	0.42427713	1.57
gb:NM_015765.1 /DB_XREF=gi:7657199 /GEN=Hsp70-4 /FEA=FLmRNA /CNT=96 /TID=Mm.89341.1 /TIER=FL+Stack /STK=57 /UG=Mm.89341 /LL=50497 /DEF=Mus musculus heat shock protein, 70 kDa 4 (Hsp70-4), mRNA. /PROD=heat shock protein, 70 kDa 4 /FL=gb:BC002056.1 gb:U08215.1 gb:NM_015765.1	154.3	103.89836	243.10002	121.09827	0.96393424	3.0	0.40618578	0.43703532	1.58
gb:AK007826.1 /DB_XREF=gi:12841627 /FEA=mRNA /CNT=37 /TID=Mm.157783.14 /TIER=Stack /STK=27 /UG=Mm.157783 /LL=104911 /UG_GENE=AI893585 /UG_TITLE=expressed sequence AI893585 /DEF=Mus musculus 10 day old male pancreas cDNA, RIKEN full-length enriched library, clone:1810048H17:immunoglobulin heavy chain, (J558 family), full insert sequence.	536.60004	343.23163	1281.9667	1260.6519	0.98811466	2.0	0.42725167	0.45680997	2.39
gb:BC007474.1 /DB_XREF=gi:13938634 /FEA=FLmRNA /CNT=521 /TID=Mm.140785.1 /TIER=FL+Stack /STK=156 /UG=Mm.140785 /LL=20249 /UG_GENE=Scd1 /DEF=Mus musculus, stearoyl-Coenzyme A desaturase 1, clone MGC:6427 IMAGE:3599932, mRNA, complete cds. /PROD=stearoyl-Coenzyme A desaturase 1 /FL=gb:BC007474.1 gb:NM_009127.1	6781.533	901.3425	11328.734	8234.607	0.95077056	2.0	0.44206956	0.4696989	1.67
gb:AK012427.1 /DB_XREF=gi:12849162 /FEA=mRNA /CNT=2 /TID=Mm.331.5 /TIER=ConsEnd /STK=0 /UG=Mm.331 /LL=22190 /UG_GENE=Ubc /UG_TITLE=ubiquitin C /DEF=Mus musculus 11 days embryo whole body cDNA, RIKEN full-length enriched library, clone:2700054O04:homolog to POLYUBIQUITIN UBC (SIMILAR TO UBIQUITIN C), full insert sequence.	1015.76666	444.64703	1562.2001	960.1859	0.8944442	2.0	0.46547025	0.49149033	1.54
gb:NM_010664.1 /DB_XREF=gi:6754481 /GEN=Krt1-18 /FEA=FLmRNA /CNT=380 /TID=Mm.22479.1 /TIER=FL+Stack /STK=149 /UG=Mm.22479 /LL=16668 /DEF=Mus musculus keratin complex 1, acidic, gene 18 (Krt1-18), mRNA. /PROD=keratin complex 1, acidic, gene 18 /FL=gb:M36376.1 gb:NM_010664.1 gb:M11686.1	497.56665	5.105219	883.39996	751.11115	0.8897052	2.0	0.46749777	0.49058408	1.78
gb:BB204720 /DB_XREF=gi:16351882 /DB_XREF=BB204720 /CLONE=A430059G22 /FEA=FLmRNA /CNT=74 /TID=Mm.182434.2 /TIER=Stack /STK=16 /UG=Mm.182434 /UG_TITLE=Mus musculus, Similar to RIKEN cDNA 1700066C05 gene, clone MGC:28125 IMAGE:3980327, mRNA, complete cds /FL=gb:BC020078.1	416.06668	181.6803	901.2	929.3317	0.88737375	2.0	0.4684986	0.48861817	2.17
gb:NM_134051.1 /DB_XREF=gi:19527267 /GEN=AI893585 /FEA=FLmRNA /CNT=3 /TID=Mm.157783.1 /TIER=FL /STK=2 /UG=Mm.157783 /LL=104911 /DEF=Mus musculus expressed sequence AI893585 (AI893585), mRNA. /PROD=expressed sequence AI893585 /FL=gb:NM_134051.1 gb:BC013488.1	1823.8334	1609.4973	5154.1665	6440.4775	0.8689117	2.0	0.47650295	0.493936	2.83
gb:BC019425.1 /DB_XREF=gi:18043870 /FEA=FLmRNA /CNT=5 /TID=Mm.157783.6 /TIER=FL /STK=1 /UG=Mm.157783 /LL=104911 /UG_GENE=AI893585 /DEF=Mus musculus, clone MGC:30346 IMAGE:4459683, mRNA, complete cds. /PROD=Unknown (protein for MGC:30346) /FL=gb:BC019425.1	893.8333	797.18445	2621.0002	3428.4812	0.8498838	2.0	0.48490024	0.4995942	2.93
gb:AF127033.1 /DB_XREF=gi:9937096 /FEA=FLmRNA /CNT=402 /TID=Mm.3760.1 /TIER=FL+Stack /STK=97 /UG=Mm.3760 /LL=14104 /UG_GENE=Fasn /DEF=Mus musculus fatty acid synthase mRNA, complete cds. /PROD=fatty acid synthase /FL=gb:AF127033.1	1379.7334	459.9069	2322.4333	1995.7894	0.797231	2.0	0.5089267	0.52119	1.68
gb:AK010307.1 /DB_XREF=gi:12845653 /FEA=mRNA /CNT=161 /TID=Mm.3014.1 /TIER=ConsEnd /STK=0 /UG=Mm.3014 /LL=66386 /UG_GENE=2310039H15Rik /UG_TITLE=RIKEN cDNA 2310039H15 gene /DEF=Mus musculus ES cells cDNA, RIKEN full-length enriched library, clone:2400010C15:homolog to ACYL CARRIER PROTEIN, MITOCHONDRIAL PRECURSOR (ACP) (NADH-UBIQUINONE OXIDOREDUCTASE 9.6 KDA SUBUNIT) (EC 1.6.5.3) (EC 1.6.99.3) (CI-SDAP), full insert sequ...	1906.5	288.7541	2880.9336	2196.1677	0.7619484	2.0	0.5256836	0.53512704	1.51
gb:BC021831.1 /DB_XREF=gi:18256103 /FEA=mRNA /CNT=1 /TID=Mm.218360.1 /TIER=ConsEnd /STK=0 /UG=Mm.218360 /DEF=Mus musculus, clone IMAGE:3983821, mRNA, partial cds. /PROD=Unknown (protein for IMAGE:3983821)	828.2	179.45923	1305.7333	1128.0948	0.72408855	2.0	0.54425615	0.5507354	1.58
gb:NM_007398.1 /DB_XREF=gi:6680635 /GEN=Ada /FEA=FLmRNA /CNT=80 /TID=Mm.388.1 /TIER=FL+Stack /STK=32 /UG=Mm.388 /LL=11486 /DEF=Mus musculus adenosine deaminase (Ada), mRNA. /PROD=adenosine deaminase /FL=gb:NM_007398.1 gb:AF483481.1 gb:AF483480.1 gb:BC002075.1 gb:M10319.1	208.63332	3.121431	331.93335	308.70947	0.691754	2.0	0.560605	0.56392217	1.59
gb:BC025093.1 /DB_XREF=gi:19263816 /FEA=FLmRNA /CNT=3 /TID=Mm.11480.2 /TIER=FL /STK=1 /UG=Mm.11480 /LL=67239 /UG_GENE=2810470K21Rik /DEF=Mus musculus, RIKEN cDNA 2810470K21 gene, clone MGC:35797 IMAGE:4009314, mRNA, complete cds. /PROD=RIKEN cDNA 2810470K21 gene /FL=gb:BC025093.1	1150.7666	101.20684	2018.6	2279.5244	0.6587568	2.0	0.5777513	0.5777513	1.75
