# Supplementary material for: Protein Phosphatase 2A Improves Cardiac Functional Response to Ischemia and Sepsis
Source: Int J Mol Sci. 2022 Apr 23;23(9):4688. doi: 10.3390/ijms23094688 (PMC9101092; doi:10.3390/ijms23094688)
Supplement: Supplementary file 1 [file ijms-23-04688-s001.zip › Supplementary Table S2.pdf]

Supplementary Table S2. Cardiac gene expression. A GeneChip Mouse Genome 430 2.0 Array (Affymetrix, Santa Clara, CA, United States) was used to analyze the basal mRNA expression in hearts from PP2A-TG (n=3) and WT (n=3) mice. Here, only genes are shown that were expressed in both PP2A-TG and WT and with a 1.5-fold or higher down-regulation in PP2A-TG. For analysis, the TM4 application MeV was used (Saeed et al., 2003) and analyzed by a t-Test (Welch approximation; alpha (overall threshold p-value): 0.05; P-values based on t-distribution). Data are sorted by p value.

Gene descriptions	WT mean	WT SD	PP2A-TG mean	PP2A-TG SD	Absolute t value	Degrees of freedom	Adj p value	False discovery rate	PP2A-TG / WT	WT / PP2A-TG
gb:BC019117.1 /DB_XREF=gi:17512286 /FEA=FLmRNA /CNT=92 /TID=Mm.27932.1 /TIER=FL+Stack /STK=39 /UG=Mm.27932 /LL=83701 /UG_GENE=Ars2-pending /DEF=Mus musculus, clone MGC:29432 IMAGE:3709664, mRNA, complete cds. /PROD=Unknown (protein for MGC:29432) /FL=gb:BC019117.1 gb:NM_031405.1	838.2334	46.69404	477.6	51.20811	9.013391	3.0	0.0028832927	1.0524018	0.57	1.76
gb:AK004894,1 /DB_XREF=gi:12836423 /FEA=mRNA /CNT=70 /TID=Mm,1183,1 /TIER=Stack /STK=31 /UG=Mm,1183 /LL=74153 /UG_GENE=1300004C08Rik /UG_TITLE=RIKEN cDNA 1300004C08 gene /DEF=Mus musculus adult male liver cDNA, RIKEN full-length enriched library, clone:1300004C08:UBE1L (FRAGMENT), full insert sequence,	610.3333	7.245238	400.9	19.546862	17.401026	2.0	0.003286286	0.5997472	0.66	1.52
gb:BC025144.1 /DB_XREF=gi:19264066 /FEA=FLmRNA /CNT=27 /TID=Mm.219658.1 /TIER=FL+Stack /STK=17 /UG=Mm.219658 /DEF=Mus musculus, Similar to GTP binding protein 5 (putative), clone MGC:36295 IMAGE:4483063, mRNA, complete cds. /PROD=Similar to GTP binding protein 5 (putative) /FL=gb:BC025144.1	627.4666	47.652325	374.16666	36.395786	7.316819	3.0	0.0052728374	0.64152855	0.6	1.68
gb:U11548.1 /DB_XREF=gi:511226 /FEA=FLmRNA /CNT=8 /TID=Mm.28587.1 /TIER=FL /STK=1 /UG=Mm.28587 /LL=26407 /UG_GENE=Map3k4 /DEF=Mus musculus TFIIi-associated transactivator factor p17 mRNA, complete cds. /PROD=TFIIi-associated transactivator factor p17 /FL=gb:U85608.1 gb:U11548.1 gb:NM_011948.1	500.83334	49.23356	250.59999	36.55503	7.0680494	3.0	0.0058227624	0.53132707	0.5	2
gb:NM_007981.1 /DB_XREF=gi:6679738 /GEN=Facl2 /FEA=FLmRNA /CNT=147 /TID=Mm.28962.1 /TIER=FL+Stack /STK=45 /UG=Mm.28962 /LL=14081 /DEF=Mus musculus fatty acid Coenzyme A ligase, long chain 2 (Facl2), mRNA. /PROD=fatty acid Coenzyme A ligase, long chain 2 /FL=gb:NM_007981.1 gb:U15977.1	3965.5664	415.30035	2066.2332	226.85002	6.9518514	3.0	0.006105606	0.44570923	0.52	1.92
gb:BG073508 /DB_XREF=gi:12556077 /DB_XREF=H3123D10-3 /CLONE=H3123D10 /FEA=mRNA /CNT=46 /TID=Mm.21795.1 /TIER=Stack /STK=33 /UG=Mm.21795 /UG_TITLE=Mus musculus, Similar to BCL2adenovirus E1B 19kD interacting protein 1, clone MGC:41600 IMAGE:1264175, mRNA, complete cds	559.89996	13.956017	347.1	27.04736	12.110144	2.0	0.0067497385	0.4106091	0.62	1.61
gb:AK003586.1 /DB_XREF=gi:12834343 /FEA=mRNA /CNT=119 /TID=Mm.20488.1 /TIER=Stack /STK=50 /UG=Mm.20488 /LL=76301 /UG_GENE=1110008K06Rik /UG_TITLE=RIKEN cDNA 1110008K06 gene /DEF=Mus musculus 18 days embryo whole body cDNA, RIKEN full-length enriched library, clone:1110008K06:homolog to FLN29 (FLN29 GENE PRODUCT), full insert sequence.	1412.2666	172.08234	591.0333	137.58932	6.455995	3.0	0.007538527	0.39308035	0.42	2.39
gb:BC006668.1 /DB_XREF=gi:13879379 /FEA=FLmRNA /CNT=135 /TID=Mm.20396.1 /TIER=FL+Stack /STK=60 /UG=Mm.20396 /LL=12908 /UG_GENE=Crat /DEF=Mus musculus, Similar to carnitine acetyltransferase, clone MGC:6046 IMAGE:3499169, mRNA, complete cds. /PROD=Similar to carnitine acetyltransferase /FL=gb:BC006668.1 gb:NM_007760.1	4650.733	358.1267	2468.2334	466.95862	6.4237013	3.0	0.007646559	0.34887424	0.53	1.88
gb:BC014685.1 /DB_XREF=gi:15928403 /FEA=FLmRNA /CNT=42 /TID=Mm.170023.1 /TIER=FL+Stack /STK=18 /UG=Mm.170023 /DEF=Mus musculus, clone MGC:25977 IMAGE:4241375, mRNA, complete cds. /PROD=Unknown (protein for MGC:25977) /FL=gb:BC014685.1	660.4333	51.97042	433.63333	39.917454	5.9945416	3.0	0.009296497	0.37702462	0.66	1.52
gb:NM_007533.1 /DB_XREF=gi:6671623 /GEN=Bckdha /FEA=FLmRNA /CNT=211 /TID=Mm.25848.1 /TIER=FL+Stack /STK=76 /UG=Mm.25848 /LL=12039 /DEF=Mus musculus branched chain ketoacid dehydrogenase E1, alpha polypeptide (Bckdha), mRNA. /PROD=branched chain ketoacid dehydrogenase E1, alphapolypeptide /FL=gb:BC003787.1 gb:NM_007533.1 gb:L47335.1	2700.0334	286.70358	1546.7666	192.90247	5.780555	3.0	0.010295443	0.37578368	0.57	1.75
gb:BC004804.1 /DB_XREF=gi:13435929 /FEA=FLmRNA /CNT=277 /TID=Mm.104540.1 /TIER=FL+Stack /STK=115 /UG=Mm.104540 /LL=108124 /UG_GENE=Napa /DEF=Mus musculus, RIKEN cDNA 1500039N14 gene, clone MGC:6399 IMAGE:3584869, mRNA, complete cds. /PROD=RIKEN cDNA 1500039N14 gene /FL=gb:NM_025898.1 gb:BC004804.1	3788.9336	236.00606	2382.4668	356.9891	5.692439	3.0	0.010747662	0.35662696	0.63	1.59
gb:AB056479.1 /DB_XREF=gi:15341205 /FEA=FLmRNA /CNT=427 /TID=Mm.43822.1 /TIER=FL+Stack /STK=32 /UG=Mm.43822 /LL=12974 /UG_GENE=Cs /DEF=Mus musculus mRNA for citrate synthase, complete cds. /PROD=citrate synthase /FL=gb:BC013554.1 gb:NM_026444.1 gb:AB056479.1	3668.7334	270.4928	2305.8333	327.08664	5.5616684	3.0	0.011467959	0.34881708	0.63	1.59
gb:BC018375.1 /DB_XREF=gi:17390875 /FEA=FLmRNA /CNT=59 /TID=Mm.4451.1 /TIER=FL+Stack /STK=30 /UG=Mm.4451 /LL=15205 /UG_GENE=Hes1 /DEF=Mus musculus, hairy and enhancer of split 1, (Drosophila), clone MGC:25356 IMAGE:4505427, mRNA, complete cds. /PROD=hairy and enhancer of split 1, (Drosophila) /FL=gb:NM_008235.1 gb:BC018375.1	557.3333	50.158768	345.19998	46.835567	5.354062	3.0	0.012746805	0.35789105	0.62	1.61
gb:NM_008916.1 /DB_XREF=gi:6679448 /GEN=Pps /FEA=FLmRNA /CNT=174 /TID=Mm.1458.1 /TIER=FL+Stack /STK=115 /UG=Mm.1458 /LL=19062 /DEF=Mus musculus putative phosphatase (Pps), mRNA. /PROD=putative phosphatase /FL=gb:AF483523.1 gb:AF483522.1 gb:NM_008916.1 gb:U96724.1	1440.2001	142.18462	787.8	161.60974	5.2495723	3.0	0.013461299	0.3509553	0.55	1.83
gb:AK004710.1 /DB_XREF=gi:12836083 /GEN=Slc9a3r2 /FEA=mRNA /CNT=23 /TID=Mm.21587.2 /TIER=Stack /STK=16 /UG=Mm.21587 /LL=65962 /UG_TITLE=solute carrier family 9 (sodiumhydrogen exchanger), isoform 3 regulator 2 /DEF=Mus musculus adult male lung cDNA, RIKEN full-length enriched library, clone:1200011K07:solute carrier family 9 (sodiumhydrogen exchanger), isoform 3 regulator 2, full insert sequence.	764.56665	76.69448	400.46667	93.46831	5.215931	3.0	0.013702431	0.33342582	0.52	1.91
gb:AK012404.1 /DB_XREF=gi:12849126 /FEA=mRNA /CNT=49 /TID=Mm.172946.1 /TIER=Stack /STK=15 /UG=Mm.172946 /LL=72579 /UG_GENE=2700049M22Rik /UG_TITLE=RIKEN cDNA 2700049M22 gene /DEF=Mus musculus 11 days embryo whole body cDNA, RIKEN full-length enriched library, clone:2700049M22:hypothetical protein, full insert sequence.	717.3	14.961293	416.63333	60.370216	8.372979	2.0	0.013965844	0.31859583	0.58	1.72
gb:AK004947.1 /DB_XREF=gi:12836517 /GEN=Csf1r /FEA=FLmRNA /CNT=97 /TID=Mm.22574.1 /TIER=Stack /STK=9 /UG=Mm.22574 /LL=12978 /UG_TITLE=colony stimulating factor 1 receptor /DEF=Mus musculus adult male liver cDNA, RIKEN full-length enriched library, clone:1300008N20:colony stimulating factor 1 receptor, full insert sequence. /FL=gb:NM_007779.1	1033.4667	80.77104	583.6	131.73853	5.0423894	3.0	0.015040075	0.32291928	0.56	1.77
gb:NM_007906.1 /DB_XREF=gi:6681272 /GEN=Eef1a2 /FEA=FLmRNA /CNT=64 /TID=Mm.2645.1 /TIER=FL+Stack /STK=31 /UG=Mm.2645 /LL=13628 /DEF=Mus musculus eukaryotic translation elongation factor 1 alpha 2 (Eef1a2), mRNA. /PROD=eukaryotic translation elongation factor 1 alpha2 /FL=gb:L26479.1 gb:NM_007906.1 gb:BC018235.1	17134.666	2077.833	9926.967	1596.6765	4.7641034	3.0	0.017563034	0.3561393	0.58	1.73
gb:NM_029632.1 /DB_XREF=gi:18390326 /GEN=Ppp1r11 /FEA=FLmRNA /CNT=146 /TID=Mm.46176.1 /TIER=FL+Stack /STK=58 /UG=Mm.46176 /LL=76497 /DEF=Mus musculus protein phosphatase 1, regulatory (inhibitor) subunit 11 (Ppp1r11), mRNA. /PROD=protein phosphatase 1, regulatory (inhibitor)subunit 11 /FL=gb:NM_029632.1	3319.6667	170.52585	1640.5333	382.8957	6.9386435	2.0	0.020145172	0.38699934	0.49	2.02
gb:BC008103.1 /DB_XREF=gi:14198073 /FEA=FLmRNA /CNT=64 /TID=Mm.196749.1 /TIER=FL+Stack /STK=24 /UG=Mm.196749 /DEF=Mus musculus, Similar to hypothetical protein FLJ10504, clone MGC:11722 IMAGE:3966815, mRNA, complete cds. /PROD=Similar to hypothetical protein FLJ10504 /FL=gb:BC008103.1	444.4	49.412437	264.86667	48.947556	4.470925	3.0	0.020850305	0.38051805	0.6	1.68
gb:AK020444.1 /DB_XREF=gi:12861029 /FEA=mRNA /CNT=70 /TID=Mm.36699.1 /TIER=Stack /STK=42 /UG=Mm.36699 /LL=77254 /UG_GENE=9430029K10Rik /UG_TITLE=RIKEN cDNA 9430029K10 gene /DEF=Mus musculus 12 days embryo embryonic body between diaphragm region and neck cDNA, RIKEN full-length enriched library, clone:9430029K10:homolog to 54TMP, full insert sequence.	703.2334	78.743706	433.4	69.91444	4.438314	3.0	0.021263769	0.36958456	0.62	1.62
gb:NM_053014.1 /DB_XREF=gi:16519540 /GEN=Agpat3 /FEA=FLmRNA /CNT=6 /TID=Mm.141230.1 /TIER=FL /STK=1 /UG=Mm.141230 /LL=28169 /DEF=Mus musculus 1-acylglycerol-3-phosphate O-acyltransferase 3 (Agpat3), mRNA. /PROD=1-acylglycerol-3-phosphate O-acyltransferase 3 /FL=gb:NM_053014.1	1276.3666	167.27182	751.9666	118.466934	4.431236	3.0	0.021354912	0.3542974	0.59	1.7
gb:AK002443.1 /DB_XREF=gi:12832431 /FEA=FLmRNA /CNT=204 /TID=Mm.180189.1 /TIER=Stack /STK=57 /UG=Mm.180189 /LL=67800 /UG_GENE=Dgat2 /UG_TITLE=diacylglycerol O-acyltransferase 2 /DEF=Mus musculus adult male kidney cDNA, RIKEN full-length enriched library, clone:0610010B06:homolog to WUGSC:H_DJ0747G18.5 PROTEIN (FRAGMENT), full insert sequence. /FL=gb:AF384160.1 gb:NM_026384.1	2753.4001	267.295	1639.7334	105.6523	6.7112303	2.0	0.021489117	0.34102297	0.6	1.68
gb:NM_007476.1 /DB_XREF=gi:6680715 /GEN=Arf1 /FEA=FLmRNA /CNT=64 /TID=Mm.6836.1 /TIER=FL /STK=2 /UG=Mm.6836 /LL=11840 /DEF=Mus musculus ADP-ribosylation factor 1 (Arf1), mRNA. /PROD=ADP-ribosylation factor 1 /FL=gb:D87898.1 gb:NM_007476.1	1804.6666	201.61327	1130.6666	170.99333	4.415943	3.0	0.021553561	0.32779375	0.63	1.6
gb:BC027445.1 /DB_XREF=gi:20071662 /FEA=FLmRNA /CNT=79 /TID=Mm.153891.1 /TIER=FL+Stack /STK=28 /UG=Mm.153891 /LL=19245 /UG_GENE=Ptp4a3 /DEF=Mus musculus, protein tyrosine phosphatase 4a3, clone MGC:36146 IMAGE:4482106, mRNA, complete cds. /PROD=protein tyrosine phosphatase 4a3 /FL=gb:NM_008975.1 gb:AF035645.1 gb:BC027445.1	3634.5999	385.0914	2027.2666	170.24298	6.6120944	2.0	0.022116944	0.3229074	0.56	1.79
gb:NM_028152.1 /DB_XREF=gi:15100155 /GEN=Mms19l /FEA=FLmRNA /CNT=64 /TID=Mm.28412.1 /TIER=FL+Stack /STK=32 /UG=Mm.28412 /LL=72199 /DEF=Mus musculus MMS19 (MET18 S. cerevisiae)-like (Mms19l), mRNA. /PROD=MMS19 (MET18 S. cerevisiae)-like /FL=gb:AF319949.1 gb:NM_028152.1	386.7667	35.257385	238.33333	17.895628	6.5022836	2.0	0.022844655	0.3207038	0.62	1.62
gb:BI412952 /DB_XREF=gi:15173875 /DB_XREF=602988681F1 /CLONE=IMAGE:5144372 /FEA=mRNA /CNT=159 /TID=Mm.35345.1 /TIER=Stack /STK=43 /UG=Mm.35345 /UG_TITLE=Mus musculus, clone IMAGE:5257902, mRNA, partial cds	512.1667	84.50942	246.5	68.90783	4.2199216	3.0	0.024325186	0.32884046	0.48	2.08
gb:BC017689.1 /DB_XREF=gi:17389268 /FEA=FLmRNA /CNT=148 /TID=Mm.19258.1 /TIER=FL+Stack /STK=47 /UG=Mm.19258 /LL=66951 /UG_GENE=2310028D20Rik /DEF=Mus musculus, Similar to thyrotroph embryonic factor, clone MGC:19233 IMAGE:4242534, mRNA, complete cds. /PROD=Similar to thyrotroph embryonic factor /FL=gb:BC017689.1	2227.3333	188.9985	1486.5	238.61961	4.2153683	3.0	0.024394894	0.31800488	0.67	1.5
gb:AK003305.1 /DB_XREF=gi:12833890 /FEA=mRNA /CNT=31 /TID=Mm.46367.1 /TIER=Stack /STK=12 /UG=Mm.46367 /LL=68453 /UG_GENE=1110002J19Rik /UG_TITLE=RIKEN cDNA 1110002J19 gene /DEF=Mus musculus 18 days embryo whole body cDNA, RIKEN full-length enriched library, clone:1110002J19:unclassifiable, full insert sequence.	5818.1997	927.7161	3272.6667	519.24005	4.1471405	3.0	0.025470959	0.32058278	0.56	1.78
gb:AF136571.1 /DB_XREF=gi:15076824 /FEA=FLmRNA /CNT=14 /TID=Mm.10211.2 /TIER=FL /STK=1 /UG=Mm.10211 /LL=12499 /UG_GENE=Entpd5 /DEF=Mus musculus Pcph proto-oncogene protein mRNA, complete cds. /PROD=Pcph proto-oncogene protein /FL=gb:AF136571.1	602.6667	72.00232	401.03336	45.249798	4.106742	3.0	0.026137074	0.31800106	0.67	1.5
gb:AK011603.1 /DB_XREF=gi:12847833 /FEA=mRNA /CNT=56 /TID=Mm.21593.1 /TIER=Stack /STK=41 /UG=Mm.21593 /LL=70201 /UG_GENE=2610028P08Rik /UG_TITLE=RIKEN cDNA 2610028P08 gene /DEF=Mus musculus 10 days embryo whole body cDNA, RIKEN full-length enriched library, clone:2610028P08:homolog to CDNA FLJ20128 FIS, CLONE COL06181, full insert sequence.	428.7333	51.981567	236.03333	62.753677	4.095951	3.0	0.02631879	0.30988252	0.55	1.82
gb:NM_011179.1 /DB_XREF=gi:7242190 /GEN=Psap /FEA=FLmRNA /CNT=332 /TID=Mm.3363.1 /TIER=FL /STK=1 /UG=Mm.3363 /LL=19156 /DEF=Mus musculus prosaposin (Psap), mRNA. /PROD=prosaposin /FL=gb:NM_011179.1	5425.933	829.29877	2215.7332	1085.7566	4.06974	3.0	0.026767017	0.3053113	0.41	2.45
gb:AK009460.1 /DB_XREF=gi:12844269 /FEA=mRNA /CNT=27 /TID=Mm.218627.2 /TIER=Stack /STK=20 /UG=Mm.218627 /LL=66053 /UG_GENE=Ppil2 /UG_TITLE=peptidylprolyl isomerase (cyclophilin)-like 2 /DEF=Mus musculus adult male tongue cDNA, RIKEN full-length enriched library, clone:2310022D01:homolog to CYCLOPHILIN-LIKE PROTEIN CYP-60, full insert sequence.	726.69995	100.58585	387.8667	104.56621	4.044865	3.0	0.027201526	0.30086538	0.53	1.87
gb:BC024138.1 /DB_XREF=gi:18848248 /FEA=FLmRNA /CNT=26 /TID=Mm.22926.1 /TIER=FL+Stack /STK=11 /UG=Mm.22926 /LL=66968 /UG_GENE=2310076L09Rik /DEF=Mus musculus, RIKEN cDNA 2310076L09 gene, clone MGC:37973 IMAGE:5136320, mRNA, complete cds. /PROD=RIKEN cDNA 2310076L09 gene /FL=gb:BC024138.1	1782.4	251.44804	1036.3667	201.14285	4.012932	3.0	0.027772743	0.29814857	0.58	1.72
gb:BC018323.1 /DB_XREF=gi:17390756 /FEA=FLmRNA /CNT=100 /TID=Mm.3459.1 /TIER=FL+Stack /STK=28 /UG=Mm.3459 /LL=13170 /UG_GENE=Dbp /DEF=Mus musculus, D site albumin promoter binding protein, clone MGC:18803 IMAGE:4195116, mRNA, complete cds. /PROD=D site albumin promoter binding protein /FL=gb:NM_016974.1 gb:BC018323.1	2181.5	125.08818	1068.4	314.64484	5.693912	2.0	0.029487085	0.30750817	0.49	2.04
gb:NM_008398.1 /DB_XREF=gi:6680479 /GEN=Itga7 /FEA=FLmRNA /CNT=47 /TID=Mm.179747.1 /TIER=FL+Stack /STK=24 /UG=Mm.179747 /LL=16404 /DEF=Mus musculus integrin alpha 7 (Itga7), mRNA. /PROD=integrin alpha 7 /FL=gb:NM_008398.1 gb:L23423.1	982.36664	117.43058	634.99994	100.058136	3.8998327	3.0	0.029924314	0.3033993	0.65	1.55
gb:AF206720.1 /DB_XREF=gi:6636320 /FEA=FLmRNA /CNT=130 /TID=Mm.38469.1 /TIER=FL+Stack /STK=83 /UG=Mm.38469 /LL=11785 /UG_GENE=Apbb1 /DEF=Mus musculus Fe65 mRNA, complete cds. /PROD=Fe65 /FL=gb:AF206720.1	1659.7334	267.31207	867.3	232.41673	3.8747864	3.0	0.030429503	0.30018294	0.52	1.91
gb:BI408679 /DB_XREF=gi:15169602 /DB_XREF=602964304F1 /CLONE=IMAGE:5119896 /FEA=mRNA /CNT=61 /TID=Mm.29389.1 /TIER=Stack /STK=30 /UG=Mm.29389 /UG_TITLE=Mus musculus, Similar to KIAA1075 protein, clone IMAGE:5099327, mRNA, partial cds	1488.9	179.23257	810.2	246.92395	3.8527741	3.0	0.030882569	0.29663518	0.54	1.84
gb:NM_010119.1 /DB_XREF=gi:7106302 /GEN=Ehd1 /FEA=FLmRNA /CNT=383 /TID=Mm.30169.1 /TIER=FL+Stack /STK=145 /UG=Mm.30169 /LL=13660 /DEF=Mus musculus EH-domain containing 1 (Ehd1), mRNA. /PROD=EH-domain containing 1 /FL=gb:NM_010119.1 gb:AF099186.1 gb:AF173156.1	517.63336	69.581696	323.16666	53.06084	3.8492374	3.0	0.03095617	0.289718	0.62	1.6
gb:NM_009983.1 /DB_XREF=gi:6753555 /GEN=Ctsd /FEA=FLmRNA /CNT=522 /TID=Mm.2147.1 /TIER=FL+Stack /STK=193 /UG=Mm.2147 /LL=13033 /DEF=Mus musculus cathepsin D (Ctsd), mRNA. /PROD=cathepsin D /FL=gb:NM_009983.1	5454.1333	777.9049	3241.0332	625.04315	3.8412483	3.0	0.031123256	0.2839997	0.59	1.68
"gb:U82470.1 /DB_XREF=gi:2690301 /GEN=Got-2 /FEA=FLmRNA /CNT=238 /TID=Mm.18916.1 /TIER=FL /STK=1 /UG=Mm.18916 /LL=14719 /DEF=Mus musculus aspartate aminotransferase precursor (Got-2) mRNA, Got-2-a allele, complete cds; nuclear gene for mitochondrial product. /PROD=aspartate aminotransferase precursor /FL=gb:U82470.1 gb:J02622.1 gb:NM_010325.1"	4684.267	741.73334	2668.0999	544.53687	3.795118	3.0	0.032111008	0.2858663	0.57	1.76
gb:AK003207.1 /DB_XREF=gi:12833731 /FEA=mRNA /CNT=179 /TID=Mm.29998.1 /TIER=Stack /STK=95 /UG=Mm.29998 /LL=66853 /UG_GENE=0610039C21Rik /UG_TITLE=RIKEN cDNA 0610039C21 gene /DEF=Mus musculus 18 days embryo whole body cDNA, RIKEN full-length enriched library, clone:1110001C14:similar to HYPOTHETICAL 19.8 KDA PROTEIN (FRAGMENT), full insert sequence.	4345.7	245.63489	2401.5999	592.12665	5.2527237	2.0	0.034385104	0.2988229	0.55	1.81
gb:BC003447.1 /DB_XREF=gi:13097416 /FEA=FLmRNA /CNT=395 /TID=Mm.12758.1 /TIER=FL+Stack /STK=94 /UG=Mm.12758 /LL=14228 /UG_GENE=Fkbp4 /DEF=Mus musculus, FK506 binding protein 4 (59 kDa), clone MGC:6528 IMAGE:2651490, mRNA, complete cds. /PROD=FK506 binding protein 4 (59 kDa) /FL=gb:NM_010219.1 gb:BC003447.1	1799.4	258.09253	969.4666	96.33808	5.217995	2.0	0.03482062	0.29557037	0.54	1.86
gb:AJ007909.1 /DB_XREF=gi:3336885 /GEN=edr /FEA=mRNA /CNT=7 /TID=Mm.35829.2 /TIER=ConsEnd /STK=5 /UG=Mm.35829 /LL=170942 /DEF=Mus musculus mRNA for erythroid differentiation regulator, partial. /PROD=erythroid differentiation regulator	3188.2668	328.84497	2022.2999	441.76965	3.6670012	3.0	0.03507333	0.2909492	0.63	1.58
gb:BC004613.1 /DB_XREF=gi:13435485 /FEA=FLmRNA /CNT=159 /TID=Mm.22597.1 /TIER=FL+Stack /STK=30 /UG=Mm.22597 /LL=69654 /UG_GENE=2310042E05Rik /DEF=Mus musculus, Similar to dynactin 2 (p50), clone MGC:6712 IMAGE:3585310, mRNA, complete cds. /PROD=Similar to dynactin 2 (p50) /FL=gb:BC004613.1	3519.4336	404.615	2197.7	185.29951	5.144202	2.0	0.035773426	0.29016224	0.62	1.6
gb:BB396904 /DB_XREF=gi:16411476 /DB_XREF=BB396904 /CLONE=C230099N07 /FEA=mRNA /CNT=175 /TID=Mm.1147.1 /TIER=Stack /STK=98 /UG=Mm.1147 /UG_TITLE=Mus musculus calmodulin III (Calm3) mRNA, 3 untranslated region	998.8	150.6383	496.90002	76.62892	5.143623	2.0	0.03578106	0.28391495	0.5	2.01
gb:AK003786.1 /DB_XREF=gi:12834661 /GEN=Nfs1 /FEA=mRNA /CNT=3 /TID=Mm.29169.2 /TIER=ConsEnd /STK=0 /UG=Mm.29169 /LL=18041 /UG_TITLE=nitrogen fixation gene 1 (S. cerevisiae) /DEF=Mus musculus 18 days embryo whole body cDNA, RIKEN full-length enriched library, clone:1110018L11:nitrogen fixation gene 1 (S. cerevisiae), full insert sequence.	542.9	9.142737	352.83334	64.04266	5.088809	2.0	0.036514103	0.28356698	0.65	1.54

gb:NM_023464.1 /DB_XREF=gi:12963686 /GEN=1110003H09Rik /FEA=FLmRNA /CNT=95 /TID=Mm.21444.1 /TIER=FL+Stack /STK=50 /UG=Mm.21444 /LL=68475 /DEF=Mus musculus RIKEN cDNA 1110003H09 gene (1110003H09Rik), mRNA. /PROD=RIKEN cDNA 1110003H09 /FL=gb:NM_023464.1	1704.7666	181.4112	1069.9333	244.86806	3.6081257	3.0	0.03655193	0.27794698	0.63	1.59
gb:BI692446 /DB_XREF=gi:15655075 /DB_XREF=603342955F1 /CLONE=IMAGE:5370829 /FEA=EST /CNT=64 /TID=Mm.27182.2 /TIER=Stack /STK=49 /UG=Mm.27182 /UG_TITLE=Mus musculus, Similar to hypothetical protein MGC4368, clone MGC:28978 IMAGE:4503381, mRNA, complete cds	748.6333	36.21829	337.9	140.35558	4.9078636	2.0	0.039097387	0.29123566	0.45	2.22
gb:BC011344.1 /DB_XREF=gi:15030176 /FEA=FLmRNA /CNT=135 /TID=Mm.220921.2 /TIER=FL+Stack /STK=83 /UG=Mm.220921 /LL=15467 /UG_GENE=Eif2ak1 /DEF=Mus musculus, clone MGC:5739 IMAGE:3481870, mRNA, complete cds. /PROD=Unknown (protein for MGC:5739) /FL=gb:BC011344.1	1550.6333	267.75513	881.4333	195.2781	3.497545	3.0	0.039550956	0.28872198	0.57	1.76
gb:BC027346.1 /DB_XREF=gi:20071091 /FEA=FLmRNA /CNT=82 /TID=Mm.11545.1 /TIER=FL+Stack /STK=51 /UG=Mm.11545 /DEF=Mus musculus, clone MGC:28292 IMAGE:4011421, mRNA, complete cds. /PROD=Unknown (protein for MGC:28292) /FL=gb:BC027346.1	1823.9	194.57033	1146.9333	274.02765	3.4888892	3.0	0.039798737	0.2848341	0.63	1.59
gb:BC028444.1 /DB_XREF=gi:20306467 /FEA=mRNA /CNT=14 /TID=Mm.22152.1 /TIER=ConsEnd /STK=0 /UG=Mm.22152 /LL=69903 /UG_GENE=2610025P08Rik /DEF=Mus musculus, Similar to RIKEN cDNA 2610025P08 gene, clone MGC:41342 IMAGE:1245387, mRNA, complete cds. /PROD=Similar to RIKEN cDNA 2610025P08 gene	393.2333	60.15492	211.43335	25.190737	4.8283324	2.0	0.040318504	0.28300488	0.54	1.86
gb:AK010167.1 /DB_XREF=gi:12845417 /GEN=Tcap /FEA=FLmRNA /CNT=56 /TID=Mm.10762.1 /TIER=Stack /STK=40 /UG=Mm.10762 /LL=21393 /UG_TITLE=titin-cap /DEF=Mus musculus adult male tongue cDNA, RIKEN full-length enriched library, clone:2310075E03:titin-cap, full insert sequence. /FL=gb:NM_011540.1	18152.068	2419.692	11988.667	1911.9014	3.4616609	3.0	0.040591177	0.279543	0.66	1.51
gb:BC003975.1 /DB_XREF=gi:13278300 /FEA=FLmRNA /CNT=232 /TID=Mm.29737.1 /TIER=FL+Stack /STK=79 /UG=Mm.29737 /DEF=Mus musculus, Similar to myeloid leukemia factor 2, clone MGC:7424 IMAGE:3488851, mRNA, complete cds. /PROD=Similar to myeloid leukemia factor 2 /FL=gb:BC003975.1	2250.2666	502.99188	1128.7333	275.59164	3.3869345	3.0	0.042871576	0.28978008	0.5	1.99
gb:NM_133932.1 /DB_XREF=gi:20149313 /GEN=AI987856 /FEA=FLmRNA /CNT=80 /TID=Mm.45116.1 /TIER=FL+Stack /STK=44 /UG=Mm.45116 /LL=101206 /DEF=Mus musculus expressed sequence AI987856 (AI987856), mRNA. /PROD=transcriptional adaptor 3-like /FL=gb:BC026630.1 gb:NM_133932.1	528.86664	70.97079	327.6	26.051298	4.611099	2.0	0.04395412	0.2916955	0.62	1.61
gb:AF206720.1 /DB_XREF=gi:6636320 /FEA=FLmRNA /CNT=130 /TID=Mm.38469.1 /TIER=FL+Stack /STK=83 /UG=Mm.38469 /LL=11785 /UG_GENE=Apbb1 /DEF=Mus musculus Fe65 mRNA, complete cds. /PROD=Fe65 /FL=gb:AF206720.1	1075.2001	207.71707	577.26666	152.6304	3.345871	3.0	0.04419417	0.2880513	0.54	1.86
gb:NM_007383.1 /DB_XREF=gi:6680619 /GEN=Acads /FEA=FLmRNA /CNT=113 /TID=Mm.18759.1 /TIER=FL+Stack /STK=35 /UG=Mm.18759 /LL=11409 /DEF=Mus musculus acetyl-Coenzyme A dehydrogenase, short chain (Acads), mRNA. /PROD=acetyl-Coenzyme A dehydrogenase, short chain /FL=gb:BC016259.1 gb:NM_007383.1 gb:L11163.1	2546.2332	583.8708	1251.8334	331.40588	3.3393998	3.0	0.044407293	0.2843625	0.49	2.03
gb:AK004616.1 /DB_XREF=gi:12835907 /FEA=FLmRNA /CNT=106 /TID=Mm.23996.1 /TIER=Stack /STK=35 /UG=Mm.23996 /LL=24059 /UG_GENE=Slc21a2 /UG_TITLE=solute carrier family 21 (prostaglandin transporter), member 2 /DEF=Mus musculus adult male lung cDNA, RIKEN full-length enriched library, clone:1200006J22:hypothetical protein, full insert sequence. /FL=gb:AF323958.1 gb:NM_026537.1 gb:NM_033314.1	382.33334	51.321564	233.36665	58.21429	3.3246837	3.0	0.044896845	0.2825405	0.61	1.64
gb:AY028963.1 /DB_XREF=gi:19847823 /FEA=FLmRNA /CNT=8 /TID=Mm.38250.1 /TIER=FL /STK=1 /UG=Mm.38250 /LL=56490 /UG_GENE=Zfp288 /DEF=Mus musculus BTBPOZ zinc finger protein DPZF mRNA, complete cds. /PROD=BTBPOZ zinc finger protein DPZF /FL=gb:AF185576.1 gb:NM_019778.1 gb:AY028963.1	1758.9	300.57047	1063.5333	202.91864	3.3210924	3.0	0.04501735	0.27849716	0.6	1.65
gb:BC003744.1 /DB_XREF=gi:13277677 /FEA=FLmRNA /CNT=135 /TID=Mm.3294.1 /TIER=FL+Stack /STK=52 /UG=Mm.3294 /LL=19060 /UG_GENE=Ppp5c /DEF=Mus musculus, protein phosphatase 5, catalytic subunit, clone MGC:5847 IMAGE:3590322, mRNA, complete cds. /PROD=protein phosphatase 5, catalytic subunit /FL=gb:BC003744.1 gb:AF018262.1	1179.0	253.04428	629.6667	141.64098	3.2810674	3.0	0.046388563	0.2821971	0.53	1.87
gb:NM_007876.1 /DB_XREF=gi:6681216 /GEN=Dpep1 /FEA=FLmRNA /CNT=49 /TID=Mm.20388.1 /TIER=FL /STK=7 /UG=Mm.20388 /LL=13479 /DEF=Mus musculus dipeptidase 1 (renal) (Dpep1), mRNA. /PROD=dipeptidase 1 (renal) /FL=gb:NM_007876.1 gb:BC003492.1	2660.8667	501.4193	1360.6333	475.69363	3.25838	3.0	0.04718934	0.28236243	0.51	1.96
Mus musculus /REF=M32599 /DEF=Mouse glyceraldehyde-3-phosphate dehydrogenase mRNA, complete cds /LEN=1228 (_5, _M, _3 represent transcript regions 5 prime, Middle, and 3 prime respectively)	34215.402	7139.477	16254.234	6410.5503	3.2422204	3.0	0.0477704	0.28122896	0.48	2.11
gb:BC025440.1 /DB_XREF=gi:19343555 /FEA=FLmRNA /CNT=93 /TID=Mm.22668.2 /TIER=FL+Stack /STK=21 /UG=Mm.22668 /LL=15356 /UG_GENE=Hmgcl /DEF=Mus musculus, 3-hydroxy-3-methylglutaryl-Coenzyme A lyase, clone MGC:29242 IMAGE:5049759, mRNA, complete cds. /PROD=3-hydroxy-3-methylglutaryl-Coenzyme A lyase /FL=gb:BC025440.1	2477.4	257.76486	1629.1998	373.01678	3.2401416	3.0	0.047845803	0.27720186	0.66	1.52
gb:BC008273.1 /DB_XREF=gi:14198433 /FEA=FLmRNA /CNT=243 /TID=Mm.22514.1 /TIER=FL+Stack /STK=139 /UG=Mm.22514 /LL=66043 /UG_GENE=0610008F14Rik /DEF=Mus musculus, RIKEN cDNA 0610008F14 gene, clone MGC:6312 IMAGE:2811419, mRNA, complete cds. /PROD=RIKEN cDNA 0610008F14 gene /FL=gb:BC008273.1	8549.866	1705.65	4106.4336	406.7802	4.3891153	2.0	0.048188154	0.27482307	0.48	2.08
gb:NM_008733.1 /DB_XREF=gi:6679123 /GEN=Nrap /FEA=FLmRNA /CNT=12 /TID=Mm.6384.1 /TIER=FL /STK=2 /UG=Mm.6384 /LL=18175 /DEF=Mus musculus nebulin-related anchoring protein (Nrap), mRNA. /PROD=nebulin-related anchoring protein /FL=gb:NM_008733.1 gb:U76618.1	4659.6	864.4257	2685.7666	622.2885	3.2097685	3.0	0.048964903	0.27495676	0.58	1.73
gb:AK009733.1 /DB_XREF=gi:12844708 /FEA=mRNA /CNT=6 /TID=Mm.41064.2 /TIER=ConsEnd /STK=1 /UG=Mm.41064 /LL=19358 /UG_GENE=Rad23a /UG_TITLE=RAD23a homolog (S. cerevisiae) /DEF=Mus musculus adult male tongue cDNA, RIKEN full-length enriched library, clone:2310040P19:hypothetical protein, full insert sequence.	583.5	161.7751	221.66667	113.26625	3.1734707	3.0	0.05034606	0.278429	0.38	2.63
gb:NM_019502.1 /DB_XREF=gi:9507186 /GEN=Fxc1 /FEA=FLmRNA /CNT=70 /TID=Mm.4050.1 /TIER=FL+Stack /STK=33 /UG=Mm.4050 /LL=14356 /DEF=Mus musculus fractured callus expressed transcript 1 (Fxc1), mRNA. /PROD=fractured callus expressed transcript 1 /FL=gb:NM_019502.1 gb:AF150103.1 gb:BC009158.1	1996.7001	257.0728	1280.0667	302.79193	3.1249733	3.0	0.052269127	0.28474975	0.64	1.56
gb:NM_021372.1 /DB_XREF=gi:10946705 /GEN=AB041541 /FEA=FLmRNA /CNT=84 /TID=Mm.103476.1 /TIER=FL+Stack /STK=53 /UG=Mm.103476 /LL=58172 /DEF=Mus musculus hypothetical protein, MNCb-1504 (AB041541), mRNA. /PROD=hypothetical protein, MNCb-1504 /FL=gb:NM_021372.1 gb:BC014726.1	712.9666	119.195244	415.06665	114.41949	3.1228788	3.0	0.05235425	0.28101915	0.58	1.72
gb:BC011116.1 /DB_XREF=gi:15029789 /FEA=FLmRNA /CNT=246 /TID=Mm.104975.1 /TIER=FL+Stack /STK=90 /UG=Mm.104975 /LL=66905 /UG_GENE=1300012C15Rik /DEF=Mus musculus, RIKEN cDNA 1300012C15 gene, clone MGC:19018 IMAGE:4023354, mRNA, complete cds. /PROD=RIKEN cDNA 1300012C15 gene /FL=gb:NM_025836.1 gb:BC011116.1	1721.4332	308.0201	978.2334	88.368774	4.017092	2.0	0.056745864	0.3001774	0.57	1.76
gb:NM_133734.1 /DB_XREF=gi:19526929 /GEN=0710008A13Rik /FEA=FLmRNA /CNT=134 /TID=Mm.11535.1 /TIER=FL+Stack /STK=76 /UG=Mm.11535 /LL=71700 /DEF=Mus musculus RIKEN cDNA 0710008A13 gene (0710008A13Rik), mRNA. /PROD=RIKEN cDNA 0710008A13 /FL=gb:NM_133734.1 gb:BC008545.1	2924.9336	463.44464	1932.3334	332.68933	3.0135894	3.0	0.057048753	0.2974685	0.66	1.51
gb:NM_133708.1 /DB_XREF=gi:19526883 /GEN=1810012N01Rik /FEA=FLmRNA /CNT=113 /TID=Mm.23951.1 /TIER=FL+Stack /STK=41 /UG=Mm.23951 /LL=69080 /DEF=Mus musculus RIKEN cDNA 1810012N01 gene (1810012N01Rik), mRNA. /PROD=RIKEN cDNA 1810012N01 /FL=gb:BC008116.1 gb:NM_133708.1	542.76666	72.52485	358.96667	35.13407	3.9504025	2.0	0.05851166	0.30079937	0.66	1.51
gb:NM_009473.1 /DB_XREF=gi:6678506 /GEN=Nr1h2 /FEA=FLmRNA /CNT=145 /TID=Mm.968.1 /TIER=FL+Stack /STK=95 /UG=Mm.968 /LL=22260 /DEF=Mus musculus nuclear receptor subfamily 1, group H, member 2 (Nr1h2), mRNA. /PROD=nuclear receptor subfamily 1, group H, member 2 /FL=gb:U09419.1 gb:NM_009473.1	792.73334	161.25073	459.53336	107.37135	2.9790237	3.0	0.058642812	0.29728648	0.58	1.73
gb:BC010596.1 /DB_XREF=gi:14714881 /FEA=FLmRNA /CNT=95 /TID=Mm.196239.1 /TIER=FL+Stack /STK=41 /UG=Mm.196239 /LL=66734 /UG_GENE=4922501H04Rik /DEF=Mus musculus, MAP1 light chain 3-like protein 1, clone MGC:6325 IMAGE:3256801, mRNA, complete cds. /PROD=MAP1 light chain 3-like protein 1 /FL=gb:BC010596.1	8346.8	1826.1411	4220.5	181.96098	3.8944106	2.0	0.060056616	0.30028307	0.51	1.98
gb:BC021381.1 /DB_XREF=gi:18204152 /FEA=FLmRNA /CNT=88 /TID=Mm.35661.1 /TIER=FL+Stack /STK=54 /UG=Mm.35661 /DEF=Mus musculus, Similar to hypothetical protein, clone MGC:29235 IMAGE:5043282, mRNA, complete cds. /PROD=Similar to hypothetical protein /FL=gb:BC023015.1 gb:BC021381.1	664.8667	123.048935	409.1	86.23846	2.9482262	3.0	0.060110424	0.2964906	0.62	1.63
gb:BM201133 /DB_XREF=gi:17754756 /DB_XREF=C0220F07-3 /CLONE=C0220F07 /FEA=mRNA /CNT=88 /TID=Mm.19077.1 /TIER=Stack /STK=54 /UG=Mm.19077 /UG_TITLE=Mus musculus, clone IMAGE:4953782, mRNA, partial cds	672.5333	109.87162	406.1333	44.638252	3.890766	2.0	0.06015923	0.29277492	0.6	1.66
gb:NM_021534.1 /DB_XREF=gi:10946965 /GEN=Pxmp4 /FEA=FLmRNA /CNT=69 /TID=Mm.24673.1 /TIER=FL+Stack /STK=12 /UG=Mm.24673 /LL=59038 /DEF=Mus musculus peroxisomal membrane protein 4 (Pxmp4), mRNA. /PROD=Pxmp4 /FL=gb:BC025796.1 gb:NM_021534.1	793.2	83.65937	508.96667	145.05557	2.9399946	3.0	0.06051046	0.29060945	0.64	1.56
gb:BC015301.1 /DB_XREF=gi:15929758 /FEA=FLmRNA /CNT=180 /TID=Mm.22277.2 /TIER=FL+Stack /STK=140 /UG=Mm.22277 /LL=74610 /UG_GENE=4833412N02Rik /DEF=Mus musculus, RIKEN cDNA 4833412N02 gene, clone MGC:19206 IMAGE:4237894, mRNA, complete cds. /PROD=RIKEN cDNA 4833412N02 gene /FL=gb:BC015301.1	415.7333	78.72625	204.73334	96.62972	2.9321463	3.0	0.06089498	0.28865802	0.49	2.03
gb:NM_015797.1 /DB_XREF=gi:7657082 /GEN=Fbxo6b /FEA=FLmRNA /CNT=86 /TID=Mm.27445.1 /TIER=FL+Stack /STK=43 /UG=Mm.27445 /LL=50762 /DEF=Mus musculus f-box only protein 6b (Fbxo6b), mRNA. /PROD=f-box only protein 6b /FL=gb:NM_015797.1 gb:AF176526.1 gb:BC017512.1	790.5	42.08992	491.06665	128.11624	3.8459196	2.0	0.06144299	0.2875217	0.62	1.61
gb:AB006361.1 /DB_XREF=gi:2317285 /FEA=FLmRNA /CNT=130 /TID=Mm.1008.1 /TIER=FL+Stack /STK=89 /UG=Mm.1008 /LL=19215 /UG_GENE=Ptgds /DEF=Mus musculus mRNA for prostaglandin D synthetase, complete cds. /PROD=prostaglandin D synthetase /FL=gb:AB006361.1	5255.2	1303.1003	2508.8	978.30804	2.9193041	3.0	0.06153079	0.28428784	0.48	2.09
gb:NM_026839.1 /DB_XREF=gi:14780885 /GEN=1110055E19Rik /FEA=FLmRNA /CNT=59 /TID=Mm.39046.1 /TIER=FL+Stack /STK=24 /UG=Mm.39046 /LL=68794 /DEF=Mus musculus RIKEN cDNA 1110055E19 gene (1110055E19Rik), mRNA. /PROD=RIKEN cDNA 1110055E19 /FL=gb:NM_026839.1	563.3333	87.33613	292.0	135.3724	2.9172091	3.0	0.0616353	0.28121105	0.52	1.93
gb:BG069616 /DB_XREF=gi:12552185 /DB_XREF=H3078C07-3 /CLONE=H3078C07 /FEA=mRNA /CNT=94 /TID=Mm.70573.1 /TIER=Stack /STK=63 /UG=Mm.70573 /UG_TITLE=Mus musculus, Similar to cyclic GMP stimulated phosphodiesterase, clone IMAGE:3598413, mRNA, partial cds	656.39996	141.72182	368.69998	95.44596	2.9163938	3.0	0.061676037	0.27792287	0.56	1.78
gb:NM_009358.1 /DB_XREF=gi:6678298 /GEN=Tex271 /FEA=FLmRNA /CNT=2 /TID=Mm.88062.1 /TIER=FL /STK=1 /UG=Mm.88062 /LL=21770 /DEF=Mus musculus testis expressed gene 271 (Tex271), mRNA. /PROD=testis expressed gene 271 /FL=gb:NM_009358.1	568.76666	92.02615	364.70004	78.98632	2.914479	3.0	0.061771825	0.27495995	0.64	1.56
gb:NM_008831.1 /DB_XREF=gi:6679298 /GEN=Phb /FEA=FLmRNA /CNT=260 /TID=Mm.2355.1 /TIER=FL+Stack /STK=58 /UG=Mm.2355 /LL=18673 /DEF=Mus musculus prohibitin (Phb), mRNA. /PROD=prohibitin /FL=gb:NM_008831.1	6689.0664	1162.0282	4307.933	824.85596	2.894155	3.0	0.06280016	0.2761694	0.64	1.55
gb:BC010817.1 /DB_XREF=gi:14789892 /FEA=FLmRNA /CNT=138 /TID=Mm.3979.1 /TIER=FL+Stack /STK=25 /UG=Mm.3979 /DEF=Mus musculus, Similar to ubiquitin-like 4, clone MGC:19132 IMAGE:4215699, mRNA, complete cds. /PROD=Similar to ubiquitin-like 4 /FL=gb:BC010817.1	1884.5333	340.92563	1204.4667	226.77893	2.8767273	3.0	0.063699044	0.27678752	0.64	1.56
gb:BC010592.1 /DB_XREF=gi:14714874 /FEA=FLmRNA /CNT=119 /TID=Mm.29553.1 /TIER=FL+Stack /STK=53 /UG=Mm.29553 /LL=66071 /UG_GENE=0610025L15Rik /DEF=Mus musculus, RIKEN cDNA 0610025L15 gene, clone MGC:7046 IMAGE:3156338, mRNA, complete cds. /PROD=RIKEN cDNA 0610025L15 gene /FL=gb:BC010592.1 gb:AB049623.1 gb:NM_023154.1	1405.4667	213.8315	834.73334	270.21564	2.8687627	3.0	0.06411519	0.27531815	0.59	1.68
gb:NM_009652.1 /DB_XREF=gi:6753033 /GEN=Akt1 /FEA=FLmRNA /CNT=192 /TID=Mm.6645.1 /TIER=FL+Stack /STK=75 /UG=Mm.6645 /LL=11651 /DEF=Mus musculus thymoma viral proto-oncogene 1 (Akt1), mRNA. /PROD=thymoma viral proto-oncogene 1 /FL=gb:NM_009652.1	1036.9332	184.56311	632.4667	32.963673	3.7366266	2.0	0.06474314	0.2747819	0.61	1.64
gb:BC022913.1 /DB_XREF=gi:18606366 /FEA=FLmRNA /CNT=27 /TID=Mm.28955.1 /TIER=FL /STK=1 /UG=Mm.28955 /LL=67739 /UG_GENE=4930570C03Rik /DEF=Mus musculus, RIKEN cDNA 4930570C03 gene, clone MGC:28670 IMAGE:4237364, mRNA, complete cds. /PROD=RIKEN cDNA 4930570C03 gene /FL=gb:NM_026353.1 gb:BC022913.1	635.23334	118.75034	370.9	31.421484	3.7272027	2.0	0.065039665	0.27286756	0.58	1.71
gb:AK003821.1 /DB_XREF=gi:12834720 /GEN=Anapc5 /FEA=mRNA /CNT=12 /TID=Mm.45312.2 /TIER=ConsEnd /STK=7 /UG=Mm.45312 /LL=59008 /UG_TITLE=anaphase-promoting complex subunit 5 /DEF=Mus musculus 18 days embryo whole body cDNA, RIKEN full-length enriched library, clone:1110019I16:anaphase-promoting complex subunit 5, full insert sequence.	946.0334	164.83359	477.7667	231.96951	2.8501327	3.0	0.06510194	0.27002507	0.51	1.98
gb:BI076714 /DB_XREF=gi:14515371 /DB_XREF=L0223B10-3 /CLONE=L0223B10 /FEA=mRNA /CNT=167 /TID=Mm.216195.1 /TIER=Stack /STK=18 /UG=Mm.216195 /UG_TITLE=Mus musculus mVL30-1 retroelement mRNA sequence	12693.434	2625.5188	6953.3003	530.13385	3.7118473	2.0	0.065527044	0.2687345	0.55	1.83
gb:AK003526.1 /DB_XREF=gi:12834245 /FEA=mRNA /CNT=4 /TID=Mm.3336.1 /TIER=ConsEnd /STK=0 /UG=Mm.3336 /LL=68480 /UG_GENE=1110007C09Rik /UG_TITLE=RIKEN cDNA 1110007C09 gene /DEF=Mus musculus 18 days embryo whole body cDNA, RIKEN full-length enriched library, clone:1110007C09:hypothetical protein, full insert sequence.	1536.2666	142.37112	866.23334	279.45187	3.7003376	2.0	0.06589585	0.26724428	0.56	1.77
gb:L40934.1 /DB_XREF=gi:722666 /GEN=rab6 /FEA=mRNA /CNT=1 /TID=Mm.22473.2 /TIER=ConsEnd /STK=0 /UG=Mm.22473 /LL=14470 /DEF=Mus musculus rab6rab5-associated protein (rab6) mRNA, partial cds. /PROD=rab6rab5-associated protein	4004.6335	935.89264	1930.6666	393.83847	3.5377922	2.0	0.071441576	0.2865514	0.48	2.07
gb:AK003388.1 /DB_XREF=gi:12834026 /FEA=mRNA /CNT=32 /TID=Mm.41330.1 /TIER=Stack /STK=17 /UG=Mm.41330 /LL=76291 /UG_GENE=1110003O08Rik /UG_TITLE=RIKEN cDNA 1110003O08 gene /DEF=Mus musculus 18 days embryo whole body cDNA, RIKEN full-length enriched library, clone:1110003O08:hypothetical protein, full insert sequence.	1215.3334	282.99225	629.76666	51.017086	3.5270977	2.0	0.07182983	0.28497708	0.52	1.93
gb:NM_008893.1 /DB_XREF=gi:6679410 /GEN=Pola2 /FEA=FLmRNA /CNT=116 /TID=Mm.320.1 /TIER=FL+Stack /STK=85 /UG=Mm.320 /LL=18969 /DEF=Mus musculus DNA polymerase alpha 2, 68 kDa (Pola2), mRNA. /PROD=DNA polymerase alpha 2, 68 kDa /FL=gb:NM_008893.1 gb:BC021424.1 gb:D13546.1	529.0	38.63884	352.3667	78.33501	3.5025952	2.0	0.07273095	0.28544945	0.67	1.5
gb:NM_016738.1 /DB_XREF=gi:7949126 /GEN=Rpl13 /FEA=FLmRNA /CNT=11 /TID=Mm.42578.1 /TIER=FL /STK=2 /UG=Mm.42578 /LL=19898 /DEF=Mus musculus ribosomal protein L13 (Rpl13), mRNA. /PROD=ribosomal protein L13 /FL=gb:U28917.1 gb:NM_016738.1	19578.768	4562.7007	10923.566	3138.9824	2.7068903	3.0	0.07335706	0.28484392	0.56	1.79
gb:NM_053162.1 /DB_XREF=gi:16716448 /GEN=Mrpl34 /FEA=FLmRNA /CNT=111 /TID=Mm.29489.1 /TIER=FL+Stack /STK=96 /UG=Mm.29489 /LL=94065 /DEF=Mus musculus mitochondrial ribosomal protein L34 (Mrpl34), mRNA. /PROD=mitochondrial ribosomal protein L34 /FL=gb:NM_053162.1 gb:AB049653.1	2390.8	640.9675	1195.1666	431.3994	2.6803503	3.0	0.07502657	0.28825998	0.5	2
gb:BC003744.1 /DB_XREF=gi:13277677 /FEA=FLmRNA /CNT=135 /TID=Mm.3294.1 /TIER=FL+Stack /STK=52 /UG=Mm.3294 /LL=19060 /UG_GENE=Ppp5c /DEF=Mus musculus, protein phosphatase 5, catalytic subunit, clone MGC:5847 IMAGE:3590322, mRNA, complete cds. /PROD=protein phosphatase 5, catalytic subunit /FL=gb:BC003744.1 gb:AF018262.1	974.39996	279.2426	455.9	185.42218	2.6792161	3.0	0.075098954	0.2855325	0.47	2.14
gb:AA170590 /DB_XREF=gi:1749133 /DB_XREF=ms84d09.r1 /CLONE=IMAGE:618257 /FEA=FLmRNA /CNT=58 /TID=Mm.40282.1 /TIER=Stack /STK=9 /UG=Mm.40282 /UG_TITLE=Mus musculus, Similar to gephyrin, clone MGC:38765 IMAGE:5358943, mRNA, complete cds /FL=gb:BC027112.1	580.1667	90.362236	372.36667	100.3798	2.664874	3.0	0.07602184	0.28606158	0.64	1.56
gb:AF078667.1 /DB_XREF=gi:4322307 /FEA=FLmRNA /CNT=4 /TID=Mm.29030.2 /TIER=FL /STK=1 /UG=Mm.29030 /LL=20411 /UG_GENE=Sorbs1 /DEF=Mus musculus ponsin-1 mRNA, complete cds. /PROD=ponsin-1 /FL=gb:AF078667.1	4500.3335	454.3644	2470.4	926.83795	3.4062045	2.0	0.07643881	0.28469557	0.55	1.82
gb:NM_026384.1 /DB_XREF=gi:16975489 /GEN=Dgat2 /FEA=FLmRNA /CNT=204 /TID=Mm.180189.1 /TIER=FL+Stack /STK=19 /UG=Mm.180189 /LL=67800 /DEF=Mus musculus diacylglycerol O-acyltransferase 2 (Dgat2), mRNA. /PROD=diacylglycerol O-acyltransferase 2 /FL=gb:AF384160.1 gb:NM_026384.1	926.3333	119.53277	567.86664	201.4024	2.651045	3.0	0.07692504	0.28361252	0.61	1.63
gb:NM_019990.1 /DB_XREF=gi:9910481 /GEN=Sdccag28 /FEA=FLmRNA /CNT=125 /TID=Mm.28896.1 /TIER=FL+Stack /STK=29 /UG=Mm.28896 /LL=56018 /DEF=Mus musculus serologically defined colon cancer antigen 28 (Sdccag28), mRNA. /PROD=serologically defined colon cancer antigen 28 /FL=gb:AB031550.1 gb:NM_019990.1	2981.7666	774.46027	1355.2666	307.86032	3.380319	2.0	0.0774809	0.28280526	0.45	2.2

gb:BC024394.1 /DB_XREF=gi:19354424 /FEA=FLmRNA /CNT=200 /TID=Mm.34570.2 /TIER=FL+Stack /STK=106 /UG=Mm.34570 /LL=20018 /UG_GENE=Rpo1-3 /DEF=Mus musculus, RNA polymerase 1-3 (16 kDa subunit), clone MGC:36184 IMAGE:4987975, mRNA, complete cds. /PROD=RNA polymerase 1-3 (16 kDa subunit) /FL=gb:BC024394.1	1394.3667	266.72238	763.7	316.98685	2.6367848	3.0	0.07787033	0.28141257	0.55	1.83
gb:BC013486.1 /DB_XREF=gi:15488676 /FEA=FLmRNA /CNT=189 /TID=Mm.41389.1 /TIER=FL+Stack /STK=141 /UG=Mm.41389 /LL=66277 /UG_GENE=Klf15 /DEF=Mus musculus, Kruppel-like factor 15, clone MGC:19125 IMAGE:4211067, mRNA, complete cds. /PROD=Kruppel-like factor 15 /FL=gb:NM_023184.1 gb:AF317225.1 gb:BC013486.1	1641.1333	293.1515	993.6667	309.8631	2.6290479	3.0	0.07838921	0.28051043	0.61	1.65
gb:NM_009777.1 /DB_XREF=gi:6753219 /GEN=C1qb /FEA=FLmRNA /CNT=149 /TID=Mm.2570.1 /TIER=FL+Stack /STK=58 /UG=Mm.2570 /LL=12260 /DEF=Mus musculus complement component 1, q subcomponent, beta polypeptide (C1qb), mRNA. /PROD=complement component 1, q subcomponent, betapolypeptide /FL=gb:M22531.1 gb:M36293.1 gb:NM_009777.1	2006.4667	304.08218	1285.4332	365.01932	2.6287265	3.0	0.078410864	0.27786374	0.64	1.56
gb:AF041858.1 /DB_XREF=gi:3241986 /FEA=mRNA /CNT=1 /TID=Mm.30717.5 /TIER=ConsEnd /STK=0 /UG=Mm.30717 /LL=20975 /UG_GENE=Synj2 /DEF=Mus musculus synaptojanin 2 isoform delta mRNA, partial cds. /PROD=synaptojanin 2 isoform delta	608.3667	48.258926	377.56668	109.53727	3.3397484	2.0	0.07915579	0.27780637	0.62	1.61
gb:NM_023371.1 /DB_XREF=gi:12963652 /GEN=Pin1 /FEA=FLmRNA /CNT=173 /TID=Mm.7906.1 /TIER=FL+Stack /STK=110 /UG=Mm.7906 /LL=23988 /DEF=Mus musculus protein (peptidyl-prolyl cistrans isomerase) NIMA-interacting 1 (Pin1), mRNA. /PROD=protein (peptidyl-prolyl cistrans isomerase)NIMA-interacting 1 /FL=gb:NM_023371.1	3336.7666	745.0641	1999.5333	480.16516	2.613035	3.0	0.07947676	0.27627635	0.6	1.67
gb:NM_008155.1 /DB_XREF=gi:6680066 /GEN=Gpi1 /FEA=FLmRNA /CNT=26 /TID=Mm.589.1 /TIER=FL /STK=2 /UG=Mm.589 /LL=14751 /DEF=Mus musculus glucose phosphate isomerase 1 complex (Gpi1), mRNA. /PROD=glucose phosphate isomerase 1 complex /FL=gb:M14220.1 gb:NM_008155.1	6017.467	1636.3217	3145.5999	981.9437	2.6065686	3.0	0.07992121	0.27520037	0.52	1.91
gb:U48800.1 /DB_XREF=gi:1234824 /GEN=VEGF-B /FEA=FLmRNA /CNT=7 /TID=Mm.15607.2 /TIER=FL /STK=1 /UG=Mm.15607 /LL=22340 /DEF=Mus musculus vascular endothelial growth factor B precursor (VEGF-B) mRNA, complete cds. /PROD=vascular endothelial growth factor B precursor /FL=gb:U43837.1 gb:U48800.1	3644.2334	921.23413	1713.7333	417.65958	3.3057415	2.0	0.08060011	0.2749443	0.47	2.13
gb:NM_130881.1 /DB_XREF=gi:18700019 /GEN=MGC6685 /FEA=FLmRNA /CNT=43 /TID=Mm.12267.1 /TIER=FL /STK=2 /UG=Mm.12267 /LL=170715 /DEF=Mus musculus similar to poly(A)-binding protein, cytoplasmic 4 (inducible form) (MGC6685), mRNA. /PROD=similar to poly(A)-binding protein, cytoplasmic4 (inducible form) /FL=gb:NM_130881.1 gb:BC003283.1	2363.5667	555.67523	1319.0	424.0053	2.5884504	3.0	0.081182934	0.27436823	0.56	1.79
gb:NM_130864.1 /DB_XREF=gi:18700003 /GEN=Acaa /FEA=FLmRNA /CNT=123 /TID=Mm.205266.1 /TIER=FL+Stack /STK=62 /UG=Mm.205266 /LL=113868 /DEF=Mus musculus acetyl-Coenzyme A acyltransferase (peroxisomal 3-oxoacyl-Coenzyme A thiolase) (Acaa), mRNA. /PROD=acetyl-Coenzyme A acyltransferase (peroxisomal3-oxoacyl-Coenzyme A thiolase) /FL=gb:NM_130864.1 gb:BC012400.1 gb:BC026669.1	796.2	190.25824	459.66666	122.50037	2.5759327	3.0	0.08206902	0.27481827	0.58	1.73
gb:AK003239.1 /DB_XREF=gi:12833782 /FEA=FLmRNA /CNT=199 /TID=Mm.34244.1 /TIER=Stack /STK=14 /UG=Mm.34244 /LL=67458 /UG_GENE=1200007D18Rik /UG_TITLE=RIKEN cDNA 1200007D18 gene /DEF=Mus musculus 18 days embryo whole body cDNA, RIKEN full-length enriched library, clone:1110001K09:homolog to KIAA1181 PROTEIN (FRAGMENT), full insert sequence. /FL=gb:NM_026170.1 gb:BC005516.1	510.6	123.165634	300.93335	69.6006	2.5669823	3.0	0.08270989	0.27444646	0.59	1.7
gb:NM_053082.1 /DB_XREF=gi:16716364 /GEN=Tm4sf7 /FEA=FLmRNA /CNT=147 /TID=Mm.195498.1 /TIER=FL+Stack /STK=105 /UG=Mm.195498 /LL=20524 /DEF=Mus musculus transmembrane 4 superfamily member 7 (Tm4sf7), mRNA. /PROD=transmembrane 4 superfamily member 7 /FL=gb:NM_053082.1 gb:BC003482.1	1432.9668	371.01614	723.1333	83.90889	3.2321563	2.0	0.083857775	0.27574852	0.5	1.98
gb:NM_015816.1 /DB_XREF=gi:7657316 /GEN=Lsm4-pending /FEA=FLmRNA /CNT=133 /TID=Mm.14585.1 /TIER=FL+Stack /STK=52 /UG=Mm.14585 /LL=50783 /DEF=Mus musculus U6 snRNA-associated SM-like protein 4 (Lsm4-pending), mRNA. /PROD=U6 snRNA-associated SM-like protein 4 /FL=gb:NM_015816.1 gb:BC026747.1	2082.0667	419.71677	1322.8334	300.10544	2.5486553	3.0	0.08404149	0.27388522	0.64	1.57
gb:NM_009045.1 /DB_XREF=gi:6677708 /GEN=Rela /FEA=FLmRNA /CNT=10 /TID=Mm.28170.1 /TIER=FL+Stack /STK=9 /UG=Mm.28170 /LL=19697 /DEF=Mus musculus v-rel reticuloendotheliosis viral oncogene homolog A, (avian) (Rela), mRNA. /PROD=avian reticuloendotheliosis viral (v-rel)oncogene homolog A /FL=gb:NM_009045.1 gb:M61909.1	1960.0332	383.5544	1213.4333	334.97433	2.5393877	3.0	0.08472489	0.2736689	0.62	1.62
gb:NM_011125.1 /DB_XREF=gi:6755111 /GEN=Pltp /FEA=FLmRNA /CNT=67 /TID=Mm.6105.1 /TIER=FL+Stack /STK=32 /UG=Mm.6105 /LL=18830 /DEF=Mus musculus phospholipid transfer protein (Pltp), mRNA. /PROD=phospholipid transfer protein /FL=gb:BC003782.1 gb:NM_011125.1 gb:U37226.1 gb:U28960.1	2012.7001	384.84665	1234.1333	370.92523	2.5229409	3.0	0.085954584	0.27520546	0.61	1.63
gb:NM_008155.1 /DB_XREF=gi:6680066 /GEN=Gpi1 /FEA=FLmRNA /CNT=26 /TID=Mm.589.1 /TIER=FL /STK=2 /UG=Mm.589 /LL=14751 /DEF=Mus musculus glucose phosphate isomerase 1 complex (Gpi1), mRNA. /PROD=glucose phosphate isomerase 1 complex /FL=gb:M14220.1 gb:NM_008155.1	8020.0	2118.5437	4423.6665	1272.875	2.5203187	3.0	0.08615266	0.27344105	0.55	1.81
gb:NM_025529.1 /DB_XREF=gi:13384949 /GEN=2310039H17Rik /FEA=FLmRNA /CNT=18 /TID=Mm.44092.1 /TIER=FL /STK=2 /UG=Mm.44092 /LL=66387 /DEF=Mus musculus RIKEN cDNA 2310039H17 gene (2310039H17Rik), mRNA. /PROD=RIKEN cDNA 2310039H17 /FL=gb:NM_025529.1	3700.1335	1125.2148	1606.6998	191.09187	3.1769488	2.0	0.08642728	0.27194792	0.43	2.3
gb:AF072370.1 /DB_XREF=gi:6682347 /GEN=Unc51.1 /FEA=FLmRNA /CNT=148 /TID=Mm.34216.1 /TIER=FL+Stack /STK=85 /UG=Mm.34216 /LL=22241 /DEF=Mus musculus UNC51.1 serinethreonine kinase (Unc51.1) mRNA, complete cds. /PROD=UNC51.1 serinethreonine kinase /FL=gb:AF072370.1 gb:AF053756.1 gb:NM_009469.1	983.8667	138.9416	596.06665	228.08847	2.514983	3.0	0.08655745	0.27002966	0.61	1.65
gb:AB054000.1 /DB_XREF=gi:14017772 /GEN=Cg10671-like /FEA=FLmRNA /CNT=35 /TID=Mm.36758.1 /TIER=FL+Stack /STK=15 /UG=Mm.36758 /LL=68680 /DEF=Mus musculus Cg10671-like mRNA, complete cds. /PROD=Cg10671-like /FL=gb:AB054000.1	1886.9332	390.19482	1093.9	189.40694	3.1668425	2.0	0.086909845	0.2688313	0.58	1.72
gb:BC024621.1 /DB_XREF=gi:19354257 /FEA=FLmRNA /CNT=208 /TID=Mm.30064.1 /TIER=FL+Stack /STK=62 /UG=Mm.30064 /LL=56486 /UG_GENE=Gabarap /DEF=Mus musculus, GABA(A) receptor-associated protein, clone MGC:25654 IMAGE:4459651, mRNA, complete cds. /PROD=GABA(A) receptor-associated protein /FL=gb:NM_019749.1 gb:BC002126.1 gb:BC024621.1 gb:AF161587.1	10245.833	2222.5093	5753.9663	1048.8822	3.165773	2.0	0.086961135	0.26672953	0.56	1.78
gb:AF250139.1 /DB_XREF=gi:7644381 /FEA=FLmRNA /CNT=159 /TID=Mm.21549.1 /TIER=FL /STK=1 /UG=Mm.21549 /LL=80888 /UG_GENE=Cryac /DEF=Mus musculus small stress protein-like protein HSP22 mRNA, complete cds. /PROD=small stress protein-like protein HSP22 /FL=gb:BC011219.1 gb:NM_030704.1 gb:AF250139.1 gb:AF273453.1	2011.4	564.6096	1067.7667	328.73923	2.501638	3.0	0.08758011	0.2663895	0.53	1.88
gb:NM_023041.1 /DB_XREF=gi:12746415 /GEN=Pxf /FEA=FLmRNA /CNT=210 /TID=Mm.144235.1 /TIER=FL /STK=1 /UG=Mm.144235 /LL=19298 /DEF=Mus musculus peroxisomal farnesylated protein (Pxf), mRNA. /PROD=peroxisomal farnesylated protein /FL=gb:NM_023041.1 gb:BC019767.1	883.8	254.4808	419.06665	37.01004	3.130145	2.0	0.0886949	0.26755074	0.47	2.11
Mus musculus /REF=M32599 /DEF=Mouse glyceraldehyde-3-phosphate dehydrogenase mRNA, complete cds /LEN=1228 (_5, _M, _3 represent transcript regions 5 prime, Middle, and 3 prime respectively)	38164.9	9613.984	22698.932	5409.2744	2.4283547	3.0	0.093467414	0.2796361	0.59	1.68
gb:NM_017393.1 /DB_XREF=gi:8393155 /GEN=Clpp /FEA=FLmRNA /CNT=134 /TID=Mm.15243.1 /TIER=FL+Stack /STK=78 /UG=Mm.15243 /LL=53895 /DEF=Mus musculus caseinolytic protease, ATP-dependent, proteolytic subunit homolog (E. coli) (Clpp), mRNA. /PROD=caseinolytic protease, ATP-dependent,proteolytic subunit homolog (E. coli) /FL=gb:NM_017393.1 gb:BC001998.1	1832.3666	349.50354	1118.9667	371.0742	2.4240038	3.0	0.09383194	0.27844438	0.61	1.64
gb:BC019420.1 /DB_XREF=gi:18043845 /FEA=FLmRNA /CNT=96 /TID=Mm.46654.1 /TIER=FL+Stack /STK=59 /UG=Mm.46654 /DEF=Mus musculus, Similar to chromosome 6 open reading frame 35, clone MGC:30293 IMAGE:5053733, mRNA, complete cds. /PROD=Similar to chromosome 6 open reading frame 35 /FL=gb:BC019420.1	1815.1333	453.52466	927.93335	445.7683	2.416461	3.0	0.09446801	0.27807117	0.51	1.96
gb:NM_031868.1 /DB_XREF=gi:13994194 /GEN=Ppp1ca /FEA=FLmRNA /CNT=386 /TID=Mm.1970.1 /TIER=FL+Stack /STK=206 /UG=Mm.1970 /LL=19045 /DEF=Mus musculus protein phosphatase 1, catalytic subunit, alpha isoform (Ppp1ca), mRNA. /PROD=protein phosphatase 1, catalytic subunit, alphaisoform /FL=gb:U25809.1 gb:NM_031868.1 gb:BC014828.1	5705.3667	1490.353	3345.9333	800.3353	2.4157786	3.0	0.09452581	0.27601534	0.59	1.71
gb:AK010664.1 /DB_XREF=gi:12846269 /FEA=mRNA /CNT=29 /TID=Mm.23836.4 /TIER=Stack /STK=23 /UG=Mm.23836 /LL=70231 /UG_GENE=Grs2-pending /UG_TITLE=golgi reassembly stacking protein 2 /DEF=Mus musculus ES cells cDNA, RIKEN full-length enriched library, clone:2410043M02:golgi reassembly stacking protein 2, full insert sequence.	537.10004	157.27226	241.5	70.8365	2.968276	2.0	0.0972279	0.28165224	0.45	2.22
gb:BC002073.1 /DB_XREF=gi:12805224 /FEA=FLmRNA /CNT=111 /TID=Mm.137.1 /TIER=FL+Stack /STK=50 /UG=Mm.137 /LL=20305 /UG_GENE=Scya6 /DEF=Mus musculus, clone MGC:6215 IMAGE:3492808, mRNA, complete cds. /PROD=Unknown (protein for MGC:6215) /FL=gb:NM_009139.1 gb:AF128192.1 gb:AF128191.1 gb:M58004.1 gb:BC002073.1 gb:AF128190.1 gb:AF128189.1 gb:AF128188.1	1573.6334	216.04079	988.26666	366.27304	2.384262	3.0	0.09724311	0.27947822	0.63	1.59
gb:NM_009519.1 /DB_XREF=gi:6678588 /GEN=Wnt11 /FEA=FLmRNA /CNT=24 /TID=Mm.22182.1 /TIER=FL+Stack /STK=10 /UG=Mm.22182 /LL=22411 /DEF=Mus musculus wingless-related MMTV integration site 11 (Wnt11), mRNA. /PROD=wingless-related MMTV integration site 11 /FL=gb:NM_009519.1	505.4	71.000206	316.5	117.53268	2.3827572	3.0	0.09737522	0.27767152	0.63	1.6
gb:BC006606.1 /DB_XREF=gi:13879271 /FEA=FLmRNA /CNT=447 /TID=Mm.1034.1 /TIER=FL+Stack /STK=230 /UG=Mm.1034 /LL=51792 /UG_GENE=Ppp2r1a /DEF=Mus musculus, protein phosphatase 2 (formerly 2A), regulatory subunit A (PR 65), alpha isoform, clone MGC:7086 IMAGE:3157293, mRNA, complete cds. /PROD=protein phosphatase 2 (formerly 2A), regulatorysubunit A (PR 65), alpha isoform /FL=gb:NM_016891.1 gb:BC006606.1 gb:AB021743.1	2269.7334	651.43805	1033.5333	312.80685	2.9629393	2.0	0.097528845	0.2759537	0.46	2.2
gb:AF302138.1 /DB_XREF=gi:15420074 /FEA=FLmRNA /CNT=158 /TID=Mm.22029.1 /TIER=FL+Stack /STK=88 /UG=Mm.22029 /LL=192292 /UG_GENE=NRBP /DEF=Mus musculus HLS7-interacting protein kinase mRNA, complete cds. /PROD=HLS7-interacting protein kinase /FL=gb:BC018463.1 gb:AF302138.1 gb:BC004756.1	1104.3334	297.56613	577.73334	241.51155	2.3799613	3.0	0.097621255	0.27409044	0.52	1.91
gb:AK006949.1 /DB_XREF=gi:12840262 /GEN=Mgll /FEA=mRNA /CNT=5 /TID=Mm.194795.3 /TIER=ConsEnd /STK=0 /UG=Mm.194795 /LL=23945 /UG_TITLE=monoglyceride lipase /DEF=Mus musculus adult male testis cDNA, RIKEN full-length enriched library, clone:1700074K19:monoglyceride lipase, full insert sequence.	1528.3666	498.10037	664.73334	387.59006	2.3701098	3.0	0.0984942	0.2744304	0.43	2.3
gb:BC021405.1 /DB_XREF=gi:18204743 /FEA=FLmRNA /CNT=55 /TID=Mm.197542.2 /TIER=FL /STK=3 /UG=Mm.197542 /DEF=Mus musculus, prion protein interacting protein, clone MGC:29172 IMAGE:3985260, mRNA, complete cds. /PROD=prion protein interacting protein /FL=gb:BC021405.1	1429.6333	272.46213	914.4666	261.58444	2.362404	3.0	0.099183634	0.27425778	0.64	1.56
gb:BM196939 /DB_XREF=gi:17748959 /DB_XREF=C0340C06-3 /CLONE=C0340C06 /FEA=mRNA /CNT=57 /TID=Mm.220848.1 /TIER=Stack /STK=16 /UG=Mm.220848 /UG_TITLE=Mus musculus, similar to putative, clone IMAGE:5343087, mRNA, partial cds	366.83334	48.94613	229.76666	88.11454	2.355309	3.0	0.09982363	0.27395207	0.63	1.6
gb:BC026631.1 /DB_XREF=gi:20072371 /FEA=mRNA /CNT=3 /TID=Mm.219579.1 /TIER=ConsEnd /STK=0 /UG=Mm.219579 /DEF=Mus musculus, clone IMAGE:4983756, mRNA, partial cds. /PROD=Unknown (protein for IMAGE:4983756)	687.3	287.4756	202.13335	12.589808	2.9203472	2.0	0.09997883	0.2723304	0.29	3.4
gb:BC003744.1 /DB_XREF=gi:13277677 /FEA=FLmRNA /CNT=135 /TID=Mm.3294.1 /TIER=FL+Stack /STK=52 /UG=Mm.3294 /LL=19060 /UG_GENE=Ppp5c /DEF=Mus musculus, protein phosphatase 5, catalytic subunit, clone MGC:5847 IMAGE:3590322, mRNA, complete cds. /PROD=protein phosphatase 5, catalytic subunit /FL=gb:BC003744.1 gb:AF018262.1	1420.2334	425.78183	649.23334	171.36757	2.9095585	2.0	0.10061329	0.27202854	0.46	2.19
gb:NM_130860.1 /DB_XREF=gi:18699997 /GEN=Cdk9 /FEA=FLmRNA /CNT=119 /TID=Mm.27557.1 /TIER=FL+Stack /STK=50 /UG=Mm.27557 /LL=107951 /DEF=Mus musculus cyclin-dependent kinase 9 (CDC2-related kinase) (Cdk9), mRNA. /PROD=cyclin-dependent kinase 9 /FL=gb:AF327431.1 gb:BC003901.1 gb:NM_130860.1	513.26666	108.13493	311.26666	103.22113	2.340426	3.0	0.1011825	0.271556	0.61	1.65
gb:NM_016742.1 /DB_XREF=gi:7949017 /GEN=Cdc37 /FEA=FLmRNA /CNT=193 /TID=Mm.4117.1 /TIER=FL+Stack /STK=68 /UG=Mm.4117 /LL=12539 /DEF=Mus musculus cell division cycle 37 homolog (S. cerevisiae) (Cdc37), mRNA. /PROD=cell division cycle 37 homolog (S. cerevisiae) /FL=gb:U43076.1 gb:NM_016742.1	5240.6997	1181.1212	3308.3665	816.72766	2.3307126	3.0	0.10208148	0.2719689	0.63	1.58
gb:BC002148.1 /DB_XREF=gi:12805356 /FEA=FLmRNA /CNT=59 /TID=Mm.582.2 /TIER=FL+Stack /STK=49 /UG=Mm.582 /LL=11770 /UG_GENE=Fabp4 /DEF=Mus musculus, clone MGC:7265 IMAGE:3484864, mRNA, complete cds. /PROD=Unknown (protein for MGC:7265) /FL=gb:BC002148.1	610.1333	54.303703	384.66666	124.90342	2.8673048	2.0	0.103153974	0.27283478	0.63	1.59
gb:NM_031260.1 /DB_XREF=gi:13994126 /GEN=Mov10l1 /FEA=FLmRNA /CNT=18 /TID=Mm.102985.1 /TIER=FL+Stack /STK=11 /UG=Mm.102985 /LL=83456 /DEF=Mus musculus Moloney leukemia virus 10-like 1 (Mov10l1), mRNA. /PROD=Moloney leukemia virus 10-like 1 /FL=gb:AF285587.1 gb:AF340211.1 gb:NM_031260.1	3885.3	658.83813	2512.8667	787.5243	2.3151417	3.0	0.10354287	0.27189314	0.65	1.55
gb:NM_019652.1 /DB_XREF=gi:12025541 /GEN=Asna1 /FEA=FLmRNA /CNT=69 /TID=Mm.41475.1 /TIER=FL+Stack /STK=26 /UG=Mm.41475 /LL=56495 /DEF=Mus musculus arsA (bacterial) arsenite transporter, ATP-binding, homolog 1 (Asna1), mRNA. /PROD=arsA (bacterial) arsenite transporter,ATP-binding, homolog 1 /FL=gb:AF039405.2 gb:NM_019652.1 gb:BC016453.1 gb:BC018430.1	1830.5668	545.8855	856.0999	226.17563	2.8564322	2.0	0.10382245	0.27067995	0.47	2.14
gb:NM_133777.1 /DB_XREF=gi:19527003 /GEN=6720465F12Rik /FEA=FLmRNA /CNT=180 /TID=Mm.22491.1 /TIER=FL+Stack /STK=71 /UG=Mm.22491 /LL=77891 /DEF=Mus musculus RIKEN cDNA 6720465F12 gene (6720465F12Rik), mRNA. /PROD=RIKEN cDNA 0910001J09 /FL=gb:BC012255.1 gb:NM_133777.1	1367.2666	37.664864	854.89996	308.40576	2.856302	2.0	0.103830494	0.26878107	0.63	1.6
gb:NM_026633.1 /DB_XREF=gi:13386123 /GEN=9530058B02Rik /FEA=FLmRNA /CNT=69 /TID=Mm.34532.1 /TIER=FL+Stack /STK=53 /UG=Mm.34532 /LL=68241 /DEF=Mus musculus RIKEN cDNA 9530058B02 gene (9530058B02Rik), mRNA. /PROD=RIKEN cDNA 9530058B02 /FL=gb:BC024332.1 gb:NM_026633.1	3585.2668	990.28937	1960.7334	43.036335	2.8386865	2.0	0.104926765	0.26970613	0.55	1.83
gb:AK004841.1 /DB_XREF=gi:12836328 /FEA=mRNA /CNT=230 /TID=Mm.214574.1 /TIER=Stack /STK=113 /UG=Mm.214574 /LL=74148 /UG_GENE=1300001I01Rik /UG_TITLE=RIKEN cDNA 1300001I01 gene /DEF=Mus musculus adult male liver cDNA, RIKEN full-length enriched library, clone:1300001I01:homolog to PUTATIVE EUKARYOTIC TRANSLATION INITIATION FACTOR 3 SUBUNIT (EIF-3) (FRAGMENT), full insert sequence.	1360.8667	367.4176	757.6667	41.588963	2.825514	2.0	0.105757274	0.2699399	0.56	1.8
gb:AK011392.1 /DB_XREF=gi:12847485 /FEA=mRNA /CNT=49 /TID=Mm.142612.1 /TIER=Stack /STK=21 /UG=Mm.142612 /LL=75410 /UG_GENE=2610014H22Rik /UG_TITLE=RIKEN cDNA 2610014H22 gene /DEF=Mus musculus 10 days embryo whole body cDNA, RIKEN full-length enriched library, clone:2610014H22:homolog to TRITHORAX HOMOLOG 2 (MIXED LINEAGE LEUKEMIA GENE HOMOLOG 2 PROTEIN), full insert sequence.	539.60004	73.6945	356.16666	117.868835	2.2855523	3.0	0.10639043	0.2696702	0.66	1.52
gb:NM_021423.1 /DB_XREF=gi:10946785 /GEN=Shank3 /FEA=FLmRNA /CNT=22 /TID=Mm.40237.1 /TIER=FL+Stack /STK=13 /UG=Mm.40237 /LL=58234 /DEF=Mus musculus SH3ankyrin domain gene 3 (Shank3), mRNA. /PROD=SH3ankyrin domain gene 3 /FL=gb:NM_021423.1	1375.2334	373.24136	767.3333	270.64325	2.283785	3.0	0.10656348	0.268246	0.56	1.79
gb:NM_027884.1 /DB_XREF=gi:13540491 /GEN=Tns /FEA=FLmRNA /CNT=81 /TID=Mm.179671.1 /TIER=FL+Stack /STK=11 /UG=Mm.179671 /LL=21961 /DEF=Mus musculus tensin (Tns), mRNA. /PROD=tensin /FL=gb:NM_027884.1	1340.1333	317.97845	823.73334	34.520206	2.796436	2.0	0.107623875	0.2690597	0.61	1.63
gb:BC023114.1 /DB_XREF=gi:18605658 /FEA=FLmRNA /CNT=87 /TID=Mm.30487.1 /TIER=FL+Stack /STK=32 /UG=Mm.30487 /LL=26569 /UG_GENE=Slc27a4 /DEF=Mus musculus, clone MGC:28448 IMAGE:4158956, mRNA, complete cds. /PROD=Unknown (protein for MGC:28448) /FL=gb:BC023114.1	480.30002	100.929085	316.69998	73.46618	2.2698915	3.0	0.10793588	0.26800406	0.66	1.52
gb:NM_008686.1 /DB_XREF=gi:6679037 /GEN=Nfe2l1 /FEA=FLmRNA /CNT=207 /TID=Mm.6743.1 /TIER=FL+Stack /STK=97 /UG=Mm.6743 /LL=18023 /DEF=Mus musculus nuclear factor, erythroid derived 2,-like 1 (Nfe2l1), mRNA. /PROD=nuclear factor, erythroid derived 2,-like 1 /FL=gb:NM_008686.1	1239.4	283.19302	807.5334	168.70483	2.269218	3.0	0.10800295	0.2663586	0.65	1.53
gb:BC006631.1 /DB_XREF=gi:13879311 /FEA=FLmRNA /CNT=141 /TID=Mm.27259.1 /TIER=FL+Stack /STK=70 /UG=Mm.27259 /LL=101706 /UG_GENE=AL022610 /DEF=Mus musculus, clone MGC:7184 IMAGE:3481703, mRNA, complete cds. /PROD=Unknown (protein for MGC:7184) /FL=gb:BC006631.1	505.16666	131.59337	295.8	92.27268	2.256301	3.0	0.10929905	0.26774597	0.59	1.71
gb:NM_015749.1 /DB_XREF=gi:7657638 /GEN=Tcn2 /FEA=FLmRNA /CNT=400 /TID=Mm.20948.1 /TIER=FL+Stack /STK=135 /UG=Mm.20948 /LL=21452 /DEF=Mus musculus transcobalamin 2 (Tcn2), mRNA. /PROD=transcobalamin 2 /FL=gb:AF090686.1 gb:NM_015749.1 gb:BC003720.1	1225.7001	330.6885	686.0	250.05399	2.254748	3.0	0.10945612	0.26634324	0.56	1.79

gb:AF108501.1 /DB_XREF=gi:6465944 /GEN=Cacc /FEA=FLmRNA /CNT=90 /TID=Mm.20897.1 /TIER=FL+Stack /STK=9 /UG=Mm.20897 /LL=80797 /DEF=Mus musculus Ca(2+)-sensitive chloride channel 2 (Cacc) mRNA, complete cds. /PROD=Ca(2+)-sensitive chloride channel 2 /FL=gb:AF115852.1 gb:BC008147.1 gb:NM_030601.1 gb:AF108501.1	276.13333	82.13977	148.26668	54.392765	2.2480638	3.0	0.11013537	0.26622126	0.54	1.86
gb:BC008158.1 /DB_XREF=gi:14198199 /FEA=FLmRNA /CNT=80 /TID=Mm.45558.1 /TIER=FL+Stack /STK=22 /UG=Mm.45558 /LL=70110 /UG_GENE=2010008K16Rik /DEF=Mus musculus, RIKEN cDNA 2010008K16 gene, clone MGC:7005 IMAGE:3155505, mRNA, complete cds. /PROD=RIKEN cDNA 2010008K16 gene /FL=gb:BC008158.1	726.6667	108.513115	468.03333	167.21202	2.2472882	3.0	0.11021451	0.26465982	0.64	1.55
gb:NM_011421.1 /DB_XREF=gi:6755581 /GEN=Smpd1 /FEA=FLmRNA /CNT=168 /TID=Mm.4628.1 /TIER=FL+Stack /STK=52 /UG=Mm.4628 /LL=20597 /DEF=Mus musculus sphingomyelin phosphodiesterase 1, acid lysosomal (Smpd1), mRNA. /PROD=sphingomyelin phosphodiesterase 1, acidlysosomal /FL=gb:NM_011421.1 gb:BC011304.1	2998.0667	631.4375	1898.3334	284.94925	2.7495928	2.0	0.11073025	0.26416042	0.63	1.58
gb:AF303106.1 /DB_XREF=gi:13183567 /GEN=Gtrgeo22 /FEA=FLmRNA /CNT=61 /TID=Mm.22623.1 /TIER=FL+Stack /STK=39 /UG=Mm.22623 /DEF=Mus musculus GTRGEO22 (Gtrgeo22) mRNA, complete cds. /PROD=GTRGEO22 /FL=gb:AF303106.1	325.69998	72.91316	214.63332	45.34692	2.2404327	3.0	0.11091705	0.2628878	0.66	1.52
gb:AK011609.1 /DB_XREF=gi:12847844 /FEA=FLmRNA /CNT=116 /TID=Mm.10433.1 /TIER=Stack /STK=45 /UG=Mm.10433 /LL=11632 /UG_GENE=Aip /UG_TITLE=aryl-hydrocarbon receptor-interacting protein /DEF=Mus musculus 10 days embryo whole body cDNA, RIKEN full-length enriched library, clone:2610029G12:aryl-hydrocarbon receptor-interacting protein, full insert sequence. /FL=gb:U85489.1 gb:NM_016666.1	1455.0333	316.4408	926.43335	113.50154	2.7234232	2.0	0.11252104	0.2649689	0.64	1.57
gb:NM_007607.1 /DB_XREF=gi:6671677 /GEN=Car4 /FEA=FLmRNA /CNT=72 /TID=Mm.1641.1 /TIER=FL+Stack /STK=30 /UG=Mm.1641 /LL=12351 /DEF=Mus musculus carbonic anhydrase 4 (Car4), mRNA. /PROD=carbonic anhydrase 4 /FL=gb:NM_007607.1 gb:BC012704.1	917.7334	34.845726	573.5	216.2765	2.7216947	2.0	0.11264075	0.2635505	0.62	1.6
gb:NM_008138.1 /DB_XREF=gi:6680036 /GEN=Gnai2 /FEA=FLmRNA /CNT=101 /TID=Mm.196464.1 /TIER=FL+Stack /STK=9 /UG=Mm.196464 /LL=14678 /DEF=Mus musculus guanine nucleotide binding protein, alpha inhibiting 2 (Gnai2), mRNA. /PROD=guanine nucleotide binding protein, alphainhibiting 2 /FL=gb:M13963.1 gb:NM_008138.1	2521.9333	796.52167	1119.0333	408.70044	2.71419	2.0	0.11316262	0.26308507	0.44	2.25
gb:AB006361.1 /DB_XREF=gi:2317285 /FEA=FLmRNA /CNT=130 /TID=Mm.1008.1 /TIER=FL+Stack /STK=89 /UG=Mm.1008 /LL=19215 /UG_GENE=Ptgds /DEF=Mus musculus mRNA for prostaglandin D synthetase, complete cds. /PROD=prostaglandin D synthetase /FL=gb:AB006361.1	19869.732	4651.599	12197.7	1564.4395	2.707691	2.0	0.113617316	0.2624704	0.61	1.63
gb:AK017621.1 /DB_XREF=gi:12856956 /GEN=Pmscl2 /FEA=mRNA /CNT=1 /TID=Mm.28159.3 /TIER=ConsEnd /STK=0 /UG=Mm.28159 /LL=50912 /UG_TITLE=polymyositisscleroderma autoantigen 2 /DEF=Mus musculus 8 days embryo whole body cDNA, RIKEN full-length enriched library, clone:5730436C18:polymyositisscleroderma autoantigen 2, full insert sequence.	784.7666	59.852512	514.5	162.2831	2.7063615	2.0	0.11371064	0.26103386	0.66	1.53
gb:NM_008049.1 /DB_XREF=gi:6679872 /GEN=Ftl2 /FEA=FLmRNA /CNT=2 /TID=Mm.30357.1 /TIER=FL /STK=1 /UG=Mm.30357 /LL=14337 /DEF=Mus musculus ferritin light chain 2 (Ftl2), mRNA. /PROD=ferritin light chain 2 /FL=gb:NM_008049.1	4378.567	944.25793	2859.5667	252.19649	2.69194	2.0	0.11473004	0.2617279	0.65	1.53
gb:NM_009225.1 /DB_XREF=gi:6678052 /GEN=Snrpb /FEA=FLmRNA /CNT=100 /TID=Mm.88216.1 /TIER=FL+Stack /STK=11 /UG=Mm.88216 /LL=20638 /DEF=Mus musculus small nuclear ribonucleoprotein B (Snrpb), mRNA. /PROD=small nuclear ribonucleoprotein B /FL=gb:NM_009225.1 gb:M58761.1	3224.0664	834.9952	1818.8002	352.37866	2.6856236	2.0	0.115180574	0.26112366	0.56	1.77
gb:AK002358.1 /DB_XREF=gi:12832278 /FEA=mRNA /CNT=204 /TID=Mm.3533.2 /TIER=Stack /STK=41 /UG=Mm.3533 /LL=56551 /UG_GENE=Txn2 /UG_TITLE=thioredoxin 2 /DEF=Mus musculus adult male kidney cDNA, RIKEN full-length enriched library, clone:0610008P06:thioredoxin, mitochondrial, full insert sequence.	1863.7334	600.1047	981.7666	354.21774	2.1921759	3.0	0.116017826	0.2613982	0.53	1.9
gb:NM_010438.1 /DB_XREF=gi:6754205 /GEN=Hk1 /FEA=FLmRNA /CNT=117 /TID=Mm.196605.1 /TIER=FL /STK=2 /UG=Mm.196605 /LL=15275 /DEF=Mus musculus hexokinase 1 (Hk1), mRNA. /PROD=hexokinase 1 /FL=gb:L16949.1 gb:NM_010438.1	1239.2334	485.96017	504.16666	328.2567	2.1710267	3.0	0.118341856	0.26499864	0.41	2.46
gb:NM_007572.1 /DB_XREF=gi:6671649 /GEN=C1qa /FEA=FLmRNA /CNT=119 /TID=Mm.370.1 /TIER=FL+Stack /STK=46 /UG=Mm.370 /LL=12259 /DEF=Mus musculus complement component 1, q subcomponent, alpha polypeptide (C1qa), mRNA. /PROD=complement component 1, q subcomponent, alphapolypeptide /FL=gb:BC002086.1 gb:NM_007572.1	1808.0332	915.5151	389.43335	199.11038	2.6225247	2.0	0.11982115	0.2666751	0.22	4.64
gb:AK003596.1 /DB_XREF=gi:12834361 /FEA=mRNA /CNT=150 /TID=Mm.23758.1 /TIER=Stack /STK=65 /UG=Mm.23758 /LL=73826 /UG_GENE=1110008P04Rik /UG_TITLE=RIKEN cDNA 1110008P04 gene /DEF=Mus musculus 18 days embryo whole body cDNA, RIKEN full-length enriched library, clone:1110008P04:unclassifiable transcript, full insert sequence.	826.93335	127.86151	525.4667	205.98315	2.1537433	3.0	0.120282605	0.2660797	0.64	1.57
gb:BC027166.1 /DB_XREF=gi:20073307 /FEA=mRNA /CNT=224 /TID=Mm.219670.1 /TIER=Stack /STK=22 /UG=Mm.219670 /DEF=Mus musculus, Similar to eukaryotic translation initiation factor 4 gamma, 1, clone IMAGE:4950789, mRNA, partial cds. /PROD=Similar to eukaryotic translation initiationfactor 4 gamma, 1	1914.3334	535.31134	1075.0	149.95964	2.6150708	2.0	0.120386615	0.2647055	0.56	1.78
gb:AK019319.1 /DB_XREF=gi:12859466 /GEN=Apoe /FEA=mRNA /CNT=1 /TID=Mm.138866.2 /TIER=ConsEnd /STK=0 /UG=Mm.138866 /LL=11816 /UG_TITLE=apolipoprotein E /DEF=Mus musculus adult male hippocampus cDNA, RIKEN full-length enriched library, clone:2900019G12:apolipoprotein E, full insert sequence.	15604.3	4270.9517	9267.634	2806.9692	2.147504	3.0	0.12099254	0.26444477	0.59	1.68
gb:NM_008512.1 /DB_XREF=gi:6678719 /GEN=Lrp1 /FEA=FLmRNA /CNT=182 /TID=Mm.7221.1 /TIER=FL+Stack /STK=49 /UG=Mm.7221 /LL=16971 /DEF=Mus musculus low density lipoprotein receptor-related protein 1 (Lrp1), mRNA. /PROD=low density lipoprotein receptor-related protein1 /FL=gb:AF367720.1 gb:NM_008512.1	2616.4001	712.1777	1542.8334	493.1368	2.1465883	3.0	0.12109717	0.263098	0.59	1.7
gb:AA049040 /DB_XREF=gi:1528711 /DB_XREF=mj48e12.r1 /CLONE=IMAGE:479374 /FEA=mRNA /CNT=44 /TID=Mm.29320.1 /TIER=Stack /STK=17 /UG=Mm.29320 /UG_TITLE=Mus musculus, clone IMAGE:5251262, mRNA, partial cds	426.29996	87.15508	284.96667	35.40795	2.6021962	2.0	0.12137216	0.26213515	0.67	1.5
gb:NM_009208.1 /DB_XREF=gi:6678022 /GEN=Slc4a3 /FEA=FLmRNA /CNT=45 /TID=Mm.5053.1 /TIER=FL+Stack /STK=23 /UG=Mm.5053 /LL=20536 /DEF=Mus musculus solute carrier family 4 (anion exchanger), member 3 (Slc4a3), mRNA. /PROD=solute carrier family 4 (anion exchanger),member 3 /FL=gb:M28383.1 gb:NM_009208.1	2166.0	661.7367	1131.3334	191.27336	2.601667	2.0	0.12141292	0.26068068	0.52	1.91
gb:BC016568.1 /DB_XREF=gi:16741509 /FEA=FLmRNA /CNT=62 /TID=Mm.196618.1 /TIER=FL+Stack /STK=36 /UG=Mm.196618 /LL=17083 /UG_GENE=Il1rl1l /DEF=Mus musculus, Similar to putative T1ST2 receptor binding protein, clone MGC:27715 IMAGE:2609517, mRNA, complete cds. /PROD=Similar to putative T1ST2 receptor bindingprotein /FL=gb:BC016568.1	971.10004	237.99785	636.8667	128.1429	2.1417065	3.0	0.12165673	0.25967664	0.66	1.52
gb:AF274027.1 /DB_XREF=gi:14971094 /FEA=FLmRNA /CNT=5 /TID=Mm.2400.2 /TIER=FL /STK=2 /UG=Mm.2400 /LL=14779 /UG_GENE=Gpx4 /DEF=Mus musculus phospholipid hydroperoxide glutathione peroxidase mRNA, complete cds, alternatively spliced. /PROD=phospholipid hydroperoxide glutathioneperoxidase /FL=gb:AF274027.1	23738.666	5152.416	15873.466	1223.0314	2.5725076	2.0	0.12368836	0.2624782	0.67	1.5
gb:AF208292.1 /DB_XREF=gi:11907600 /FEA=FLmRNA /CNT=1 /TID=Mm.20934.3 /TIER=FL /STK=1 /UG=Mm.20934 /LL=15258 /UG_GENE=Hipk2 /DEF=Mus musculus protein kinase HIPK2 mRNA, complete cds. /PROD=protein kinase HIPK2 /FL=gb:AF208292.1	991.60004	246.03113	588.3	115.181015	2.5713825	2.0	0.12377735	0.26114875	0.59	1.69
gb:BC006753.1 /DB_XREF=gi:13879545 /FEA=FLmRNA /CNT=190 /TID=Mm.3634.1 /TIER=FL+Stack /STK=71 /UG=Mm.3634 /DEF=Mus musculus, clone MGC:11670 IMAGE:3709076, mRNA, complete cds. /PROD=Unknown (protein for MGC:11670) /FL=gb:AY028317.1 gb:BC006753.1	1298.4333	74.20587	789.0334	335.8784	2.5650096	2.0	0.12428313	0.26070887	0.61	1.65
gb:NM_009349.1 /DB_XREF=gi:6678280 /GEN=Temt /FEA=FLmRNA /CNT=97 /TID=Mm.299.1 /TIER=FL+Stack /STK=19 /UG=Mm.299 /LL=21743 /DEF=Mus musculus thioether S-methyltransferase (Temt), mRNA. /PROD=thioether S-methyltransferase /FL=gb:M88694.1 gb:NM_009349.1 gb:BC013518.1	3501.0999	800.25867	2259.0667	277.9903	2.539362	2.0	0.12634811	0.26352605	0.65	1.55
gb:NM_007752.1 /DB_XREF=gi:6680996 /GEN=Cp /FEA=FLmRNA /CNT=332 /TID=Mm.13787.1 /TIER=FL+Stack /STK=43 /UG=Mm.13787 /LL=12870 /DEF=Mus musculus ceruloplasmin (Cp), mRNA. /PROD=ceruloplasmin /FL=gb:U49430.1 gb:NM_007752.1	685.9666	216.51062	329.0	199.82114	2.098529	3.0	0.12674259	0.26284686	0.48	2.09
gb:NM_009077.1 /DB_XREF=gi:6677770 /GEN=Rpl18 /FEA=FLmRNA /CNT=2 /TID=Mm.41923.1 /TIER=FL /STK=2 /UG=Mm.41923 /LL=19899 /DEF=Mus musculus ribosomal protein L18 (Rpl18), mRNA. /PROD=ribosomal protein L18 /FL=gb:NM_009077.1 gb:L04128.1	12883.8	2866.7754	8467.8	986.7063	2.5228121	2.0	0.12770614	0.26334882	0.66	1.52
gb:BC016596.1 /DB_XREF=gi:16741583 /FEA=FLmRNA /CNT=125 /TID=Mm.24646.1 /TIER=FL+Stack /STK=107 /UG=Mm.24646 /LL=74504 /UG_GENE=5430419M09Rik /DEF=Mus musculus, clone MGC:27806 IMAGE:3257498, mRNA, complete cds. /PROD=Unknown (protein for MGC:27806) /FL=gb:AF396665.1 gb:BC016596.1	520.8	112.5472	341.0	97.78733	2.0887575	3.0	0.1279285	0.2623253	0.65	1.53
gb:AF038538.1 /DB_XREF=gi:3089387 /GEN=Nspl1 /FEA=FLmRNA /CNT=42 /TID=Mm.24142.1 /TIER=FL+Stack /STK=14 /UG=Mm.24142 /LL=20167 /DEF=Mus musculus brain NSP-like 1 (Nspl1) mRNA, complete cds. /PROD=NSP-like 1 /FL=gb:AF038539.1 gb:AF038538.1 gb:NM_013648.1	835.89996	240.5471	474.03333	63.353172	2.5196848	2.0	0.12796506	0.26093435	0.57	1.76
gb:AK017523.1 /DB_XREF=gi:12856802 /FEA=mRNA /CNT=39 /TID=Mm.29092.2 /TIER=ConsEnd /STK=1 /UG=Mm.29092 /LL=70510 /UG_GENE=5730408C10Rik /UG_TITLE=RIKEN cDNA 5730408C10 gene /DEF=Mus musculus 8 days embryo whole body cDNA, RIKEN full-length enriched library, clone:5730408C10:C3HC4 type (RING finger) Zinc finger containing protein, full insert sequence.	1612.5	484.06375	910.66675	328.9321	2.0770903	3.0	0.12936181	0.262317	0.56	1.77
gb:AK008165.1 /DB_XREF=gi:12842184 /FEA=mRNA /CNT=125 /TID=Mm.30082.1 /TIER=Stack /STK=93 /UG=Mm.30082 /LL=70266 /UG_GENE=2010009K05Rik /UG_TITLE=RIKEN cDNA 2010009K05 gene /DEF=Mus musculus adult male small intestine cDNA, RIKEN full-length enriched library, clone:2010009K05:homolog to KYNURENINE AMINOTRANSFERASEGLUTAMINE TRANSAMINASE K, full insert sequence.	451.53333	114.36959	274.23334	45.350895	2.4960191	2.0	0.12994832	0.26205048	0.61	1.65
gb:NM_019472.1 /DB_XREF=gi:9506908 /GEN=Myo10 /FEA=FLmRNA /CNT=126 /TID=Mm.60590.1 /TIER=FL+Stack /STK=40 /UG=Mm.60590 /LL=17909 /DEF=Mus musculus myosin X (Myo10), mRNA. /PROD=myosin X /FL=gb:NM_019472.1	479.13333	90.41983	304.33334	115.5587	2.063405	3.0	0.13106741	0.26285496	0.64	1.57
gb:NM_009149.1 /DB_XREF=gi:6677904 /GEN=Selel /FEA=FLmRNA /CNT=216 /TID=Mm.488.1 /TIER=FL+Stack /STK=57 /UG=Mm.488 /LL=20340 /DEF=Mus musculus selectin, endothelial cell, ligand (Selel), mRNA. /PROD=selectin, endothelial cell, ligand /FL=gb:NM_009149.1 gb:BC021306.1	4186.1665	1061.3314	2741.0332	597.75024	2.054902	3.0	0.13214059	0.26355913	0.65	1.53
gb:NM_008968.1 /DB_XREF=gi:6679534 /GEN=Ptgis /FEA=FLmRNA /CNT=65 /TID=Mm.2339.1 /TIER=FL+Stack /STK=38 /UG=Mm.2339 /LL=19223 /DEF=Mus musculus prostaglandin I2 (prostacyclin) synthase (Ptgis), mRNA. /PROD=prostaglandin I2 (prostacyclin) synthase /FL=gb:AB001607.1 gb:NM_008968.1	3961.1667	958.07043	2481.9668	417.30057	2.4517064	2.0	0.13377887	0.26537657	0.63	1.6
gb:BB725358 /DB_XREF=gi:16108633 /DB_XREF=BB725358 /CLONE=E630011P21 /FEA=EST /CNT=144 /TID=Mm.29786.2 /TIER=Stack /STK=141 /UG=Mm.29786 /UG_TITLE=Mus musculus, clone MGC:36705 IMAGE:3673890, mRNA, complete cds	270.7333	70.47129	174.93335	41.09371	2.0340204	3.0	0.13482064	0.2659975	0.65	1.55
gb:BC013670.1 /DB_XREF=gi:15489119 /FEA=FLmRNA /CNT=368 /TID=Mm.30074.1 /TIER=FL+Stack /STK=43 /UG=Mm.30074 /DEF=Mus musculus, clone MGC:19042 IMAGE:4188988, mRNA, complete cds. /PROD=Unknown (protein for MGC:19042) /FL=gb:BC013670.1	9587.733	1456.2542	6396.4336	2294.6082	2.0338852	3.0	0.1348382	0.26460186	0.67	1.5
gb:NM_023320.1 /DB_XREF=gi:12963636 /GEN=2810052M02Rik /FEA=FLmRNA /CNT=105 /TID=Mm.29475.1 /TIER=FL+Stack /STK=55 /UG=Mm.29475 /LL=67220 /DEF=Mus musculus RIKEN cDNA 2810052M02 gene (2810052M02Rik), mRNA. /PROD=TNF intracellular domain-interacting protein /FL=gb:NM_023320.1 gb:AF168675.1	730.6333	244.26994	373.46667	74.85	2.4214394	2.0	0.1364862	0.26640356	0.51	1.96
gb:NM_008749.1 /DB_XREF=gi:6679157 /GEN=Nucb /FEA=FLmRNA /CNT=332 /TID=Mm.2283.1 /TIER=FL+Stack /STK=64 /UG=Mm.2283 /LL=18220 /DEF=Mus musculus nucleobindin (Nucb), mRNA. /PROD=nucleobindin /FL=gb:NM_008749.1 gb:M96823.1	2056.5332	457.68692	1335.7333	414.98807	2.020778	3.0	0.13655357	0.26511732	0.65	1.54
gb:BC026486.1 /DB_XREF=gi:20071462 /FEA=FLmRNA /CNT=174 /TID=Mm.31239.1 /TIER=FL+Stack /STK=156 /UG=Mm.31239 /DEF=Mus musculus, Similar to LGN protein, clone MGC:31563 IMAGE:4504055, mRNA, complete cds. /PROD=Similar to LGN protein /FL=gb:BC026486.1	1239.2333	239.45042	746.6333	348.11758	2.0193365	3.0	0.1367438	0.26408195	0.6	1.66
gb:BC021463.1 /DB_XREF=gi:18204796 /FEA=FLmRNA /CNT=46 /TID=Mm.29372.1 /TIER=FL+Stack /STK=27 /UG=Mm.29372 /LL=67675 /UG_GENE=0610039D01Rik /DEF=Mus musculus, Similar to RIKEN cDNA 0610039D01 gene, clone MGC:29169 IMAGE:5053934, mRNA, complete cds. /PROD=Similar to RIKEN cDNA 0610039D01 gene /FL=gb:BC021463.1	2120.3	536.66815	1357.9	103.11544	2.4163818	2.0	0.13694601	0.2630805	0.64	1.56
gb:BC027021.1 /DB_XREF=gi:20071695 /FEA=FLmRNA /CNT=111 /TID=Mm.28577.1 /TIER=FL+Stack /STK=38 /UG=Mm.28577 /LL=68463 /UG_GENE=1110006I11Rik /DEF=Mus musculus, RIKEN cDNA 1110006I11 gene, clone MGC:36524 IMAGE:5374870, mRNA, complete cds. /PROD=RIKEN cDNA 1110006I11 gene /FL=gb:BC027021.1	2301.3	584.0858	1471.8667	408.19916	2.0160584	3.0	0.13717754	0.26214558	0.64	1.56
gb:NM_007405.1 /DB_XREF=gi:6671512 /GEN=Adcy6 /FEA=FLmRNA /CNT=77 /TID=Mm.157091.1 /TIER=FL+Stack /STK=29 /UG=Mm.157091 /LL=11512 /DEF=Mus musculus adenylate cyclase 6 (Adcy6), mRNA. /PROD=adenylate cyclase 6 /FL=gb:M93422.1 gb:NM_007405.1	861.8333	207.73645	544.8333	94.94369	2.4038906	2.0	0.1380909	0.26251656	0.63	1.58
gb:BC006760.1 /DB_XREF=gi:13879559 /FEA=FLmRNA /CNT=160 /TID=Mm.24353.1 /TIER=FL+Stack /STK=69 /UG=Mm.24353 /LL=56418 /UG_GENE=Ykt6-pending /DEF=Mus musculus, prenylated SNARE protein, clone MGC:5770 IMAGE:3257673, mRNA, complete cds. /PROD=prenylated SNARE protein /FL=gb:AF076956.1 gb:NM_019661.1 gb:BC006760.1	1423.2999	314.74496	936.23334	278.5027	2.0073311	3.0	0.13834026	0.26162797	0.66	1.52
gb:BM245572 /DB_XREF=gi:17880842 /DB_XREF=K0726E03-3 /CLONE=K0726E03 /FEA=mRNA /CNT=10 /TID=Mm.204648.1 /TIER=ConsEnd /STK=7 /UG=Mm.204648 /UG_TITLE=Mus musculus adult male kidney cDNA, RIKEN full-length enriched library, clone:0610010O05:lymphocyte antigen 6 complex, locus E, full insert sequence	7878.033	2055.934	5033.4663	1347.9259	2.004116	3.0	0.13877152	0.26109076	0.64	1.57
gb:BC005621.1 /DB_XREF=gi:13542843 /FEA=FLmRNA /CNT=178 /TID=Mm.29097.1 /TIER=FL+Stack /STK=27 /UG=Mm.29097 /LL=110829 /UG_GENE=Lims1l /DEF=Mus musculus, RIKEN cDNA 4921524A02 gene, clone MGC:11585 IMAGE:3711611, mRNA, complete cds. /PROD=RIKEN cDNA 4921524A02 gene /FL=gb:NM_026148.1 gb:BC005621.1	3386.3333	739.64	2136.9666	790.78	1.9985387	3.0	0.13952343	0.26115924	0.63	1.58
gb:AK002926.1 /DB_XREF=gi:12833269 /GEN=Klf13 /FEA=mRNA /CNT=1 /TID=Mm.41170.2 /TIER=ConsEnd /STK=0 /UG=Mm.41170 /LL=50794 /UG_TITLE=Kruppel-like factor 13 /DEF=Mus musculus adult male kidney cDNA, RIKEN full-length enriched library, clone:0610043C13:Kruppel-like factor 13, full insert sequence.	655.4	152.72194	433.86667	51.660942	2.3799775	2.0	0.14032003	0.26131028	0.66	1.51
gb:NM_007752.1 /DB_XREF=gi:6680996 /GEN=Cp /FEA=FLmRNA /CNT=332 /TID=Mm.13787.1 /TIER=FL+Stack /STK=43 /UG=Mm.13787 /LL=12870 /DEF=Mus musculus ceruloplasmin (Cp), mRNA. /PROD=ceruloplasmin /FL=gb:U49430.1 gb:NM_007752.1	4454.0	455.7452	2840.6335	1083.2803	2.3777456	2.0	0.14053063	0.260374	0.64	1.57
gb:NM_019966.1 /DB_XREF=gi:9910433 /GEN=Mlycd /FEA=FLmRNA /CNT=8 /TID=Mm.20260.1 /TIER=FL /STK=3 /UG=Mm.20260 /LL=56690 /DEF=Mus musculus malonyl-CoA decarboxylase (Mlycd), mRNA. /PROD=malonyl-CoA decarboxylase /FL=gb:NM_019966.1	1357.1666	386.15103	839.89996	232.48802	1.9877075	3.0	0.14099741	0.25991946	0.62	1.62
gb:BC024615.1 /DB_XREF=gi:19354240 /FEA=FLmRNA /CNT=95 /TID=Mm.27608.1 /TIER=FL+Stack /STK=74 /UG=Mm.27608 /DEF=Mus musculus, Similar to chromosome 9 open reading frame 16, clone MGC:19388 IMAGE:2812475, mRNA, complete cds. /PROD=Similar to chromosome 9 open reading frame 16 /FL=gb:BC024615.1	906.23334	235.48155	547.0	115.93778	2.3705509	2.0	0.1412125	0.25900784	0.6	1.66
gb:BC027285.1 /DB_XREF=gi:20071022 /FEA=FLmRNA /CNT=65 /TID=Mm.175661.1 /TIER=FL+Stack /STK=42 /UG=Mm.175661 /LL=68713 /UG_GENE=1110036C17Rik /DEF=Mus musculus, Similar to RIKEN cDNA 1110036C17 gene, clone MGC:27862 IMAGE:3492212, mRNA, complete cds. /PROD=Similar to RIKEN cDNA 1110036C17 gene /FL=gb:BC027285.1	1052.9667	369.74796	534.93335	259.89047	1.9853188	3.0	0.14132495	0.25791803	0.51	1.97
gb:BC019124.1 /DB_XREF=gi:17512304 /FEA=FLmRNA /CNT=52 /TID=Mm.200426.1 /TIER=FL+Stack /STK=23 /UG=Mm.200426 /DEF=Mus musculus, Similar to LIM and cysteine-rich domains 1, clone MGC:29184 IMAGE:5007525, mRNA, complete cds. /PROD=Similar to LIM and cysteine-rich domains 1 /FL=gb:BC019124.1	1226.9667	314.50266	799.7666	205.74823	1.9688225	3.0	0.14361149	0.26078704	0.65	1.53

gb:AK019396.1 /DB_XREF=gi:12859581 /FEA=mRNA /CNT=68 /TID=Mm.44226.2 /TIER=Stack /STK=39 /UG=Mm.44226 /LL=68066 /UG_GENE=3010027G13Rik /UG_TITLE=RIKEN cDNA 3010027G13 gene /DEF=Mus musculus 12 days embryo head cDNA, RIKEN full-length enriched library, clone:3010027G13:homolog to HYPOTHETICAL 38.5 KDA PROTEIN, full insert sequence.	1165.7667	342.06116	664.5	142.46161	2.3431087	2.0	0.14385577	0.2599374	0.57	1.75
gb:NM_019722.1 /DB_XREF=gi:9789878 /GEN=Arl2 /FEA=FLmRNA /CNT=74 /TID=Mm.21071.1 /TIER=FL+Stack /STK=39 /UG=Mm.21071 /LL=56327 /DEF=Mus musculus ADP-ribosylation-like 2 (Arl2), mRNA. /PROD=ADP-ribosylation-like 2 /FL=gb:NM_019722.1 gb:AF143680.1	1832.2001	534.95233	1139.4667	293.3553	1.9666193	3.0	0.14392015	0.2587727	0.62	1.61
gb:NM_021443.1 /DB_XREF=gi:10946817 /GEN=Scya8 /FEA=FLmRNA /CNT=15 /TID=Mm.42029.1 /TIER=FL /STK=7 /UG=Mm.42029 /LL=20307 /DEF=Mus musculus small inducible cytokine A8 (Scya8), mRNA. /PROD=small inducible cytokine A8 /FL=gb:AB023418.1 gb:NM_021443.1	517.3	171.56436	271.66666	59.66963	2.3422065	2.0	0.14394383	0.25754657	0.53	1.9
gb:NM_013602.1 /DB_XREF=gi:7305284 /GEN=Mt1 /FEA=FLmRNA /CNT=145 /TID=Mm.219629.1 /TIER=FL+Stack /STK=36 /UG=Mm.219629 /LL=17748 /DEF=Mus musculus metallothionein 1 (Mt1), mRNA. /PROD=metallothionein 1 /FL=gb:NM_013602.1	11612.833	3314.8948	7316.1665	1936.7217	1.9384388	3.0	0.14793773	0.26340133	0.63	1.59
gb:BC026642.1 /DB_XREF=gi:20071854 /FEA=FLmRNA /CNT=89 /TID=Mm.204991.1 /TIER=FL+Stack /STK=49 /UG=Mm.204991 /DEF=Mus musculus, Similar to hypothetical protein FLJ20234, clone MGC:37525 IMAGE:4986113, mRNA, complete cds. /PROD=Similar to hypothetical protein FLJ20234 /FL=gb:BC026642.1	1105.6666	249.39113	739.5334	120.517914	2.2895179	2.0	0.14921854	0.26439208	0.67	1.5
gb:U43884.1 /DB_XREF=gi:1244792 /GEN=Id1B /FEA=FLmRNA /CNT=3 /TID=Mm.444.2 /TIER=FL /STK=1 /UG=Mm.444 /LL=15901 /DEF=Mus musculus transcription factor Id1B (Id1B) mRNA, complete cds. /PROD=Id1B /FL=gb:U43884.1	1394.6	364.95178	889.7	273.0372	1.9186989	3.0	0.15083033	0.26595685	0.64	1.57
gb:BC026751.1 /DB_XREF=gi:20071192 /FEA=FLmRNA /CNT=36 /TID=Mm.41486.1 /TIER=FL /STK=1 /UG=Mm.41486 /LL=70103 /UG_GENE=2700001K05Rik /DEF=Mus musculus, Similar to putative cyclin G1 interacting protein, clone MGC:25516 IMAGE:3153902, mRNA, complete cds. /PROD=Similar to putative cyclin G1 interactingprotein /FL=gb:BC026751.1	1355.3667	312.11258	905.0999	262.02222	1.9137493	3.0	0.15156595	0.2659691	0.67	1.5
gb:BC024610.1 /DB_XREF=gi:19354311 /FEA=FLmRNA /CNT=39 /TID=Mm.46628.2 /TIER=FL+Stack /STK=21 /UG=Mm.46628 /LL=22344 /UG_GENE=Vezf1 /DEF=Mus musculus, vascular endothelial zinc finger 1, clone MGC:28455 IMAGE:4159675, mRNA, complete cds. /PROD=vascular endothelial zinc finger 1 /FL=gb:BC024610.1	1260.9	340.7898	789.93335	122.7772	2.251978	2.0	0.15314065	0.26744658	0.63	1.6
gb:AK011360.1 /DB_XREF=gi:12847432 /FEA=mRNA /CNT=64 /TID=Mm.142187.1 /TIER=Stack /STK=24 /UG=Mm.142187 /LL=69202 /UG_GENE=2610009E16Rik /UG_TITLE=RIKEN cDNA 2610009E16 gene /DEF=Mus musculus 10 days embryo whole body cDNA, RIKEN full-length enriched library, clone:2610009E16:homolog to PARATHYMOSIN (ZINC-BINDING 11.5 KDA PROTEIN), full insert sequence.	3908.8	1215.2148	2384.5	673.47577	1.9002765	3.0	0.15358968	0.2669535	0.61	1.64
gb:NM_009088.1 /DB_XREF=gi:6677792 /GEN=Rpo1-4 /FEA=FLmRNA /CNT=139 /TID=Mm.7387.1 /TIER=FL+Stack /STK=36 /UG=Mm.7387 /LL=20019 /DEF=Mus musculus RNA polymerase 1-4 (194 kDa subunit) (Rpo1-4), mRNA. /PROD=RNA polymerase 1-4 (194 kDa subunit) /FL=gb:NM_009088.1 gb:AF000938.1	442.7667	120.27112	271.9	99.23125	1.8980509	3.0	0.15392701	0.26627183	0.61	1.63
gb:AK007209.1 /DB_XREF=gi:12840620 /GEN=Cappb1 /FEA=mRNA /CNT=2 /TID=Mm.2945.3 /TIER=ConsEnd /STK=1 /UG=Mm.2945 /LL=12345 /UG_TITLE=capping protein beta 1 /DEF=Mus musculus adult male testis cDNA, RIKEN full-length enriched library, clone:1700120C01:capping protein beta 1, full insert sequence.	3347.5	1118.8156	1890.4668	725.1011	1.892879	3.0	0.15471424	0.26637122	0.56	1.77
gb:NM_017476.1 /DB_XREF=gi:8567377 /GEN=Nakap95-pending /FEA=FLmRNA /CNT=87 /TID=Mm.9590.1 /TIER=FL+Stack /STK=37 /UG=Mm.9590 /LL=54194 /DEF=Mus musculus neighbor of A-kinase anchoring protein 95 (Nakap95-pending), mRNA. /PROD=neighbor of A-kinase anchoring protein 95 /FL=gb:AB028921.1 gb:NM_017476.1	538.3333	148.90977	345.46664	20.051022	2.2232726	2.0	0.15623604	0.26772842	0.64	1.56
gb:BC007140.1 /DB_XREF=gi:13938048 /FEA=FLmRNA /CNT=129 /TID=Mm.219663.2 /TIER=FL+Stack /STK=107 /UG=Mm.219663 /LL=14114 /UG_GENE=Fbln1 /DEF=Mus musculus, Similar to fibulin 1, clone MGC:6128 IMAGE:3495754, mRNA, complete cds. /PROD=Similar to fibulin 1 /FL=gb:BC007140.1	2261.4333	914.7857	1040.7667	275.78848	2.2128296	2.0	0.15738344	0.26843438	0.46	2.17
gb:BE984258 /DB_XREF=gi:10656305 /DB_XREF=UI-M-CG0p-bgd-c-01-0-UI.s1 /CLONE=UI-M-CG0p-bgd-c-01-0-UI /FEA=FLmRNA /CNT=156 /TID=Mm.22543.1 /TIER=Stack /STK=15 /UG=Mm.22543 /UG_TITLE=Mus musculus brain and reproductive organ-expressed protein (Bre) mRNA, complete cds /FL=gb:AF440752.1	915.3333	239.33093	607.63336	154.42604	1.8711394	3.0	0.15807502	0.2683599	0.66	1.51
gb:NM_010312.1 /DB_XREF=gi:13937390 /GEN=Gnb2 /FEA=FLmRNA /CNT=367 /TID=Mm.30141.1 /TIER=FL+Stack /STK=141 /UG=Mm.30141 /LL=14693 /DEF=Mus musculus guanine nucleotide binding protein, beta 2 (Gnb2), mRNA. /PROD=guanine nucleotide-binding protein, beta-2subunit /FL=gb:U34960.1 gb:NM_010312.1	1156.7001	482.16302	477.7333	227.77388	2.2053285	2.0	0.15821478	0.26735365	0.41	2.42
gb:NM_011797.1 /DB_XREF=gi:6753263 /GEN=Car14 /FEA=FLmRNA /CNT=41 /TID=Mm.34556.1 /TIER=FL+Stack /STK=16 /UG=Mm.34556 /LL=23831 /DEF=Mus musculus carbonic anhydrase 14 (Car14), mRNA. /PROD=carbonic anhydrase 14 /FL=gb:NM_011797.1 gb:AB005450.1	1273.2001	392.50107	750.2666	284.58118	1.8682411	3.0	0.15852948	0.26665094	0.59	1.7
gb:AK004089.1 /DB_XREF=gi:12835132 /FEA=mRNA /CNT=42 /TID=Mm.29458.1 /TIER=Stack /STK=17 /UG=Mm.29458 /LL=68746 /UG_GENE=1110034G11Rik /UG_TITLE=RIKEN cDNA 1110034G11 gene /DEF=Mus musculus 18 days embryo whole body cDNA, RIKEN full-length enriched library, clone:1110034G11:related to CG12379 PROTEIN, full insert sequence.	2279.8	659.10315	1438.1333	63.327145	2.2016687	2.0	0.15862256	0.26558363	0.63	1.59
gb:U16321.1 /DB_XREF=gi:1185384 /FEA=FLmRNA /CNT=80 /TID=Mm.4269.2 /TIER=FL+Stack /STK=55 /UG=Mm.4269 /LL=21413 /UG_GENE=Tcf4 /DEF=Mus musculus basic transcription factor MITF-2A mRNA, complete cds. /PROD=MITF-2A protein /FL=gb:U16321.1 gb:BC014293.1	1709.9332	440.62988	1114.2999	333.23102	1.8674502	3.0	0.15865374	0.2644229	0.65	1.53
gb:AK008582.1 /DB_XREF=gi:12842855 /FEA=mRNA /CNT=284 /TID=Mm.35779.1 /TIER=Stack /STK=41 /UG=Mm.35779 /LL=71752 /UG_GENE=1300004C11Rik /UG_TITLE=RIKEN cDNA 1300004C11 gene /DEF=Mus musculus adult male small intestine cDNA, RIKEN full-length enriched library, clone:2010319A12:homolog to TFIIIC2 SUBUNIT, full insert sequence.	595.0	171.37785	374.63333	27.457663	2.1991158	2.0	0.15890788	0.2636426	0.63	1.59
gb:AF252873.1 /DB_XREF=gi:7644395 /FEA=FLmRNA /CNT=73 /TID=Mm.30211.1 /TIER=FL /STK=5 /UG=Mm.30211 /LL=57266 /UG_GENE=Scyb14 /DEF=Mus musculus CXC chemokine MIP-2gamma precursor, mRNA, complete cds. /PROD=CXC chemokine MIP-2gamma precursor /FL=gb:AF192557.1 gb:NM_019568.1 gb:AF144754.1 gb:AF152377.1 gb:AF252873.1	540.5	160.72278	317.30002	72.58843	2.1921403	2.0	0.15969104	0.2637431	0.59	1.7
gb:AF332085.1 /DB_XREF=gi:14193736 /GEN=Rad21 /FEA=FLmRNA /CNT=282 /TID=Mm.182628.1 /TIER=FL /STK=2 /UG=Mm.182628 /LL=19357 /DEF=Mus musculus strain ISS Rad21 (Rad21) mRNA, complete cds. /PROD=Rad21 /FL=gb:AF332086.1 gb:AF332085.1 gb:NM_009009.1 gb:D49429.1	823.76666	335.08176	380.09998	241.96138	1.8592664	3.0	0.15994632	0.2629748	0.46	2.17
gb:BC004829.1 /DB_XREF=gi:13435995 /FEA=FLmRNA /CNT=206 /TID=Mm.2079.1 /TIER=FL+Stack /STK=57 /UG=Mm.2079 /LL=20973 /UG_GENE=Syngr2 /DEF=Mus musculus, synaptogyrin 2, clone MGC:6483 IMAGE:2646657, mRNA, complete cds. /PROD=synaptogyrin 2 /FL=gb:AF364049.1 gb:BC004829.1 gb:AF151985.1 gb:NM_009304.1	1586.0668	384.5148	1058.5	310.58392	1.8486891	3.0	0.1616351	0.2645597	0.67	1.5
gb:AK007572.1 /DB_XREF=gi:12841200 /FEA=mRNA /CNT=1 /TID=Mm.161667.1 /TIER=ConsEnd /STK=0 /UG=Mm.161667 /LL=69115 /UG_GENE=1810021D01Rik /UG_TITLE=RIKEN cDNA 1810021D01 gene /DEF=Mus musculus 10 day old male pancreas cDNA, RIKEN full-length enriched library, clone:1810021D01:homolog to CDNA FLJ10479 FIS, CLONE NT2RP2000120 (DC1) (HYPOTHETICAL 39.8 KDA PROTEIN), full insert sequence.	941.5667	57.336903	622.8334	247.26883	2.1749341	2.0	0.16164558	0.2633957	0.66	1.51
gb:NM_026468.1 /DB_XREF=gi:13385959 /GEN=Atp5g2 /FEA=FLmRNA /CNT=334 /TID=Mm.10314.1 /TIER=FL+Stack /STK=154 /UG=Mm.10314 /LL=67942 /DEF=Mus musculus ATP synthase, H+ transporting, mitochondrial F0 complex, subunit c (subunit 9), isoform 2 (Atp5g2), mRNA. /PROD=ATP synthase, H+ transporting, mitochondrial F0complex, subunit c (subunit 9), isoform 2 /FL=gb:NM_026468.1 gb:BC006813.1	13885.966	3905.741	8379.966	1993.4896	2.1748064	2.0	0.16166022	0.2622488	0.6	1.66
gb:BC019442.1 /DB_XREF=gi:18043191 /FEA=FLmRNA /CNT=127 /TID=Mm.185518.1 /TIER=FL+Stack /STK=87 /UG=Mm.185518 /DEF=Mus musculus, clone MGC:25803 IMAGE:4036849, mRNA, complete cds. /PROD=Unknown (protein for MGC:25803) /FL=gb:BC014795.1 gb:BC019442.1	772.5	196.32268	505.13333	85.66692	2.1619692	2.0	0.16314003	0.26347837	0.65	1.53
gb:NM_008713.1 /DB_XREF=gi:6679089 /GEN=Nos3 /FEA=FLmRNA /CNT=49 /TID=Mm.12837.1 /TIER=FL+Stack /STK=25 /UG=Mm.12837 /LL=18127 /DEF=Mus musculus nitric oxide synthase 3, endothelial cell (Nos3), mRNA. /PROD=nitric oxide synthase 3, endothelial cell /FL=gb:U53142.1 gb:NM_008713.1	1535.2333	382.03305	1020.69995	303.48578	1.8265747	3.0	0.16523309	0.26568317	0.66	1.5
gb:AK004695.1 /DB_XREF=gi:12836055 /FEA=mRNA /CNT=184 /TID=Mm.34763.1 /TIER=Stack /STK=111 /UG=Mm.34763 /LL=71732 /UG_GENE=1200011A11Rik /UG_TITLE=RIKEN cDNA 1200011A11 gene /DEF=Mus musculus adult male lung cDNA, RIKEN full-length enriched library, clone:1200011A11:homolog to VACUOLAR PROTEIN SORTING PROTEIN 11, full insert sequence.	578.6333	199.9345	324.93332	53.362926	2.123492	2.0	0.1676879	0.26844773	0.56	1.78
gb:BC019836.1 /DB_XREF=gi:18043722 /FEA=FLmRNA /CNT=420 /TID=Mm.22248.2 /TIER=FL+Stack /STK=125 /UG=Mm.22248 /LL=16010 /UG_GENE=Igfbp4 /DEF=Mus musculus, insulin-like growth factor binding protein 4, clone MGC:29917 IMAGE:5123738, mRNA, complete cds. /PROD=insulin-like growth factor binding protein 4 /FL=gb:BC019836.1	5639.267	1676.5708	3555.6335	1113.3978	1.7931857	3.0	0.17084232	0.27230328	0.63	1.59
gb:BC016669.1 /DB_XREF=gi:16741750 /FEA=FLmRNA /CNT=207 /TID=Mm.28492.1 /TIER=FL+Stack /STK=110 /UG=Mm.28492 /DEF=Mus musculus, clone MGC:6816 IMAGE:2648797, mRNA, complete cds. /PROD=Unknown (protein for MGC:6816) /FL=gb:BC016669.1	860.1333	288.7083	484.16666	115.139244	2.0950768	2.0	0.17115806	0.27162042	0.56	1.78
gb:BC002055.1 /DB_XREF=gi:12805192 /FEA=FLmRNA /CNT=194 /TID=Mm.1508.1 /TIER=FL+Stack /STK=73 /UG=Mm.1508 /LL=12257 /UG_GENE=Bzrp /DEF=Mus musculus, Similar to benzodiazepine receptor, peripheral, clone MGC:6086 IMAGE:3493196, mRNA, complete cds. /PROD=Similar to benzodiazepine receptor, peripheral /FL=gb:NM_009775.1 gb:L17306.1 gb:BC002055.1 gb:D21207.1	1181.9333	402.33136	650.36664	178.55083	2.091686	2.0	0.17157866	0.27110913	0.55	1.82
gb:NM_009281.2 /DB_XREF=gi:7949140 /GEN=Zfp143 /FEA=FLmRNA /CNT=51 /TID=Mm.10815.1 /TIER=FL+Stack /STK=29 /UG=Mm.10815 /LL=20841 /DEF=Mus musculus zinc finger protein 143 (Zfp143), mRNA. /PROD=zinc finger protein 143 /FL=gb:AF011758.2 gb:NM_009281.2	352.66666	124.71	200.73334	18.59068	2.087083	2.0	0.17215186	0.27084237	0.57	1.76
gb:NM_008761.2 /DB_XREF=gi:11612666 /GEN=Fxyd5 /FEA=FLmRNA /CNT=58 /TID=Mm.1870.1 /TIER=FL+Stack /STK=26 /UG=Mm.1870 /LL=18301 /DEF=Mus musculus FXYD domain-containing ion transport regulator 5 (Fxyd5), mRNA. /PROD=related to ion channel /FL=gb:U72680.1 gb:NM_008761.2	1579.3999	339.63223	955.8667	502.16968	1.7814642	3.0	0.17286329	0.27079442	0.61	1.65
gb:NM_008940.1 /DB_XREF=gi:6679486 /GEN=Klk8 /FEA=FLmRNA /CNT=11 /TID=Mm.5193.2 /TIER=FL /STK=7 /UG=Mm.5193 /LL=16624 /DEF=Mus musculus kallikrein 8 (Klk8), mRNA. /PROD=protease, serine, 19 (neuropsin) /FL=gb:D30785.1 gb:NM_008940.1	514.4666	147.93588	316.46667	73.88783	2.0739162	2.0	0.173806	0.27110764	0.62	1.63
gb:NM_008592.1 /DB_XREF=gi:6679834 /GEN=Foxc1 /FEA=FLmRNA /CNT=61 /TID=Mm.12949.1 /TIER=FL+Stack /STK=9 /UG=Mm.12949 /LL=17300 /DEF=Mus musculus forkhead box C1 (Foxc1), mRNA. /PROD=forkhead box C1 /FL=gb:AF045017.1 gb:NM_008592.1	284.06668	94.30697	169.09999	61.060375	1.7724156	3.0	0.17444223	0.27094218	0.6	1.68
gb:NM_023202.1 /DB_XREF=gi:12963570 /GEN=Ndufa7 /FEA=FLmRNA /CNT=13 /TID=Mm.29513.1 /TIER=FL /STK=0 /UG=Mm.29513 /LL=66416 /DEF=Mus musculus NADH dehydrogenase (ubiquinone) 1 alpha subcomplex, 7 (14.5kD, B14.5a) (Ndufa7), mRNA. /PROD=NADH dehydrogenase (ubiquinone) 1 alphasubcomplex, 7 (14.5kD, B14.5a) /FL=gb:NM_023202.1	12473.101	4055.233	7557.9	2600.8333	1.7671416	3.0	0.17537016	0.27122927	0.61	1.65
gb:NM_007472.1 /DB_XREF=gi:6680709 /GEN=Aqp1 /FEA=FLmRNA /CNT=262 /TID=Mm.18625.1 /TIER=FL+Stack /STK=113 /UG=Mm.18625 /LL=11826 /DEF=Mus musculus aquaporin 1 (Aqp1), mRNA. /PROD=aquaporin 1 /FL=gb:NM_007472.1 gb:BC007125.1 gb:L02914.1	9189.899	2563.1602	5969.3003	1845.5371	1.7661334	3.0	0.17554818	0.270359	0.65	1.54
gb:NM_007632.1 /DB_XREF=gi:6724312 /GEN=Ccnd3 /FEA=FLmRNA /CNT=286 /TID=Mm.7417.1 /TIER=FL+Stack /STK=172 /UG=Mm.7417 /LL=12445 /DEF=Mus musculus cyclin D3 (Ccnd3), mRNA. /PROD=cyclin D3 /FL=gb:BC004076.1 gb:NM_007632.1 gb:M86183.1 gb:BC005605.1	1193.2001	406.31543	681.76666	146.32567	2.051192	2.0	0.17671187	0.27100772	0.57	1.75
gb:AK014262.1 /DB_XREF=gi:12851992 /FEA=mRNA /CNT=54 /TID=Mm.45020.1 /TIER=Stack /STK=41 /UG=Mm.45020 /LL=73224 /UG_GENE=3110080A02Rik /UG_TITLE=RIKEN cDNA 3110080A02 gene /DEF=Mus musculus 13 days embryo head cDNA, RIKEN full-length enriched library, clone:3110080A02:unclassifiable transcript, full insert sequence.	483.83334	61.59841	314.56668	129.55139	2.0437663	2.0	0.17767572	0.27134576	0.65	1.54
gb:NM_080837.1 /DB_XREF=gi:18250287 /GEN=Il25 /FEA=FLmRNA /CNT=150 /TID=Mm.29925.1 /TIER=FL+Stack /STK=73 /UG=Mm.29925 /LL=28106 /DEF=Mus musculus interleukin 25 (Il25), mRNA. /PROD=interleukin 25 /FL=gb:AY038184.1 gb:NM_080837.1	531.9666	172.19682	323.43335	40.16644	2.0427072	2.0	0.17781377	0.2704251	0.61	1.64
gb:NM_025569.1 /DB_XREF=gi:13385009 /GEN=Mgst3 /FEA=FLmRNA /CNT=216 /TID=Mm.29823.1 /TIER=FL+Stack /STK=98 /UG=Mm.29823 /LL=66447 /DEF=Mus musculus microsomal glutathione S-transferase 3 (Mgst3), mRNA. /PROD=microsomal glutathione S-transferase 3 /FL=gb:NM_025569.1	5083.5	1483.3075	3071.2334	1323.4027	1.7533147	3.0	0.17782989	0.26932743	0.6	1.66
gb:NM_007932.1 /DB_XREF=gi:6679648 /GEN=Eng /FEA=FLmRNA /CNT=113 /TID=Mm.4851.1 /TIER=FL+Stack /STK=50 /UG=Mm.4851 /LL=13805 /DEF=Mus musculus endoglin (Eng), mRNA. /PROD=endoglin /FL=gb:NM_007932.1	2786.6335	1294.6776	1264.8334	141.4751	2.0238533	2.0	0.18029575	0.27193367	0.45	2.2
gb:NM_008898.1 /DB_XREF=gi:6679420 /GEN=Por /FEA=FLmRNA /CNT=179 /TID=Mm.3863.1 /TIER=FL+Stack /STK=63 /UG=Mm.3863 /LL=18984 /DEF=Mus musculus P450 (cytochrome) oxidoreductase (Por), mRNA. /PROD=P450 (cytochrome) oxidoreductase /FL=gb:D17571.1 gb:NM_008898.1	721.4001	186.83733	446.43335	200.67395	1.7369798	3.0	0.18078685	0.27155226	0.62	1.62
gb:NM_008322.1 /DB_XREF=gi:6680342 /GEN=Idh2 /FEA=FLmRNA /CNT=70 /TID=Mm.2966.1 /TIER=FL /STK=2 /UG=Mm.2966 /LL=15927 /DEF=Mus musculus isocitrate dehydrogenase 2 (NADP+), mitochondrial (Idh2), mRNA. /PROD=isocitrate dehydrogenase 2 (NADP+),mitochondrial /FL=gb:NM_008322.1 gb:U51167.1	11169.633	2583.2095	7453.2993	2658.36	1.736538	3.0	0.1808676	0.27056015	0.67	1.5
gb:NM_134093.1 /DB_XREF=gi:19527321 /GEN=AI593524 /FEA=FLmRNA /CNT=55 /TID=Mm.201624.1 /TIER=FL+Stack /STK=17 /UG=Mm.201624 /LL=105872 /DEF=Mus musculus expressed sequence AI593524 (AI593524), mRNA. /PROD=cervical cancer receptor /FL=gb:AF287293.1 gb:NM_134093.1	236.06667	73.37986	148.2	17.3156	2.0185578	2.0	0.18100129	0.269655	0.63	1.59
gb:NM_133950.1 /DB_XREF=gi:19527169 /GEN=8030486F04Rik /FEA=FLmRNA /CNT=142 /TID=Mm.25045.1 /TIER=FL+Stack /STK=40 /UG=Mm.25045 /LL=68137 /DEF=Mus musculus RIKEN cDNA 8030486F04 gene (8030486F04Rik), mRNA. /PROD=putative KDEL receptor /FL=gb:BC011370.1 gb:NM_133950.1	1906.1666	557.6326	1163.2	488.95154	1.7351526	3.0	0.1811211	0.26873657	0.61	1.64
gb:BC006820.1 /DB_XREF=gi:13905071 /FEA=FLmRNA /CNT=69 /TID=Mm.41261.1 /TIER=FL+Stack /STK=43 /UG=Mm.41261 /LL=75687 /UG_GENE=2310066E14Rik /DEF=Mus musculus, RIKEN cDNA 2310066E14 gene, clone MGC:11838 IMAGE:3596826, mRNA, complete cds. /PROD=RIKEN cDNA 2310066E14 gene /FL=gb:BC006820.1	628.8	52.460636	411.63333	179.04707	2.016053	2.0	0.18133631	0.26796663	0.65	1.53
gb:NM_024166.2 /DB_XREF=gi:18079333 /GEN=Etohi6 /FEA=FLmRNA /CNT=20 /TID=Mm.30133.1 /TIER=FL /STK=0 /UG=Mm.30133 /LL=14004 /DEF=Mus musculus ethanol induced 6 (Etohi6), mRNA. /PROD=ethanol induced 6 /FL=gb:NM_024166.2	14814.734	4323.9595	9379.066	1765.9126	2.0157433	2.0	0.1813778	0.26694715	0.63	1.58
gb:BC010564.1 /DB_XREF=gi:14714826 /FEA=FLmRNA /CNT=83 /TID=Mm.219659.1 /TIER=FL+Stack /STK=24 /UG=Mm.219659 /LL=15267 /UG_GENE=Hist2 /DEF=Mus musculus, H2A histone family, member O, clone MGC:5956 IMAGE:3582122, mRNA, complete cds. /PROD=H2A histone family, member O /FL=gb:NM_013549.1 gb:BC010564.1	869.1333	246.55516	570.23334	169.00517	1.7319441	3.0	0.18170972	0.26636162	0.66	1.52
gb:AK017622.1 /DB_XREF=gi:12856958 /FEA=mRNA /CNT=1 /TID=Mm.24997.2 /TIER=ConsEnd /STK=0 /UG=Mm.24997 /LL=107817 /UG_GENE=Ptdsr /UG_TITLE=phosphatidylserine receptor /DEF=Mus musculus 8 days embryo whole body cDNA, RIKEN full-length enriched library, clone:5730436I23:APOPTOTIC CELL CLEARANCE RECEPTOR PTDSERR (UNKNOWN) (PROTEIN FOR MGC:8280), full insert sequence.	370.9	118.138725	236.09999	67.12652	1.7183143	3.0	0.1842347	0.26898265	0.64	1.57
gb:NM_008173.1 /DB_XREF=gi:6680102 /GEN=Nr3c1 /FEA=FLmRNA /CNT=182 /TID=Mm.4918.1 /TIER=FL /STK=1 /UG=Mm.4918 /LL=14815 /DEF=Mus musculus nuclear receptor subfamily 3, group C, member 1 (Nr3c1), mRNA. /PROD=glucocorticoid receptor 1 /FL=gb:NM_008173.1	321.3	118.45721	176.36668	85.73624	1.7167078	3.0	0.18453492	0.2683476	0.55	1.82

gb:AF004833.1 /DB_XREF=gi:2897076 /GEN=TFPI /FEA=FLmRNA /CNT=10 /TID=Mm.3601.1 /TIER=FL /STK=1 /UG=Mm.3601 /LL=21788 /DEF=Mus musculus tissue factor pathway inhibitor (TFPI) mRNA, complete cds. /PROD=tissue factor pathway inhibitor /FL=gb:AF004833.1	1606.7666	382.47437	1031.9667	440.50238	1.7065847	3.0	0.18643968	0.27004158	0.64	1.56
gb:NM_133964.1 /DB_XREF=gi:19527181 /GEN=1110033C18Rik /FEA=FLmRNA /CNT=109 /TID=Mm.4022.1 /TIER=FL+Stack /STK=48 /UG=Mm.4022 /LL=102115 /DEF=Mus musculus RIKEN cDNA 1110033C18 gene (1110033C18Rik), mRNA. /PROD=RIKEN cDNA 1110033C18 /FL=gb:BC002295.1 gb:NM_133964.1	448.66666	162.7051	255.36665	47.330883	1.975841	2.0	0.18683113	0.26953897	0.57	1.76
gb:BC016492.1 /DB_XREF=gi:16741319 /FEA=FLmRNA /CNT=172 /TID=Mm.18843.1 /TIER=FL+Stack /STK=43 /UG=Mm.18843 /LL=21817 /UG_GENE=Tgm2 /DEF=Mus musculus, transglutaminase 2, C polypeptide, clone MGC:6152 IMAGE:3256943, mRNA, complete cds. /PROD=transglutaminase 2, C polypeptide /FL=gb:NM_009373.1 gb:AF076928.1 gb:BC016492.1	6511.0	2017.0101	4233.3	1157.5739	1.6963931	3.0	0.18837993	0.27070343	0.65	1.54
gb:NM_013868.1 /DB_XREF=gi:7305170 /GEN=Hsp25-2 /FEA=FLmRNA /CNT=15 /TID=Mm.46181.1 /TIER=FL /STK=2 /UG=Mm.46181 /LL=29818 /DEF=Mus musculus heat shock protein 25 kDa 2 (cardiovascular) (Hsp25-2), mRNA. /PROD=heat shock protein 25 kDa 2 (cardiovascular) /FL=gb:AF155909.1 gb:NM_013868.1	2128.9001	1409.7396	512.4666	235.13553	1.9589396	2.0	0.18920761	0.27082658	0.24	4.15
gb:AK010963.1 /DB_XREF=gi:12846763 /FEA=mRNA /CNT=80 /TID=Mm.87628.1 /TIER=Stack /STK=48 /UG=Mm.87628 /LL=70312 /UG_GENE=2510012J08Rik /UG_TITLE=RIKEN cDNA 2510012J08 gene /DEF=Mus musculus 13 days embryo liver cDNA, RIKEN full-length enriched library, clone:2510012J08:homolog to NY-REN-24 ANTIGEN (FRAGMENT), full insert sequence.	780.8667	238.60535	497.13333	77.86105	1.9580262	2.0	0.1893372	0.2699534	0.64	1.57
gb:NM_018860.1 /DB_XREF=gi:9256635 /GEN=Rpl41 /FEA=FLmRNA /CNT=37 /TID=Mm.13859.1 /TIER=FL+Stack /STK=25 /UG=Mm.13859 /LL=67945 /DEF=Mus musculus ribosomal protein L41 (Rpl41), mRNA. /PROD=ribosomal protein L41 /FL=gb:NM_018860.1 gb:U93862.1	18386.701	5389.7344	11991.466	1807.8418	1.9484895	2.0	0.19069731	0.27083468	0.65	1.53
gb:BC020153.1 /DB_XREF=gi:18043303 /FEA=FLmRNA /CNT=98 /TID=Mm.21473.1 /TIER=FL+Stack /STK=18 /UG=Mm.21473 /LL=66227 /UG_GENE=1190017B19Rik /DEF=Mus musculus, RIKEN cDNA 1190017B19 gene, clone MGC:28240 IMAGE:3992930, mRNA, complete cds. /PROD=RIKEN cDNA 1190017B19 gene /FL=gb:NM_023175.1 gb:AF284573.1 gb:BC020153.1	1109.8334	308.6267	713.69995	266.0716	1.6837974	3.0	0.19080964	0.26994386	0.64	1.56
gb:NM_011793.1 /DB_XREF=gi:6753177 /GEN=Bcrp1-pending /FEA=FLmRNA /CNT=16 /TID=Mm.7508.1 /TIER=FL /STK=2 /UG=Mm.7508 /LL=23825 /DEF=Mus musculus breakpoint cluster region protein 1 (Bcrp1-pending), mRNA. /PROD=breakpoint cluster region protein 1 /FL=gb:NM_011793.1 gb:AB025349.1	1092.2001	301.97952	725.06665	226.80002	1.6837565	3.0	0.19081758	0.2689128	0.66	1.51
gb:NM_011778.1 /DB_XREF=gi:6753493 /GEN=Coro1b /FEA=FLmRNA /CNT=209 /TID=Mm.28652.1 /TIER=FL+Stack /STK=87 /UG=Mm.28652 /LL=23789 /DEF=Mus musculus coronin, actin binding protein 1B (Coro1b), mRNA. /PROD=coronin, actin binding protein 1B /FL=gb:NM_011778.1 gb:AF143956.1	970.4333	345.4004	573.5667	79.223885	1.9397628	2.0	0.19195344	0.26947308	0.59	1.69
gb:NM_026353.1 /DB_XREF=gi:13385855 /GEN=4930570C03Rik /FEA=FLmRNA /CNT=27 /TID=Mm.28955.1 /TIER=FL /STK=1 /UG=Mm.28955 /LL=67739 /DEF=Mus musculus RIKEN cDNA 4930570C03 gene (4930570C03Rik), mRNA. /PROD=RIKEN cDNA 4930570C03 /FL=gb:NM_026353.1 gb:BC022913.1	1148.1333	399.32288	666.1333	160.61934	1.9396348	2.0	0.19197194	0.2684665	0.58	1.72
gb:NM_011295.1 /DB_XREF=gi:6755365 /GEN=Rps12 /FEA=FLmRNA /CNT=156 /TID=Mm.21289.1 /TIER=FL+Stack /STK=88 /UG=Mm.21289 /LL=20042 /DEF=Mus musculus ribosomal protein S12 (Rps12), mRNA. /PROD=ribosomal protein S12 /FL=gb:BC002079.1 gb:BC018362.1 gb:NM_011295.1	14910.867	4813.6387	9274.433	1604.0054	1.9240993	2.0	0.19423601	0.27059597	0.62	1.61
gb:NM_007506.1 /DB_XREF=gi:6680749 /GEN=Atp5g1 /FEA=FLmRNA /CNT=253 /TID=Mm.258.1 /TIER=FL+Stack /STK=143 /UG=Mm.258 /LL=11951 /DEF=Mus musculus ATP synthase, H+ transporting, mitochondrial F0 complex, subunit c (subunit 9), isoform 1 (Atp5g1), mRNA. /PROD=ATP synthase, H+ transporting, mitochondrial F0complex, subunit c (subunit 9), isoform 1 /FL=gb:NM_007506.1 gb:L19737.1 gb:BC003854.1	31173.766	9255.258	20473.766	2719.352	1.9212122	2.0	0.1946607	0.27015647	0.66	1.52
gb:NM_007574.1 /DB_XREF=gi:6671651 /GEN=C1qc /FEA=FLmRNA /CNT=46 /TID=Mm.3453.1 /TIER=FL+Stack /STK=11 /UG=Mm.3453 /LL=12262 /DEF=Mus musculus complement component 1, q subcomponent, c polypeptide (C1qc), mRNA. /PROD=complement component 1, q subcomponent, cpolypeptide /FL=gb:NM_007574.1	997.1667	317.45963	617.10004	238.51569	1.6578506	3.0	0.19592747	0.27088457	0.62	1.62
gb:BC022157.1 /DB_XREF=gi:18381057 /FEA=mRNA /CNT=32 /TID=Mm.21597.1 /TIER=ConsEnd /STK=0 /UG=Mm.21597 /LL=106522 /UG_GENE=AI115348 /DEF=Mus musculus, clone IMAGE:5134400, mRNA, partial cds. /PROD=Unknown (protein for IMAGE:5134400)	885.1667	329.22888	505.46667	100.522125	1.9105076	2.0	0.1962462	0.2703014	0.57	1.75
gb:BC019836.1 /DB_XREF=gi:18043722 /FEA=FLmRNA /CNT=420 /TID=Mm.22248.2 /TIER=FL+Stack /STK=125 /UG=Mm.22248 /LL=16010 /UG_GENE=Igfbp4 /DEF=Mus musculus, insulin-like growth factor binding protein 4, clone MGC:29917 IMAGE:5123738, mRNA, complete cds. /PROD=insulin-like growth factor binding protein 4 /FL=gb:BC019836.1	3464.2	1083.6925	2282.7666	594.74207	1.6553617	3.0	0.1964265	0.26953262	0.66	1.52
gb:AK017037.1 /DB_XREF=gi:12856093 /FEA=mRNA /CNT=3 /TID=Mm.80542.4 /TIER=ConsEnd /STK=2 /UG=Mm.80542 /LL=67582 /UG_GENE=4930433D19Rik /UG_TITLE=RIKEN cDNA 4930433D19 gene /DEF=Mus musculus adult male testis cDNA, RIKEN full-length enriched library, clone:4933433F13:Mitochondrial carrier proteins containing protein, full insert sequence.	357.06665	118.251274	211.0	60.100258	1.9072695	2.0	0.19672921	0.26893696	0.59	1.69
gb:NM_008847.1 /DB_XREF=gi:6679330 /GEN=Pip5k1b /FEA=FLmRNA /CNT=99 /TID=Mm.87521.1 /TIER=FL+Stack /STK=29 /UG=Mm.87521 /LL=18720 /DEF=Mus musculus phosphatidylinositol-4-phosphate 5-kinase, type 1 beta (Pip5k1b), mRNA. /PROD=phosphatidylinositol-4-phosphate 5-kinase, type1 beta /FL=gb:NM_008847.1 gb:D86177.1	665.6667	119.53984	443.2333	200.45299	1.6507344	3.0	0.19735807	0.26878992	0.67	1.5
gb:NM_017402.1 /DB_XREF=gi:8393893 /GEN=Arhgef7 /FEA=FLmRNA /CNT=63 /TID=Mm.3439.1 /TIER=FL+Stack /STK=24 /UG=Mm.3439 /LL=54126 /DEF=Mus musculus Rho guanine nucleotide exchange factor (GEF7) (Arhgef7), mRNA. /PROD=Rho guanine nucleotide exchange factor (GEF7) /FL=gb:NM_017402.1 gb:U96634.1	747.76666	208.25203	491.36667	105.98007	1.9005513	2.0	0.19773641	0.26830405	0.66	1.52
gb:BC028550.1 /DB_XREF=gi:20306503 /FEA=mRNA /CNT=8 /TID=Mm.144300.1 /TIER=ConsEnd /STK=0 /UG=Mm.144300 /DEF=Mus musculus, H4 histone family, member A, clone MGC:41466 IMAGE:1398106, mRNA, complete cds. /PROD=H4 histone family, member A	1164.7999	442.33972	648.8333	169.54597	1.8865175	2.0	0.19986269	0.27018476	0.56	1.8
gb:NM_010833.1 /DB_XREF=gi:6754749 /GEN=Msn /FEA=FLmRNA /CNT=271 /TID=Mm.28687.1 /TIER=FL /STK=1 /UG=Mm.28687 /LL=17698 /DEF=Mus musculus moesin (Msn), mRNA. /PROD=moesin /FL=gb:NM_010833.1	977.89996	476.53815	421.33334	187.60748	1.8823091	2.0	0.20050626	0.27005455	0.43	2.32
gb:NM_010286.1 /DB_XREF=gi:6753985 /GEN=Gilz /FEA=FLmRNA /CNT=5 /TID=Mm.22216.1 /TIER=FL /STK=3 /UG=Mm.22216 /LL=14605 /DEF=Mus musculus glucocorticoid-induced leucine zipper (Gilz), mRNA. /PROD=glucocorticoid-induced leucine zipper /FL=gb:AF024519.1 gb:NM_010286.1	1798.2334	487.1003	1107.8667	554.0929	1.620791	3.0	0.20350805	0.27308986	0.62	1.62
gb:NM_008453.1 /DB_XREF=gi:6680581 /GEN=Klf3 /FEA=FLmRNA /CNT=4 /TID=Mm.28787.1 /TIER=FL /STK=2 /UG=Mm.28787 /LL=16599 /DEF=Mus musculus Kruppel-like factor 3 (basic) (Klf3), mRNA. /PROD=Kruppel-like factor 3 (basic) /FL=gb:U36340.1 gb:NM_008453.1	432.2333	66.69755	247.90001	159.92696	1.8425595	2.0	0.20672318	0.27638814	0.57	1.74
gb:NM_008738.1 /DB_XREF=gi:6679135 /GEN=Nrtn /FEA=FLmRNA /CNT=31 /TID=Mm.8074.1 /TIER=FL+Stack /STK=16 /UG=Mm.8074 /LL=18188 /DEF=Mus musculus neurturin (Nrtn), mRNA. /PROD=neurturin /FL=gb:NM_008738.1 gb:U78109.1	1260.7999	561.4893	664.8	20.29703	1.8373072	2.0	0.20756371	0.27649912	0.53	1.9
gb:NM_011072.1 /DB_XREF=gi:6755039 /GEN=Pfn1 /FEA=FLmRNA /CNT=168 /TID=Mm.2647.1 /TIER=FL+Stack /STK=26 /UG=Mm.2647 /LL=18643 /DEF=Mus musculus profilin 1 (Pfn1), mRNA. /PROD=profilin 1 /FL=gb:BC002080.1 gb:NM_011072.1	1880.2666	486.36215	1148.6666	624.1759	1.6013907	3.0	0.20760737	0.27555162	0.61	1.64
gb:NM_025650.1 /DB_XREF=gi:13385111 /GEN=0710008D09Rik /FEA=FLmRNA /CNT=200 /TID=Mm.43162.1 /TIER=FL+Stack /STK=100 /UG=Mm.43162 /LL=66594 /DEF=Mus musculus RIKEN cDNA 0710008D09 gene (0710008D09Rik), mRNA. /PROD=RIKEN cDNA 0710008D09 /FL=gb:NM_025650.1	17802.533	6000.5195	10966.7	2426.666	1.8292425	2.0	0.20886318	0.276214	0.62	1.62
gb:NM_023119.1 /DB_XREF=gi:12963490 /GEN=Eno1 /FEA=FLmRNA /CNT=270 /TID=Mm.90587.1 /TIER=FL+Stack /STK=14 /UG=Mm.90587 /LL=13806 /DEF=Mus musculus enolase 1, alpha non-neuron (Eno1), mRNA. /PROD=enolase 1, alpha non-neuron /FL=gb:BC024644.1 gb:BC003891.1 gb:BC010685.1 gb:NM_023119.1	6644.033	2078.6033	4233.8335	1600.5298	1.5912825	3.0	0.20977983	0.27642468	0.64	1.57
gb:NM_007751.1 /DB_XREF=gi:6680994 /GEN=Cox8b /FEA=FLmRNA /CNT=27 /TID=Mm.3841.1 /TIER=FL+Stack /STK=18 /UG=Mm.3841 /LL=12869 /DEF=Mus musculus cytochrome c oxidase, subunit VIIIb (Cox8b), mRNA. /PROD=cytochrome c oxidase, subunit VIIIb /FL=gb:U15541.1 gb:NM_007751.1	30768.635	9435.1045	20126.934	3702.992	1.8185111	2.0	0.21060908	0.27651912	0.65	1.55
gb:NM_007899.1 /DB_XREF=gi:6681256 /GEN=Ecm1 /FEA=FLmRNA /CNT=126 /TID=Mm.3433.1 /TIER=FL+Stack /STK=53 /UG=Mm.3433 /LL=13601 /DEF=Mus musculus extracellular matrix protein 1 (Ecm1), mRNA. /PROD=extracellular matrix protein 1 /FL=gb:NM_007899.1 gb:L33416.1	2801.5664	1210.8658	1481.3999	358.34644	1.8107661	2.0	0.21188116	0.2771922	0.53	1.89
gb:NM_133240.1 /DB_XREF=gi:18875407 /GEN=Pte1 /FEA=FLmRNA /CNT=68 /TID=Mm.18641.1 /TIER=FL+Stack /STK=45 /UG=Mm.18641 /LL=170789 /DEF=Mus musculus peroxisomal acyl-CoA thioesterase 1 (Pte1), mRNA. /PROD=peroxisomal acyl-CoA thioesterase 1 /FL=gb:BC005792.1 gb:NM_133240.1 gb:AF441166.1	465.36667	222.47429	224.0	63.115204	1.807794	2.0	0.212372	0.2768421	0.48	2.08
gb:NM_053122.1 /DB_XREF=gi:16716420 /GEN=Immp2l-pending /FEA=FLmRNA /CNT=25 /TID=Mm.41069.1 /TIER=FL+Stack /STK=9 /UG=Mm.41069 /LL=93757 /DEF=Mus musculus inner mitochondrial membrane peptidase 2-like (S. cerevisiae) (Immp2l-pending), mRNA. /PROD=inner mitochondrial membrane peptidase 2-like(S. cerevisiae) /FL=gb:AF359564.1 gb:NM_053122.1	1377.0668	475.85724	873.3333	85.10619	1.8048774	2.0	0.21285515	0.27648443	0.63	1.58
gb:AK018713.1 /DB_XREF=gi:12858561 /GEN=Cyba /FEA=mRNA /CNT=1 /TID=Mm.448.2 /TIER=ConsEnd /STK=0 /UG=Mm.448 /LL=13057 /UG_TITLE=cytochrome b-245, alpha polypeptide /DEF=Mus musculus adult male kidney cDNA, RIKEN full-length enriched library, clone:0610007F11:cytochrome b-245, alpha polypeptide, full insert sequence.	1930.9332	586.1127	1270.2334	430.65775	1.5734007	3.0	0.21368538	0.2765786	0.66	1.52
gb:BC019745.1 /DB_XREF=gi:18043950 /FEA=FLmRNA /CNT=137 /TID=Mm.26702.1 /TIER=FL+Stack /STK=18 /UG=Mm.26702 /LL=83921 /UG_GENE=Tmem2 /DEF=Mus musculus, clone MGC:30634 IMAGE:3675225, mRNA, complete cds. /PROD=Unknown (protein for MGC:30634) /FL=gb:BC019745.1	492.83334	196.5322	273.26666	78.078445	1.798335	2.0	0.21394421	0.2759351	0.55	1.8
gb:BC019407.1 /DB_XREF=gi:18043858 /FEA=FLmRNA /CNT=30 /TID=Mm.29321.1 /TIER=FL /STK=4 /UG=Mm.29321 /LL=77090 /UG_GENE=9430098E02Rik /DEF=Mus musculus, Similar to RIKEN cDNA 9430098E02 gene, clone MGC:30323 IMAGE:5151818, mRNA, complete cds. /PROD=Similar to RIKEN cDNA 9430098E02 gene /FL=gb:BC019407.1	534.0	172.33046	330.2	145.73456	1.5640509	3.0	0.21575966	0.27729675	0.62	1.62
gb:NM_019684.1 /DB_XREF=gi:9790110 /GEN=Stk23 /FEA=FLmRNA /CNT=28 /TID=Mm.41679.1 /TIER=FL+Stack /STK=18 /UG=Mm.41679 /LL=56504 /DEF=Mus musculus serinethreonine kinase 23 (Stk23), mRNA. /PROD=serinethreonine kinase 23 /FL=gb:NM_019684.1 gb:AF043288.1	562.9333	168.13858	369.09998	138.33885	1.5419214	3.0	0.22075884	0.28272623	0.66	1.53
gb:BC026638.1 /DB_XREF=gi:20072373 /FEA=FLmRNA /CNT=59 /TID=Mm.86759.1 /TIER=FL+Stack /STK=18 /UG=Mm.86759 /DEF=Mus musculus, Similar to mannosyl (alpha-1,3-)-glycoprotein beta-1,4-N-acetylglucosaminyltransferase, isoenzyme B, clone MGC:37502 IMAGE:4984907, mRNA, complete cds. /PROD=Similar to mannosyl (alpha-1,3-)-glycoproteinbeta-1,4-N-acetylglucosaminyltransferase, isoenzyme B /FL=gb:BC026638.1	1303.5	507.88135	790.4333	9.6656	1.7494179	2.0	0.22232446	0.28373575	0.61	1.65
gb:NM_025427.1 /DB_XREF=gi:13384823 /GEN=1190002H23Rik /FEA=FLmRNA /CNT=94 /TID=Mm.29811.1 /TIER=FL+Stack /STK=31 /UG=Mm.29811 /LL=66214 /DEF=Mus musculus RIKEN cDNA 1190002H23 gene (1190002H23Rik), mRNA. /PROD=RIKEN cDNA 1190002H23 /FL=gb:AF276981.1 gb:NM_025427.1	866.4666	374.6637	459.36667	265.9359	1.534698	3.0	0.2224183	0.28286648	0.53	1.89
gb:NM_011335.1 /DB_XREF=gi:7549786 /GEN=Scya21a /FEA=FLmRNA /CNT=15 /TID=Mm.220853.1 /TIER=FL+Stack /STK=10 /UG=Mm.220853 /LL=20298 /DEF=Mus musculus small inducible cytokine A21a (serine) (Scya21a), mRNA. /PROD=small inducible cytokine A21a (serine) /FL=gb:NM_011335.1 gb:U88322.1	2090.3665	517.5319	1365.1	638.4589	1.5284649	3.0	0.22386134	0.28371316	0.65	1.53
gb:BC014728.1 /DB_XREF=gi:15928499 /FEA=FLmRNA /CNT=90 /TID=Mm.212927.1 /TIER=FL /STK=1 /UG=Mm.212927 /DEF=Mus musculus, Similar to hypothetical protein MGC3169, clone MGC:25675 IMAGE:4504294, mRNA, complete cds. /PROD=Similar to hypothetical protein MGC3169 /FL=gb:BC024788.1 gb:BC014728.1	1344.0667	777.81036	559.3	75.45601	1.7393757	2.0	0.22409798	0.28303033	0.42	2.4
gb:NM_023258.1 /DB_XREF=gi:12963604 /GEN=Asc-pending /FEA=FLmRNA /CNT=83 /TID=Mm.24163.1 /TIER=FL+Stack /STK=47 /UG=Mm.24163 /LL=66824 /DEF=Mus musculus apoptosis-associated speck-like protein containing a CARD (Asc-pending), mRNA. /PROD=apoptosis-associated speck-like proteincontaining a CARD /FL=gb:AB032249.1 gb:AF310104.1 gb:BC008252.1 gb:NM_023258.1	554.9333	198.16301	353.8667	29.345747	1.7384704	2.0	0.22425878	0.28225675	0.64	1.57
gb:NM_019822.1 /DB_XREF=gi:9789884 /GEN=Adrm1 /FEA=FLmRNA /CNT=68 /TID=Mm.181562.1 /TIER=FL+Stack /STK=11 /UG=Mm.181562 /LL=56436 /DEF=Mus musculus adhesion regulating molecule 1 (Adrm1), mRNA. /PROD=adhesion regulating molecule 1 /FL=gb:AF225959.1 gb:NM_019822.1 gb:BC008974.1	1396.5	490.32642	901.6667	86.00303	1.721688	2.0	0.2272671	0.2850601	0.65	1.55
gb:NM_008198.1 /DB_XREF=gi:6996918 /GEN=H2-Bf /FEA=FLmRNA /CNT=187 /TID=Mm.653.1 /TIER=FL+Stack /STK=48 /UG=Mm.653 /LL=14962 /DEF=Mus musculus histocompatibility 2, complement component factor B (H2-Bf), mRNA. /PROD=histocompatibility 2, complement componentfactor B /FL=gb:NM_008198.1 gb:BC005451.1 gb:M57890.1	387.16666	33.340126	248.46666	135.52997	1.7212474	2.0	0.2273468	0.28418347	0.64	1.56
gb:NM_008284.1 /DB_XREF=gi:6680270 /GEN=Hras1 /FEA=FLmRNA /CNT=1 /TID=Mm.212499.1 /TIER=FL /STK=1 /UG=Mm.212499 /LL=15461 /DEF=Mus musculus Harvey rat sarcoma virus oncogene (Hras1), mRNA. /PROD=Harvey rat sarcoma virus oncogene /FL=gb:NM_008284.1	837.60004	352.40677	478.6	81.31537	1.7192805	2.0	0.22770299	0.2836573	0.57	1.75
gb:BB206220 /DB_XREF=gi:8871173 /DB_XREF=BB206220 /CLONE=A430073H16 /FEA=EST /CNT=26 /TID=Mm.30018.2 /TIER=Stack /STK=26 /UG=Mm.30018 /UG_TITLE=Mus musculus, serine threonine kinase pim3, clone MGC:27707 IMAGE:4924687, mRNA, complete cds	4001.0332	1582.3501	2393.8	378.13297	1.7111088	2.0	0.22919065	0.2845394	0.6	1.67
gb:NM_009003.1 /DB_XREF=gi:6679594 /GEN=Rab4a /FEA=FLmRNA /CNT=64 /TID=Mm.9221.1 /TIER=FL+Stack /STK=25 /UG=Mm.9221 /LL=19341 /DEF=Mus musculus RAB4A, member RAS oncogene family (Rab4a), mRNA. /PROD=RAB4A, member RAS oncogene family /FL=gb:D86563.1 gb:NM_009003.1	372.7667	25.8206	248.5	123.12388	1.71091	2.0	0.229227	0.28361985	0.67	1.5
gb:AK019470.1 /DB_XREF=gi:12859695 /FEA=mRNA /CNT=81 /TID=Mm.40681.1 /TIER=Stack /STK=8 /UG=Mm.40681 /LL=78754 /UG_GENE=4631401E18Rik /UG_TITLE=RIKEN cDNA 4631401E18 gene /DEF=Mus musculus 0 day neonate skin cDNA, RIKEN full-length enriched library, clone:4631401E18:homolog to UDP-GALNAC:POLYPEPTIDE N-ACETYLGALACTOSAMINYLTRANSFERASE, full insert sequence.	696.39996	259.79987	426.3667	86.50446	1.7080796	2.0	0.22974534	0.28330085	0.61	1.63
gb:NM_007990.1 /DB_XREF=gi:6679752 /GEN=Fau /FEA=FLmRNA /CNT=140 /TID=Mm.4890.1 /TIER=FL+Stack /STK=44 /UG=Mm.4890 /LL=14109 /DEF=Mus musculus Finkel-Biskis-Reilly murine sarcoma virus (FBR-MuSV) ubiquitously expressed (fox derived) (Fau), mRNA. /PROD=Finkel-Biskis-Reilly murine sarcoma virus(FBR-MuSV) ubiquitously expressed (fox derived) /FL=gb:NM_007990.1 gb:AF147745.1 gb:D26610.1	13061.8	3870.341	8593.399	3409.3862	1.5005265	3.0	0.23045751	0.2832222	0.66	1.52
gb:BG244243 /DB_XREF=gi:12754058 /DB_XREF=602356434F1 /CLONE=IMAGE:4485056 /FEA=EST /CNT=39 /TID=Mm.44227.2 /TIER=Stack /STK=39 /UG=Mm.44227 /UG_TITLE=Mus musculus, clone MGC:37950 IMAGE:5132866, mRNA, complete cds	23382.932	10109.377	13031.466	6374.3315	1.500204	3.0	0.23053491	0.28236657	0.56	1.79
Mus musculus /REF=M12481 /DEF=Mouse cytoplasmic beta-actin mRNA /LEN=1109 (_5, _M, _3 represent transcript regions 5 prime, Middle, and 3 prime respectively)	11262.766	4487.95	6811.467	2567.0461	1.4912015	3.0	0.23270646	0.28407308	0.6	1.65
gb:AK013376.1 /DB_XREF=gi:12850695 /GEN=Aplp2 /FEA=mRNA /CNT=1 /TID=Mm.19133.3 /TIER=ConsEnd /STK=0 /UG=Mm.19133 /LL=11804 /UG_TITLE=amyloid beta (A4) precursor-like protein 2 /DEF=Mus musculus 10, 11 days embryo whole body cDNA, RIKEN full-length enriched library, clone:2810462O13:amyloid beta (A4) precursor-like protein 2, full insert sequence.	303.0	123.79224	173.83333	47.059147	1.6893035	2.0	0.23322287	0.2837545	0.57	1.74
gb:AK008824.1 /DB_XREF=gi:12843252 /FEA=mRNA /CNT=3 /TID=Mm.158805.1 /TIER=ConsEnd /STK=1 /UG=Mm.158805 /LL=72279 /UG_GENE=2210404C19Rik /UG_TITLE=RIKEN cDNA 2210404C19 gene /DEF=Mus musculus adult male stomach cDNA, RIKEN full-length enriched library, clone:2210404C19:related to GB|AAF24540.1, full insert sequence.	1792.4332	644.0837	1104.4	297.82657	1.6793883	2.0	0.23508698	0.28507224	0.62	1.62

gb:AJ293897.1 /DB_XREF=gi:13397924 /FEA=mRNA /CNT=57 /TID=Mm.90071.1 /TIER=Stack /STK=18 /UG=Mm.90071 /LL=69961 /UG_GENE=2810432D09Rik /DEF=Mus musculus mRNA for hypothetical protein (ORF1), related to clone Telethon(Italy_B41)_Strait02713_FL143. /PROD=hypothetical protein	786.4666	281.34113	482.2333	139.6	1.6777941	2.0	0.23538849	0.2844927	0.61	1.63
gb:NM_021549.1 /DB_XREF=gi:10946987 /GEN=Pnkp /FEA=FLmRNA /CNT=118 /TID=Mm.29545.1 /TIER=FL+Stack /STK=94 /UG=Mm.29545 /LL=59047 /DEF=Mus musculus polynucleotide kinase 3- phosphatase (Pnkp), mRNA. /PROD=polynucleotide kinase 3-phosphatase /FL=gb:AF129451.1 gb:NM_021549.1	358.53333	134.20358	225.66667	82.84723	1.4591552	3.0	0.24062012	0.2898559	0.63	1.59
gb:BC024112.1 /DB_XREF=gi:18848232 /FEA=FLmRNA /CNT=473 /TID=Mm.218862.1 /TIER=FL+Stack /STK=69 /UG=Mm.218862 /DEF=Mus musculus, clone MGC:36398 IMAGE:5102118, mRNA, complete cds. /PROD=Unknown (protein for MGC:36398) /FL=gb:BC026577.1 gb:BC024112.1 gb:BC022113.1 gb:BC016435.1	412.36667	169.73123	249.23334	26.214762	1.6452143	2.0	0.24166198	0.29015335	0.6	1.65
gb:BC004732.1 /DB_XREF=gi:13435746 /FEA=FLmRNA /CNT=285 /TID=Mm.30016.2 /TIER=FL+Stack /STK=79 /UG=Mm.30016 /LL=97755 /UG_GENE=C87222 /DEF=Mus musculus, clone MGC:7904 IMAGE:3583026, mRNA, complete cds. /PROD=Unknown (protein for MGC:7904) /FL=gb:BC004732.1	2339.9668	1092.4368	1295.1666	129.02925	1.6450883	2.0	0.24168666	0.28923157	0.55	1.81
gb:NM_011777.1 /DB_XREF=gi:6756084 /GEN=Zyx /FEA=FLmRNA /CNT=160 /TID=Mm.4895.1 /TIER=FL+Stack /STK=51 /UG=Mm.4895 /LL=22793 /DEF=Mus musculus zyxin (Zyx), mRNA. /PROD=zyxin /FL=gb:NM_011777.1	1814.6	746.12103	1055.8	285.90887	1.6448554	2.0	0.2417323	0.28834084	0.58	1.72
gb:NM_009720.1 /DB_XREF=gi:6753135 /GEN=Atox1 /FEA=FLmRNA /CNT=114 /TID=Mm.9703.1 /TIER=FL+Stack /STK=96 /UG=Mm.9703 /LL=11927 /DEF=Mus musculus ATX1 (antioxidant protein 1) homolog 1 (yeast) (Atox1), mRNA. /PROD=ATX1 (antioxidant protein 1, yeast) homolog 1 /FL=gb:AF004591.1 gb:NM_009720.1	1247.1666	382.0489	829.26666	321.92905	1.4488084	3.0	0.24323751	0.2891912	0.66	1.5
gb:NM_010580.1 /DB_XREF=gi:6754379 /GEN=Itgb5 /FEA=FLmRNA /CNT=117 /TID=Mm.6424.1 /TIER=FL+Stack /STK=42 /UG=Mm.6424 /LL=16419 /DEF=Mus musculus integrin beta 5 (Itgb5), mRNA. /PROD=integrin beta 5 /FL=gb:NM_010580.1 gb:AF022110.1 gb:AF043257.1 gb:AF043256.1	3718.7666	1939.8148	1818.9335	550.6831	1.6318688	2.0	0.24429424	0.28950456	0.49	2.04
Mus musculus /REF=M12481 /DEF=Mouse cytoplasmic beta-actin mRNA /LEN=1109 (_5, _M, _3 represent transcript regions 5 prime, Middle, and 3 prime respectively)	7098.533	3205.232	3795.8333	1436.3824	1.6286589	2.0	0.24493289	0.28932202	0.53	1.87
gb:AK006418.1 /DB_XREF=gi:12839509 /FEA=mRNA /CNT=9 /TID=Mm.139700.2 /TIER=ConsEnd /STK=7 /UG=Mm.139700 /LL=76171 /UG_GENE=6330544B05Rik /UG_TITLE=RIKEN cDNA 6330544B05 gene /DEF=Mus musculus adult male testis cDNA, RIKEN full-length enriched library, clone:1700027H16:homolog to ARF-LIKE 2 BINDING PROTEIN BART1 (BINDER OF ARL TWO), full insert sequence.	1334.9333	456.44818	879.5999	167.46419	1.6220952	2.0	0.24624552	0.28993425	0.66	1.52
gb:AF305501.1 /DB_XREF=gi:10764832 /FEA=FLmRNA /CNT=12 /TID=Mm.29744.3 /TIER=FL /STK=1 /UG=Mm.29744 /LL=63959 /UG_GENE=Slc29a1 /DEF=Mus musculus equilibrative nitrobenzylthioinosine-sensitive nucleoside transporter ENT1b mRNA, complete cds, alternatively spliced. /PROD=equilibrative nitrobenzylthioinosine-sensitivenucleoside transporter ENT1b /FL=gb:AF305501.1	722.4666	340.17065	391.33334	98.5355	1.6194623	2.0	0.24677461	0.28962293	0.54	1.85
gb:C76322 /DB_XREF=gi:2516652 /DB_XREF=C76322 /CLONE=J0008F11 /FEA=FLmRNA /CNT=215 /TID=Mm.22632.1 /TIER=Stack /STK=32 /UG=Mm.22632 /UG_TITLE=Mus musculus, Similar to transgelin 2, clone MGC:6300 IMAGE:2654381, mRNA, complete cds /FL=gb:BC009076.1 gb:AF149291.1	1422.2999	547.77124	888.6667	163.41887	1.6169243	2.0	0.247286	0.28929293	0.62	1.6
gb:AK012780.1 /DB_XREF=gi:12849748 /FEA=mRNA /CNT=10 /TID=Mm.1980.2 /TIER=ConsEnd /STK=6 /UG=Mm.1980 /LL=69253 /UG_GENE=Hspb2 /UG_TITLE=heat shock 27kD protein 2 /DEF=Mus musculus 10, 11 days embryo whole body cDNA, RIKEN full-length enriched library, clone:2810021G24:homolog to HEAT-SHOCK PROTEIN, BETA-2 (DMPK-BINDING PROTEIN) (MKBP), full insert sequence.	1949.6333	717.2411	1206.8	345.3317	1.6162702	2.0	0.24741803	0.28852263	0.62	1.62
gb:NM_021304.2 /DB_XREF=gi:15011863 /GEN=Labh1-pending /FEA=FLmRNA /CNT=54 /TID=Mm.80532.1 /TIER=FL+Stack /STK=29 /UG=Mm.80532 /LL=57742 /DEF=Mus musculus lung alphabeta hydrolase 1 (Labh1-pending), mRNA. /PROD=lung alphabeta hydrolase 1 /FL=gb:AF189764.2 gb:NM_021304.2 gb:BC013505.1	652.4666	206.55586	427.63333	178.43198	1.4267027	3.0	0.24893364	0.28936556	0.66	1.53
gb:NM_138749.1 /DB_XREF=gi:20270280 /GEN=Plxnb2 /FEA=FLmRNA /CNT=197 /TID=Mm.28780.1 /TIER=FL+Stack /STK=74 /UG=Mm.28780 /LL=140570 /DEF=Mus musculus plexin B2 (Plxnb2), mRNA. /PROD=plexin B2 /FL=gb:NM_138749.1 gb:BC007481.1 gb:BC003293.1	1338.4667	664.8858	697.9333	183.63422	1.6083946	2.0	0.24901477	0.28854093	0.52	1.92
gb:NM_008816.1 /DB_XREF=gi:6679272 /GEN=Pecam /FEA=FLmRNA /CNT=15 /TID=Mm.2822.1 /TIER=FL /STK=2 /UG=Mm.2822 /LL=18613 /DEF=Mus musculus plateletendothelial cell adhesion molecule (Pecam), mRNA. /PROD=plateletendothelial cell adhesion molecule /FL=gb:NM_008816.1 gb:L06039.1	1024.2666	261.8756	663.30005	358.61865	1.407957	3.0	0.2538771	0.29324412	0.65	1.54
gb:NM_007597.1 /DB_XREF=gi:6671663 /GEN=Canx /FEA=FLmRNA /CNT=222 /TID=Mm.153481.1 /TIER=FL+Stack /STK=9 /UG=Mm.153481 /LL=12330 /DEF=Mus musculus calnexin (Canx), mRNA. /PROD=calnexin /FL=gb:L18888.1 gb:NM_007597.1 gb:BC012408.1	673.89996	228.80792	448.30002	95.59043	1.5757788	2.0	0.25577018	0.2944988	0.67	1.5
gb:BC005632.1 /DB_XREF=gi:13542873 /FEA=FLmRNA /CNT=155 /TID=Mm.29802.1 /TIER=FL+Stack /STK=69 /UG=Mm.29802 /DEF=Mus musculus, Similar to CGI-67 protein, clone MGC:11699 IMAGE:3964094, mRNA, complete cds. /PROD=Similar to CGI-67 protein /FL=gb:BC005632.1	960.5667	325.30927	635.3333	165.06606	1.5442263	2.0	0.26253024	0.30133188	0.66	1.51
gb:BC012707.1 /DB_XREF=gi:15215221 /FEA=FLmRNA /CNT=73 /TID=Mm.24118.1 /TIER=FL+Stack /STK=34 /UG=Mm.24118 /LL=14872 /UG_GENE=Gstt2 /DEF=Mus musculus, glutathione S-transferase, theta 2, clone MGC:13991 IMAGE:3994154, mRNA, complete cds. /PROD=glutathione S-transferase, theta 2 /FL=gb:NM_010361.1 gb:BC012707.1 gb:U48420.1	445.63333	176.02786	271.19998	89.63192	1.5294957	2.0	0.26576418	0.30408755	0.61	1.64
gb:NM_013670.1 /DB_XREF=gi:7305508 /GEN=Snrpn /FEA=FLmRNA /CNT=253 /TID=Mm.43721.1 /TIER=FL+Stack /STK=178 /UG=Mm.43721 /LL=20646 /DEF=Mus musculus small nuclear ribonucleoprotein N (Snrpn), mRNA. /PROD=small nuclear ribonucleoprotein N /FL=gb:BC024880.1 gb:NM_013670.1 gb:BC019589.1	3676.5	1964.3711	1895.9	476.49503	1.5257676	2.0	0.26659068	0.30408	0.52	1.94
gb:NM_011793.1 /DB_XREF=gi:6753177 /GEN=Bcrp1-pending /FEA=FLmRNA /CNT=16 /TID=Mm.7508.1 /TIER=FL /STK=2 /UG=Mm.7508 /LL=23825 /DEF=Mus musculus breakpoint cluster region protein 1 (Bcrp1-pending), mRNA. /PROD=breakpoint cluster region protein 1 /FL=gb:NM_011793.1 gb:AB025349.1	1313.1333	341.6223	867.7	460.86154	1.3448688	3.0	0.27130315	0.3084911	0.66	1.51
gb:NM_022318.1 /DB_XREF=gi:11612496 /GEN=Pop2-pending /FEA=FLmRNA /CNT=49 /TID=Mm.23890.1 /TIER=FL+Stack /STK=36 /UG=Mm.23890 /LL=64082 /DEF=Mus musculus popeye 2 (Pop2-pending), mRNA. /PROD=popeye 2 /FL=gb:AF204175.1 gb:NM_022318.1	7395.8667	2724.9272	4811.0	1195.4429	1.504601	2.0	0.2713454	0.30758098	0.65	1.54
gb:NM_031176.1 /DB_XREF=gi:13928671 /GEN=Tnxb /FEA=FLmRNA /CNT=13 /TID=Mm.183266.1 /TIER=FL /STK=2 /UG=Mm.183266 /LL=81877 /DEF=Mus musculus tenascin XB (Tnxb), mRNA. /PROD=tenascin XB /FL=gb:NM_031176.1	1731.1666	907.9988	932.7	486.432	1.342591	3.0	0.27195567	0.30731833	0.54	1.86
gb:BC021637.1 /DB_XREF=gi:18204915 /FEA=FLmRNA /CNT=41 /TID=Mm.15819.1 /TIER=FL+Stack /STK=20 /UG=Mm.15819 /LL=12514 /UG_GENE=Cd68 /DEF=Mus musculus, CD68 antigen, clone MGC:13848 IMAGE:3981024, mRNA, complete cds. /PROD=CD68 antigen /FL=gb:BC021637.1 gb:NM_009853.1	839.6333	224.20682	550.23334	301.93274	1.3328619	3.0	0.27476168	0.3095309	0.66	1.53
gb:BC021408.1 /DB_XREF=gi:18204099 /FEA=FLmRNA /CNT=13 /TID=Mm.218625.1 /TIER=FL /STK=7 /UG=Mm.218625 /DEF=Mus musculus, clone MGC:29439 IMAGE:3964501, mRNA, complete cds. /PROD=Unknown (protein for MGC:29439) /FL=gb:BC021408.1	416.5	199.25493	240.03333	52.015617	1.4842213	2.0	0.27602518	0.3099975	0.58	1.74
gb:NM_007598.1 /DB_XREF=gi:6671665 /GEN=Cap1 /FEA=FLmRNA /CNT=180 /TID=Mm.8687.1 /TIER=FL /STK=2 /UG=Mm.8687 /LL=12331 /DEF=Mus musculus adenylyl cyclase-associated CAP protein homolog 1 (S. cerevisiae, S. pombe) (Cap1), mRNA. /PROD=adenylyl cyclase-associated CAP protein, yeasthomolog 1 /FL=gb:BC005446.1 gb:BC005472.1 gb:NM_007598.1 gb:L12367.1	701.73334	249.20934	462.16666	192.9016	1.3166695	3.0	0.2794998	0.3129369	0.66	1.52
gb:BC002136.1 /DB_XREF=gi:12805334 /FEA=FLmRNA /CNT=197 /TID=Mm.27317.1 /TIER=FL+Stack /STK=107 /UG=Mm.27317 /LL=12721 /UG_GENE=Coro1a /DEF=Mus musculus, Similar to coronin, actin binding protein 1A, clone MGC:7245 IMAGE:3484185, mRNA, complete cds. /PROD=Similar to coronin, actin binding protein 1A /FL=gb:BC002136.1 gb:NM_009898.1 gb:AF047388.1	503.53336	182.02663	331.19998	91.522285	1.4650537	2.0	0.28051975	0.31311837	0.66	1.52
gb:BC002090.1 /DB_XREF=gi:12805254 /FEA=FLmRNA /CNT=31 /TID=Mm.27128.1 /TIER=FL /STK=2 /UG=Mm.27128 /LL=66556 /UG_GENE=2310074H19Rik /DEF=Mus musculus, Similar to DR1-associated protein 1 (negative cofactor 2 alpha), clone MGC:6341 IMAGE:3488139, mRNA, complete cds. /PROD=Similar to DR1-associated protein 1 (negativecofactor 2 alpha) /FL=gb:BC002090.1	3833.4668	1725.8673	2366.5999	262.58167	1.4553742	2.0	0.28282443	0.3147284	0.62	1.62
gb:AK011499.1 /DB_XREF=gi:12847662 /FEA=FLmRNA /CNT=196 /TID=Mm.21707.1 /TIER=Stack /STK=16 /UG=Mm.21707 /LL=67819 /UG_GENE=1110021N07Rik /UG_TITLE=RIKEN cDNA 1110021N07 gene /DEF=Mus musculus 10 days embryo whole body cDNA, RIKEN full-length enriched library, clone:2610021E07:related to F25D7.1 PROTEIN, full insert sequence. /FL=gb:NM_024207.1 gb:BC003454.1	587.36664	221.63065	383.20004	101.27491	1.4512329	2.0	0.2838177	0.31487375	0.65	1.53
gb:NM_029796.1 /DB_XREF=gi:16418334 /GEN=Lrg-pending /FEA=FLmRNA /CNT=132 /TID=Mm.176946.1 /TIER=FL+Stack /STK=49 /UG=Mm.176946 /LL=76905 /DEF=Mus musculus leucine-rich alpha-2-glycoprotein (Lrg-pending), mRNA. /PROD=leucine-rich alpha-2-glycoprotein /FL=gb:AF403429.1 gb:AB055885.1 gb:NM_029796.1	990.1667	377.34503	612.86664	335.041	1.2950386	3.0	0.2859639	0.3162934	0.62	1.62
gb:NM_007607.1 /DB_XREF=gi:6671677 /GEN=Car4 /FEA=FLmRNA /CNT=72 /TID=Mm.1641.1 /TIER=FL+Stack /STK=30 /UG=Mm.1641 /LL=12351 /DEF=Mus musculus carbonic anhydrase 4 (Car4), mRNA. /PROD=carbonic anhydrase 4 /FL=gb:NM_007607.1 gb:BC012704.1	727.7666	346.57535	436.16666	190.54303	1.2770278	3.0	0.29146564	0.3214047	0.6	1.67
gb:NM_010907.1 /DB_XREF=gi:6754839 /GEN=Nfkbia /FEA=FLmRNA /CNT=183 /TID=Mm.8884.1 /TIER=FL+Stack /STK=97 /UG=Mm.8884 /LL=18035 /DEF=Mus musculus nuclear factor of kappa light chain gene enhancer in B-cells inhibitor, alpha (Nfkbia), mRNA. /PROD=nuclear factor of kappa light chain geneenhancer in B-cells inhibitor, alpha /FL=gb:NM_010907.1 gb:U36277.1	2398.0999	1137.4896	1406.9668	523.849	1.3708122	2.0	0.30399716	0.33421373	0.59	1.7
gb:NM_010828.1 /DB_XREF=gi:6754741 /GEN=Cited2 /FEA=FLmRNA /CNT=9 /TID=Mm.9524.1 /TIER=FL /STK=2 /UG=Mm.9524 /LL=17684 /DEF=Mus musculus Cbpp300-interacting transactivator, with GluAsp-rich carboxy-terminal domain, 2 (Cited2), mRNA. /PROD=melanocyte specific gene 2 /FL=gb:NM_010828.1 gb:U86445.1	639.86664	303.79355	393.63333	70.07797	1.3679527	2.0	0.30474684	0.33403182	0.62	1.63
gb:AF282255.1 /DB_XREF=gi:9652249 /GEN=Dscr1 /FEA=FLmRNA /CNT=119 /TID=Mm.56.1 /TIER=FL /STK=1 /UG=Mm.56 /LL=54720 /DEF=Mus musculus Down syndrome candidate region 1 protein (Dscr1) mRNA, complete cds. /PROD=Down syndrome candidate region 1 protein /FL=gb:AF260717.1 gb:AF282255.1 gb:AF237789.1 gb:NM_019466.1	870.0999	438.59137	487.6	314.48032	1.2275844	3.0	0.30714175	0.33564892	0.56	1.78
gb:NM_008802.1 /DB_XREF=gi:6679246 /GEN=Pde7a /FEA=FLmRNA /CNT=63 /TID=Mm.5034.1 /TIER=ConsEnd /STK=0 /UG=Mm.5034 /LL=18583 /DEF=Mus musculus phosphodiesterase 7A (Pde7a), mRNA. /PROD=phosphodiesterase 7A /FL=gb:U68171.1 gb:NM_008802.1	368.5	157.21631	238.13335	95.86294	1.2262657	3.0	0.30757156	0.33511528	0.65	1.55
gb:NM_013609.1 /DB_XREF=gi:7305312 /GEN=Ngfb /FEA=FLmRNA /CNT=26 /TID=Mm.1259.1 /TIER=FL /STK=7 /UG=Mm.1259 /LL=18049 /DEF=Mus musculus nerve growth factor, beta (Ngfb), mRNA. /PROD=nerve growth factor, beta /FL=gb:BC011123.1 gb:NM_013609.1 gb:M35075.1 gb:M14805.1	412.63333	183.96332	258.86667	115.96821	1.2247092	3.0	0.3080797	0.3346699	0.63	1.59
gb:NM_011122.1 /DB_XREF=gi:6755105 /GEN=Plod1 /FEA=FLmRNA /CNT=198 /TID=Mm.37371.1 /TIER=FL+Stack /STK=101 /UG=Mm.37371 /LL=18822 /DEF=Mus musculus procollagen-lysine, 2-oxoglutarate 5-dioxygenase 1 (Plod1), mRNA. /PROD=procollagen-lysine, 2-oxoglutarate 5-dioxygenase1 /FL=gb:BC010268.1 gb:BC006599.1 gb:AF046782.1 gb:NM_011122.1	1086.6333	485.95712	700.2	121.54659	1.3361669	2.0	0.313235	0.33926046	0.64	1.55
gb:BC014690.1 /DB_XREF=gi:15928416 /FEA=FLmRNA /CNT=145 /TID=Mm.1291.1 /TIER=FL+Stack /STK=44 /UG=Mm.1291 /LL=21809 /UG_GENE=Tgfb3 /DEF=Mus musculus, Transforming growth factor, beta 3, clone MGC:18512 IMAGE:3663150, mRNA, complete cds. /PROD=Transforming growth factor, beta 3 /FL=gb:NM_009368.1 gb:M32745.1 gb:BC014690.1	1086.0333	464.13068	691.8667	225.32773	1.3232585	2.0	0.31676453	0.3420682	0.64	1.57
gb:NM_011116.1 /DB_XREF=gi:7242180 /GEN=Pld3 /FEA=FLmRNA /CNT=258 /TID=Mm.6483.1 /TIER=FL+Stack /STK=145 /UG=Mm.6483 /LL=18807 /DEF=Mus musculus phospholipase D3 (Pld3), mRNA. /PROD=phospholipase D3 /FL=gb:AF026124.1 gb:NM_011116.1	607.39996	275.89005	383.2667	106.79449	1.3122373	2.0	0.31981644	0.34434512	0.63	1.58
gb:NM_138598.1 /DB_XREF=gi:20070413 /GEN=D11Wsu99e /FEA=FLmRNA /CNT=114 /TID=Mm.99677.1 /TIER=FL+Stack /STK=16 /UG=Mm.99677 /LL=28081 /DEF=Mus musculus DNA segment, Chr 11, Wayne State University 99, expressed (D11Wsu99e), mRNA. /PROD=DNA segment, Chr 11, Wayne State University 99,expressed /FL=gb:NM_138598.1	276.23334	133.28076	176.09999	32.544277	1.264143	2.0	0.33355832	0.35808465	0.64	1.57
gb:NM_011340.1 /DB_XREF=gi:6755443 /GEN=Serpinf1 /FEA=FLmRNA /CNT=296 /TID=Mm.2044.1 /TIER=FL+Stack /STK=119 /UG=Mm.2044 /LL=20317 /DEF=Mus musculus serine (or cysteine) proteinase inhibitor, clade F (alpha-2 antiplasmin, pigment epithelium derived factor), member 1 (Serpinf1), mRNA. /PROD=serine (or cysteine) proteinase inhibitor, cladeF (alpha-2 antiplasmin, pigment epithelium derivedfactor), member 1 /FL=gb:D50460.1 gb:AF036164.1 gb:D87975.1 gb:NM_011340.1 gb:AF017057.1 gb:BC019852.1	6529.9336	3005.3062	4241.4	1786.3384	1.1337867	3.0	0.3392925	0.36317232	0.65	1.54
gb:AK004646.1 /DB_XREF=gi:12835962 /FEA=mRNA /CNT=40 /TID=Mm.200636.1 /TIER=Stack /STK=11 /UG=Mm.200636 /LL=71710 /UG_GENE=1200008A14Rik /UG_TITLE=RIKEN cDNA 1200008A14 gene /DEF=Mus musculus adult male lung cDNA, RIKEN full-length enriched library, clone:1200008A14:Leucine-rich repeat containing protein, full insert sequence.	249.0	130.56323	155.23334	16.480694	1.2341149	2.0	0.34249836	0.36553186	0.62	1.6
gb:NM_012043.1 /DB_XREF=gi:6754373 /GEN=Islr /FEA=FLmRNA /CNT=72 /TID=Mm.38426.1 /TIER=FL+Stack /STK=23 /UG=Mm.38426 /LL=26968 /DEF=Mus musculus immunoglobulin superfamily containing leucine-rich repeat (Islr), mRNA. /PROD=immunoglobulin superfamily containingleucine-rich repeat /FL=gb:BC006602.1 gb:NM_012043.1 gb:AB024538.1	2557.5	1151.92	1698.9668	672.7698	1.1147153	3.0	0.34623244	0.36843976	0.66	1.51
gb:BC009652.1 /DB_XREF=gi:16307128 /FEA=FLmRNA /CNT=37 /TID=Mm.1418.1 /TIER=FL+Stack /STK=19 /UG=Mm.1418 /LL=20266 /UG_GENE=Scn1b /DEF=Mus musculus, sodium channel, voltage-gated, type I, beta polypeptide, clone MGC:5891 IMAGE:3489368, mRNA, complete cds. /PROD=sodium channel, voltage-gated, type I, betapolypeptide /FL=gb:U46681.1 gb:NM_011322.1 gb:BC009652.1 gb:U85786.1	635.0333	361.45532	368.93335	135.81224	1.1936418	2.0	0.3550031	0.37667477	0.58	1.72
gb:AK011360.1 /DB_XREF=gi:12847432 /FEA=mRNA /CNT=64 /TID=Mm.142187.1 /TIER=Stack /STK=24 /UG=Mm.142187 /LL=69202 /UG_GENE=2610009E16Rik /UG_TITLE=RIKEN cDNA 2610009E16 gene /DEF=Mus musculus 10 days embryo whole body cDNA, RIKEN full-length enriched library, clone:2610009E16:homolog to PARATHYMOSIN (ZINC-BINDING 11.5 KDA PROTEIN), full insert sequence.	3434.7334	1605.6941	2261.0	795.5188	1.1344959	2.0	0.37425452	0.39595047	0.66	1.52
gb:NM_007936.1 /DB_XREF=gi:6679656 /GEN=Epha4 /FEA=FLmRNA /CNT=39 /TID=Mm.3249.1 /TIER=FL /STK=1 /UG=Mm.3249 /LL=13838 /DEF=Mus musculus Eph receptor A4 (Epha4), mRNA. /PROD=Eph receptor A4 /FL=gb:NM_007936.1 gb:BC004782.1	439.93335	226.86636	287.86667	60.133537	1.1222262	2.0	0.3783984	0.3991775	0.65	1.53
gb:BC025618.1 /DB_XREF=gi:19343735 /FEA=FLmRNA /CNT=513 /TID=Mm.205791.1 /TIER=FL+Stack /STK=208 /UG=Mm.205791 /DEF=Mus musculus, clone MGC:38134 IMAGE:5320976, mRNA, complete cds. /PROD=Unknown (protein for MGC:38134) /FL=gb:BC025811.1 gb:BC025618.1 gb:BC025627.1 gb:BC021496.1	4273.3335	4025.0786	1660.7999	424.9315	1.117999	2.0	0.3798383	0.39954174	0.39	2.57
gb:NM_011693.1 /DB_XREF=gi:6755960 /GEN=Vcam1 /FEA=FLmRNA /CNT=224 /TID=Mm.1021.1 /TIER=FL+Stack /STK=153 /UG=Mm.1021 /LL=22329 /DEF=Mus musculus vascular cell adhesion molecule 1 (Vcam1), mRNA. /PROD=vascular cell adhesion molecule 1 /FL=gb:NM_011693.1 gb:M84487.1	1582.9332	759.63043	1035.5	381.54373	1.1154205	2.0	0.38071966	0.39931804	0.65	1.53
gb:AI852593 /DB_XREF=gi:5496499 /DB_XREF=UI-M-BH0-aiu-b-12-0-UI.s1 /CLONE=UI-M-BH0-aiu-b-12-0-UI /FEA=mRNA /CNT=61 /TID=Mm.26671.1 /TIER=Stack /STK=10 /UG=Mm.26671 /UG_TITLE=Mus musculus, clone IMAGE:3487914, mRNA	1663.8334	910.53546	1027.2001	404.96207	1.1065224	2.0	0.38377926	0.40137374	0.62	1.62
gb:U29539.1 /DB_XREF=gi:924941 /FEA=FLmRNA /CNT=1 /TID=MmAffx.1.13 /TIER=FL /STK=1 /NOTE=sequence(s) not in UniGene /DEF=Mus musculus retinoic acid-inducible E3 protein mRNA, complete cds. /PROD=E3 protein /FL=gb:U29539.1	440.80002	237.59859	282.53333	75.13217	1.1000478	2.0	0.38602325	0.40256712	0.64	1.56

gb:L42114.1 /DB_XREF=gi:18654373 /GEN=GDF10 /FEA=FLmRNA /CNT=85 /TID=Mm.40323.1 /TIER=FL+Stack /STK=65 /UG=Mm.40323 /DEF=Mus musculus growthdifferentiation factor 10 (GDF10) mRNA, complete cds. /PROD=growthdifferentiation factor 10 /FL=gb:L42114.1	339.76666	192.45802	215.83333	47.5599	1.0827825	2.0	0.39208058	0.40771914	0.64	1.57
gb:NM_007657.1 /DB_XREF=gi:6680893 /GEN=Cd9 /FEA=FLmRNA /CNT=324 /TID=Mm.2956.1 /TIER=FL+Stack /STK=135 /UG=Mm.2956 /LL=12527 /DEF=Mus musculus CD9 antigen (Cd9), mRNA. /PROD=CD9 antigen /FL=gb:L08115.1 gb:NM_007657.1	2590.3333	1327.7048	1686.2667	684.79297	1.0481877	2.0	0.40454426	0.41948482	0.65	1.54
gb:AK004342.1 /DB_XREF=gi:12835491 /FEA=mRNA /CNT=37 /TID=Mm.178550.2 /TIER=Stack /STK=19 /UG=Mm.178550 /LL=66478 /UG_GENE=2410026K10Rik /UG_TITLE=RIKEN cDNA 2410026K10 gene /DEF=Mus musculus 18 days embryo whole body cDNA, RIKEN full-length enriched library, clone:1110061M03:homolog to T-CELL SURFACE GLYCOPROTEIN E2 PRECURSOR (E2 ANTIGEN) (CD99) (MIC2 PROTEIN) (12E7), full insert sequence.	849.5334	656.2247	453.93335	76.852875	1.0370659	2.0	0.40864527	0.4225369	0.53	1.87
gb:BC010564.1 /DB_XREF=gi:14714826 /FEA=FLmRNA /CNT=83 /TID=Mm.219659.1 /TIER=FL+Stack /STK=24 /UG=Mm.219659 /LL=15267 /UG_GENE=Hist2 /DEF=Mus musculus, H2A histone family, member O, clone MGC:5956 IMAGE:3582122, mRNA, complete cds. /PROD=H2A histone family, member O /FL=gb:NM_013549.1 gb:BC010564.1	1537.0668	912.65375	992.8333	134.19629	1.0218685	2.0	0.41432413	0.42719862	0.65	1.55
gb:BC015459.1 /DB_XREF=gi:15930030 /FEA=FLmRNA /CNT=83 /TID=Mm.18814.1 /TIER=FL+Stack /STK=10 /UG=Mm.18814 /LL=66898 /UG_GENE=1300006M19Rik /DEF=Mus musculus, RIKEN cDNA 1300006M19 gene, clone MGC:8269 IMAGE:3592632, mRNA, complete cds. /PROD=RIKEN cDNA 1300006M19 gene /FL=gb:BC015459.1	992.26666	1056.4161	371.96667	48.13329	1.015961	2.0	0.41655517	0.42828912	0.37	2.67
gb:NM_010890.1 /DB_XREF=gi:13994148 /GEN=Nedd4a /FEA=FLmRNA /CNT=23 /TID=Mm.16553.1 /TIER=FL /STK=1 /UG=Mm.16553 /LL=17999 /DEF=Mus musculus neural precursor cell expressed, developmentally down-regulated gene 4a (Nedd4a), mRNA. /PROD=neural precursor cell expressed, developmentallydown-regulated gene 4a /FL=gb:NM_010890.1	1768.7333	992.5988	1140.6	479.7904	0.98683304	2.0	0.42775115	0.4385651	0.64	1.55
gb:BC019775.1 /DB_XREF=gi:18043602 /FEA=FLmRNA /CNT=156 /TID=Mm.214514.1 /TIER=FL+Stack /STK=98 /UG=Mm.214514 /LL=116847 /UG_GENE=Prelp /DEF=Mus musculus, proline arginine-rich end leucine-rich repeat, clone MGC:25825 IMAGE:4165772, mRNA, complete cds. /PROD=proline arginine-rich end leucine-rich repeat /FL=gb:BC019775.1 gb:NM_054077.1	3675.5667	1965.7422	2444.2334	1320.7708	0.90055424	3.0	0.4341985	0.44392845	0.66	1.5
gb:AK008203.1 /DB_XREF=gi:12842246 /FEA=FLmRNA /CNT=83 /TID=Mm.18814.1 /TIER=Stack /STK=25 /UG=Mm.18814 /LL=66898 /UG_GENE=1300006M19Rik /UG_TITLE=RIKEN cDNA 1300006M19 gene /DEF=Mus musculus adult male small intestine cDNA, RIKEN full-length enriched library, clone:2010012D17:homolog to INSULIN RECEPTOR TYROSINE KINASE SUBSTRATE, full insert sequence. /FL=gb:BC015459.1	2052.7	2641.6304	592.10004	181.3516	0.95542985	2.0	0.4401908	0.44879788	0.29	3.47
gb:NM_013550.1 /DB_XREF=gi:7305140 /GEN=Hist4 /FEA=FLmRNA /CNT=9 /TID=Mm.193557.1 /TIER=FL /STK=1 /UG=Mm.193557 /LL=15269 /DEF=Mus musculus histone 4 protein (Hist4), mRNA. /PROD=histone 4 protein /FL=gb:NM_013550.1	2056.1667	1616.3438	1194.2001	14.616449	0.9236334	2.0	0.45318398	0.4607581	0.58	1.72
gb:AB010742.1 /DB_XREF=gi:3062840 /FEA=FLmRNA /CNT=27 /TID=Mm.6254.1 /TIER=FL /STK=3 /UG=Mm.6254 /LL=22229 /UG_GENE=Ucp3 /DEF=Mus musculus mRNA for UCP3, complete cds. /PROD=UCP3 /FL=gb:AB010742.1 gb:AF030164.1 gb:NM_009464.1 gb:AB008216.1 gb:AF053352.1 gb:AF032902.1	529.93335	380.70724	337.53336	105.21071	0.84371	2.0	0.4876572	0.4944302	0.64	1.57
gb:NM_017370.1 /DB_XREF=gi:8850218 /GEN=Hp /FEA=FLmRNA /CNT=154 /TID=Mm.26730.1 /TIER=FL+Stack /STK=33 /UG=Mm.26730 /LL=15439 /DEF=Mus musculus haptoglobin (Hp), mRNA. /PROD=haptoglobin /FL=gb:NM_017370.1 gb:M96827.1	705.19995	506.99698	440.69998	253.31256	0.8083316	2.0	0.5037641	0.50934595	0.62	1.6
gb:NM_010180.1 /DB_XREF=gi:6753821 /GEN=Fbln1 /FEA=FLmRNA /CNT=91 /TID=Mm.219663.1 /TIER=FL+Stack /STK=41 /UG=Mm.219663 /LL=14114 /DEF=Mus musculus fibulin 1 (Fbln1), mRNA. /PROD=fibulin 1 /FL=gb:NM_010180.1	835.6333	728.61206	505.8666	168.10336	0.76385224	2.0	0.52476585	0.5291147	0.61	1.65
gb:NM_009052.1 /DB_XREF=gi:6677720 /GEN=Rex3 /FEA=FLmRNA /CNT=77 /TID=Mm.14768.1 /TIER=FL+Stack /STK=56 /UG=Mm.14768 /LL=19716 /DEF=Mus musculus reduced expression 3 (Rex3), mRNA. /PROD=reduced expression 3 /FL=gb:AF051347.1 gb:NM_009052.1 gb:AF097438.1	1297.2999	1452.1323	657.06665	84.7658	0.76234937	2.0	0.5254902	0.52838546	0.51	1.97
gb:AK004564.1 /DB_XREF=gi:12835818 /FEA=mRNA /CNT=74 /TID=Mm.46346.1 /TIER=Stack /STK=30 /UG=Mm.46346 /LL=66866 /UG_GENE=1200003G01Rik /UG_TITLE=RIKEN cDNA 1200003G01 gene /DEF=Mus musculus adult male lung cDNA, RIKEN full-length enriched library, clone:1200003G01:NHL repeat containing protein, full insert sequence.	495.19998	386.70343	330.69998	64.495186	0.72675955	2.0	0.5429257	0.54441726	0.67	1.5
gb:NM_054037.1 /DB_XREF=gi:16905068 /GEN=Scgb3a1 /FEA=FLmRNA /CNT=13 /TID=Mm.22802.1 /TIER=FL /STK=7 /UG=Mm.22802 /LL=68662 /DEF=Mus musculus secretoglobin, family 3A, member 1 (Scgb3a1), mRNA. /PROD=UGRP2 type A /FL=gb:NM_054037.1 gb:AF313456.1	6362.733	6998.1064	3526.5332	5637.334	0.5466609	3.0	0.62264764	0.62264764	0.55	1.8
