# Supplementary material for: Protein Phosphatase 2A Improves Cardiac Functional Response to Ischemia and Sepsis
Source: Int J Mol Sci. 2022 Apr 23;23(9):4688. doi: 10.3390/ijms23094688 (PMC9101092; doi:10.3390/ijms23094688)
Supplement: Supplementary file 1 [file ijms-23-04688-s001.zip › Supplementary Table S3.pdf]

Supplementary Table S3. Cardiac gene expression. A GeneChip Mouse Genome 430 2.0 Array (Affymetrix, Santa Clara, CA, United States) was used to analyze the basal mRNA expression in hearts from PP2A-TG (n=3) and WT (n=3) mice. For analysis, the TM4 application MeV was used (Saeed et al., 2003). Here, analysis was performed with genes that were expressed in both PP2A-TG and WT. First, data were adjusted by a variance filter (percent mode; value 50) and, secondly, analyzed by a t-Test (Welch approximation; alpha (overall threshold p-value): 0.05; P-values based on t-distribution).

Gene descriptions	WT mean	WT SD	PP2A-TG mean	PP2A-TG SD	Absolute t value	Degrees of freedom	Adj p value	False discovery rate
gb:NM_007981.1 /DB_XREF=gi:6679738 /GEN=Facl2 /FEA=FLmRNA /CNT=147 /TID=Mm.28962.1 /TIER=FL+Stack /STK=45 /UG=Mm.28962 /LL=14081 /DEF=Mus musculus fatty acid Coenzyme A ligase, long chain 2 (Facl2), mRNA. /PROD=fatty acid Coenzyme A ligase, long chain 2 /FL=gb:NM_007981.1 gb:U15977.1	3965.5664	415.30035	2066.2332	226.85002	6.9518514	3.0	0.006105606	2.7719452
gb:AK003586.1 /DB_XREF=gi:12834343 /FEA=mRNA /CNT=119 /TID=Mm.20488.1 /TIER=Stack /STK=50 /UG=Mm.20488 /LL=76301 /UG_GENE=1110008K06Rik /UG_TITLE=RIKEN cDNA 1110008K06 gene /DEF=Mus musculus 18 days embryo whole body cDNA, RIKEN full-length enriched library, clone:1110008K06:homolog to FLN29 (FLN29 GENE PRODUCT), full insert sequence.	1412.2666	172.08234	591.0333	137.58932	6.455995	3.0	0.007538527	1.7112457
gb:BC006668.1 /DB_XREF=gi:13879379 /FEA=FLmRNA /CNT=135 /TID=Mm.20396.1 /TIER=FL+Stack /STK=60 /UG=Mm.20396 /LL=12908 /UG_GENE=Crat /DEF=Mus musculus, Similar to carnitine acetyltransferase, clone MGC:6046 IMAGE:3499169, mRNA, complete cds. /PROD=Similar to carnitine acetyltransferase /FL=gb:BC006668.1 gb:NM_007760.1	4650.733	358.1267	2468.2334	466.95862	6.4237013	3.0	0.007646559	1.1571792
gb:NM_007533.1 /DB_XREF=gi:6671623 /GEN=Bckdha /FEA=FLmRNA /CNT=211 /TID=Mm.25848.1 /TIER=FL+Stack /STK=76 /UG=Mm.25848 /LL=12039 /DEF=Mus musculus branched chain ketoacid dehydrogenase E1, alpha polypeptide (Bckdha), mRNA. /PROD=branched chain ketoacid dehydrogenase E1, alphapolypeptide /FL=gb:BC003787.1 gb:NM_007533.1 gb:L47335.1	2700.0334	286.70358	1546.7666	192.90247	5.780555	3.0	0.010295443	1.1685328
gb:BC004804.1 /DB_XREF=gi:13435929 /FEA=FLmRNA /CNT=277 /TID=Mm.104540.1 /TIER=FL+Stack /STK=115 /UG=Mm.104540 /LL=108124 /UG_GENE=Napa /DEF=Mus musculus, RIKEN cDNA 1500039N14 gene, clone MGC:6399 IMAGE:3584869, mRNA, complete cds. /PROD=RIKEN cDNA 1500039N14 gene /FL=gb:NM_025898.1 gb:BC004804.1	3788.9336	236.00606	2382.4668	356.9891	5.692439	3.0	0.010747662	0.9758877
gb:AB056479.1 /DB_XREF=gi:15341205 /FEA=FLmRNA /CNT=427 /TID=Mm.43822.1 /TIER=FL+Stack /STK=32 /UG=Mm.43822 /LL=12974 /UG_GENE=Cs /DEF=Mus musculus mRNA for citrate synthase, complete cds. /PROD=citrate synthase /FL=gb:BC013554.1 gb:NM_026444.1 gb:AB056479.1	3668.7334	270.4928	2305.8333	327.08664	5.5616684	3.0	0.011467959	0.8677422
gb:NM_008916.1 /DB_XREF=gi:6679448 /GEN=Pps /FEA=FLmRNA /CNT=174 /TID=Mm.1458.1 /TIER=FL+Stack /STK=115 /UG=Mm.1458 /LL=19062 /DEF=Mus musculus putative phosphatase (Pps), mRNA. /PROD=putative phosphatase /FL=gb:AF483523.1 gb:AF483522.1 gb:NM_008916.1 gb:U96724.1	1440.2001	142.18462	787.8	161.60974	5.2495723	3.0	0.013461299	0.8730614
gb:NM_007906.1 /DB_XREF=gi:6681272 /GEN=Eef1a2 /FEA=FLmRNA /CNT=64 /TID=Mm.2645.1 /TIER=FL+Stack /STK=31 /UG=Mm.2645 /LL=13628 /DEF=Mus musculus eukaryotic translation elongation factor 1 alpha 2 (Eef1a2), mRNA. /PROD=eukaryotic translation elongation factor 1 alpha2 /FL=gb:L26479.1 gb:NM_007906.1 gb:BC018235.1	17134.666	2077.833	9926.967	1596.6765	4.7641034	3.0	0.017563034	0.9967022
gb:NM_029632.1 /DB_XREF=gi:18390326 /GEN=Ppp1r11 /FEA=FLmRNA /CNT=146 /TID=Mm.46176.1 /TIER=FL+Stack /STK=58 /UG=Mm.46176 /LL=76497 /DEF=Mus musculus protein phosphatase 1, regulatory (inhibitor) subunit 11 (Ppp1r11), mRNA. /PROD=protein phosphatase 1, regulatory (inhibitor)subunit 11 /FL=gb:NM_029632.1	3319.6667	170.52585	1640.5333	382.8957	6.9386435	2.0	0.020145172	1.0162121
gb:BC018355.1 /DB_XREF=gi:17390829 /FEA=FLmRNA /CNT=80 /TID=Mm.23809.1 /TIER=FL+Stack /STK=27 /UG=Mm.23809 /LL=56748 /UG_GENE=Hirip5 /DEF=Mus musculus, histone cell cycle regulation defective interacting protein 5, clone MGC:5675 IMAGE:3584656, mRNA, complete cds. /PROD=histone cell cycle regulation defectiveinteracting protein 5 /FL=gb:NM_020045.1 gb:BC018355.1	3284.8333	224.78094	2328.8333	292.29797	4.490613	3.0	0.020605719	0.93549967
gb:AK002443.1 /DB_XREF=gi:12832431 /FEA=FLmRNA /CNT=204 /TID=Mm.180189.1 /TIER=Stack /STK=57 /UG=Mm.180189 /LL=67800 /UG_GENE=Dgat2 /UG_TITLE=diacylglycerol O-acyltransferase 2 /DEF=Mus musculus adult male kidney cDNA, RIKEN full-length enriched library, clone:0610010B06:homolog to WUGSC:H_DJ0747G18.5 PROTEIN (FRAGMENT), full insert sequence. /FL=gb:AF384160.1 gb:NM_026384.1	2753.4001	267.295	1639.7334	105.6523	6.7112303	2.0	0.021489117	0.88691443
gb:NM_007476.1 /DB_XREF=gi:6680715 /GEN=Arf1 /FEA=FLmRNA /CNT=64 /TID=Mm.6836.1 /TIER=FL /STK=2 /UG=Mm.6836 /LL=11840 /DEF=Mus musculus ADP-ribosylation factor 1 (Arf1), mRNA. /PROD=ADP-ribosylation factor 1 /FL=gb:D87898.1 gb:NM_007476.1	1804.6666	201.61327	1130.6666	170.99333	4.415943	3.0	0.021553561	0.81544304
gb:BC027445.1 /DB_XREF=gi:20071662 /FEA=FLmRNA /CNT=79 /TID=Mm.153891.1 /TIER=FL+Stack /STK=28 /UG=Mm.153891 /LL=19245 /UG_GENE=Ptp4a3 /DEF=Mus musculus, protein tyrosine phosphatase 4a3, clone MGC:36146 IMAGE:4482106, mRNA, complete cds. /PROD=protein tyrosine phosphatase 4a3 /FL=gb:NM_008975.1 gb:AF035645.1 gb:BC027445.1	3634.5999	385.0914	2027.2666	170.24298	6.6120944	2.0	0.022116944	0.77239174
gb:BC017689.1 /DB_XREF=gi:17389268 /FEA=FLmRNA /CNT=148 /TID=Mm.19258.1 /TIER=FL+Stack /STK=47 /UG=Mm.19258 /LL=66951 /UG_GENE=2310028D20Rik /DEF=Mus musculus, Similar to thyrotroph embryonic factor, clone MGC:19233 IMAGE:4242534, mRNA, complete cds. /PROD=Similar to thyrotroph embryonic factor /FL=gb:BC017689.1	2227.3333	188.9985	1486.5	238.61961	4.2153683	3.0	0.024394894	0.79109156
gb:AK003305.1 /DB_XREF=gi:12833890 /FEA=mRNA /CNT=31 /TID=Mm.46367.1 /TIER=Stack /STK=12 /UG=Mm.46367 /LL=68453 /UG_GENE=1110002J19Rik /UG_TITLE=RIKEN cDNA 1110002J19 gene /DEF=Mus musculus 18 days embryo whole body cDNA, RIKEN full-length enriched library, clone:1110002J19:unclassifiable, full insert sequence.	5818.1997	927.7161	3272.6667	519.24005	4.1471405	3.0	0.025470959	0.770921
gb:NM_011179.1 /DB_XREF=gi:7242190 /GEN=Psap /FEA=FLmRNA /CNT=332 /TID=Mm.3363.1 /TIER=FL /STK=1 /UG=Mm.3363 /LL=19156 /DEF=Mus musculus prosaposin (Psap), mRNA. /PROD=prosaposin /FL=gb:NM_011179.1	5425.933	829.29877	2215.7332	1085.7566	4.06974	3.0	0.026767017	0.75951415
gb:BC024138.1 /DB_XREF=gi:18848248 /FEA=FLmRNA /CNT=26 /TID=Mm.22926.1 /TIER=FL+Stack /STK=11 /UG=Mm.22926 /LL=66968 /UG_GENE=2310076L09Rik /DEF=Mus musculus, RIKEN cDNA 2310076L09 gene, clone MGC:37973 IMAGE:5136320, mRNA, complete cds. /PROD=RIKEN cDNA 2310076L09 gene /FL=gb:BC024138.1	1782.4	251.44804	1036.3667	201.14285	4.012932	3.0	0.027772743	0.7416956
gb:AF422244.1 /DB_XREF=gi:17026373 /GEN=Mdp77 /FEA=FLmRNA /CNT=35 /TID=Mm.33173.1 /TIER=FL /STK=4 /UG=Mm.33173 /LL=69506 /DEF=Mus musculus muscle-derived protein MDP77 variant 1 (Mdp77) mRNA, complete cds. /PROD=muscle-derived protein MDP77 variant 1 /FL=gb:AF422244.1 gb:NM_138628.1 gb:AF422245.1	5097.6665	429.47256	3562.9666	519.5502	3.9434373	3.0	0.029070232	0.73321587
gb:BC018323.1 /DB_XREF=gi:17390756 /FEA=FLmRNA /CNT=100 /TID=Mm.3459.1 /TIER=FL+Stack /STK=28 /UG=Mm.3459 /LL=13170 /UG_GENE=Dbp /DEF=Mus musculus, D site albumin promoter binding protein, clone MGC:18803 IMAGE:4195116, mRNA, complete cds. /PROD=D site albumin promoter binding protein /FL=gb:NM_016974.1 gb:BC018323.1	2181.5	125.08818	1068.4	314.64484	5.693912	2.0	0.029487085	0.70458615
gb:AF206720.1 /DB_XREF=gi:6636320 /FEA=FLmRNA /CNT=130 /TID=Mm.38469.1 /TIER=FL+Stack /STK=83 /UG=Mm.38469 /LL=11785 /UG_GENE=Apbb1 /DEF=Mus musculus Fe65 mRNA, complete cds. /PROD=Fe65 /FL=gb:AF206720.1	1659.7334	267.31207	867.3	232.41673	3.8747864	3.0	0.030429503	0.69074976
gb:BI408679 /DB_XREF=gi:15169602 /DB_XREF=602964304F1 /CLONE=IMAGE:5119896 /FEA=mRNA /CNT=61 /TID=Mm.29389.1 /TIER=Stack /STK=30 /UG=Mm.29389 /UG_TITLE=Mus musculus, Similar to KIAA1075 protein, clone IMAGE:5099327, mRNA, partial cds	1488.9	179.23257	810.2	246.92395	3.8527741	3.0	0.030882569	0.6676517
gb:NM_009983.1 /DB_XREF=gi:6753555 /GEN=Ctsd /FEA=FLmRNA /CNT=522 /TID=Mm.2147.1 /TIER=FL+Stack /STK=193 /UG=Mm.2147 /LL=13033 /DEF=Mus musculus cathepsin D (Ctsd), mRNA. /PROD=cathepsin D /FL=gb:NM_009983.1	5454.1333	777.9049	3241.0332	625.04315	3.8412483	3.0	0.031123256	0.6422708
"gb:U82470.1 /DB_XREF=gi:2690301 /GEN=Got-2 /FEA=FLmRNA /CNT=238 /TID=Mm.18916.1 /TIER=FL /STK=1 /UG=Mm.18916 /LL=14719 /DEF=Mus musculus aspartate aminotransferase precursor (Got-2) mRNA, Got-2-a allele, complete cds; nuclear gene for mitochondrial product. /PROD=aspartate aminotransferase precursor /FL=gb:U82470.1 gb:J02622.1 gb:NM_010325.1"	4684.267	741.73334	2668.0999	544.53687	3.795118	3.0	0.032111008	0.63384336
gb:AK003207.1 /DB_XREF=gi:12833731 /FEA=mRNA /CNT=179 /TID=Mm.29998.1 /TIER=Stack /STK=95 /UG=Mm.29998 /LL=66853 /UG_GENE=0610039C21Rik /UG_TITLE=RIKEN cDNA 0610039C21 gene /DEF=Mus musculus 18 days embryo whole body cDNA, RIKEN full-length enriched library, clone:1110001C14:similar to HYPOTHETICAL 19.8 KDA PROTEIN (FRAGMENT), full insert sequence.	4345.7	245.63489	2401.5999	592.12665	5.2527237	2.0	0.034385104	0.65045154
gb:BC003447.1 /DB_XREF=gi:13097416 /FEA=FLmRNA /CNT=395 /TID=Mm.12758.1 /TIER=FL+Stack /STK=94 /UG=Mm.12758 /LL=14228 /UG_GENE=Fkbp4 /DEF=Mus musculus, FK506 binding protein 4 (59 kDa), clone MGC:6528 IMAGE:2651490, mRNA, complete cds. /PROD=FK506 binding protein 4 (59 kDa) /FL=gb:NM_010219.1 gb:BC003447.1	1799.4	258.09253	969.4666	96.33808	5.217995	2.0	0.03482062	0.63234246
gb:AJ007909.1 /DB_XREF=gi:3336885 /GEN=edr /FEA=mRNA /CNT=7 /TID=Mm.35829.2 /TIER=ConsEnd /STK=5 /UG=Mm.35829 /LL=170942 /DEF=Mus musculus mRNA for erythroid differentiation regulator, partial. /PROD=erythroid differentiation regulator	3188.2668	328.84497	2022.2999	441.76965	3.6670012	3.0	0.03507333	0.61243427
gb:BC004613.1 /DB_XREF=gi:13435485 /FEA=FLmRNA /CNT=159 /TID=Mm.22597.1 /TIER=FL+Stack /STK=30 /UG=Mm.22597 /LL=69654 /UG_GENE=2310042E05Rik /DEF=Mus musculus, Similar to dynactin 2 (p50), clone MGC:6712 IMAGE:3585310, mRNA, complete cds. /PROD=Similar to dynactin 2 (p50) /FL=gb:BC004613.1	3519.4336	404.615	2197.7	185.29951	5.144202	2.0	0.035773426	0.6015235
gb:NM_023464.1 /DB_XREF=gi:12963686 /GEN=1110003H09Rik /FEA=FLmRNA /CNT=95 /TID=Mm.21444.1 /TIER=FL+Stack /STK=50 /UG=Mm.21444 /LL=68475 /DEF=Mus musculus RIKEN cDNA 1110003H09 gene (1110003H09Rik), mRNA. /PROD=RIKEN cDNA 1110003H09 /FL=gb:NM_023464.1	1704.7666	181.4112	1069.9333	244.86806	3.6081257	3.0	0.03655193	0.5926634
gb:NM_011390.1 /DB_XREF=gi:6755533 /GEN=Slc12a7 /FEA=FLmRNA /CNT=91 /TID=Mm.24510.1 /TIER=FL+Stack /STK=27 /UG=Mm.24510 /LL=20499 /DEF=Mus musculus solute carrier family 12, member 7 (Slc12a7), mRNA. /PROD=solute carrier family 12, member 7 /FL=gb:NM_011390.1 gb:AF087436.1	2386.3	263.8338	1715.7999	187.08417	3.5906699	3.0	0.037005566	0.57932854
gb:NM_024225.1 /DB_XREF=gi:18034768 /GEN=Snx5 /FEA=FLmRNA /CNT=386 /TID=Mm.20847.1 /TIER=FL+Stack /STK=39 /UG=Mm.20847 /LL=69178 /DEF=Mus musculus sorting nexin 5 (Snx5), mRNA. /PROD=sorting nexin 5 /FL=gb:NM_024225.1 gb:BC002242.1	1845.6333	502.8171	3112.2	355.3908	3.562837	3.0	0.037743855	0.57119036
gb:BC011344.1 /DB_XREF=gi:15030176 /FEA=FLmRNA /CNT=135 /TID=Mm.220921.2 /TIER=FL+Stack /STK=83 /UG=Mm.220921 /LL=15467 /UG_GENE=Eif2ak1 /DEF=Mus musculus, clone MGC:5739 IMAGE:3481870, mRNA, complete cds. /PROD=Unknown (protein for MGC:5739) /FL=gb:BC011344.1	1550.6333	267.75513	881.4333	195.2781	3.497545	3.0	0.039550956	0.5792301
gb:BC027346.1 /DB_XREF=gi:20071091 /FEA=FLmRNA /CNT=82 /TID=Mm.11545.1 /TIER=FL+Stack /STK=51 /UG=Mm.11545 /DEF=Mus musculus, clone MGC:28292 IMAGE:4011421, mRNA, complete cds. /PROD=Unknown (protein for MGC:28292) /FL=gb:BC027346.1	1823.9	194.57033	1146.9333	274.02765	3.4888892	3.0	0.039798737	0.5646446
gb:AK010167.1 /DB_XREF=gi:12845417 /GEN=Tcap /FEA=FLmRNA /CNT=56 /TID=Mm.10762.1 /TIER=Stack /STK=40 /UG=Mm.10762 /LL=21393 /UG_TITLE=titin-cap /DEF=Mus musculus adult male tongue cDNA, RIKEN full-length enriched library, clone:2310075E03:titin-cap, full insert sequence. /FL=gb:NM_011540.1	18152.068	2419.692	11988.667	1911.9014	3.4616609	3.0	0.040591177	0.5584362
gb:BC003975.1 /DB_XREF=gi:13278300 /FEA=FLmRNA /CNT=232 /TID=Mm.29737.1 /TIER=FL+Stack /STK=79 /UG=Mm.29737 /DEF=Mus musculus, Similar to myeloid leukemia factor 2, clone MGC:7424 IMAGE:3488851, mRNA, complete cds. /PROD=Similar to myeloid leukemia factor 2 /FL=gb:BC003975.1	2250.2666	502.99188	1128.7333	275.59164	3.3869345	3.0	0.042871576	0.5724616
gb:NM_007383.1 /DB_XREF=gi:6680619 /GEN=Acads /FEA=FLmRNA /CNT=113 /TID=Mm.18759.1 /TIER=FL+Stack /STK=35 /UG=Mm.18759 /LL=11409 /DEF=Mus musculus acetyl-Coenzyme A dehydrogenase, short chain (Acads), mRNA. /PROD=acetyl-Coenzyme A dehydrogenase, short chain /FL=gb:BC016259.1 gb:NM_007383.1 gb:L11163.1	2546.2332	583.8708	1251.8334	331.40588	3.3393998	3.0	0.044407293	0.576026
gb:AY028963.1 /DB_XREF=gi:19847823 /FEA=FLmRNA /CNT=8 /TID=Mm.38250.1 /TIER=FL /STK=1 /UG=Mm.38250 /LL=56490 /UG_GENE=Zfp288 /DEF=Mus musculus BTBPOZ zinc finger protein DPZF mRNA, complete cds. /PROD=BTBPOZ zinc finger protein DPZF /FL=gb:AF185576.1 gb:NM_019778.1 gb:AY028963.1	1758.9	300.57047	1063.5333	202.91864	3.3210924	3.0	0.04501735	0.5677188
gb:BC003744.1 /DB_XREF=gi:13277677 /FEA=FLmRNA /CNT=135 /TID=Mm.3294.1 /TIER=FL+Stack /STK=52 /UG=Mm.3294 /LL=19060 /UG_GENE=Ppp5c /DEF=Mus musculus, protein phosphatase 5, catalytic subunit, clone MGC:5847 IMAGE:3590322, mRNA, complete cds. /PROD=protein phosphatase 5, catalytic subunit /FL=gb:BC003744.1 gb:AF018262.1	1179.0	253.04428	629.6667	141.64098	3.2810674	3.0	0.046388563	0.5692002
gb:NM_007876.1 /DB_XREF=gi:6681216 /GEN=Dpep1 /FEA=FLmRNA /CNT=49 /TID=Mm.20388.1 /TIER=FL /STK=7 /UG=Mm.20388 /LL=13479 /DEF=Mus musculus dipeptidase 1 (renal) (Dpep1), mRNA. /PROD=dipeptidase 1 (renal) /FL=gb:NM_007876.1 gb:BC003492.1	2660.8667	501.4193	1360.6333	475.69363	3.25838	3.0	0.04718934	0.5637884
gb:BC015254.1 /DB_XREF=gi:15929639 /FEA=FLmRNA /CNT=72 /TID=Mm.6522.1 /TIER=FL+Stack /STK=40 /UG=Mm.6522 /LL=12778 /UG_GENE=Cmkor1 /DEF=Mus musculus, chemokine orphan receptor 1, clone MGC:18378 IMAGE:4242244, mRNA, complete cds. /PROD=chemokine orphan receptor 1 /FL=gb:BC015254.1 gb:NM_007722.1 gb:AF000236.1	3317.8333	177.26184	5048.1665	652.2934	4.433797	2.0	0.04728927	0.5504956
Mus musculus /REF=M32599 /DEF=Mouse glyceraldehyde-3-phosphate dehydrogenase mRNA, complete cds /LEN=1228 (_5, _M, _3 represent transcript regions 5 prime, Middle, and 3 prime respectively)	34215.402	7139.477	16254.234	6410.5503	3.2422204	3.0	0.0477704	0.54219407
gb:BC025440.1 /DB_XREF=gi:19343555 /FEA=FLmRNA /CNT=93 /TID=Mm.22668.2 /TIER=FL+Stack /STK=21 /UG=Mm.22668 /LL=15356 /UG_GENE=Hmgcl /DEF=Mus musculus, 3-hydroxy-3-methylglutaryl-Coenzyme A lyase, clone MGC:29242 IMAGE:5049759, mRNA, complete cds. /PROD=3-hydroxy-3-methylglutaryl-Coenzyme A lyase /FL=gb:BC025440.1	2477.4	257.76486	1629.1998	373.01678	3.2401416	3.0	0.047845803	0.52980477
gb:BC008273.1 /DB_XREF=gi:14198433 /FEA=FLmRNA /CNT=243 /TID=Mm.22514.1 /TIER=FL+Stack /STK=139 /UG=Mm.22514 /LL=66043 /UG_GENE=0610008F14Rik /DEF=Mus musculus, RIKEN cDNA 0610008F14 gene, clone MGC:6312 IMAGE:2811419, mRNA, complete cds. /PROD=RIKEN cDNA 0610008F14 gene /FL=gb:BC008273.1	8549.866	1705.65	4106.4336	406.7802	4.3891153	2.0	0.048188154	0.520891
gb:NM_008733.1 /DB_XREF=gi:6679123 /GEN=Nrap /FEA=FLmRNA /CNT=12 /TID=Mm.6384.1 /TIER=FL /STK=2 /UG=Mm.6384 /LL=18175 /DEF=Mus musculus nebulin-related anchoring protein (Nrap), mRNA. /PROD=nebulin-related anchoring protein /FL=gb:NM_008733.1 gb:U76618.1	4659.6	864.4257	2685.7666	622.2885	3.2097685	3.0	0.048964903	0.51697826

gb:AK012776.1 /DB_XREF=gi:12849742 /FEA=mRNA /CNT=22 /TID=Mm.154145.2 /TIER=ConsEnd /STK=6 /UG=Mm.154145 /LL=66376 /UG_GENE=2310011F05Rik /UG_TITLE=RIKEN cDNA 2310011F05 gene /DEF=Mus musculus 10, 11 days embryo whole body cDNA, RIKEN full-length enriched library, clone:2810021G02:Zinc finger, C2H2 type containing protein, full insert sequence.	550.86664	266.142	1449.6334	410.57803	3.1815608	3.0	0.05003401	0.51625997
gb:AF071315.1 /DB_XREF=gi:3309171 /GEN=COPS6 /FEA=FLmRNA /CNT=115 /TID=Mm.3981.1 /TIER=FL+Stack /STK=29 /UG=Mm.3981 /LL=26893 /DEF=Mus musculus COP9 complex subunit 6 (COPS6) mRNA, complete cds. /PROD=COP9 complex subunit 6 /FL=gb:AF071315.1 gb:BC014286.1 gb:BC004664.1	4076.3333	382.2838	2798.6667	588.63916	3.1529322	3.0	0.05114939	0.5160405
gb:NM_013562.1 /DB_XREF=gi:7305178 /GEN=Ifrd1 /FEA=FLmRNA /CNT=225 /TID=Mm.168.1 /TIER=FL+Stack /STK=134 /UG=Mm.168 /LL=15982 /DEF=Mus musculus interferon-related developmental regulator 1 (Ifrd1), mRNA. /PROD=interferon-related developmental regulator 1 /FL=gb:NM_013562.1	1775.8002	335.17688	2668.5	362.82657	3.1302788	3.0	0.052054286	0.5137532
gb:NM_019502.1 /DB_XREF=gi:9507186 /GEN=Fxc1 /FEA=FLmRNA /CNT=70 /TID=Mm.4050.1 /TIER=FL+Stack /STK=33 /UG=Mm.4050 /LL=14356 /DEF=Mus musculus fractured callus expressed transcript 1 (Fxc1), mRNA. /PROD=fractured callus expressed transcript 1 /FL=gb:NM_019502.1 gb:AF150103.1 gb:BC009158.1	1996.7001	257.0728	1280.0667	302.79193	3.1249733	3.0	0.052269127	0.50489753
gb:NM_026252.1 /DB_XREF=gi:13385759 /GEN=4930447D24Rik /FEA=FLmRNA /CNT=161 /TID=Mm.23178.1 /TIER=ConsEnd /STK=0 /UG=Mm.23178 /LL=67579 /DEF=Mus musculus RIKEN cDNA 4930447D24 gene (4930447D24Rik), mRNA. /PROD=RIKEN cDNA 4930447D24 /FL=gb:NM_026252.1	2246.3333	522.5565	3440.5332	407.56915	3.12117	3.0	0.052423827	0.495842
gb:U20344.1 /DB_XREF=gi:912487 /FEA=FLmRNA /CNT=140 /TID=Mm.4325.1 /TIER=FL+Stack /STK=46 /UG=Mm.4325 /LL=16600 /UG_GENE=Klf4 /DEF=Mus musculus gut-enriched Kruppel-like factor GKLF mRNA, complete cds. /PROD=gut-enriched Kruppel-like factor /FL=gb:BC010301.1 gb:U20344.1 gb:U70662.1 gb:NM_010637.1	2218.3	658.10254	3810.5332	593.3005	3.1124587	3.0	0.05278034	0.48902604
gb:BC011116.1 /DB_XREF=gi:15029789 /FEA=FLmRNA /CNT=246 /TID=Mm.104975.1 /TIER=FL+Stack /STK=90 /UG=Mm.104975 /LL=66905 /UG_GENE=1300012C15Rik /DEF=Mus musculus, RIKEN cDNA 1300012C15 gene, clone MGC:19018 IMAGE:4023354, mRNA, complete cds. /PROD=RIKEN cDNA 1300012C15 gene /FL=gb:NM_025836.1 gb:BC011116.1	1721.4332	308.0201	978.2334	88.368774	4.017092	2.0	0.056745864	0.5152525
gb:NM_016715.1 /DB_XREF=gi:7710051 /GEN=Tslpr /FEA=FLmRNA /CNT=43 /TID=Mm.35771.1 /TIER=FL+Stack /STK=30 /UG=Mm.35771 /LL=57914 /DEF=Mus musculus thymic stromal-derived lymphopoietin, receptor (Tslpr), mRNA. /PROD=thymic stromal-derived lymphopoietin, receptor /FL=gb:AF232936.1 gb:AB031333.1 gb:AB039945.1 gb:NM_016715.1	912.63336	302.9743	1570.6333	225.301	3.0185394	3.0	0.05682495	0.5058535
gb:BC016260.1 /DB_XREF=gi:16740779 /FEA=FLmRNA /CNT=210 /TID=Mm.19055.1 /TIER=FL+Stack /STK=103 /UG=Mm.19055 /LL=68877 /UG_GENE=1110068E11Rik /DEF=Mus musculus, RIKEN cDNA 1110068E11 gene, clone MGC:28902 IMAGE:4914167, mRNA, complete cds. /PROD=RIKEN cDNA 1110068E11 gene /FL=gb:BC016260.1	2465.0332	287.6765	1808.0668	243.65489	3.018338	3.0	0.056834035	0.49620482
gb:NM_133734.1 /DB_XREF=gi:19526929 /GEN=0710008A13Rik /FEA=FLmRNA /CNT=134 /TID=Mm.11535.1 /TIER=FL+Stack /STK=76 /UG=Mm.11535 /LL=71700 /DEF=Mus musculus RIKEN cDNA 0710008A13 gene (0710008A13Rik), mRNA. /PROD=RIKEN cDNA 0710008A13 /FL=gb:NM_133734.1 gb:BC008545.1	2924.9336	463.44464	1932.3334	332.68933	3.0135894	3.0	0.057048753	0.4886818
gb:BC019459.1 /DB_XREF=gi:18043385 /FEA=mRNA /CNT=128 /TID=Mm.31025.1 /TIER=Stack /STK=66 /UG=Mm.31025 /LL=76437 /UG_GENE=2310015K15Rik /UG_TITLE=RIKEN cDNA 2310015K15 gene /DEF=Mus musculus, Similar to RIKEN cDNA 2310015K15 gene, clone IMAGE:4035590, mRNA.	682.73334	297.8792	1340.9333	235.16748	3.003885	3.0	0.057490736	0.48334804
gb:BC010596.1 /DB_XREF=gi:14714881 /FEA=FLmRNA /CNT=95 /TID=Mm.196239.1 /TIER=FL+Stack /STK=41 /UG=Mm.196239 /LL=66734 /UG_GENE=4922501H04Rik /DEF=Mus musculus, MAP1 light chain 3-like protein 1, clone MGC:6325 IMAGE:3256801, mRNA, complete cds. /PROD=MAP1 light chain 3-like protein 1 /FL=gb:BC010596.1	8346.8	1826.1411	4220.5	181.96098	3.8944106	2.0	0.060056616	0.49574006
gb:AB006361.1 /DB_XREF=gi:2317285 /FEA=FLmRNA /CNT=130 /TID=Mm.1008.1 /TIER=FL+Stack /STK=89 /UG=Mm.1008 /LL=19215 /UG_GENE=Ptgds /DEF=Mus musculus mRNA for prostaglandin D synthetase, complete cds. /PROD=prostaglandin D synthetase /FL=gb:AB006361.1	5255.2	1303.1003	2508.8	978.30804	2.9193041	3.0	0.06153079	0.4988389
gb:NM_008831.1 /DB_XREF=gi:6679298 /GEN=Phb /FEA=FLmRNA /CNT=260 /TID=Mm.2355.1 /TIER=FL+Stack /STK=58 /UG=Mm.2355 /LL=18673 /DEF=Mus musculus prohibitin (Phb), mRNA. /PROD=prohibitin /FL=gb:NM_008831.1	6689.0664	1162.0282	4307.933	824.85596	2.894155	3.0	0.06280016	0.50019777
gb:NM_013692.1 /DB_XREF=gi:7305570 /GEN=Tieg /FEA=FLmRNA /CNT=184 /TID=Mm.4292.1 /TIER=FL+Stack /STK=141 /UG=Mm.4292 /LL=21847 /DEF=Mus musculus TGFB inducible early growth response (Tieg), mRNA. /PROD=TGFB inducible early growth response /FL=gb:AF064088.1 gb:BC003316.1 gb:NM_013692.1 gb:AF049879.1	528.2	266.4009	1155.1333	107.51492	3.7798882	2.0	0.06340678	0.49632204
gb:BC010817.1 /DB_XREF=gi:14789892 /FEA=FLmRNA /CNT=138 /TID=Mm.3979.1 /TIER=FL+Stack /STK=25 /UG=Mm.3979 /DEF=Mus musculus, Similar to ubiquitin-like 4, clone MGC:19132 IMAGE:4215699, mRNA, complete cds. /PROD=Similar to ubiquitin-like 4 /FL=gb:BC010817.1	1884.5333	340.92563	1204.4667	226.77893	2.8767273	3.0	0.063699044	0.49015874
gb:BC010592.1 /DB_XREF=gi:14714874 /FEA=FLmRNA /CNT=119 /TID=Mm.29553.1 /TIER=FL+Stack /STK=53 /UG=Mm.29553 /LL=66071 /UG_GENE=0610025L15Rik /DEF=Mus musculus, RIKEN cDNA 0610025L15 gene, clone MGC:7046 IMAGE:3156338, mRNA, complete cds. /PROD=RIKEN cDNA 0610025L15 gene /FL=gb:BC010592.1 gb:AB049623.1 gb:NM_023154.1	1405.4667	213.8315	834.73334	270.21564	2.8687627	3.0	0.06411519	0.48513827
gb:NM_007408.1 /DB_XREF=gi:6680649 /GEN=Adfp /FEA=FLmRNA /CNT=309 /TID=Mm.381.1 /TIER=FL+Stack /STK=92 /UG=Mm.381 /LL=11520 /DEF=Mus musculus adipose differentiation related protein (Adfp), mRNA. /PROD=adipose differentiation related protein /FL=gb:M93275.1 gb:NM_007408.1	2498.2666	257.47202	1771.2334	357.4763	2.8584013	3.0	0.06466167	0.4812524
gb:BI076714 /DB_XREF=gi:14515371 /DB_XREF=L0223B10-3 /CLONE=L0223B10 /FEA=mRNA /CNT=167 /TID=Mm.216195.1 /TIER=Stack /STK=18 /UG=Mm.216195 /UG_TITLE=Mus musculus mVL30-1 retroelement mRNA sequence	12693.434	2625.5188	6953.3003	530.13385	3.7118473	2.0	0.065527044	0.47982705
gb:AK003526.1 /DB_XREF=gi:12834245 /FEA=mRNA /CNT=4 /TID=Mm.3336.1 /TIER=ConsEnd /STK=0 /UG=Mm.3336 /LL=68480 /UG_GENE=1110007C09Rik /UG_TITLE=RIKEN cDNA 1110007C09 gene /DEF=Mus musculus 18 days embryo whole body cDNA, RIKEN full-length enriched library, clone:1110007C09:hypothetical protein, full insert sequence.	1536.2666	142.37112	866.23334	279.45187	3.7003376	2.0	0.06589585	0.47486848
gb:NM_009150.1 /DB_XREF=gi:6677906 /GEN=Selenbp1 /FEA=FLmRNA /CNT=79 /TID=Mm.636.1 /TIER=FL+Stack /STK=22 /UG=Mm.636 /LL=20341 /DEF=Mus musculus selenium binding protein 1 (Selenbp1), mRNA. /PROD=selenium binding protein 1 /FL=gb:NM_009150.1	3233.7	409.58347	2182.2666	504.01556	2.8041036	3.0	0.06762234	0.479696
gb:NM_009091.1 /DB_XREF=gi:6677798 /GEN=Rps15 /FEA=FLmRNA /CNT=294 /TID=Mm.643.1 /TIER=FL+Stack /STK=129 /UG=Mm.643 /LL=20054 /DEF=Mus musculus ribosomal protein S15 (Rps15), mRNA. /PROD=ribosomal protein S15 /FL=gb:M33330.1 gb:NM_009091.1 gb:BC010763.1	23885.0	3503.3015	16750.367	2677.3313	2.8026602	3.0	0.06770333	0.47288173
gb:L40934.1 /DB_XREF=gi:722666 /GEN=rab6 /FEA=mRNA /CNT=1 /TID=Mm.22473.2 /TIER=ConsEnd /STK=0 /UG=Mm.22473 /LL=14470 /DEF=Mus musculus rab6rab5-associated protein (rab6) mRNA, partial cds. /PROD=rab6rab5-associated protein	4004.6335	935.89264	1930.6666	393.83847	3.5377922	2.0	0.071441576	0.49143144
gb:AK003388.1 /DB_XREF=gi:12834026 /FEA=mRNA /CNT=32 /TID=Mm.41330.1 /TIER=Stack /STK=17 /UG=Mm.41330 /LL=76291 /UG_GENE=1110003O08Rik /UG_TITLE=RIKEN cDNA 1110003O08 gene /DEF=Mus musculus 18 days embryo whole body cDNA, RIKEN full-length enriched library, clone:1110003O08:hypothetical protein, full insert sequence.	1215.3334	282.99225	629.76666	51.017086	3.5270977	2.0	0.07182983	0.48672754
gb:NM_016738.1 /DB_XREF=gi:7949126 /GEN=Rpl13 /FEA=FLmRNA /CNT=11 /TID=Mm.42578.1 /TIER=FL /STK=2 /UG=Mm.42578 /LL=19898 /DEF=Mus musculus ribosomal protein L13 (Rpl13), mRNA. /PROD=ribosomal protein L13 /FL=gb:U28917.1 gb:NM_016738.1	19578.768	4562.7007	10923.566	3138.9824	2.7068903	3.0	0.07335706	0.48976624
gb:NM_025570.1 /DB_XREF=gi:20270193 /GEN=Mrpl20 /FEA=FLmRNA /CNT=181 /TID=Mm.23825.1 /TIER=FL+Stack /STK=128 /UG=Mm.23825 /LL=66448 /DEF=Mus musculus mitochondrial ribosomal protein L20 (Mrpl20), mRNA. /PROD=mitochondrial ribosomal protein L20 /FL=gb:AB049645.1 gb:BC016591.1 gb:NM_025570.1	3240.5664	473.0782	2285.8	388.91672	2.700274	3.0	0.07376892	0.48537812
gb:NM_133721.1 /DB_XREF=gi:19526903 /GEN=Itga9 /FEA=FLmRNA /CNT=110 /TID=Mm.23386.1 /TIER=FL /STK=2 /UG=Mm.23386 /LL=70107 /DEF=Mus musculus integrin alpha 9 (Itga9), mRNA. /PROD=integrin alpha 9 /FL=gb:NM_133721.1	1774.6666	433.60614	2705.9	413.57755	2.691753	3.0	0.074303575	0.48191175
gb:NM_016878.1 /DB_XREF=gi:8393267 /GEN=Dnpep /FEA=FLmRNA /CNT=197 /TID=Mm.24680.1 /TIER=FL+Stack /STK=123 /UG=Mm.24680 /LL=13437 /DEF=Mus musculus aspartyl aminopeptidase (Dnpep), mRNA. /PROD=aspartyl aminopeptidase /FL=gb:AF005051.1 gb:NM_016878.1	1693.6334	243.04478	1160.1666	242.70084	2.6901348	3.0	0.07440565	0.47577694
gb:NM_053162.1 /DB_XREF=gi:16716448 /GEN=Mrpl34 /FEA=FLmRNA /CNT=111 /TID=Mm.29489.1 /TIER=FL+Stack /STK=96 /UG=Mm.29489 /LL=94065 /DEF=Mus musculus mitochondrial ribosomal protein L34 (Mrpl34), mRNA. /PROD=mitochondrial ribosomal protein L34 /FL=gb:NM_053162.1 gb:AB049653.1	2390.8	640.9675	1195.1666	431.3994	2.6803503	3.0	0.07502657	0.47308424
gb:BC003744.1 /DB_XREF=gi:13277677 /FEA=FLmRNA /CNT=135 /TID=Mm.3294.1 /TIER=FL+Stack /STK=52 /UG=Mm.3294 /LL=19060 /UG_GENE=Ppp5c /DEF=Mus musculus, protein phosphatase 5, catalytic subunit, clone MGC:5847 IMAGE:3590322, mRNA, complete cds. /PROD=protein phosphatase 5, catalytic subunit /FL=gb:BC003744.1 gb:AF018262.1	974.39996	279.2426	455.9	185.42218	2.6792161	3.0	0.075098954	0.46705377
gb:AF078667.1 /DB_XREF=gi:4322307 /FEA=FLmRNA /CNT=4 /TID=Mm.29030.2 /TIER=FL /STK=1 /UG=Mm.29030 /LL=20411 /UG_GENE=Sorbs1 /DEF=Mus musculus ponsin-1 mRNA, complete cds. /PROD=ponsin-1 /FL=gb:AF078667.1	4500.3335	454.3644	2470.4	926.83795	3.4062045	2.0	0.07643881	0.46896243
gb:NM_019990.1 /DB_XREF=gi:9910481 /GEN=Sdccag28 /FEA=FLmRNA /CNT=125 /TID=Mm.28896.1 /TIER=FL+Stack /STK=29 /UG=Mm.28896 /LL=56018 /DEF=Mus musculus serologically defined colon cancer antigen 28 (Sdccag28), mRNA. /PROD=serologically defined colon cancer antigen 28 /FL=gb:AB031550.1 gb:NM_019990.1	2981.7666	774.46027	1355.2666	307.86032	3.380319	2.0	0.0774809	0.4690177
gb:NM_011875.1 /DB_XREF=gi:6755209 /GEN=Psmd13 /FEA=FLmRNA /CNT=192 /TID=Mm.29760.1 /TIER=FL+Stack /STK=95 /UG=Mm.29760 /LL=23997 /DEF=Mus musculus proteasome (prosome, macropain) 26S subunit, non-ATPase, 13 (Psmd13), mRNA. /PROD=proteasome (prosome, macropain) 26S subunit,non-ATPase, 13 /FL=gb:NM_011875.1 gb:AF107838.1	1570.9667	226.58565	1053.6	253.27943	2.6368415	3.0	0.07786655	0.46515015
gb:BC024394.1 /DB_XREF=gi:19354424 /FEA=FLmRNA /CNT=200 /TID=Mm.34570.2 /TIER=FL+Stack /STK=106 /UG=Mm.34570 /LL=20018 /UG_GENE=Rpo1-3 /DEF=Mus musculus, RNA polymerase 1-3 (16 kDa subunit), clone MGC:36184 IMAGE:4987975, mRNA, complete cds. /PROD=RNA polymerase 1-3 (16 kDa subunit) /FL=gb:BC024394.1	1394.3667	266.72238	763.7	316.98685	2.6367848	3.0	0.07787033	0.45913157
gb:NM_007638.1 /DB_XREF=gi:6671703 /GEN=Cct7 /FEA=FLmRNA /CNT=513 /TID=Mm.914.1 /TIER=FL+Stack /STK=236 /UG=Mm.914 /LL=12468 /DEF=Mus musculus chaperonin subunit 7 (eta) (Cct7), mRNA. /PROD=chaperonin subunit 7 (eta) /FL=gb:BC008255.1 gb:NM_007638.1	2315.9333	46.717625	1610.0668	360.1172	3.3667843	2.0	0.07803395	0.4541976
gb:BC013486.1 /DB_XREF=gi:15488676 /FEA=FLmRNA /CNT=189 /TID=Mm.41389.1 /TIER=FL+Stack /STK=141 /UG=Mm.41389 /LL=66277 /UG_GENE=Klf15 /DEF=Mus musculus, Kruppel-like factor 15, clone MGC:19125 IMAGE:4211067, mRNA, complete cds. /PROD=Kruppel-like factor 15 /FL=gb:NM_023184.1 gb:AF317225.1 gb:BC013486.1	1641.1333	293.1515	993.6667	309.8631	2.6290479	3.0	0.07838921	0.45048988
gb:NM_009777.1 /DB_XREF=gi:6753219 /GEN=C1qb /FEA=FLmRNA /CNT=149 /TID=Mm.2570.1 /TIER=FL+Stack /STK=58 /UG=Mm.2570 /LL=12260 /DEF=Mus musculus complement component 1, q subcomponent, beta polypeptide (C1qb), mRNA. /PROD=complement component 1, q subcomponent, betapolypeptide /FL=gb:M22531.1 gb:M36293.1 gb:NM_009777.1	2006.4667	304.08218	1285.4332	365.01932	2.6287265	3.0	0.078410864	0.44498166
gb:NM_023371.1 /DB_XREF=gi:12963652 /GEN=Pin1 /FEA=FLmRNA /CNT=173 /TID=Mm.7906.1 /TIER=FL+Stack /STK=110 /UG=Mm.7906 /LL=23988 /DEF=Mus musculus protein (peptidyl-prolyl cistrans isomerase) NIMA-interacting 1 (Pin1), mRNA. /PROD=protein (peptidyl-prolyl cistrans isomerase)NIMA-interacting 1 /FL=gb:NM_023371.1	3336.7666	745.0641	1999.5333	480.16516	2.613035	3.0	0.07947676	0.44546235
gb:NM_008155.1 /DB_XREF=gi:6680066 /GEN=Gpi1 /FEA=FLmRNA /CNT=26 /TID=Mm.589.1 /TIER=FL /STK=2 /UG=Mm.589 /LL=14751 /DEF=Mus musculus glucose phosphate isomerase 1 complex (Gpi1), mRNA. /PROD=glucose phosphate isomerase 1 complex /FL=gb:M14220.1 gb:NM_008155.1	6017.467	1636.3217	3145.5999	981.9437	2.6065686	3.0	0.07992121	0.4424906
gb:U48800.1 /DB_XREF=gi:1234824 /GEN=VEGF-B /FEA=FLmRNA /CNT=7 /TID=Mm.15607.2 /TIER=FL /STK=1 /UG=Mm.15607 /LL=22340 /DEF=Mus musculus vascular endothelial growth factor B precursor (VEGF-B) mRNA, complete cds. /PROD=vascular endothelial growth factor B precursor /FL=gb:U43837.1 gb:U48800.1	3644.2334	921.23413	1713.7333	417.65958	3.3057415	2.0	0.08060011	0.4408729
gb:AK010619.1 /DB_XREF=gi:12846191 /GEN=Phb /FEA=FLmRNA /CNT=260 /TID=Mm.2355.1 /TIER=Stack /STK=55 /UG=Mm.2355 /LL=18673 /UG_TITLE=prohibitin /DEF=Mus musculus ES cells cDNA, RIKEN full-length enriched library, clone:2410030I08:prohibitin, full insert sequence. /FL=gb:NM_008831.1	3106.8665	494.61856	2189.1	362.6443	2.5918353	3.0	0.080945365	0.43749043
gb:NM_130881.1 /DB_XREF=gi:18700019 /GEN=MGC6685 /FEA=FLmRNA /CNT=43 /TID=Mm.12267.1 /TIER=FL /STK=2 /UG=Mm.12267 /LL=170715 /DEF=Mus musculus similar to poly(A)-binding protein, cytoplasmic 4 (inducible form) (MGC6685), mRNA. /PROD=similar to poly(A)-binding protein, cytoplasmic4 (inducible form) /FL=gb:NM_130881.1 gb:BC003283.1	2363.5667	555.67523	1319.0	424.0053	2.5884504	3.0	0.081182934	0.43361238
gb:NM_053082.1 /DB_XREF=gi:16716364 /GEN=Tm4sf7 /FEA=FLmRNA /CNT=147 /TID=Mm.195498.1 /TIER=FL+Stack /STK=105 /UG=Mm.195498 /LL=20524 /DEF=Mus musculus transmembrane 4 superfamily member 7 (Tm4sf7), mRNA. /PROD=transmembrane 4 superfamily member 7 /FL=gb:NM_053082.1 gb:BC003482.1	1432.9668	371.01614	723.1333	83.90889	3.2321563	2.0	0.083857775	0.44269103
gb:NM_015816.1 /DB_XREF=gi:7657316 /GEN=Lsm4-pending /FEA=FLmRNA /CNT=133 /TID=Mm.14585.1 /TIER=FL+Stack /STK=52 /UG=Mm.14585 /LL=50783 /DEF=Mus musculus U6 snRNA-associated SM-like protein 4 (Lsm4-pending), mRNA. /PROD=U6 snRNA-associated SM-like protein 4 /FL=gb:NM_015816.1 gb:BC026747.1	2082.0667	419.71677	1322.8334	300.10544	2.5486553	3.0	0.08404149	0.43856132
gb:NM_007940.1 /DB_XREF=gi:6679664 /GEN=Ephx2 /FEA=FLmRNA /CNT=154 /TID=Mm.15295.1 /TIER=FL+Stack /STK=66 /UG=Mm.15295 /LL=13850 /DEF=Mus musculus epoxide hydrolase 2, cytoplasmic (Ephx2), mRNA. /PROD=epoxide hydrolase 2, cytoplasmic /FL=gb:BC015087.1 gb:L05781.1 gb:NM_007940.1	6488.4336	906.62756	4647.6	401.75757	3.2152436	2.0	0.08463317	0.43663022
gb:NM_009045.1 /DB_XREF=gi:6677708 /GEN=Rela /FEA=FLmRNA /CNT=10 /TID=Mm.28170.1 /TIER=FL+Stack /STK=9 /UG=Mm.28170 /LL=19697 /DEF=Mus musculus v-rel reticuloendotheliosis viral oncogene homolog A, (avian) (Rela), mRNA. /PROD=avian reticuloendotheliosis viral (v-rel)oncogene homolog A /FL=gb:NM_009045.1 gb:M61909.1	1960.0332	383.5544	1213.4333	334.97433	2.5393877	3.0	0.08472489	0.43219215
gb:NM_019503.1 /DB_XREF=gi:9506704 /GEN=Fxyd1 /FEA=FLmRNA /CNT=7 /TID=Mm.1491.1 /TIER=FL /STK=2 /UG=Mm.1491 /LL=56188 /DEF=Mus musculus FXYD domain-containing ion transport regulator 1 (Fxyd1), mRNA. /PROD=FXYD domain-containing ion transport regulator1 /FL=gb:AF089734.1 gb:NM_019503.1	14909.3	2605.9375	10422.3	1641.9779	2.5232031	3.0	0.0859348	0.43349335
gb:NM_011125.1 /DB_XREF=gi:6755111 /GEN=Pltp /FEA=FLmRNA /CNT=67 /TID=Mm.6105.1 /TIER=FL+Stack /STK=32 /UG=Mm.6105 /LL=18830 /DEF=Mus musculus phospholipid transfer protein (Pltp), mRNA. /PROD=phospholipid transfer protein /FL=gb:BC003782.1 gb:NM_011125.1 gb:U37226.1 gb:U28960.1	2012.7001	384.84665	1234.1333	370.92523	2.5229409	3.0	0.085954584	0.42882836

gb:NM_008155.1 /DB_XREF=gi:6680066 /GEN=Gpi1 /FEA=FLmRNA /CNT=26 /TID=Mm.589.1 /TIER=FL /STK=2 /UG=Mm.589 /LL=14751 /DEF=Mus musculus glucose phosphate isomerase 1 complex (Gpi1), mRNA. /PROD=glucose phosphate isomerase 1 complex /FL=gb:M14220.1 gb:NM_008155.1	8020.0	2118.5437	4423.6665	1272.875	2.5203187	3.0	0.08615266	0.42514464
gb:NM_024223.1 /DB_XREF=gi:13195645 /GEN=0610010I23Rik /FEA=FLmRNA /CNT=180 /TID=Mm.133825.1 /TIER=FL+Stack /STK=48 /UG=Mm.133825 /LL=68337 /DEF=Mus musculus RIKEN cDNA 0610010I23 gene (0610010I23Rik), mRNA. /PROD=RIKEN cDNA 0610010I23 /FL=gb:NM_024223.1 gb:BC002096.1 gb:BC002093.1	24228.334	3708.0068	16887.434	3422.41	2.519776	3.0	0.08619372	0.42077363
gb:NM_025529.1 /DB_XREF=gi:13384949 /GEN=2310039H17Rik /FEA=FLmRNA /CNT=18 /TID=Mm.44092.1 /TIER=FL /STK=2 /UG=Mm.44092 /LL=66387 /DEF=Mus musculus RIKEN cDNA 2310039H17 gene (2310039H17Rik), mRNA. /PROD=RIKEN cDNA 2310039H17 /FL=gb:NM_025529.1	3700.1335	1125.2148	1606.6998	191.09187	3.1769488	2.0	0.08642728	0.41742536
gb:AB054000.1 /DB_XREF=gi:14017772 /GEN=Cg10671-like /FEA=FLmRNA /CNT=35 /TID=Mm.36758.1 /TIER=FL+Stack /STK=15 /UG=Mm.36758 /LL=68680 /DEF=Mus musculus Cg10671-like mRNA, complete cds. /PROD=Cg10671-like /FL=gb:AB054000.1	1886.9332	390.19482	1093.9	189.40694	3.1668425	2.0	0.086909845	0.41533756
gb:BC024621.1 /DB_XREF=gi:19354257 /FEA=FLmRNA /CNT=208 /TID=Mm.30064.1 /TIER=FL+Stack /STK=62 /UG=Mm.30064 /LL=56486 /UG_GENE=Gabarap /DEF=Mus musculus, GABA(A) receptor-associated protein, clone MGC:25654 IMAGE:4459651, mRNA, complete cds. /PROD=GABA(A) receptor-associated protein /FL=gb:NM_019749.1 gb:BC002126.1 gb:BC024621.1 gb:AF161587.1	10245.833	2222.5093	5753.9663	1048.8822	3.165773	2.0	0.086961135	0.4112537
gb:AF250139.1 /DB_XREF=gi:7644381 /FEA=FLmRNA /CNT=159 /TID=Mm.21549.1 /TIER=FL /STK=1 /UG=Mm.21549 /LL=80888 /UG_GENE=Cryac /DEF=Mus musculus small stress protein-like protein HSP22 mRNA, complete cds. /PROD=small stress protein-like protein HSP22 /FL=gb:BC011219.1 gb:NM_030704.1 gb:AF250139.1 gb:AF273453.1	2011.4	564.6096	1067.7667	328.73923	2.501638	3.0	0.08758011	0.40991104
gb:NM_019414.1 /DB_XREF=gi:9507078 /GEN=Selenbp2 /FEA=FLmRNA /CNT=105 /TID=Mm.196558.1 /TIER=FL+Stack /STK=41 /UG=Mm.196558 /LL=20342 /DEF=Mus musculus selenium binding protein 2 (Selenbp2), mRNA. /PROD=selenium binding protein 2 /FL=gb:NM_019414.1 gb:BC024106.1 gb:BC011202.1	4026.9336	494.99008	2721.0667	759.7327	2.4944136	3.0	0.088139914	0.40832165
gb:NM_024174.1 /DB_XREF=gi:15022814 /GEN=Mrps23 /FEA=FLmRNA /CNT=43 /TID=Mm.41910.1 /TIER=FL /STK=1 /UG=Mm.41910 /LL=64656 /DEF=Mus musculus mitochondrial ribosomal protein S23 (Mrps23), mRNA. /PROD=mitochondrial ribosomal protein S23 /FL=gb:NM_024174.1	2356.1335	299.46243	1698.9668	349.67368	2.4724076	3.0	0.08987236	0.41214195
gb:AK013135.1 /DB_XREF=gi:12850309 /FEA=mRNA /CNT=121 /TID=Mm.141864.1 /TIER=Stack /STK=79 /UG=Mm.141864 /LL=75620 /UG_GENE=2810422J05Rik /UG_TITLE=RIKEN cDNA 2810422J05 gene /DEF=Mus musculus 10, 11 days embryo whole body cDNA, RIKEN full-length enriched library, clone:2810422J05:homolog to R26445_1 (FRAGMENT), full insert sequence.	1843.7001	200.65141	1282.5333	344.3946	2.4385602	3.0	0.09261915	0.42049095
Mus musculus /REF=M32599 /DEF=Mouse glyceraldehyde-3-phosphate dehydrogenase mRNA, complete cds /LEN=1228 (_5, _M, _3 represent transcript regions 5 prime, Middle, and 3 prime respectively)	38164.9	9613.984	22698.932	5409.2744	2.4283547	3.0	0.093467414	0.42014065
gb:NM_025554.1 /DB_XREF=gi:13384981 /GEN=Polr2e /FEA=FLmRNA /CNT=89 /TID=Mm.18579.1 /TIER=FL+Stack /STK=55 /UG=Mm.18579 /LL=66420 /DEF=Mus musculus polymerase (RNA) II (DNA directed) polypeptide E (25kD) (Polr2e), mRNA. /PROD=polymerase (RNA) II (DNA directed) polypeptide E(25kD) /FL=gb:NM_025554.1	3954.4001	400.78058	2848.5667	680.5213	2.4252167	3.0	0.09373015	0.41719106
gb:NM_017393.1 /DB_XREF=gi:8393155 /GEN=Clpp /FEA=FLmRNA /CNT=134 /TID=Mm.15243.1 /TIER=FL+Stack /STK=78 /UG=Mm.15243 /LL=53895 /DEF=Mus musculus caseinolytic protease, ATP-dependent, proteolytic subunit homolog (E. coli) (Clpp), mRNA. /PROD=caseinolytic protease, ATP-dependent,proteolytic subunit homolog (E. coli) /FL=gb:NM_017393.1 gb:BC001998.1	1832.3666	349.50354	1118.9667	371.0742	2.4240038	3.0	0.09383194	0.41358933
gb:BC019420.1 /DB_XREF=gi:18043845 /FEA=FLmRNA /CNT=96 /TID=Mm.46654.1 /TIER=FL+Stack /STK=59 /UG=Mm.46654 /DEF=Mus musculus, Similar to chromosome 6 open reading frame 35, clone MGC:30293 IMAGE:5053733, mRNA, complete cds. /PROD=Similar to chromosome 6 open reading frame 35 /FL=gb:BC019420.1	1815.1333	453.52466	927.93335	445.7683	2.416461	3.0	0.09446801	0.4123892
gb:NM_031868.1 /DB_XREF=gi:13994194 /GEN=Ppp1ca /FEA=FLmRNA /CNT=386 /TID=Mm.1970.1 /TIER=FL+Stack /STK=206 /UG=Mm.1970 /LL=19045 /DEF=Mus musculus protein phosphatase 1, catalytic subunit, alpha isoform (Ppp1ca), mRNA. /PROD=protein phosphatase 1, catalytic subunit, alphaisoform /FL=gb:U25809.1 gb:NM_031868.1 gb:BC014828.1	5705.3667	1490.353	3345.9333	800.3353	2.4157786	3.0	0.09452581	0.40871158
gb:AK009753.1 /DB_XREF=gi:12844739 /FEA=mRNA /CNT=8 /TID=Mm.34838.1 /TIER=ConsEnd /STK=3 /UG=Mm.34838 /LL=76463 /UG_GENE=2310042I22Rik /UG_TITLE=RIKEN cDNA 2310042I22 gene /DEF=Mus musculus adult male tongue cDNA, RIKEN full-length enriched library, clone:2310042I22:unclassifiable transcript, full insert sequence.	2922.1003	540.96954	1978.4667	35.65691	3.0147402	2.0	0.09466275	0.40544236
gb:BC002073.1 /DB_XREF=gi:12805224 /FEA=FLmRNA /CNT=111 /TID=Mm.137.1 /TIER=FL+Stack /STK=50 /UG=Mm.137 /LL=20305 /UG_GENE=Scya6 /DEF=Mus musculus, clone MGC:6215 IMAGE:3492808, mRNA, complete cds. /PROD=Unknown (protein for MGC:6215) /FL=gb:NM_009139.1 gb:AF128192.1 gb:AF128191.1 gb:M58004.1 gb:BC002073.1 gb:AF128190.1 gb:AF128189.1 gb:AF128188.1	1573.6334	216.04079	988.26666	366.27304	2.384262	3.0	0.09724311	0.4126016
gb:BC006606.1 /DB_XREF=gi:13879271 /FEA=FLmRNA /CNT=447 /TID=Mm.1034.1 /TIER=FL+Stack /STK=230 /UG=Mm.1034 /LL=51792 /UG_GENE=Ppp2r1a /DEF=Mus musculus, protein phosphatase 2 (formerly 2A), regulatory subunit A (PR 65), alpha isoform, clone MGC:7086 IMAGE:3157293, mRNA, complete cds. /PROD=protein phosphatase 2 (formerly 2A), regulatorysubunit A (PR 65), alpha isoform /FL=gb:NM_016891.1 gb:BC006606.1 gb:AB021743.1	2269.7334	651.43805	1033.5333	312.80685	2.9629393	2.0	0.097528845	0.40998238
gb:AF302138.1 /DB_XREF=gi:15420074 /FEA=FLmRNA /CNT=158 /TID=Mm.22029.1 /TIER=FL+Stack /STK=88 /UG=Mm.22029 /LL=192292 /UG_GENE=NRBP /DEF=Mus musculus HLS7-interacting protein kinase mRNA, complete cds. /PROD=HLS7-interacting protein kinase /FL=gb:BC018463.1 gb:AF302138.1 gb:BC004756.1	1104.3334	297.56613	577.73334	241.51155	2.3799613	3.0	0.097621255	0.40660596
gb:AK006949.1 /DB_XREF=gi:12840262 /GEN=Mgll /FEA=mRNA /CNT=5 /TID=Mm.194795.3 /TIER=ConsEnd /STK=0 /UG=Mm.194795 /LL=23945 /UG_TITLE=monoglyceride lipase /DEF=Mus musculus adult male testis cDNA, RIKEN full-length enriched library, clone:1700074K19:monoglyceride lipase, full insert sequence.	1528.3666	498.10037	664.73334	387.59006	2.3701098	3.0	0.0984942	0.40651244
gb:BC021405.1 /DB_XREF=gi:18204743 /FEA=FLmRNA /CNT=55 /TID=Mm.197542.2 /TIER=FL /STK=3 /UG=Mm.197542 /DEF=Mus musculus, prion protein interacting protein, clone MGC:29172 IMAGE:3985260, mRNA, complete cds. /PROD=prion protein interacting protein /FL=gb:BC021405.1	1429.6333	272.46213	914.4666	261.58444	2.362404	3.0	0.099183634	0.40567
gb:NM_011099.1 /DB_XREF=gi:6755073 /GEN=Pk3 /FEA=FLmRNA /CNT=305 /TID=Mm.2635.1 /TIER=FL+Stack /STK=48 /UG=Mm.2635 /LL=18746 /DEF=Mus musculus pyruvate kinase 3 (Pk3), mRNA. /PROD=pyruvate kinase 3 /FL=gb:D38379.1 gb:BC016619.1 gb:NM_011099.1	10005.267	1748.6866	7033.5664	1307.78	2.3571577	3.0	0.09965639	0.40396428
gb:BC003744.1 /DB_XREF=gi:13277677 /FEA=FLmRNA /CNT=135 /TID=Mm.3294.1 /TIER=FL+Stack /STK=52 /UG=Mm.3294 /LL=19060 /UG_GENE=Ppp5c /DEF=Mus musculus, protein phosphatase 5, catalytic subunit, clone MGC:5847 IMAGE:3590322, mRNA, complete cds. /PROD=protein phosphatase 5, catalytic subunit /FL=gb:BC003744.1 gb:AF018262.1	1420.2334	425.78183	649.23334	171.36757	2.9095585	2.0	0.10061329	0.4042339
gb:NM_016742.1 /DB_XREF=gi:7949017 /GEN=Cdc37 /FEA=FLmRNA /CNT=193 /TID=Mm.4117.1 /TIER=FL+Stack /STK=68 /UG=Mm.4117 /LL=12539 /DEF=Mus musculus cell division cycle 37 homolog (S. cerevisiae) (Cdc37), mRNA. /PROD=cell division cycle 37 homolog (S. cerevisiae) /FL=gb:U43076.1 gb:NM_016742.1	5240.6997	1181.1212	3308.3665	816.72766	2.3307126	3.0	0.10208148	0.40653503
gb:AK004745.1 /DB_XREF=gi:12836150 /FEA=mRNA /CNT=188 /TID=Mm.26696.1 /TIER=Stack /STK=47 /UG=Mm.26696 /LL=74132 /UG_GENE=1200013I08Rik /UG_TITLE=RIKEN cDNA 1200013I08 gene /DEF=Mus musculus adult male lung cDNA, RIKEN full-length enriched library, clone:1200013I08:homolog to RING FINGER PROTEIN 6, full insert sequence.	1038.2334	374.87088	1668.3667	59.046204	2.8760056	2.0	0.10262341	0.40513936
gb:NM_031260.1 /DB_XREF=gi:13994126 /GEN=Mov10l1 /FEA=FLmRNA /CNT=18 /TID=Mm.102985.1 /TIER=FL+Stack /STK=11 /UG=Mm.102985 /LL=83456 /DEF=Mus musculus Moloney leukemia virus 10-like 1 (Mov10l1), mRNA. /PROD=Moloney leukemia virus 10-like 1 /FL=gb:AF285587.1 gb:AF340211.1 gb:NM_031260.1	3885.3	658.83813	2512.8667	787.5243	2.3151417	3.0	0.10354287	0.4052454
gb:NM_019652.1 /DB_XREF=gi:12025541 /GEN=Asna1 /FEA=FLmRNA /CNT=69 /TID=Mm.41475.1 /TIER=FL+Stack /STK=26 /UG=Mm.41475 /LL=56495 /DEF=Mus musculus arsA (bacterial) arsenite transporter, ATP-binding, homolog 1 (Asna1), mRNA. /PROD=arsA (bacterial) arsenite transporter,ATP-binding, homolog 1 /FL=gb:AF039405.2 gb:NM_019652.1 gb:BC016453.1 gb:BC018430.1	1830.5668	545.8855	856.0999	226.17563	2.8564322	2.0	0.10382245	0.40286657
gb:BC024625.1 /DB_XREF=gi:19354249 /FEA=FLmRNA /CNT=148 /TID=Mm.3624.1 /TIER=FL+Stack /STK=92 /UG=Mm.3624 /LL=14923 /UG_GENE=Guk1 /DEF=Mus musculus, guanylate kinase 1, clone MGC:25313 IMAGE:4485215, mRNA, complete cds. /PROD=guanylate kinase 1 /FL=gb:U53514.1 gb:NM_008193.1 gb:BC024625.1	2587.4001	450.45706	1893.2334	261.50537	2.308353	3.0	0.10418795	0.40085873
gb:NM_026633.1 /DB_XREF=gi:13386123 /GEN=9530058B02Rik /FEA=FLmRNA /CNT=69 /TID=Mm.34532.1 /TIER=FL+Stack /STK=53 /UG=Mm.34532 /LL=68241 /DEF=Mus musculus RIKEN cDNA 9530058B02 gene (9530058B02Rik), mRNA. /PROD=RIKEN cDNA 9530058B02 /FL=gb:BC024332.1 gb:NM_026633.1	3585.2668	990.28937	1960.7334	43.036335	2.8386865	2.0	0.104926765	0.40030885
gb:AK004841.1 /DB_XREF=gi:12836328 /FEA=mRNA /CNT=230 /TID=Mm.214574.1 /TIER=Stack /STK=113 /UG=Mm.214574 /LL=74148 /UG_GENE=1300001I01Rik /UG_TITLE=RIKEN cDNA 1300001I01 gene /DEF=Mus musculus adult male liver cDNA, RIKEN full-length enriched library, clone:1300001I01:homolog to PUTATIVE EUKARYOTIC TRANSLATION INITIATION FACTOR 3 SUBUNIT (EIF-3) (FRAGMENT), full insert sequence.	1360.8667	367.4176	757.6667	41.588963	2.825514	2.0	0.105757274	0.400115
gb:NM_021423.1 /DB_XREF=gi:10946785 /GEN=Shank3 /FEA=FLmRNA /CNT=22 /TID=Mm.40237.1 /TIER=FL+Stack /STK=13 /UG=Mm.40237 /LL=58234 /DEF=Mus musculus SH3ankyrin domain gene 3 (Shank3), mRNA. /PROD=SH3ankyrin domain gene 3 /FL=gb:NM_021423.1	1375.2334	373.24136	767.3333	270.64325	2.283785	3.0	0.10656348	0.39983323
gb:NM_007710.1 /DB_XREF=gi:6671761 /GEN=Ckmm /FEA=FLmRNA /CNT=129 /TID=Mm.2375.1 /TIER=FL+Stack /STK=40 /UG=Mm.2375 /LL=12715 /DEF=Mus musculus creatine kinase, muscle (Ckmm), mRNA. /PROD=creatine kinase, muscle /FL=gb:NM_007710.1	22673.635	4027.4065	16177.934	2840.3452	2.2829409	3.0	0.10664627	0.39686397
gb:NM_027884.1 /DB_XREF=gi:13540491 /GEN=Tns /FEA=FLmRNA /CNT=81 /TID=Mm.179671.1 /TIER=FL+Stack /STK=11 /UG=Mm.179671 /LL=21961 /DEF=Mus musculus tensin (Tns), mRNA. /PROD=tensin /FL=gb:NM_027884.1	1340.1333	317.97845	823.73334	34.520206	2.796436	2.0	0.107623875	0.39724585
gb:NM_130889.1 /DB_XREF=gi:18700031 /GEN=Anp32b /FEA=FLmRNA /CNT=202 /TID=Mm.154985.1 /TIER=FL+Stack /STK=57 /UG=Mm.154985 /LL=67628 /DEF=Mus musculus acidic nuclear phosphoprotein 32 family, member B (Anp32b), mRNA. /PROD=acidic nuclear phosphoprotein 32 family, memberB /FL=gb:BC005628.1 gb:BC003489.1 gb:NM_130889.1 gb:AB025582.1	1617.6666	659.1917	2732.2334	218.32278	2.7800574	2.0	0.10869584	0.397967
gb:AK010644.1 /DB_XREF=gi:12846235 /FEA=mRNA /CNT=220 /TID=Mm.12858.1 /TIER=Stack /STK=20 /UG=Mm.12858 /LL=13681 /UG_GENE=Eif4a1 /UG_TITLE=eukaryotic translation initiation factor 4A1 /DEF=Mus musculus ES cells cDNA, RIKEN full-length enriched library, clone:2410039K14:eukaryotic translation initiation factor 4A1, full insert sequence.	1660.4	233.5561	1117.5333	344.78094	2.257882	3.0	0.10913939	0.39639428
gb:NM_015749.1 /DB_XREF=gi:7657638 /GEN=Tcn2 /FEA=FLmRNA /CNT=400 /TID=Mm.20948.1 /TIER=FL+Stack /STK=135 /UG=Mm.20948 /LL=21452 /DEF=Mus musculus transcobalamin 2 (Tcn2), mRNA. /PROD=transcobalamin 2 /FL=gb:AF090686.1 gb:NM_015749.1 gb:BC003720.1	1225.7001	330.6885	686.0	250.05399	2.254748	3.0	0.10945612	0.3943895
gb:BC013503.1 /DB_XREF=gi:15488720 /FEA=FLmRNA /CNT=67 /TID=Mm.28712.1 /TIER=FL+Stack /STK=38 /UG=Mm.28712 /LL=75406 /UG_GENE=1010001M04Rik /DEF=Mus musculus, RIKEN cDNA 1010001M04 gene, clone MGC:19170 IMAGE:4223916, mRNA, complete cds. /PROD=RIKEN cDNA 1010001M04 gene /FL=gb:BC013503.1	13387.966	2335.991	9823.967	1456.2285	2.242521	3.0	0.11070248	0.39573956
gb:NM_011421.1 /DB_XREF=gi:6755581 /GEN=Smpd1 /FEA=FLmRNA /CNT=168 /TID=Mm.4628.1 /TIER=FL+Stack /STK=52 /UG=Mm.4628 /LL=20597 /DEF=Mus musculus sphingomyelin phosphodiesterase 1, acid lysosomal (Smpd1), mRNA. /PROD=sphingomyelin phosphodiesterase 1, acidlysosomal /FL=gb:NM_011421.1 gb:BC011304.1	2998.0667	631.4375	1898.3334	284.94925	2.7495928	2.0	0.11073025	0.3927464
gb:BC003916.1 /DB_XREF=gi:13278141 /FEA=FLmRNA /CNT=88 /TID=Mm.28679.1 /TIER=FL+Stack /STK=10 /UG=Mm.28679 /LL=67669 /UG_GENE=0610007P06Rik /DEF=Mus musculus, RIKEN cDNA 0610007P06 gene, clone MGC:7275 IMAGE:3485003, mRNA, complete cds. /PROD=RIKEN cDNA 0610007P06 gene /FL=gb:NM_026304.1 gb:BC003916.1	797.39996	238.46638	1268.1666	276.37323	2.2337542	3.0	0.11160665	0.39278618
gb:NM_013469.1 /DB_XREF=gi:7304884 /GEN=Anxa11 /FEA=FLmRNA /CNT=81 /TID=Mm.1427.1 /TIER=FL+Stack /STK=23 /UG=Mm.1427 /LL=11744 /DEF=Mus musculus annexin A11 (Anxa11), mRNA. /PROD=annexin A11 /FL=gb:U65986.1 gb:BC012875.1 gb:NM_013469.1	1036.0	315.00784	1605.0668	310.22586	2.2293785	3.0	0.11206127	0.39135244
gb:AK011609.1 /DB_XREF=gi:12847844 /FEA=FLmRNA /CNT=116 /TID=Mm.10433.1 /TIER=Stack /STK=45 /UG=Mm.10433 /LL=11632 /UG_GENE=Aip /UG_TITLE=aryl-hydrocarbon receptor-interacting protein /DEF=Mus musculus 10 days embryo whole body cDNA, RIKEN full-length enriched library, clone:2610029G12:aryl-hydrocarbon receptor-interacting protein, full insert sequence. /FL=gb:U85489.1 gb:NM_016666.1	1455.0333	316.4408	926.43335	113.50154	2.7234232	2.0	0.11252104	0.38995838
gb:NM_008138.1 /DB_XREF=gi:6680036 /GEN=Gnai2 /FEA=FLmRNA /CNT=101 /TID=Mm.196464.1 /TIER=FL+Stack /STK=9 /UG=Mm.196464 /LL=14678 /DEF=Mus musculus guanine nucleotide binding protein, alpha inhibiting 2 (Gnai2), mRNA. /PROD=guanine nucleotide binding protein, alphainhibiting 2 /FL=gb:M13963.1 gb:NM_008138.1	2521.9333	796.52167	1119.0333	408.70044	2.71419	2.0	0.11316262	0.38921085
gb:AB006361.1 /DB_XREF=gi:2317285 /FEA=FLmRNA /CNT=130 /TID=Mm.1008.1 /TIER=FL+Stack /STK=89 /UG=Mm.1008 /LL=19215 /UG_GENE=Ptgds /DEF=Mus musculus mRNA for prostaglandin D synthetase, complete cds. /PROD=prostaglandin D synthetase /FL=gb:AB006361.1	19869.732	4651.599	12197.7	1564.4395	2.707691	2.0	0.113617316	0.38783655
gb:NM_008049.1 /DB_XREF=gi:6679872 /GEN=Ftl2 /FEA=FLmRNA /CNT=2 /TID=Mm.30357.1 /TIER=FL /STK=1 /UG=Mm.30357 /LL=14337 /DEF=Mus musculus ferritin light chain 2 (Ftl2), mRNA. /PROD=ferritin light chain 2 /FL=gb:NM_008049.1	4378.567	944.25793	2859.5667	252.19649	2.69194	2.0	0.11473004	0.38871223
gb:NM_009225.1 /DB_XREF=gi:6678052 /GEN=Snrpb /FEA=FLmRNA /CNT=100 /TID=Mm.88216.1 /TIER=FL+Stack /STK=11 /UG=Mm.88216 /LL=20638 /DEF=Mus musculus small nuclear ribonucleoprotein B (Snrpb), mRNA. /PROD=small nuclear ribonucleoprotein B /FL=gb:NM_009225.1 gb:M58761.1	3224.0664	834.9952	1818.8002	352.37866	2.6856236	2.0	0.115180574	0.38734803
gb:AK002358.1 /DB_XREF=gi:12832278 /FEA=mRNA /CNT=204 /TID=Mm.3533.2 /TIER=Stack /STK=41 /UG=Mm.3533 /LL=56551 /UG_GENE=Txn2 /UG_TITLE=thioredoxin 2 /DEF=Mus musculus adult male kidney cDNA, RIKEN full-length enriched library, clone:0610008P06:thioredoxin, mitochondrial, full insert sequence.	1863.7334	600.1047	981.7666	354.21774	2.1921759	3.0	0.116017826	0.3872948
gb:NM_008379.1 /DB_XREF=gi:6680448 /GEN=Impnb /FEA=FLmRNA /CNT=313 /TID=Mm.16710.1 /TIER=FL+Stack /STK=42 /UG=Mm.16710 /LL=16211 /DEF=Mus musculus importin beta (Impnb), mRNA. /PROD=importin beta /FL=gb:D45836.1 gb:NM_008379.1 gb:D67015.1	976.60004	305.5066	1489.2334	132.98216	2.6648316	2.0	0.116681345	0.38666666

gb:NM_007964.1 /DB_XREF=gi:6679706 /GEN=Evi5 /FEA=FLmRNA /CNT=132 /TID=Mm.35796.1 /TIER=FL+Stack /STK=43 /UG=Mm.35796 /LL=14020 /DEF=Mus musculus ecotropic viral integration site 5 (Evi5), mRNA. /PROD=ecotropic viral integration site 5 /FL=gb:U53586.1 gb:NM_007964.1	792.3667	377.52588	1390.3002	92.49189	2.6644602	2.0	0.11670841	0.38395375
gb:NM_010438.1 /DB_XREF=gi:6754205 /GEN=Hk1 /FEA=FLmRNA /CNT=117 /TID=Mm.196605.1 /TIER=FL /STK=2 /UG=Mm.196605 /LL=15275 /DEF=Mus musculus hexokinase 1 (Hk1), mRNA. /PROD=hexokinase 1 /FL=gb:L16949.1 gb:NM_010438.1	1239.2334	485.96017	504.16666	328.2567	2.1710267	3.0	0.118341856	0.38652664
gb:BC007152.1 /DB_XREF=gi:13938071 /FEA=FLmRNA /CNT=413 /TID=Mm.27818.1 /TIER=FL+Stack /STK=17 /UG=Mm.27818 /LL=13629 /UG_GENE=Eef2 /DEF=Mus musculus, eukaryotic translation elongation factor 2, clone MGC:6761 IMAGE:3600352, mRNA, complete cds. /PROD=eukaryotic translation elongation factor 2 /FL=gb:BC007152.1	14758.233	2760.599	10545.767	1951.1	2.1583295	3.0	0.11976393	0.3883773
gb:NM_007572.1 /DB_XREF=gi:6671649 /GEN=C1qa /FEA=FLmRNA /CNT=119 /TID=Mm.370.1 /TIER=FL+Stack /STK=46 /UG=Mm.370 /LL=12259 /DEF=Mus musculus complement component 1, q subcomponent, alpha polypeptide (C1qa), mRNA. /PROD=complement component 1, q subcomponent, alphapolypeptide /FL=gb:BC002086.1 gb:NM_007572.1	1808.0332	915.5151	389.43335	199.11038	2.6225247	2.0	0.11982115	0.38580713
gb:BC027166.1 /DB_XREF=gi:20073307 /FEA=mRNA /CNT=224 /TID=Mm.219670.1 /TIER=Stack /STK=22 /UG=Mm.219670 /DEF=Mus musculus, Similar to eukaryotic translation initiation factor 4 gamma, 1, clone IMAGE:4950789, mRNA, partial cds. /PROD=Similar to eukaryotic translation initiationfactor 4 gamma, 1	1914.3334	535.31134	1075.0	149.95964	2.6150708	2.0	0.120386615	0.38489804
gb:NM_033617.1 /DB_XREF=gi:15991794 /GEN=Atp6v0b /FEA=FLmRNA /CNT=145 /TID=Mm.3479.1 /TIER=FL+Stack /STK=70 /UG=Mm.3479 /LL=114143 /DEF=Mus musculus ATPase, H+ transporting, lysosomal 21kDa, V0 subunit B (Atp6v0b), mRNA. /PROD=ATPase, H+ transporting, lysosomal 21kDa, V0subunit B /FL=gb:NM_033617.1 gb:AB060654.1 gb:AF356006.1	2499.8333	531.6897	1691.5333	374.72598	2.1523097	3.0	0.12044529	0.38239273
gb:U25633.1 /DB_XREF=gi:1628496 /GEN=TMP /FEA=FLmRNA /CNT=216 /TID=Mm.30024.1 /TIER=FL+Stack /STK=83 /UG=Mm.30024 /LL=13730 /DEF=Mus musculus tumor-associated membrane protein (TMP) mRNA, complete cds. /PROD=tumor-associated membrane protein /FL=gb:U25633.1 gb:NM_010128.1	3414.7334	885.31415	5353.6997	1285.9819	2.1510766	3.0	0.120585434	0.3801791
gb:AK019319.1 /DB_XREF=gi:12859466 /GEN=Apoe /FEA=mRNA /CNT=1 /TID=Mm.138866.2 /TIER=ConsEnd /STK=0 /UG=Mm.138866 /LL=11816 /UG_TITLE=apolipoprotein E /DEF=Mus musculus adult male hippocampus cDNA, RIKEN full-length enriched library, clone:2900019G12:apolipoprotein E, full insert sequence.	15604.3	4270.9517	9267.634	2806.9692	2.147504	3.0	0.12099254	0.3788318
gb:NM_008512.1 /DB_XREF=gi:6678719 /GEN=Lrp1 /FEA=FLmRNA /CNT=182 /TID=Mm.7221.1 /TIER=FL+Stack /STK=49 /UG=Mm.7221 /LL=16971 /DEF=Mus musculus low density lipoprotein receptor-related protein 1 (Lrp1), mRNA. /PROD=low density lipoprotein receptor-related protein1 /FL=gb:AF367720.1 gb:NM_008512.1	2616.4001	712.1777	1542.8334	493.1368	2.1465883	3.0	0.12109717	0.37656245
gb:NM_011918.1 /DB_XREF=gi:6756042 /GEN=Ldb3 /FEA=FLmRNA /CNT=97 /TID=Mm.29733.1 /TIER=FL+Stack /STK=71 /UG=Mm.29733 /LL=24131 /DEF=Mus musculus LIM domain binding 3 (Ldb3), mRNA. /PROD=LIM domain binding 3 /FL=gb:AF114379.2 gb:NM_011918.1	14779.5	2508.98	10855.767	719.2049	2.6038458	2.0	0.12124525	0.3744581
gb:NM_009208.1 /DB_XREF=gi:6678022 /GEN=Slc4a3 /FEA=FLmRNA /CNT=45 /TID=Mm.5053.1 /TIER=FL+Stack /STK=23 /UG=Mm.5053 /LL=20536 /DEF=Mus musculus solute carrier family 4 (anion exchanger), member 3 (Slc4a3), mRNA. /PROD=solute carrier family 4 (anion exchanger),member 3 /FL=gb:M28383.1 gb:NM_009208.1	2166.0	661.7367	1131.3334	191.27336	2.601667	2.0	0.12141292	0.37244233
gb:AV225808 /DB_XREF=gi:6177123 /DB_XREF=AV225808 /CLONE=3830432A03 /FEA=EST /CNT=20 /TID=Mm.219675.2 /TIER=Stack /STK=13 /UG=Mm.219675 /UG_TITLE=Mus musculus, Similar to elongation factor G2, clone MGC:28160 IMAGE:3984129, mRNA, complete cds	2209.2	1237.3901	4263.6665	585.0482	2.599816	2.0	0.1215556	0.37037745
gb:NM_010240.1 /DB_XREF=gi:6753913 /GEN=Ftl1 /FEA=FLmRNA /CNT=109 /TID=Mm.7500.1 /TIER=FL+Stack /STK=24 /UG=Mm.7500 /LL=14325 /DEF=Mus musculus ferritin light chain 1 (Ftl1), mRNA. /PROD=ferritin light chain 1 /FL=gb:J04716.1 gb:NM_010240.1 gb:BC019840.1	5862.8335	943.54614	4308.5664	432.8185	2.5933146	2.0	0.122058645	0.36943084
gb:AF274027.1 /DB_XREF=gi:14971094 /FEA=FLmRNA /CNT=5 /TID=Mm.2400.2 /TIER=FL /STK=2 /UG=Mm.2400 /LL=14779 /UG_GENE=Gpx4 /DEF=Mus musculus phospholipid hydroperoxide glutathione peroxidase mRNA, complete cds, alternatively spliced. /PROD=phospholipid hydroperoxide glutathioneperoxidase /FL=gb:AF274027.1	23738.666	5152.416	15873.466	1223.0314	2.5725076	2.0	0.12368836	0.3718842
gb:BC006753.1 /DB_XREF=gi:13879545 /FEA=FLmRNA /CNT=190 /TID=Mm.3634.1 /TIER=FL+Stack /STK=71 /UG=Mm.3634 /DEF=Mus musculus, clone MGC:11670 IMAGE:3709076, mRNA, complete cds. /PROD=Unknown (protein for MGC:11670) /FL=gb:AY028317.1 gb:BC006753.1	1298.4333	74.20587	789.0334	335.8784	2.5650096	2.0	0.12428313	0.37121406
gb:NM_021538.1 /DB_XREF=gi:10946971 /GEN=Cope /FEA=FLmRNA /CNT=222 /TID=Mm.28668.1 /TIER=FL+Stack /STK=93 /UG=Mm.28668 /LL=59042 /DEF=Mus musculus coatomer protein complex, subunit epsilon (Cope), mRNA. /PROD=coatomer protein complex, subunit epsilon /FL=gb:AB039837.1 gb:BC009170.1 gb:NM_021538.1	2886.7668	464.41937	2023.2333	537.0535	2.1065724	3.0	0.12577623	0.3732184
gb:NM_009349.1 /DB_XREF=gi:6678280 /GEN=Temt /FEA=FLmRNA /CNT=97 /TID=Mm.299.1 /TIER=FL+Stack /STK=19 /UG=Mm.299 /LL=21743 /DEF=Mus musculus thioether S-methyltransferase (Temt), mRNA. /PROD=thioether S-methyltransferase /FL=gb:M88694.1 gb:NM_009349.1 gb:BC013518.1	3501.0999	800.25867	2259.0667	277.9903	2.539362	2.0	0.12634811	0.37248078
gb:AK003715.1 /DB_XREF=gi:12834551 /GEN=D11Wsu68e /FEA=mRNA /CNT=48 /TID=Mm.24855.1 /TIER=Stack /STK=29 /UG=Mm.24855 /LL=28084 /UG_TITLE=DNA segment, Chr 11, Wayne State University 68, expressed /DEF=Mus musculus 18 days embryo whole body cDNA, RIKEN full-length enriched library, clone:1110014N10:DNA segment, Chr 11, Wayne State University 68, expressed, full insert sequence.	2237.9	257.32095	1627.3334	434.5673	2.0939682	3.0	0.12729448	0.37284964
gb:NM_009077.1 /DB_XREF=gi:6677770 /GEN=Rpl18 /FEA=FLmRNA /CNT=2 /TID=Mm.41923.1 /TIER=FL /STK=2 /UG=Mm.41923 /LL=19899 /DEF=Mus musculus ribosomal protein L18 (Rpl18), mRNA. /PROD=ribosomal protein L18 /FL=gb:NM_009077.1 gb:L04128.1	12883.8	2866.7754	8467.8	986.7063	2.5228121	2.0	0.12770614	0.3716576
gb:AK017523.1 /DB_XREF=gi:12856802 /FEA=mRNA /CNT=39 /TID=Mm.29092.2 /TIER=ConsEnd /STK=1 /UG=Mm.29092 /LL=70510 /UG_GENE=5730408C10Rik /UG_TITLE=RIKEN cDNA 5730408C10 gene /DEF=Mus musculus 8 days embryo whole body cDNA, RIKEN full-length enriched library, clone:5730408C10:C3HC4 type (RING finger) Zinc finger containing protein, full insert sequence.	1612.5	484.06375	910.66675	328.9321	2.0770903	3.0	0.12936181	0.3740781
gb:NM_020255.1 /DB_XREF=gi:9937979 /GEN=Scand1 /FEA=FLmRNA /CNT=74 /TID=Mm.29477.1 /TIER=FL+Stack /STK=34 /UG=Mm.29477 /LL=19018 /DEF=Mus musculus SCAN domain-containing 1 (Scand1), mRNA. /PROD=paroxisome proliferative activated receptor,gamma, coactivator 1 /FL=gb:BC010724.1 gb:NM_020255.1 gb:AF106473.1 gb:AF220501.1	1806.9667	392.72913	1233.5	58.524418	2.5015333	2.0	0.1294824	0.37205702
gb:NM_017379.1 /DB_XREF=gi:8394492 /GEN=Tuba8 /FEA=FLmRNA /CNT=31 /TID=Mm.32884.1 /TIER=FL+Stack /STK=9 /UG=Mm.32884 /LL=53857 /DEF=Mus musculus tubulin, alpha 8 (Tuba8), mRNA. /PROD=tubulin, alpha 8 /FL=gb:BC017631.1 gb:NM_017379.1	3518.2668	731.5148	2430.4001	541.90027	2.069757	3.0	0.13027246	0.37197295
gb:NM_008906.1 /DB_XREF=gi:6679436 /GEN=Ppgb /FEA=FLmRNA /CNT=327 /TID=Mm.7046.1 /TIER=FL+Stack /STK=187 /UG=Mm.7046 /LL=19025 /DEF=Mus musculus protective protein for beta-galactosidase (Ppgb), mRNA. /PROD=protective protein for beta-galactosidase /FL=gb:BC018534.1 gb:J05261.1 gb:NM_008906.1	3347.4336	684.5403	2355.2668	469.90833	2.069697	3.0	0.13027996	0.3696694
gb:BC025650.1 /DB_XREF=gi:19343805 /FEA=FLmRNA /CNT=336 /TID=Mm.29490.1 /TIER=FL+Stack /STK=189 /UG=Mm.29490 /DEF=Mus musculus, Similar to conserved gene amplified in osteosarcoma, clone MGC:38258 IMAGE:5324816, mRNA, complete cds. /PROD=Similar to conserved gene amplified inosteosarcoma /FL=gb:BC025650.1	6629.7007	1163.0481	4860.3335	919.62646	2.0669303	3.0	0.13062552	0.36834773
gb:NM_009149.1 /DB_XREF=gi:6677904 /GEN=Selel /FEA=FLmRNA /CNT=216 /TID=Mm.488.1 /TIER=FL+Stack /STK=57 /UG=Mm.488 /LL=20340 /DEF=Mus musculus selectin, endothelial cell, ligand (Selel), mRNA. /PROD=selectin, endothelial cell, ligand /FL=gb:NM_009149.1 gb:BC021306.1	4186.1665	1061.3314	2741.0332	597.75024	2.054902	3.0	0.13214059	0.37031993
gb:AK003436.1 /DB_XREF=gi:12834106 /GEN=Psmd8 /FEA=FLmRNA /CNT=285 /TID=Mm.35812.1 /TIER=Stack /STK=17 /UG=Mm.35812 /LL=57296 /UG_TITLE=proteasome (prosome, macropain) 26S subunit, non-ATPase, 8 /DEF=Mus musculus 18 days embryo whole body cDNA, RIKEN full-length enriched library, clone:1110004L21:proteasome (prosome, macropain) 26S subunit, non-ATPase, 8, full insert sequence. /FL=gb:BC005717.1 gb:BC004075.1 gb:NM_026545.1	3733.7	823.347	2583.7334	512.53284	2.0537407	3.0	0.13228798	0.36845854
gb:BC028256.1 /DB_XREF=gi:20380524 /FEA=mRNA /CNT=19 /TID=Mm.45357.2 /TIER=ConsEnd /STK=0 /UG=Mm.45357 /LL=59079 /UG_GENE=Erbb2ip /DEF=Mus musculus, Similar to erbb2 interacting protein, clone IMAGE:4988135, mRNA, partial cds. /PROD=Similar to erbb2 interacting protein	1193.3667	589.4717	2155.9001	562.5233	2.0460775	3.0	0.1332654	0.36891764
gb:NM_008968.1 /DB_XREF=gi:6679534 /GEN=Ptgis /FEA=FLmRNA /CNT=65 /TID=Mm.2339.1 /TIER=FL+Stack /STK=38 /UG=Mm.2339 /LL=19223 /DEF=Mus musculus prostaglandin I2 (prostacyclin) synthase (Ptgis), mRNA. /PROD=prostaglandin I2 (prostacyclin) synthase /FL=gb:AB001607.1 gb:NM_008968.1	3961.1667	958.07043	2481.9668	417.30057	2.4517064	2.0	0.13377887	0.3680946
gb:NM_008896.1 /DB_XREF=gi:6679416 /GEN=Pon2 /FEA=FLmRNA /CNT=70 /TID=Mm.22681.1 /TIER=FL+Stack /STK=27 /UG=Mm.22681 /LL=18980 /DEF=Mus musculus paraoxonase 2 (Pon2), mRNA. /PROD=paraoxonase 2 /FL=gb:L48514.1 gb:NM_008896.1	1238.8	514.217	1998.3667	390.5902	2.0373657	3.0	0.13438699	0.36754033
gb:NM_025933.1 /DB_XREF=gi:13385425 /GEN=2010110M21Rik /FEA=FLmRNA /CNT=99 /TID=Mm.29353.1 /TIER=FL+Stack /STK=31 /UG=Mm.29353 /LL=67044 /DEF=Mus musculus RIKEN cDNA 2010110M21 gene (2010110M21Rik), mRNA. /PROD=RIKEN cDNA 2010110M21 /FL=gb:NM_025933.1 gb:BC021471.1	9029.7	1679.2811	6378.6333	848.9092	2.440287	2.0	0.13479145	0.366439
gb:BC013670.1 /DB_XREF=gi:15489119 /FEA=FLmRNA /CNT=368 /TID=Mm.30074.1 /TIER=FL+Stack /STK=43 /UG=Mm.30074 /DEF=Mus musculus, clone MGC:19042 IMAGE:4188988, mRNA, complete cds. /PROD=Unknown (protein for MGC:19042) /FL=gb:BC013670.1	9587.733	1456.2542	6396.4336	2294.6082	2.0338852	3.0	0.1348382	0.36438417
gb:NM_011892.1 /DB_XREF=gi:6755487 /GEN=Sgcg /FEA=FLmRNA /CNT=12 /TID=Mm.72173.1 /TIER=FL /STK=2 /UG=Mm.72173 /LL=24053 /DEF=Mus musculus sarcoglycan, gamma (35kD dystrophin-associated glycoprotein) (Sgcg), mRNA. /PROD=sarcoglycan, gamma (35kD dystrophin-associatedglycoprotein) /FL=gb:NM_011892.1 gb:AB024922.1	3929.0	206.34653	2650.0	888.793	2.4278996	2.0	0.13590199	0.36508584
gb:NM_008749.1 /DB_XREF=gi:6679157 /GEN=Nucb /FEA=FLmRNA /CNT=332 /TID=Mm.2283.1 /TIER=FL+Stack /STK=64 /UG=Mm.2283 /LL=18220 /DEF=Mus musculus nucleobindin (Nucb), mRNA. /PROD=nucleobindin /FL=gb:NM_008749.1 gb:M96823.1	2056.5332	457.68692	1335.7333	414.98807	2.020778	3.0	0.13655357	0.36467835
gb:BC026486.1 /DB_XREF=gi:20071462 /FEA=FLmRNA /CNT=174 /TID=Mm.31239.1 /TIER=FL+Stack /STK=156 /UG=Mm.31239 /DEF=Mus musculus, Similar to LGN protein, clone MGC:31563 IMAGE:4504055, mRNA, complete cds. /PROD=Similar to LGN protein /FL=gb:BC026486.1	1239.2333	239.45042	746.6333	348.11758	2.0193365	3.0	0.1367438	0.3630508
gb:BC021463.1 /DB_XREF=gi:18204796 /FEA=FLmRNA /CNT=46 /TID=Mm.29372.1 /TIER=FL+Stack /STK=27 /UG=Mm.29372 /LL=67675 /UG_GENE=0610039D01Rik /DEF=Mus musculus, Similar to RIKEN cDNA 0610039D01 gene, clone MGC:29169 IMAGE:5053934, mRNA, complete cds. /PROD=Similar to RIKEN cDNA 0610039D01 gene /FL=gb:BC021463.1	2120.3	536.66815	1357.9	103.11544	2.4163818	2.0	0.13694601	0.36147377
gb:BC027021.1 /DB_XREF=gi:20071695 /FEA=FLmRNA /CNT=111 /TID=Mm.28577.1 /TIER=FL+Stack /STK=38 /UG=Mm.28577 /LL=68463 /UG_GENE=1110006I11Rik /DEF=Mus musculus, RIKEN cDNA 1110006I11 gene, clone MGC:36524 IMAGE:5374870, mRNA, complete cds. /PROD=RIKEN cDNA 1110006I11 gene /FL=gb:BC027021.1	2301.3	584.0858	1471.8667	408.19916	2.0160584	3.0	0.13717754	0.35999194
gb:NM_023133.1 /DB_XREF=gi:12963510 /GEN=Rps19 /FEA=FLmRNA /CNT=72 /TID=Mm.103634.1 /TIER=FL+Stack /STK=17 /UG=Mm.103634 /LL=20085 /DEF=Mus musculus ribosomal protein S19 (Rps19), mRNA. /PROD=ribosomal protein S19 /FL=gb:NM_023133.1	12766.899	2580.3882	8981.033	886.1002	2.4034498	2.0	0.13813154	0.36041218
gb:NM_023341.1 /DB_XREF=gi:12963648 /GEN=4632432J16Rik /FEA=FLmRNA /CNT=129 /TID=Mm.28337.1 /TIER=FL+Stack /STK=58 /UG=Mm.28337 /LL=67426 /DEF=Mus musculus RIKEN cDNA 4632432J16 gene (4632432J16Rik), mRNA. /PROD=RIKEN cDNA 4632432J16 /FL=gb:NM_023341.1	7496.6665	1431.8107	5438.7334	389.1163	2.402333	2.0	0.13823459	0.35862002
gb:BC006760.1 /DB_XREF=gi:13879559 /FEA=FLmRNA /CNT=160 /TID=Mm.24353.1 /TIER=FL+Stack /STK=69 /UG=Mm.24353 /LL=56418 /UG_GENE=Ykt6-pending /DEF=Mus musculus, prenylated SNARE protein, clone MGC:5770 IMAGE:3257673, mRNA, complete cds. /PROD=prenylated SNARE protein /FL=gb:AF076956.1 gb:NM_019661.1 gb:BC006760.1	1423.2999	314.74496	936.23334	278.5027	2.0073311	3.0	0.13834026	0.35685498
gb:BM245572 /DB_XREF=gi:17880842 /DB_XREF=K0726E03-3 /CLONE=K0726E03 /FEA=mRNA /CNT=10 /TID=Mm.204648.1 /TIER=ConsEnd /STK=7 /UG=Mm.204648 /UG_TITLE=Mus musculus adult male kidney cDNA, RIKEN full-length enriched library, clone:0610010O05:lymphocyte antigen 6 complex, locus E, full insert sequence	7878.033	2055.934	5033.4663	1347.9259	2.004116	3.0	0.13877152	0.35594502
gb:BC025450.1 /DB_XREF=gi:19343774 /FEA=mRNA /CNT=171 /TID=Mm.24463.1 /TIER=Stack /STK=124 /UG=Mm.24463 /LL=70049 /UG_GENE=2610044B22Rik /UG_TITLE=RIKEN cDNA 2610044B22 gene /DEF=Mus musculus, clone IMAGE:5345597, mRNA.	2023.8334	822.1557	3157.9336	534.31946	2.0033257	3.0	0.1388778	0.3542164
gb:BC005621.1 /DB_XREF=gi:13542843 /FEA=FLmRNA /CNT=178 /TID=Mm.29097.1 /TIER=FL+Stack /STK=27 /UG=Mm.29097 /LL=110829 /UG_GENE=Lims1l /DEF=Mus musculus, RIKEN cDNA 4921524A02 gene, clone MGC:11585 IMAGE:3711611, mRNA, complete cds. /PROD=RIKEN cDNA 4921524A02 gene /FL=gb:NM_026148.1 gb:BC005621.1	3386.3333	739.64	2136.9666	790.78	1.9985387	3.0	0.13952343	0.35387507
gb:NM_007752.1 /DB_XREF=gi:6680996 /GEN=Cp /FEA=FLmRNA /CNT=332 /TID=Mm.13787.1 /TIER=FL+Stack /STK=43 /UG=Mm.13787 /LL=12870 /DEF=Mus musculus ceruloplasmin (Cp), mRNA. /PROD=ceruloplasmin /FL=gb:U49430.1 gb:NM_007752.1	4454.0	455.7452	2840.6335	1083.2803	2.3777456	2.0	0.14053063	0.35444948
gb:NM_019966.1 /DB_XREF=gi:9910433 /GEN=Mlycd /FEA=FLmRNA /CNT=8 /TID=Mm.20260.1 /TIER=FL /STK=3 /UG=Mm.20260 /LL=56690 /DEF=Mus musculus malonyl-CoA decarboxylase (Mlycd), mRNA. /PROD=malonyl-CoA decarboxylase /FL=gb:NM_019966.1	1357.1666	386.15103	839.89996	232.48802	1.9877075	3.0	0.14099741	0.353662
gb:BC027285.1 /DB_XREF=gi:20071022 /FEA=FLmRNA /CNT=65 /TID=Mm.175661.1 /TIER=FL+Stack /STK=42 /UG=Mm.175661 /LL=68713 /UG_GENE=1110036C17Rik /DEF=Mus musculus, Similar to RIKEN cDNA 1110036C17 gene, clone MGC:27862 IMAGE:3492212, mRNA, complete cds. /PROD=Similar to RIKEN cDNA 1110036C17 gene /FL=gb:BC027285.1	1052.9667	369.74796	534.93335	259.89047	1.9853188	3.0	0.14132495	0.35253587
gb:AK019396.1 /DB_XREF=gi:12859581 /FEA=mRNA /CNT=68 /TID=Mm.44226.2 /TIER=Stack /STK=39 /UG=Mm.44226 /LL=68066 /UG_GENE=3010027G13Rik /UG_TITLE=RIKEN cDNA 3010027G13 gene /DEF=Mus musculus 12 days embryo head cDNA, RIKEN full-length enriched library, clone:3010027G13:homolog to HYPOTHETICAL 38.5 KDA PROTEIN, full insert sequence.	1165.7667	342.06116	664.5	142.46161	2.3431087	2.0	0.14385577	0.35688806
gb:NM_019722.1 /DB_XREF=gi:9789878 /GEN=Arl2 /FEA=FLmRNA /CNT=74 /TID=Mm.21071.1 /TIER=FL+Stack /STK=39 /UG=Mm.21071 /LL=56327 /DEF=Mus musculus ADP-ribosylation-like 2 (Arl2), mRNA. /PROD=ADP-ribosylation-like 2 /FL=gb:NM_019722.1 gb:AF143680.1	1832.2001	534.95233	1139.4667	293.3553	1.9666193	3.0	0.14392015	0.35510734

gb:NM_016763.1 /DB_XREF=gi:7949046 /GEN=Hsd17b10 /FEA=FLmRNA /CNT=195 /TID=Mm.6994.1 /TIER=FL+Stack /STK=101 /UG=Mm.6994 /LL=15108 /DEF=Mus musculus hydroxysteroid (17-beta) dehydrogenase 10 (Hsd17b10), mRNA. /PROD=hydroxysteroid (17-beta) dehydrogenase 10 /FL=gb:AF233685.1 gb:NM_016763.1 gb:U96116.1	7606.8335	1108.047	5424.6333	1571.2577	1.9658613	3.0	0.14402652	0.35344887
gb:NM_009739.1 /DB_XREF=gi:6753163 /GEN=Bckdk /FEA=FLmRNA /CNT=129 /TID=Mm.8903.1 /TIER=FL+Stack /STK=68 /UG=Mm.8903 /LL=12041 /DEF=Mus musculus branched chain ketoacid dehydrogenase kinase (Bckdk), mRNA. /PROD=branched chain keto acid dehydrogenase kinase /FL=gb:AF043070.1 gb:NM_009739.1	1531.5	364.39032	1027.8334	89.51983	2.3249393	2.0	0.14564368	0.35549587
gb:NM_030561.1 /DB_XREF=gi:13385411 /GEN=BC004004 /FEA=FLmRNA /CNT=146 /TID=Mm.25308.1 /TIER=FL+Stack /STK=78 /UG=Mm.25308 /LL=80748 /DEF=Mus musculus hypothetical protein, MGC:7550 (BC004004), mRNA. /PROD=hypothetical protein MGC7550 /FL=gb:BC004004.1 gb:NM_030561.1	7795.6333	1556.6836	5527.3667	672.38983	2.3169024	2.0	0.14644434	0.35553867
gb:NM_013602.1 /DB_XREF=gi:7305284 /GEN=Mt1 /FEA=FLmRNA /CNT=145 /TID=Mm.219629.1 /TIER=FL+Stack /STK=36 /UG=Mm.219629 /LL=17748 /DEF=Mus musculus metallothionein 1 (Mt1), mRNA. /PROD=metallothionein 1 /FL=gb:NM_013602.1	11612.833	3314.8948	7316.1665	1936.7217	1.9384388	3.0	0.14793773	0.35725388
gb:U43884.1 /DB_XREF=gi:1244792 /GEN=Id1B /FEA=FLmRNA /CNT=3 /TID=Mm.444.2 /TIER=FL /STK=1 /UG=Mm.444 /LL=15901 /DEF=Mus musculus transcription factor Id1B (Id1B) mRNA, complete cds. /PROD=Id1B /FL=gb:U43884.1	1394.6	364.95178	889.7	273.0372	1.9186989	3.0	0.15083033	0.36231202
gb:BC026751.1 /DB_XREF=gi:20071192 /FEA=FLmRNA /CNT=36 /TID=Mm.41486.1 /TIER=FL /STK=1 /UG=Mm.41486 /LL=70103 /UG_GENE=2700001K05Rik /DEF=Mus musculus, Similar to putative cyclin G1 interacting protein, clone MGC:25516 IMAGE:3153902, mRNA, complete cds. /PROD=Similar to putative cyclin G1 interactingprotein /FL=gb:BC026751.1	1355.3667	312.11258	905.0999	262.02222	1.9137493	3.0	0.15156595	0.36216286
gb:BC025071.1 /DB_XREF=gi:19264072 /FEA=FLmRNA /CNT=132 /TID=Mm.2875.2 /TIER=FL+Stack /STK=58 /UG=Mm.2875 /LL=66855 /UG_GENE=1100001J13Rik /DEF=Mus musculus, Similar to RIKEN cDNA 1100001J13 gene, clone MGC:36794 IMAGE:3498003, mRNA, complete cds. /PROD=Similar to RIKEN cDNA 1100001J13 gene /FL=gb:BC025071.1	2475.1667	329.6616	1750.5	568.4756	1.9100156	3.0	0.15212366	0.36159238
gb:NM_024227.1 /DB_XREF=gi:13195649 /GEN=1110015G04Rik /FEA=FLmRNA /CNT=160 /TID=Mm.27332.1 /TIER=FL+Stack /STK=102 /UG=Mm.27332 /LL=68611 /DEF=Mus musculus RIKEN cDNA 1110015G04 gene (1110015G04Rik), mRNA. /PROD=RIKEN cDNA 1110015G04 /FL=gb:NM_024227.1 gb:BC002293.1 gb:BC005797.1	4231.767	809.5277	3110.2998	290.3462	2.2585943	2.0	0.15243916	0.3604551
gb:BC024610.1 /DB_XREF=gi:19354311 /FEA=FLmRNA /CNT=39 /TID=Mm.46628.2 /TIER=FL+Stack /STK=21 /UG=Mm.46628 /LL=22344 /UG_GENE=Vezf1 /DEF=Mus musculus, vascular endothelial zinc finger 1, clone MGC:28455 IMAGE:4159675, mRNA, complete cds. /PROD=vascular endothelial zinc finger 1 /FL=gb:BC024610.1	1260.9	340.7898	789.93335	122.7772	2.251978	2.0	0.15314065	0.3602376
gb:AK011360.1 /DB_XREF=gi:12847432 /FEA=mRNA /CNT=64 /TID=Mm.142187.1 /TIER=Stack /STK=24 /UG=Mm.142187 /LL=69202 /UG_GENE=2610009E16Rik /UG_TITLE=RIKEN cDNA 2610009E16 gene /DEF=Mus musculus 10 days embryo whole body cDNA, RIKEN full-length enriched library, clone:2610009E16:homolog to PARATHYMOSIN (ZINC-BINDING 11.5 KDA PROTEIN), full insert sequence.	3908.8	1215.2148	2384.5	673.47577	1.9002765	3.0	0.15358968	0.35943154
gb:AK007209.1 /DB_XREF=gi:12840620 /GEN=Cappb1 /FEA=mRNA /CNT=2 /TID=Mm.2945.3 /TIER=ConsEnd /STK=1 /UG=Mm.2945 /LL=12345 /UG_TITLE=capping protein beta 1 /DEF=Mus musculus adult male testis cDNA, RIKEN full-length enriched library, clone:1700120C01:capping protein beta 1, full insert sequence.	3347.5	1118.8156	1890.4668	725.1011	1.892879	3.0	0.15471424	0.36020648
gb:BC027637.1 /DB_XREF=gi:20380352 /FEA=mRNA /CNT=2 /TID=Mm.29528.1 /TIER=ConsEnd /STK=0 /UG=Mm.29528 /LL=68212 /UG_GENE=0610007H07Rik /DEF=Mus musculus, clone MGC:41260 IMAGE:1346012, mRNA, complete cds. /PROD=Unknown (protein for MGC:41260)	2423.5	500.8672	1659.0332	496.18042	1.8780776	3.0	0.15699328	0.36364773
gb:BC007140.1 /DB_XREF=gi:13938048 /FEA=FLmRNA /CNT=129 /TID=Mm.219663.2 /TIER=FL+Stack /STK=107 /UG=Mm.219663 /LL=14114 /UG_GENE=Fbln1 /DEF=Mus musculus, Similar to fibulin 1, clone MGC:6128 IMAGE:3495754, mRNA, complete cds. /PROD=Similar to fibulin 1 /FL=gb:BC007140.1	2261.4333	914.7857	1040.7667	275.78848	2.2128296	2.0	0.15738344	0.3627009
gb:NM_019932.1 /DB_XREF=gi:9910485 /GEN=Pf4 /FEA=FLmRNA /CNT=47 /TID=Mm.23905.1 /TIER=FL+Stack /STK=28 /UG=Mm.23905 /LL=56744 /DEF=Mus musculus platelet factor 4 (Pf4), mRNA. /PROD=platelet factor 4 /FL=gb:AB017491.2 gb:NM_019932.1	3736.8665	923.7606	2606.4666	488.46692	1.8736765	3.0	0.15767846	0.36154556
gb:NM_010312.1 /DB_XREF=gi:13937390 /GEN=Gnb2 /FEA=FLmRNA /CNT=367 /TID=Mm.30141.1 /TIER=FL+Stack /STK=141 /UG=Mm.30141 /LL=14693 /DEF=Mus musculus guanine nucleotide binding protein, beta 2 (Gnb2), mRNA. /PROD=guanine nucleotide-binding protein, beta-2subunit /FL=gb:U34960.1 gb:NM_010312.1	1156.7001	482.16302	477.7333	227.77388	2.2053285	2.0	0.15821478	0.3609523
gb:NM_011797.1 /DB_XREF=gi:6753263 /GEN=Car14 /FEA=FLmRNA /CNT=41 /TID=Mm.34556.1 /TIER=FL+Stack /STK=16 /UG=Mm.34556 /LL=23831 /DEF=Mus musculus carbonic anhydrase 14 (Car14), mRNA. /PROD=carbonic anhydrase 14 /FL=gb:NM_011797.1 gb:AB005450.1	1273.2001	392.50107	750.2666	284.58118	1.8682411	3.0	0.15852948	0.3598619
gb:AK004089.1 /DB_XREF=gi:12835132 /FEA=mRNA /CNT=42 /TID=Mm.29458.1 /TIER=Stack /STK=17 /UG=Mm.29458 /LL=68746 /UG_GENE=1110034G11Rik /UG_TITLE=RIKEN cDNA 1110034G11 gene /DEF=Mus musculus 18 days embryo whole body cDNA, RIKEN full-length enriched library, clone:1110034G11:related to CG12379 PROTEIN, full insert sequence.	2279.8	659.10315	1438.1333	63.327145	2.2016687	2.0	0.15862256	0.3582818
gb:U16321.1 /DB_XREF=gi:1185384 /FEA=FLmRNA /CNT=80 /TID=Mm.4269.2 /TIER=FL+Stack /STK=55 /UG=Mm.4269 /LL=21413 /UG_GENE=Tcf4 /DEF=Mus musculus basic transcription factor MITF-2A mRNA, complete cds. /PROD=MITF-2A protein /FL=gb:U16321.1 gb:BC014293.1	1709.9332	440.62988	1114.2999	333.23102	1.8674502	3.0	0.15865374	0.3565782
gb:AF332085.1 /DB_XREF=gi:14193736 /GEN=Rad21 /FEA=FLmRNA /CNT=282 /TID=Mm.182628.1 /TIER=FL /STK=2 /UG=Mm.182628 /LL=19357 /DEF=Mus musculus strain ISS Rad21 (Rad21) mRNA, complete cds. /PROD=Rad21 /FL=gb:AF332086.1 gb:AF332085.1 gb:NM_009009.1 gb:D49429.1	823.76666	335.08176	380.09998	241.96138	1.8592664	3.0	0.15994632	0.35771248
gb:NM_133739.1 /DB_XREF=gi:19526937 /GEN=2310075C12Rik /FEA=FLmRNA /CNT=150 /TID=Mm.146516.1 /TIER=FL+Stack /STK=52 /UG=Mm.146516 /LL=71929 /DEF=Mus musculus RIKEN cDNA 2310075C12 gene (2310075C12Rik), mRNA. /PROD=RIKEN cDNA 2310075C12 /FL=gb:BC010292.1 gb:NM_133739.1	797.3333	489.41528	1390.7999	263.78073	1.8488518	3.0	0.16160896	0.35965917
gb:BC004829.1 /DB_XREF=gi:13435995 /FEA=FLmRNA /CNT=206 /TID=Mm.2079.1 /TIER=FL+Stack /STK=57 /UG=Mm.2079 /LL=20973 /UG_GENE=Syngr2 /DEF=Mus musculus, synaptogyrin 2, clone MGC:6483 IMAGE:2646657, mRNA, complete cds. /PROD=synaptogyrin 2 /FL=gb:AF364049.1 gb:BC004829.1 gb:AF151985.1 gb:NM_009304.1	1586.0668	384.5148	1058.5	310.58392	1.8486891	3.0	0.1616351	0.3579626
gb:NM_026468.1 /DB_XREF=gi:13385959 /GEN=Atp5g2 /FEA=FLmRNA /CNT=334 /TID=Mm.10314.1 /TIER=FL+Stack /STK=154 /UG=Mm.10314 /LL=67942 /DEF=Mus musculus ATP synthase, H+ transporting, mitochondrial F0 complex, subunit c (subunit 9), isoform 2 (Atp5g2), mRNA. /PROD=ATP synthase, H+ transporting, mitochondrial F0complex, subunit c (subunit 9), isoform 2 /FL=gb:NM_026468.1 gb:BC006813.1	13885.966	3905.741	8379.966	1993.4896	2.1748064	2.0	0.16166022	0.3562803
gb:NM_019719.1 /DB_XREF=gi:9789906 /GEN=Stub1 /FEA=FLmRNA /CNT=166 /TID=Mm.29000.1 /TIER=FL+Stack /STK=80 /UG=Mm.29000 /LL=56424 /DEF=Mus musculus STIP1 homology and U-Box containing protein 1 (Stub1), mRNA. /PROD=STIP1 homology and U-Box containing protein 1 /FL=gb:NM_019719.1 gb:AF129086.1	4352.6333	730.2896	3130.9333	883.24316	1.8463746	3.0	0.16200738	0.35532054
gb:BC026760.1 /DB_XREF=gi:20070870 /FEA=FLmRNA /CNT=109 /TID=Mm.3328.1 /TIER=FL+Stack /STK=41 /UG=Mm.3328 /LL=17904 /UG_GENE=Myln /DEF=Mus musculus, clone MGC:25573 IMAGE:3990090, mRNA, complete cds. /PROD=Unknown (protein for MGC:25573) /FL=gb:BC026760.1	10349.899	2328.1802	7504.567	1345.0183	1.8329036	3.0	0.16419399	0.35838494
gb:NM_008713.1 /DB_XREF=gi:6679089 /GEN=Nos3 /FEA=FLmRNA /CNT=49 /TID=Mm.12837.1 /TIER=FL+Stack /STK=25 /UG=Mm.12837 /LL=18127 /DEF=Mus musculus nitric oxide synthase 3, endothelial cell (Nos3), mRNA. /PROD=nitric oxide synthase 3, endothelial cell /FL=gb:U53142.1 gb:NM_008713.1	1535.2333	382.03305	1020.69995	303.48578	1.8265747	3.0	0.16523309	0.3589274
gb:NM_010347.1 /DB_XREF=gi:6754071 /GEN=Aes /FEA=FLmRNA /CNT=60 /TID=Mm.2626.1 /TIER=FL /STK=3 /UG=Mm.2626 /LL=14797 /DEF=Mus musculus amino-terminal enhancer of split (Aes), mRNA. /PROD=related to Drosophila groucho gene /FL=gb:NM_010347.1 gb:L12140.1	3260.8	854.0968	2197.4668	143.65387	2.1264997	2.0	0.16732621	0.36174333
gb:AK007494.1 /DB_XREF=gi:12841074 /FEA=mRNA /CNT=44 /TID=Mm.41915.1 /TIER=Stack /STK=25 /UG=Mm.41915 /LL=69064 /UG_GENE=1810014F10Rik /UG_TITLE=RIKEN cDNA 1810014F10 gene /DEF=Mus musculus 10 day old male pancreas cDNA, RIKEN full-length enriched library, clone:1810014F10:hypothetical protein, full insert sequence.	3619.5999	929.4744	2477.6335	72.28253	2.1216178	2.0	0.16791381	0.36129323
gb:NM_011289.1 /DB_XREF=gi:8567399 /GEN=Rpl27 /FEA=FLmRNA /CNT=260 /TID=Mm.28985.1 /TIER=FL+Stack /STK=127 /UG=Mm.28985 /LL=19942 /DEF=Mus musculus ribosomal protein L27 (Rpl27), mRNA. /PROD=ribosomal protein L27 /FL=gb:BC024366.1 gb:NM_011289.1 gb:AF214527.1	21329.068	4761.8315	15259.966	3353.0945	1.8049625	3.0	0.16883916	0.36157066
gb:BC027196.1 /DB_XREF=gi:20071133 /FEA=FLmRNA /CNT=203 /TID=Mm.34411.1 /TIER=FL+Stack /STK=128 /UG=Mm.34411 /DEF=Mus musculus, Similar to pyruvate dehydrogenase kinase, isoenzyme 1, clone MGC:28719 IMAGE:4458562, mRNA, complete cds. /PROD=Similar to pyruvate dehydrogenase kinase,isoenzyme 1 /FL=gb:BC027196.1	813.5	142.77159	1572.3334	606.1789	2.110487	2.0	0.16926411	0.3607789
gb:NM_009438.1 /DB_XREF=gi:7110730 /GEN=Rpl13a /FEA=FLmRNA /CNT=73 /TID=Mm.13020.1 /TIER=FL+Stack /STK=40 /UG=Mm.13020 /LL=22121 /DEF=Mus musculus ribosomal protein L13a (Rpl13a), mRNA. /PROD=ribosomal protein L13a /FL=gb:NM_009438.1	17476.0	4075.9622	12270.466	1285.9536	2.1095538	2.0	0.16937798	0.35933462
gb:BC012697.1 /DB_XREF=gi:15215192 /FEA=FLmRNA /CNT=299 /TID=Mm.144413.1 /TIER=FL+Stack /STK=139 /UG=Mm.144413 /LL=22228 /UG_GENE=Ucp2 /DEF=Mus musculus, Similar to uncoupling protein 2, mitochondrial, clone MGC:13955 IMAGE:4205625, mRNA, complete cds. /PROD=Similar to uncoupling protein 2, mitochondrial /FL=gb:BC012967.1 gb:BC012697.1 gb:AF111999.1 gb:AF111998.1 gb:U69135.1 gb:NM_011671.1 gb:U94593.1	1638.2001	268.19226	1130.7334	408.5851	1.7984086	3.0	0.16995057	0.35887238
gb:NM_010212.1 /DB_XREF=gi:6753865 /GEN=Fhl2 /FEA=FLmRNA /CNT=37 /TID=Mm.6799.1 /TIER=FL+Stack /STK=12 /UG=Mm.6799 /LL=14200 /DEF=Mus musculus four and a half LIM domains 2 (Fhl2), mRNA. /PROD=four and a half LIM domains 2 /FL=gb:NM_010212.1 gb:AF153340.1 gb:AF114381.1 gb:U77040.2 gb:BC021468.1 gb:AF055889.1	24918.4	5382.6875	18191.166	3614.4695	1.7971222	3.0	0.17016971	0.35767153
gb:M25487.1 /DB_XREF=gi:556309 /GEN=His2b /FEA=mRNA /CNT=1 /TID=Mm.21579.3 /TIER=ConsEnd /STK=0 /UG=Mm.21579 /LL=68024 /UG_TITLE=H2B histone family, member S /DEF=Mus musculus histone H2b mRNA, 3 end.	8434.167	1950.1221	5890.8667	773.1769	2.0998752	2.0	0.17056525	0.35685077
gb:BC019836.1 /DB_XREF=gi:18043722 /FEA=FLmRNA /CNT=420 /TID=Mm.22248.2 /TIER=FL+Stack /STK=125 /UG=Mm.22248 /LL=16010 /UG_GENE=Igfbp4 /DEF=Mus musculus, insulin-like growth factor binding protein 4, clone MGC:29917 IMAGE:5123738, mRNA, complete cds. /PROD=insulin-like growth factor binding protein 4 /FL=gb:BC019836.1	5639.267	1676.5708	3555.6335	1113.3978	1.7931857	3.0	0.17084232	0.35579088
gb:BC002055.1 /DB_XREF=gi:12805192 /FEA=FLmRNA /CNT=194 /TID=Mm.1508.1 /TIER=FL+Stack /STK=73 /UG=Mm.1508 /LL=12257 /UG_GENE=Bzrp /DEF=Mus musculus, Similar to benzodiazepine receptor, peripheral, clone MGC:6086 IMAGE:3493196, mRNA, complete cds. /PROD=Similar to benzodiazepine receptor, peripheral /FL=gb:NM_009775.1 gb:L17306.1 gb:BC002055.1 gb:D21207.1	1181.9333	402.33136	650.36664	178.55083	2.091686	2.0	0.17157866	0.35569274
gb:NM_023202.1 /DB_XREF=gi:12963570 /GEN=Ndufa7 /FEA=FLmRNA /CNT=13 /TID=Mm.29513.1 /TIER=FL /STK=0 /UG=Mm.29513 /LL=66416 /DEF=Mus musculus NADH dehydrogenase (ubiquinone) 1 alpha subcomplex, 7 (14.5kD, B14.5a) (Ndufa7), mRNA. /PROD=NADH dehydrogenase (ubiquinone) 1 alphasubcomplex, 7 (14.5kD, B14.5a) /FL=gb:NM_023202.1	14773.0	3598.2625	10314.233	2397.5046	1.7861034	3.0	0.17206016	0.3550696
gb:NM_008761.2 /DB_XREF=gi:11612666 /GEN=Fxyd5 /FEA=FLmRNA /CNT=58 /TID=Mm.1870.1 /TIER=FL+Stack /STK=26 /UG=Mm.1870 /LL=18301 /DEF=Mus musculus FXYD domain-containing ion transport regulator 5 (Fxyd5), mRNA. /PROD=related to ion channel /FL=gb:U72680.1 gb:NM_008761.2	1579.3999	339.63223	955.8667	502.16968	1.7814642	3.0	0.17286329	0.35511282
gb:NM_025963.1 /DB_XREF=gi:13399309 /GEN=2210402A09Rik /FEA=FLmRNA /CNT=181 /TID=Mm.39130.1 /TIER=FL+Stack /STK=71 /UG=Mm.39130 /LL=67097 /DEF=Mus musculus RIKEN cDNA 2210402A09 gene (2210402A09Rik), mRNA. /PROD=RIKEN cDNA 2210402A09 /FL=gb:BC019725.1 gb:BC003853.1 gb:NM_025963.1	24991.568	5849.624	17276.7	2730.6064	2.0699267	2.0	0.17431144	0.35647476
gb:BB504983 /DB_XREF=gi:9493777 /DB_XREF=BB504983 /CLONE=D630050N19 /FEA=EST /CNT=35 /TID=Mm.30245.4 /TIER=Stack /STK=34 /UG=Mm.30245 /UG_TITLE=Mus musculus, Similar to phosphatidylserine decarboxylase, clone MGC:7133 IMAGE:3158145, mRNA, complete cds	1228.9	481.8121	1871.4332	403.02145	1.7717181	3.0	0.17456463	0.35539168
gb:NM_023202.1 /DB_XREF=gi:12963570 /GEN=Ndufa7 /FEA=FLmRNA /CNT=13 /TID=Mm.29513.1 /TIER=FL /STK=0 /UG=Mm.29513 /LL=66416 /DEF=Mus musculus NADH dehydrogenase (ubiquinone) 1 alpha subcomplex, 7 (14.5kD, B14.5a) (Ndufa7), mRNA. /PROD=NADH dehydrogenase (ubiquinone) 1 alphasubcomplex, 7 (14.5kD, B14.5a) /FL=gb:NM_023202.1	12473.101	4055.233	7557.9	2600.8333	1.7671416	3.0	0.17537016	0.35543773
gb:NM_007472.1 /DB_XREF=gi:6680709 /GEN=Aqp1 /FEA=FLmRNA /CNT=262 /TID=Mm.18625.1 /TIER=FL+Stack /STK=113 /UG=Mm.18625 /LL=11826 /DEF=Mus musculus aquaporin 1 (Aqp1), mRNA. /PROD=aquaporin 1 /FL=gb:NM_007472.1 gb:BC007125.1 gb:L02914.1	9189.899	2563.1602	5969.3003	1845.5371	1.7661334	3.0	0.17554818	0.35421723
gb:NM_007632.1 /DB_XREF=gi:6724312 /GEN=Ccnd3 /FEA=FLmRNA /CNT=286 /TID=Mm.7417.1 /TIER=FL+Stack /STK=172 /UG=Mm.7417 /LL=12445 /DEF=Mus musculus cyclin D3 (Ccnd3), mRNA. /PROD=cyclin D3 /FL=gb:BC004076.1 gb:NM_007632.1 gb:M86183.1 gb:BC005605.1	1193.2001	406.31543	681.76666	146.32567	2.051192	2.0	0.17671187	0.35498756
gb:NM_134118.1 /DB_XREF=gi:19923069 /GEN=AI173355 /FEA=FLmRNA /CNT=229 /TID=Mm.142511.1 /TIER=FL+Stack /STK=95 /UG=Mm.142511 /LL=106529 /DEF=Mus musculus expressed sequence AI173355 (AI173355), mRNA. /PROD=synaptic glycoprotein SC2 /FL=gb:NM_134118.1 gb:BC019984.1	4182.3667	1073.5255	2884.2668	694.285	1.7586433	3.0	0.17687732	0.35375464
gb:NM_025569.1 /DB_XREF=gi:13385009 /GEN=Mgst3 /FEA=FLmRNA /CNT=216 /TID=Mm.29823.1 /TIER=FL+Stack /STK=98 /UG=Mm.29823 /LL=66447 /DEF=Mus musculus microsomal glutathione S-transferase 3 (Mgst3), mRNA. /PROD=microsomal glutathione S-transferase 3 /FL=gb:NM_025569.1	5083.5	1483.3075	3071.2334	1323.4027	1.7533147	3.0	0.17782989	0.35409987
gb:BB433489 /DB_XREF=gi:9273216 /DB_XREF=BB433489 /CLONE=C820001M03 /FEA=EST /CNT=10 /TID=Mm.217102.3 /TIER=Stack /STK=8 /UG=Mm.217102 /UG_TITLE=Mus musculus, clone MGC:25836 IMAGE:4190175, mRNA, complete cds	1077.0333	378.58313	1656.7334	431.17288	1.7498903	3.0	0.17844518	0.35377342
gb:BM225280 /DB_XREF=gi:17786807 /DB_XREF=K0209C02-3 /CLONE=K0209C02 /FEA=FLmRNA /CNT=29 /TID=Mm.65655.1 /TIER=Stack /STK=13 /UG=Mm.65655 /UG_TITLE=Mus musculus, clone MGC:35978 IMAGE:5353641, mRNA, complete cds /FL=gb:BC021527.1	651.3333	209.51527	1085.6	375.84653	1.7480214	3.0	0.178782	0.35290012
gb:NM_007932.1 /DB_XREF=gi:6679648 /GEN=Eng /FEA=FLmRNA /CNT=113 /TID=Mm.4851.1 /TIER=FL+Stack /STK=50 /UG=Mm.4851 /LL=13805 /DEF=Mus musculus endoglin (Eng), mRNA. /PROD=endoglin /FL=gb:NM_007932.1	2786.6335	1294.6776	1264.8334	141.4751	2.0238533	2.0	0.18029575	0.3543475

gb:NM_008322.1 /DB_XREF=gi:6680342 /GEN=Idh2 /FEA=FLmRNA /CNT=70 /TID=Mm.2966.1 /TIER=FL /STK=2 /UG=Mm.2966 /LL=15927 /DEF=Mus musculus isocitrate dehydrogenase 2 (NADP+), mitochondrial (Idh2), mRNA. /PROD=isocitrate dehydrogenase 2 (NADP+),mitochondrial /FL=gb:NM_008322.1 gb:U51167.1	11169.633	2583.2095	7453.2993	2658.36	1.736538	3.0	0.1808676	0.35393918
gb:NM_133950.1 /DB_XREF=gi:19527169 /GEN=8030486F04Rik /FEA=FLmRNA /CNT=142 /TID=Mm.25045.1 /TIER=FL+Stack /STK=40 /UG=Mm.25045 /LL=68137 /DEF=Mus musculus RIKEN cDNA 8030486F04 gene (8030486F04Rik), mRNA. /PROD=putative KDEL receptor /FL=gb:BC011370.1 gb:NM_133950.1	1906.1666	557.6326	1163.2	488.95154	1.7351526	3.0	0.1811211	0.3529141
gb:NM_007475.1 /DB_XREF=gi:6671568 /GEN=Arbp /FEA=FLmRNA /CNT=149 /TID=Mm.5286.1 /TIER=FL+Stack /STK=9 /UG=Mm.5286 /LL=11837 /DEF=Mus musculus acidic ribosomal phosphoprotein PO (Arbp), mRNA. /PROD=acidic ribosomal phosphoprotein PO /FL=gb:NM_007475.1 gb:BC003833.1 gb:BC011106.1 gb:BC011291.1	24874.898	5400.472	17917.0	2557.1738	2.0168743	2.0	0.18122636	0.3516101
gb:NM_024166.2 /DB_XREF=gi:18079333 /GEN=Etohi6 /FEA=FLmRNA /CNT=20 /TID=Mm.30133.1 /TIER=FL /STK=0 /UG=Mm.30133 /LL=14004 /DEF=Mus musculus ethanol induced 6 (Etohi6), mRNA. /PROD=ethanol induced 6 /FL=gb:NM_024166.2	14814.734	4323.9595	9379.066	1765.9126	2.0157433	2.0	0.1813778	0.35040647
gb:NM_023167.1 /DB_XREF=gi:12963548 /GEN=Mrpl4 /FEA=FLmRNA /CNT=140 /TID=Mm.155033.1 /TIER=FL+Stack /STK=84 /UG=Mm.155033 /LL=66163 /DEF=Mus musculus mitochondrial ribosomal protein L4 (Mrpl4), mRNA. /PROD=RIKEN cDNA 1110017G11 /FL=gb:NM_023167.1 gb:AB049634.1	3499.3665	784.99615	2496.5332	365.56644	2.0058568	2.0	0.18270874	0.35148206
gb:BC006928.1 /DB_XREF=gi:13905259 /FEA=FLmRNA /CNT=146 /TID=Mm.29878.1 /TIER=FL+Stack /STK=74 /UG=Mm.29878 /LL=74158 /UG_GENE=1300006C06Rik /DEF=Mus musculus, RIKEN cDNA 1300006C06 gene, clone MGC:6306 IMAGE:2655826, mRNA, complete cds. /PROD=RIKEN cDNA 1300006C06 gene /FL=gb:BC006928.1	1356.2333	376.64792	917.5334	227.8885	1.7260557	3.0	0.18279569	0.35016558
gb:BB311034 /DB_XREF=gi:16402667 /DB_XREF=BB311034 /CLONE=B230321B18 /FEA=FLmRNA /CNT=290 /TID=Mm.194045.1 /TIER=Stack /STK=161 /UG=Mm.194045 /UG_TITLE=Mus musculus, clone MGC:36474 IMAGE:5361238, mRNA, complete cds /FL=gb:BC025804.1 gb:BC027056.1	2448.6667	768.1256	3768.6335	1084.3678	1.7204585	3.0	0.18383482	0.35067654
gb:NM_026341.1 /DB_XREF=gi:13385843 /GEN=4933433B15Rik /FEA=FLmRNA /CNT=6 /TID=Mm.41966.1 /TIER=FL /STK=1 /UG=Mm.41966 /LL=67725 /DEF=Mus musculus RIKEN cDNA 4933433B15 gene (4933433B15Rik), mRNA. /PROD=RIKEN cDNA 4933433B15 /FL=gb:NM_026341.1	754.6667	307.8523	1166.3334	280.70532	1.7114762	3.0	0.1855165	0.35240373
gb:NM_054098.1 /DB_XREF=gi:16905132 /GEN=Tiarp-pending /FEA=FLmRNA /CNT=68 /TID=Mm.31403.1 /TIER=FL+Stack /STK=60 /UG=Mm.31403 /LL=117167 /DEF=Mus musculus Tnfa-induced adipose-related protein (Tiarp-pending), mRNA. /PROD=Tnfa-induced adipose-related protein /FL=gb:NM_054098.1	2168.6333	604.4637	1476.2666	43.419083	1.9788327	2.0	0.18641466	0.3526344
gb:AF004833.1 /DB_XREF=gi:2897076 /GEN=TFPI /FEA=FLmRNA /CNT=10 /TID=Mm.3601.1 /TIER=FL /STK=1 /UG=Mm.3601 /LL=21788 /DEF=Mus musculus tissue factor pathway inhibitor (TFPI) mRNA, complete cds. /PROD=tissue factor pathway inhibitor /FL=gb:AF004833.1	1606.7666	382.47437	1031.9667	440.50238	1.7065847	3.0	0.18643968	0.3512183
gb:AF361882.1 /DB_XREF=gi:13991772 /GEN=Esam /FEA=FLmRNA /CNT=73 /TID=Mm.41751.1 /TIER=FL+Stack /STK=39 /UG=Mm.41751 /LL=69524 /DEF=Mus musculus endothelial cell-selective adhesion molecule (Esam) mRNA, complete cds. /PROD=endothelial cell-selective adhesion molecule /FL=gb:AF361882.1	2765.1667	719.87036	1949.3002	409.42258	1.7063507	3.0	0.18648396	0.3498501
gb:NM_009949.1 /DB_XREF=gi:6753513 /GEN=Cpt2 /FEA=FLmRNA /CNT=145 /TID=Mm.29499.1 /TIER=FL+Stack /STK=70 /UG=Mm.29499 /LL=12896 /DEF=Mus musculus carnitine palmitoyltransferase 2 (Cpt2), mRNA. /PROD=carnitine palmitoyltransferase 2 /FL=gb:U01163.1 gb:NM_009949.1	6123.467	1516.8256	4317.567	480.7747	1.9657608	2.0	0.18824361	0.35169792
gb:NM_015751.1 /DB_XREF=gi:7657517 /GEN=Abce1 /FEA=FLmRNA /CNT=245 /TID=Mm.5831.1 /TIER=FL+Stack /STK=145 /UG=Mm.5831 /LL=24015 /DEF=Mus musculus ATP-binding cassette, sub-family E (OABP), member 1 (Abce1), mRNA. /PROD=ATP-binding cassette, subfamily E, member 1 /FL=gb:NM_015751.1 gb:U90446.1 gb:BC005422.1	1349.0668	637.7267	2110.8	209.44952	1.9655546	2.0	0.18827267	0.35031062
gb:BC016492.1 /DB_XREF=gi:16741319 /FEA=FLmRNA /CNT=172 /TID=Mm.18843.1 /TIER=FL+Stack /STK=43 /UG=Mm.18843 /LL=21817 /UG_GENE=Tgm2 /DEF=Mus musculus, transglutaminase 2, C polypeptide, clone MGC:6152 IMAGE:3256943, mRNA, complete cds. /PROD=transglutaminase 2, C polypeptide /FL=gb:NM_009373.1 gb:AF076928.1 gb:BC016492.1	6511.0	2017.0101	4233.3	1157.5739	1.6963931	3.0	0.18837993	0.34907955
gb:NM_013868.1 /DB_XREF=gi:7305170 /GEN=Hsp25-2 /FEA=FLmRNA /CNT=15 /TID=Mm.46181.1 /TIER=FL /STK=2 /UG=Mm.46181 /LL=29818 /DEF=Mus musculus heat shock protein 25 kDa 2 (cardiovascular) (Hsp25-2), mRNA. /PROD=heat shock protein 25 kDa 2 (cardiovascular) /FL=gb:AF155909.1 gb:NM_013868.1	2128.9001	1409.7396	512.4666	235.13553	1.9589396	2.0	0.18920761	0.34918803
gb:U60001.1 /DB_XREF=gi:1519045 /GEN=mPKCI /FEA=FLmRNA /CNT=127 /TID=Mm.425.1 /TIER=FL+Stack /STK=62 /UG=Mm.425 /LL=15254 /DEF=Mus musculus protein kinase C inhibitor (mPKCI) mRNA, complete cds. /PROD=protein kinase C inhibitor /FL=gb:U60001.1	19355.8	4637.8774	14079.034	533.226	1.957752	2.0	0.18937612	0.34808406
gb:AF017174.2 /DB_XREF=gi:16950577 /FEA=FLmRNA /CNT=55 /TID=Mm.34881.1 /TIER=FL+Stack /STK=25 /UG=Mm.34881 /LL=12895 /UG_GENE=Cpt1b /DEF=Mus musculus carnitine palmitoyltransferase I mRNA, complete cds. /PROD=carnitine palmitoyltransferase I /FL=gb:AF017174.2 gb:NM_009948.1 gb:BC018270.1 gb:AB010826.1	2422.7666	256.9645	1728.7001	557.9454	1.9570363	2.0	0.18947777	0.34686655
gb:AB057663.2 /DB_XREF=gi:18157433 /GEN=cRAF /FEA=FLmRNA /CNT=3 /TID=Mm.22467.2 /TIER=FL /STK=2 /UG=Mm.22467 /LL=110157 /DEF=Mus musculus cRAF mRNA for protein kinase raf 1, complete cds. /PROD=protein kinase raf 1 /FL=gb:AB057663.2	2376.9666	584.58136	1679.4335	199.08739	1.9563724	2.0	0.18957213	0.34564555
gb:AK003566.1 /DB_XREF=gi:12834309 /GEN=Asb2 /FEA=mRNA /CNT=56 /TID=Mm.27159.1 /TIER=Stack /STK=42 /UG=Mm.27159 /LL=65256 /UG_TITLE=ankyrin repeat and SOCS box-containing protein 2 /DEF=Mus musculus 18 days embryo whole body cDNA, RIKEN full-length enriched library, clone:1110008E15:ankyrin repeat and SOCS box-containing protein 2, full insert sequence.	8086.7007	2274.8245	5422.0337	631.6684	1.9549096	2.0	0.18978025	0.34464094
gb:NM_011296.1 /DB_XREF=gi:6755367 /GEN=Rps18 /FEA=FLmRNA /CNT=56 /TID=Mm.42790.1 /TIER=FL+Stack /STK=43 /UG=Mm.42790 /LL=20084 /DEF=Mus musculus ribosomal protein S18 (Rps18), mRNA. /PROD=ribosomal protein S18 /FL=gb:NM_011296.1 gb:M76763.1	22368.432	5003.2515	16096.301	2429.0657	1.9532846	2.0	0.1900118	0.3436867
gb:NM_138583.1 /DB_XREF=gi:20070387 /GEN=D16H22S680E /FEA=FLmRNA /CNT=59 /TID=Mm.27502.1 /TIER=FL+Stack /STK=33 /UG=Mm.27502 /LL=27883 /DEF=Mus musculus DNA segment, Chr 16, human D22S680E, expressed (D16H22S680E), mRNA. /PROD=DNA segment, Chr 16, human D22S680E, expressed /FL=gb:NM_138583.1 gb:BC005445.1	2440.9	520.3055	1791.8667	247.71368	1.950771	2.0	0.19037074	0.34296948
gb:NM_018860.1 /DB_XREF=gi:9256635 /GEN=Rpl41 /FEA=FLmRNA /CNT=37 /TID=Mm.13859.1 /TIER=FL+Stack /STK=25 /UG=Mm.13859 /LL=67945 /DEF=Mus musculus ribosomal protein L41 (Rpl41), mRNA. /PROD=ribosomal protein L41 /FL=gb:NM_018860.1 gb:U93862.1	18386.701	5389.7344	11991.466	1807.8418	1.9484895	2.0	0.19069731	0.34219992
gb:NM_026353.1 /DB_XREF=gi:13385855 /GEN=4930570C03Rik /FEA=FLmRNA /CNT=27 /TID=Mm.28955.1 /TIER=FL /STK=1 /UG=Mm.28955 /LL=67739 /DEF=Mus musculus RIKEN cDNA 4930570C03 gene (4930570C03Rik), mRNA. /PROD=RIKEN cDNA 4930570C03 /FL=gb:NM_026353.1 gb:BC022913.1	1148.1333	399.32288	666.1333	160.61934	1.9396348	2.0	0.19197194	0.34313095
gb:NM_011295.1 /DB_XREF=gi:6755365 /GEN=Rps12 /FEA=FLmRNA /CNT=156 /TID=Mm.21289.1 /TIER=FL+Stack /STK=88 /UG=Mm.21289 /LL=20042 /DEF=Mus musculus ribosomal protein S12 (Rps12), mRNA. /PROD=ribosomal protein S12 /FL=gb:BC002079.1 gb:BC018362.1 gb:NM_011295.1	14910.867	4813.6387	9274.433	1604.0054	1.9240993	2.0	0.19423601	0.34581628
gb:NM_007506.1 /DB_XREF=gi:6680749 /GEN=Atp5g1 /FEA=FLmRNA /CNT=253 /TID=Mm.258.1 /TIER=FL+Stack /STK=143 /UG=Mm.258 /LL=11951 /DEF=Mus musculus ATP synthase, H+ transporting, mitochondrial F0 complex, subunit c (subunit 9), isoform 1 (Atp5g1), mRNA. /PROD=ATP synthase, H+ transporting, mitochondrial F0complex, subunit c (subunit 9), isoform 1 /FL=gb:NM_007506.1 gb:L19737.1 gb:BC003854.1	31173.766	9255.258	20473.766	2719.352	1.9212122	2.0	0.1946607	0.3452186
gb:NM_007918.1 /DB_XREF=gi:6681294 /GEN=Eif4ebp1 /FEA=FLmRNA /CNT=114 /TID=Mm.6700.1 /TIER=FL+Stack /STK=41 /UG=Mm.6700 /LL=13685 /DEF=Mus musculus eukaryotic translation initiation factor 4E binding protein 1 (Eif4ebp1), mRNA. /PROD=eukaryotic translation initiation factor 4Ebinding protein 1 /FL=gb:U28656.1 gb:NM_007918.1 gb:BC002045.1	3291.5999	586.9502	2234.0	935.8051	1.6582847	3.0	0.19584058	0.3459596
gb:BC019836.1 /DB_XREF=gi:18043722 /FEA=FLmRNA /CNT=420 /TID=Mm.22248.2 /TIER=FL+Stack /STK=125 /UG=Mm.22248 /LL=16010 /UG_GENE=Igfbp4 /DEF=Mus musculus, insulin-like growth factor binding protein 4, clone MGC:29917 IMAGE:5123738, mRNA, complete cds. /PROD=insulin-like growth factor binding protein 4 /FL=gb:BC019836.1	3464.2	1083.6925	2282.7666	594.74207	1.6553617	3.0	0.1964265	0.34564972
gb:NM_010015.1 /DB_XREF=gi:6753597 /GEN=Dad1 /FEA=FLmRNA /CNT=36 /TID=Mm.2547.1 /TIER=FL+Stack /STK=22 /UG=Mm.2547 /LL=13135 /DEF=Mus musculus defender against cell death 1 (Dad1), mRNA. /PROD=defender against cell death 1 /FL=gb:NM_010015.1 gb:U83628.1 gb:BC024378.1	2949.5334	784.35864	2008.8999	596.2382	1.6536152	3.0	0.19677751	0.34493047
gb:NM_009840.1 /DB_XREF=gi:6753327 /GEN=Cct8 /FEA=FLmRNA /CNT=482 /TID=Mm.17989.1 /TIER=FL+Stack /STK=281 /UG=Mm.17989 /LL=12469 /DEF=Mus musculus chaperonin subunit 8 (theta) (Cct8), mRNA. /PROD=chaperonin subunit 8 (theta) /FL=gb:NM_009840.1 gb:BC009007.1	1954.2334	813.74225	2928.2002	348.0841	1.9060308	2.0	0.1969144	0.34384286
gb:BM117243 /DB_XREF=gi:17080261 /DB_XREF=L0848C02-3 /CLONE=L0848C02 /FEA=mRNA /CNT=252 /TID=Mm.29308.1 /TIER=ConsEnd /STK=0 /UG=Mm.29308 /UG_TITLE=Mus musculus, Similar to chromosome 20 open reading frame 36, clone IMAGE:5356821, mRNA, partial cds	1115.3667	606.996	1858.0	489.62155	1.6493825	3.0	0.1976312	0.3437723
gb:NM_023423.1 /DB_XREF=gi:12963672 /GEN=6330407G11Rik /FEA=FLmRNA /CNT=142 /TID=Mm.27903.1 /TIER=FL+Stack /STK=64 /UG=Mm.27903 /LL=68050 /DEF=Mus musculus RIKEN cDNA 6330407G11 gene (6330407G11Rik), mRNA. /PROD=RIKEN cDNA 6330407G11 /FL=gb:BC003291.1 gb:NM_023423.1	767.13336	60.56274	1230.6333	419.40765	1.8944916	2.0	0.1986508	0.34422696
"gb:BC011117.1 /DB_XREF=gi:15029791 /FEA=FLmRNA /CNT=173 /TID=Mm.44202.1 /TIER=FL+Stack /STK=75 /UG=Mm.44202 /LL=68090 /UG_GENE=5330422J23Rik /DEF=Mus musculus, Similar to putative transmembrane protein; homolog of yeast Golgi membrane protein Yif1p (Yip1p-interacting factor), clone MGC:18791 IMAGE:4190535, mRNA, complete cds. /PROD=Similar to putative transmembrane protein;homolog of yeast Golgi membrane protein Yif1p(Yip1p-interacting factor) /FL=gb:BC011117.1"	3100.8997	796.23926	2096.9	699.3586	1.6409115	3.0	0.19935222	0.34412894
gb:BC028550.1 /DB_XREF=gi:20306503 /FEA=mRNA /CNT=8 /TID=Mm.144300.1 /TIER=ConsEnd /STK=0 /UG=Mm.144300 /DEF=Mus musculus, H4 histone family, member A, clone MGC:41466 IMAGE:1398106, mRNA, complete cds. /PROD=H4 histone family, member A	1164.7999	442.33972	648.8333	169.54597	1.8865175	2.0	0.19986269	0.34370327
gb:NM_018853.1 /DB_XREF=gi:9256518 /GEN=Rplp1 /FEA=FLmRNA /CNT=193 /TID=Mm.3158.1 /TIER=FL+Stack /STK=102 /UG=Mm.3158 /LL=56040 /DEF=Mus musculus ribosomal protein, large, P1 (Rplp1), mRNA. /PROD=ribosomal protein, large, P1 /FL=gb:NM_018853.1 gb:U29402.1	22579.867	5383.7686	16234.766	2247.2065	1.8838094	2.0	0.2002765	0.3431152
gb:NM_010833.1 /DB_XREF=gi:6754749 /GEN=Msn /FEA=FLmRNA /CNT=271 /TID=Mm.28687.1 /TIER=FL /STK=1 /UG=Mm.28687 /LL=17698 /DEF=Mus musculus moesin (Msn), mRNA. /PROD=moesin /FL=gb:NM_010833.1	977.89996	476.53815	421.33334	187.60748	1.8823091	2.0	0.20050626	0.34221742
gb:AY030292.1 /DB_XREF=gi:17223645 /GEN=Gtf2i /FEA=FLmRNA /CNT=21 /TID=Mm.22593.3 /TIER=FL /STK=1 /UG=Mm.22593 /LL=14886 /DEF=Mus musculus transcription factor TFII-I-delta (Gtf2i) mRNA, complete cds, alternatively spliced. /PROD=transcription factor TFII-I-delta /FL=gb:AY030292.1 gb:AF043220.1	2074.7666	556.9498	1469.0333	325.74814	1.6260589	3.0	0.20241062	0.34417385
gb:BC024696.1 /DB_XREF=gi:19353040 /FEA=FLmRNA /CNT=38 /TID=Mm.35837.1 /TIER=FL /STK=7 /UG=Mm.35837 /LL=76799 /UG_GENE=2510006D16Rik /DEF=Mus musculus, RIKEN cDNA 2510006D16 gene, clone MGC:19069 IMAGE:4193049, mRNA, complete cds. /PROD=RIKEN cDNA 2510006D16 gene /FL=gb:BC024696.1	2058.7	425.93628	1417.5668	534.88104	1.624086	3.0	0.20282084	0.34358457
gb:NM_008161.1 /DB_XREF=gi:6680076 /GEN=Gpx3 /FEA=FLmRNA /CNT=307 /TID=Mm.7156.1 /TIER=FL+Stack /STK=22 /UG=Mm.7156 /LL=14778 /DEF=Mus musculus glutathione peroxidase 3 (Gpx3), mRNA. /PROD=glutathione peroxidase 3 /FL=gb:U13705.1 gb:NM_008161.1	19679.033	4879.123	13987.534	2023.1467	1.8663505	2.0	0.20297192	0.3425623
gb:NM_025508.1 /DB_XREF=gi:17975499 /GEN=Gmpr /FEA=FLmRNA /CNT=110 /TID=Mm.25808.1 /TIER=FL+Stack /STK=63 /UG=Mm.25808 /LL=66355 /DEF=Mus musculus guanosine monophosphate reductase (Gmpr), mRNA. /PROD=guanosine monophosphate reductase /FL=gb:BC010827.1 gb:NM_025508.1	2275.5999	528.1235	1673.6	182.65222	1.8658966	2.0	0.20304264	0.34141245
gb:NM_010286.1 /DB_XREF=gi:6753985 /GEN=Gilz /FEA=FLmRNA /CNT=5 /TID=Mm.22216.1 /TIER=FL /STK=3 /UG=Mm.22216 /LL=14605 /DEF=Mus musculus glucocorticoid-induced leucine zipper (Gilz), mRNA. /PROD=glucocorticoid-induced leucine zipper /FL=gb:AF024519.1 gb:NM_010286.1	1798.2334	487.1003	1107.8667	554.0929	1.620791	3.0	0.20350805	0.34093228
gb:BC021616.1 /DB_XREF=gi:18204464 /FEA=FLmRNA /CNT=122 /TID=Mm.44227.1 /TIER=FL+Stack /STK=81 /UG=Mm.44227 /DEF=Mus musculus, clone MGC:37950 IMAGE:5132866, mRNA, complete cds. /PROD=Unknown (protein for MGC:37950) /FL=gb:BC021616.1	12563.3	3060.4165	8879.767	1558.9458	1.8575878	2.0	0.20434298	0.34107247
gb:BC010238.1 /DB_XREF=gi:16307390 /FEA=FLmRNA /CNT=195 /TID=Mm.196533.1 /TIER=FL+Stack /STK=69 /UG=Mm.196533 /LL=66940 /UG_GENE=2310008D10Rik /DEF=Mus musculus, RIKEN cDNA 2310008D10 gene, clone MGC:7872 IMAGE:3581921, mRNA, complete cds. /PROD=RIKEN cDNA 2310008D10 gene /FL=gb:BC010238.1	1952.7334	562.42065	1314.1333	389.6318	1.6166142	3.0	0.20438288	0.3398895
gb:AK017277.1 /DB_XREF=gi:12856436 /FEA=mRNA /CNT=21 /TID=Mm.103495.1 /TIER=Stack /STK=10 /UG=Mm.103495 /LL=71324 /UG_GENE=5430405N12Rik /UG_TITLE=RIKEN cDNA 5430405N12 gene /DEF=Mus musculus 6 days neonate head cDNA, RIKEN full-length enriched library, clone:5430405N12:hypothetical protein, full insert sequence.	1315.7	752.4374	2206.2	358.13565	1.8508978	2.0	0.20539802	0.34033105
gb:NM_011668.1 /DB_XREF=gi:7106440 /GEN=Ube3a /FEA=FLmRNA /CNT=174 /TID=Mm.9002.1 /TIER=FL+Stack /STK=74 /UG=Mm.9002 /LL=22215 /DEF=Mus musculus ubiquitin protein ligase E3A (Ube3a), mRNA. /PROD=ubiquitin protein ligase E3A /FL=gb:U82122.1 gb:U96636.1 gb:NM_011668.1	2141.9333	729.3072	3311.8	1026.2908	1.6093861	3.0	0.20590682	0.33993343
gb:NM_134101.1 /DB_XREF=gi:19882200 /GEN=Psmd2 /FEA=FLmRNA /CNT=415 /TID=Mm.147697.1 /TIER=FL+Stack /STK=223 /UG=Mm.147697 /LL=21762 /DEF=Mus musculus proteasome (prosome, macropain) 26S subunit, non-ATPase, 2 (Psmd2), mRNA. /PROD=proteasome (prosome, macropain) 26S subunit,non-ATPase, 2 /FL=gb:BC021508.1 gb:NM_134101.1 gb:BC027388.1 gb:BC024830.1	4122.5	1210.1099	2827.2998	694.2559	1.6080008	3.0	0.20620035	0.3391846
gb:BC021536.1 /DB_XREF=gi:18204854 /FEA=FLmRNA /CNT=112 /TID=Mm.41683.1 /TIER=FL+Stack /STK=60 /UG=Mm.41683 /LL=106264 /UG_GENE=0610012G03Rik /DEF=Mus musculus, Similar to RIKEN cDNA 0610012G03 gene, clone MGC:35773 IMAGE:5352549, mRNA, complete cds. /PROD=Similar to RIKEN cDNA 0610012G03 gene /FL=gb:BC021536.1 gb:NM_025320.1	6951.5664	2023.6892	4687.8667	675.45154	1.8378059	2.0	0.20748371	0.34006354
gb:NM_008738.1 /DB_XREF=gi:6679135 /GEN=Nrtn /FEA=FLmRNA /CNT=31 /TID=Mm.8074.1 /TIER=FL+Stack /STK=16 /UG=Mm.8074 /LL=18188 /DEF=Mus musculus neurturin (Nrtn), mRNA. /PROD=neurturin /FL=gb:NM_008738.1 gb:U78109.1	1260.7999	561.4893	664.8	20.29703	1.8373072	2.0	0.20756371	0.33897096

gb:NM_011072.1 /DB_XREF=gi:6755039 /GEN=Pfn1 /FEA=FLmRNA /CNT=168 /TID=Mm.2647.1 /TIER=FL+Stack /STK=26 /UG=Mm.2647 /LL=18643 /DEF=Mus musculus profilin 1 (Pfn1), mRNA. /PROD=profilin 1 /FL=gb:BC002080.1 gb:NM_011072.1	1880.2666	486.36215	1148.6666	624.1759	1.6013907	3.0	0.20760737	0.33782706
gb:BC019131.1 /DB_XREF=gi:17512319 /FEA=FLmRNA /CNT=89 /TID=Mm.76694.1 /TIER=FL+Stack /STK=38 /UG=Mm.76694 /LL=68864 /UG_GENE=1110067D22Rik /DEF=Mus musculus, clone MGC:29165 IMAGE:5036749, mRNA, complete cds. /PROD=Unknown (protein for MGC:29165) /FL=gb:BC019131.1	1780.6666	942.588	2963.0667	868.2394	1.5980754	3.0	0.20831712	0.33777133
gb:NM_025650.1 /DB_XREF=gi:13385111 /GEN=0710008D09Rik /FEA=FLmRNA /CNT=200 /TID=Mm.43162.1 /TIER=FL+Stack /STK=100 /UG=Mm.43162 /LL=66594 /DEF=Mus musculus RIKEN cDNA 0710008D09 gene (0710008D09Rik), mRNA. /PROD=RIKEN cDNA 0710008D09 /FL=gb:NM_025650.1	17802.533	6000.5195	10966.7	2426.666	1.8292425	2.0	0.20886318	0.33745155
gb:NM_023119.1 /DB_XREF=gi:12963490 /GEN=Eno1 /FEA=FLmRNA /CNT=270 /TID=Mm.90587.1 /TIER=FL+Stack /STK=14 /UG=Mm.90587 /LL=13806 /DEF=Mus musculus enolase 1, alpha non-neuron (Eno1), mRNA. /PROD=enolase 1, alpha non-neuron /FL=gb:BC024644.1 gb:BC003891.1 gb:BC010685.1 gb:NM_023119.1	6644.033	2078.6033	4233.8335	1600.5298	1.5912825	3.0	0.20977983	0.33773065
gb:BC016534.1 /DB_XREF=gi:16741429 /FEA=FLmRNA /CNT=23 /TID=Mm.144699.2 /TIER=FL+Stack /STK=8 /UG=Mm.144699 /LL=56409 /UG_GENE=Nudt3 /DEF=Mus musculus, nudix (nucleotide diphosphate linked moiety X)-type motif 3, clone MGC:28998 IMAGE:2615904, mRNA, complete cds. /PROD=nudix (nucleotide diphosphate linked moietyX)-type motif 3 /FL=gb:BC016534.1	4924.8003	1210.6158	3560.6335	466.26718	1.8213212	2.0	0.21015003	0.33713117
gb:NM_007751.1 /DB_XREF=gi:6680994 /GEN=Cox8b /FEA=FLmRNA /CNT=27 /TID=Mm.3841.1 /TIER=FL+Stack /STK=18 /UG=Mm.3841 /LL=12869 /DEF=Mus musculus cytochrome c oxidase, subunit VIIIb (Cox8b), mRNA. /PROD=cytochrome c oxidase, subunit VIIIb /FL=gb:U15541.1 gb:NM_007751.1	30768.635	9435.1045	20126.934	3702.992	1.8185111	2.0	0.21060908	0.33667788
gb:AJ002387.1 /DB_XREF=gi:2598561 /FEA=mRNA /CNT=14 /TID=Mm.918.2 /TIER=ConsEnd /STK=0 /UG=Mm.918 /LL=14828 /UG_GENE=Hspa5 /DEF=Mus musculus mRNA for BiP. /PROD=BiP	3538.3337	1849.5023	5539.8667	464.41464	1.8179885	2.0	0.2106946	0.3356328
gb:NM_016808.1 /DB_XREF=gi:7949157 /GEN=Usp2 /FEA=FLmRNA /CNT=113 /TID=Mm.12914.1 /TIER=FL+Stack /STK=54 /UG=Mm.12914 /LL=53376 /DEF=Mus musculus ubiquitin specific protease 2 (Usp2), mRNA. /PROD=ubiquitin-specific protease 2 /FL=gb:NM_016808.1 gb:BC017517.1 gb:AF079565.1	3728.6335	542.1459	2686.4001	1003.7924	1.5823406	3.0	0.21172279	0.33609143
gb:NM_007899.1 /DB_XREF=gi:6681256 /GEN=Ecm1 /FEA=FLmRNA /CNT=126 /TID=Mm.3433.1 /TIER=FL+Stack /STK=53 /UG=Mm.3433 /LL=13601 /DEF=Mus musculus extracellular matrix protein 1 (Ecm1), mRNA. /PROD=extracellular matrix protein 1 /FL=gb:NM_007899.1 gb:L33416.1	2801.5664	1210.8658	1481.3999	358.34644	1.8107661	2.0	0.21188116	0.3351709
gb:AK002661.1 /DB_XREF=gi:12832810 /FEA=mRNA /CNT=77 /TID=Mm.27395.1 /TIER=Stack /STK=34 /UG=Mm.27395 /LL=76263 /UG_GENE=0610025I19Rik /UG_TITLE=RIKEN cDNA 0610025I19 gene /DEF=Mus musculus adult male kidney cDNA, RIKEN full-length enriched library, clone:0610025I19:homolog to GLUTATHIONE S-TRANSFERASE, MITOCHONDRIAL (EC 2.5.1.18) (GST 13-13) (GLUTATHIONE S-TRANSFERASE SUBUNIT 13) (GST CLASS-KAPPA) (HDCMD47P), full inser...	3342.1003	954.06024	2284.0	660.2486	1.5795715	3.0	0.21232854	0.33471236
gb:NM_053122.1 /DB_XREF=gi:16716420 /GEN=Immp2l-pending /FEA=FLmRNA /CNT=25 /TID=Mm.41069.1 /TIER=FL+Stack /STK=9 /UG=Mm.41069 /LL=93757 /DEF=Mus musculus inner mitochondrial membrane peptidase 2-like (S. cerevisiae) (Immp2l-pending), mRNA. /PROD=inner mitochondrial membrane peptidase 2-like(S. cerevisiae) /FL=gb:AF359564.1 gb:NM_053122.1	1377.0668	475.85724	873.3333	85.10619	1.8048774	2.0	0.21285515	0.33438143
gb:BM235840 /DB_XREF=gi:17871110 /DB_XREF=K0426C06-3 /CLONE=K0426C06 /FEA=mRNA /CNT=47 /TID=Mm.159813.1 /TIER=Stack /STK=11 /UG=Mm.159813 /UG_TITLE=Mus musculus, clone IMAGE:5102112, mRNA, partial cds	1349.6	955.2317	2434.4333	712.7231	1.5765651	3.0	0.21298838	0.33343697
gb:AK018713.1 /DB_XREF=gi:12858561 /GEN=Cyba /FEA=mRNA /CNT=1 /TID=Mm.448.2 /TIER=ConsEnd /STK=0 /UG=Mm.448 /LL=13057 /UG_TITLE=cytochrome b-245, alpha polypeptide /DEF=Mus musculus adult male kidney cDNA, RIKEN full-length enriched library, clone:0610007F11:cytochrome b-245, alpha polypeptide, full insert sequence.	1930.9332	586.1127	1270.2334	430.65775	1.5734007	3.0	0.21368538	0.33337855
gb:NM_011722.1 /DB_XREF=gi:6756008 /GEN=Dctn6 /FEA=FLmRNA /CNT=129 /TID=Mm.90496.1 /TIER=FL+Stack /STK=85 /UG=Mm.90496 /LL=22428 /DEF=Mus musculus dynactin 6 (Dctn6), mRNA. /PROD=dynactin 6 /FL=gb:AF124788.1 gb:NM_011722.1 gb:AF190796.1	1629.3002	83.222305	1194.2333	410.49402	1.7991323	2.0	0.21381108	0.3324323
gb:AK017822.1 /DB_XREF=gi:12857270 /GEN=Ssb /FEA=mRNA /CNT=2 /TID=Mm.10508.2 /TIER=ConsEnd /STK=1 /UG=Mm.10508 /LL=20823 /UG_TITLE=Sjogren syndrome antigen B /DEF=Mus musculus 8 days embryo whole body cDNA, RIKEN full-length enriched library, clone:5730546E07:Sjogren syndrome antigen B, full insert sequence.	938.3333	519.42316	1485.9	91.67742	1.798105	2.0	0.21398263	0.3315635
gb:NM_025509.1 /DB_XREF=gi:13384929 /GEN=2310008M10Rik /FEA=FLmRNA /CNT=192 /TID=Mm.9811.1 /TIER=FL+Stack /STK=21 /UG=Mm.9811 /LL=66357 /DEF=Mus musculus RIKEN cDNA 2310008M10 gene (2310008M10Rik), mRNA. /PROD=RIKEN cDNA 2310008M10 /FL=gb:BC021935.1 gb:NM_025509.1	1907.1666	421.60797	1362.2667	432.72708	1.5621666	3.0	0.21618043	0.33382964
gb:NM_007931.1 /DB_XREF=gi:6679646 /GEN=Endog /FEA=FLmRNA /CNT=21 /TID=Mm.4449.1 /TIER=FL /STK=2 /UG=Mm.4449 /LL=13804 /DEF=Mus musculus endonuclease G (Endog), mRNA. /PROD=endonuclease G /FL=gb:AB012108.1 gb:NM_007931.1	2059.8667	481.12747	1506.5333	383.07217	1.5583705	3.0	0.21703082	0.33400676
gb:BC026821.1 /DB_XREF=gi:20070721 /FEA=FLmRNA /CNT=85 /TID=Mm.87443.1 /TIER=FL+Stack /STK=32 /UG=Mm.87443 /LL=16649 /UG_GENE=Kpna4 /DEF=Mus musculus, karyopherin (importin) alpha 4, clone MGC:19244 IMAGE:3672708, mRNA, complete cds. /PROD=karyopherin (importin) alpha 4 /FL=gb:AF020771.1 gb:NM_008467.1 gb:BC026821.1	4096.9004	875.8401	6336.9	2006.1893	1.7723727	2.0	0.21833913	0.33488503
gb:NM_010189.1 /DB_XREF=gi:6753835 /GEN=Fcgrt /FEA=FLmRNA /CNT=178 /TID=Mm.3303.1 /TIER=FL+Stack /STK=61 /UG=Mm.3303 /LL=14132 /DEF=Mus musculus Fc receptor, IgG, alpha chain transporter (Fcgrt), mRNA. /PROD=Fc receptor, IgG, alpha chain transporter /FL=gb:L17022.1 gb:BC003786.1 gb:D37874.1 gb:NM_010189.1	1773.7666	361.7444	1248.7333	462.58038	1.5485989	3.0	0.21923696	0.33512992
gb:BC026638.1 /DB_XREF=gi:20072373 /FEA=FLmRNA /CNT=59 /TID=Mm.86759.1 /TIER=FL+Stack /STK=18 /UG=Mm.86759 /DEF=Mus musculus, Similar to mannosyl (alpha-1,3-)-glycoprotein beta-1,4-N-acetylglucosaminyltransferase, isoenzyme B, clone MGC:37502 IMAGE:4984907, mRNA, complete cds. /PROD=Similar to mannosyl (alpha-1,3-)-glycoproteinbeta-1,4-N-acetylglucosaminyltransferase, isoenzyme B /FL=gb:BC026638.1	1303.5	507.88135	790.4333	9.6656	1.7494179	2.0	0.22232446	0.33870906
gb:NM_025427.1 /DB_XREF=gi:13384823 /GEN=1190002H23Rik /FEA=FLmRNA /CNT=94 /TID=Mm.29811.1 /TIER=FL+Stack /STK=31 /UG=Mm.29811 /LL=66214 /DEF=Mus musculus RIKEN cDNA 1190002H23 gene (1190002H23Rik), mRNA. /PROD=RIKEN cDNA 1190002H23 /FL=gb:AF276981.1 gb:NM_025427.1	866.4666	374.6637	459.36667	265.9359	1.534698	3.0	0.2224183	0.33771876
gb:NM_012029.1 /DB_XREF=gi:6755521 /GEN=Sitpec-pending /FEA=FLmRNA /CNT=66 /TID=Mm.29790.1 /TIER=FL+Stack /STK=58 /UG=Mm.29790 /LL=26940 /DEF=Mus musculus signaling intermediate in Toll pathway-evolutionarily conserved (Sitpec-pending), mRNA. /PROD=signaling intermediate in Tollpathway-evolutionarily conserved /FL=gb:AF182510.1 gb:NM_012029.1	1686.1	384.39545	1222.3666	355.22467	1.5346091	3.0	0.22243881	0.3366241
gb:NM_133748.1 /DB_XREF=gi:19526953 /GEN=2900053I11Rik /FEA=FLmRNA /CNT=102 /TID=Mm.27136.1 /TIER=FL+Stack /STK=32 /UG=Mm.27136 /LL=72999 /DEF=Mus musculus RIKEN cDNA 2900053I11 gene (2900053I11Rik), mRNA. /PROD=RIKEN cDNA 2900053I11 /FL=gb:BC024411.1 gb:BC015288.1 gb:NM_133748.1 gb:BC023067.1	1265.4667	550.9264	1911.3334	479.13797	1.5321524	3.0	0.2230064	0.33636183
gb:AK018466.1 /DB_XREF=gi:12858179 /FEA=mRNA /CNT=65 /TID=Mm.99916.1 /TIER=Stack /STK=48 /UG=Mm.99916 /LL=71506 /UG_GENE=8430436O14Rik /UG_TITLE=RIKEN cDNA 8430436O14 gene /DEF=Mus musculus 16 days embryo lung cDNA, RIKEN full-length enriched library, clone:8430436O14:unclassifiable transcript, full insert sequence.	838.56665	393.75806	1282.5333	198.18613	1.7444108	2.0	0.22320645	0.33554876
gb:NM_011335.1 /DB_XREF=gi:7549786 /GEN=Scya21a /FEA=FLmRNA /CNT=15 /TID=Mm.220853.1 /TIER=FL+Stack /STK=10 /UG=Mm.220853 /LL=20298 /DEF=Mus musculus small inducible cytokine A21a (serine) (Scya21a), mRNA. /PROD=small inducible cytokine A21a (serine) /FL=gb:NM_011335.1 gb:U88322.1	2090.3665	517.5319	1365.1	638.4589	1.5284649	3.0	0.22386134	0.33542258
gb:BC014728.1 /DB_XREF=gi:15928499 /FEA=FLmRNA /CNT=90 /TID=Mm.212927.1 /TIER=FL /STK=1 /UG=Mm.212927 /DEF=Mus musculus, Similar to hypothetical protein MGC3169, clone MGC:25675 IMAGE:4504294, mRNA, complete cds. /PROD=Similar to hypothetical protein MGC3169 /FL=gb:BC024788.1 gb:BC014728.1	1344.0667	777.81036	559.3	75.45601	1.7393757	2.0	0.22409798	0.33467263
gb:NM_011694.1 /DB_XREF=gi:6755962 /GEN=Vdac1 /FEA=FLmRNA /CNT=381 /TID=Mm.3555.1 /TIER=FL+Stack /STK=149 /UG=Mm.3555 /LL=22333 /DEF=Mus musculus voltage-dependent anion channel 1 (Vdac1), mRNA. /PROD=voltage-dependent anion channel 1 /FL=gb:NM_011694.1 gb:U30840.1	11073.7	1535.2026	7599.4663	3120.6746	1.7302487	2.0	0.22572601	0.3359987
gb:NM_019822.1 /DB_XREF=gi:9789884 /GEN=Adrm1 /FEA=FLmRNA /CNT=68 /TID=Mm.181562.1 /TIER=FL+Stack /STK=11 /UG=Mm.181562 /LL=56436 /DEF=Mus musculus adhesion regulating molecule 1 (Adrm1), mRNA. /PROD=adhesion regulating molecule 1 /FL=gb:AF225959.1 gb:NM_019822.1 gb:BC008974.1	1396.5	490.32642	901.6667	86.00303	1.721688	2.0	0.2272671	0.33718714
gb:BB206220 /DB_XREF=gi:8871173 /DB_XREF=BB206220 /CLONE=A430073H16 /FEA=EST /CNT=26 /TID=Mm.30018.2 /TIER=Stack /STK=26 /UG=Mm.30018 /UG_TITLE=Mus musculus, serine threonine kinase pim3, clone MGC:27707 IMAGE:4924687, mRNA, complete cds	4001.0332	1582.3501	2393.8	378.13297	1.7111088	2.0	0.22919065	0.33893338
gb:BC024518.1 /DB_XREF=gi:19354490 /FEA=FLmRNA /CNT=81 /TID=Mm.22138.1 /TIER=FL+Stack /STK=35 /UG=Mm.22138 /DEF=Mus musculus, Similar to ubiquinol-cytochrome c reductase complex (7.2 kD), clone MGC:37480 IMAGE:4984172, mRNA, complete cds. /PROD=Similar to ubiquinol-cytochrome c reductasecomplex (7.2 kD) /FL=gb:BC024518.1	17520.266	5364.1704	12089.233	1239.3374	1.70863	2.0	0.22964443	0.33850184
gb:NM_007990.1 /DB_XREF=gi:6679752 /GEN=Fau /FEA=FLmRNA /CNT=140 /TID=Mm.4890.1 /TIER=FL+Stack /STK=44 /UG=Mm.4890 /LL=14109 /DEF=Mus musculus Finkel-Biskis-Reilly murine sarcoma virus (FBR-MuSV) ubiquitously expressed (fox derived) (Fau), mRNA. /PROD=Finkel-Biskis-Reilly murine sarcoma virus(FBR-MuSV) ubiquitously expressed (fox derived) /FL=gb:NM_007990.1 gb:AF147745.1 gb:D26610.1	13061.8	3870.341	8593.399	3409.3862	1.5005265	3.0	0.23045751	0.33860102
gb:BG244243 /DB_XREF=gi:12754058 /DB_XREF=602356434F1 /CLONE=IMAGE:4485056 /FEA=EST /CNT=39 /TID=Mm.44227.2 /TIER=Stack /STK=39 /UG=Mm.44227 /UG_TITLE=Mus musculus, clone MGC:37950 IMAGE:5132866, mRNA, complete cds	23382.932	10109.377	13031.466	6374.3315	1.500204	3.0	0.23053491	0.3376221
Mus musculus /REF=M12481 /DEF=Mouse cytoplasmic beta-actin mRNA /LEN=1109 (_5, _M, _3 represent transcript regions 5 prime, Middle, and 3 prime respectively)	11262.766	4487.95	6811.467	2567.0461	1.4912015	3.0	0.23270646	0.3397065
gb:NM_013899.1 /DB_XREF=gi:7305574 /GEN=Timm13a /FEA=FLmRNA /CNT=90 /TID=Mm.21826.1 /TIER=FL+Stack /STK=43 /UG=Mm.21826 /LL=30059 /DEF=Mus musculus translocase of inner mitochondrial membrane 13 homolog a (yeast) (Timm13a), mRNA. /PROD=translocase of inner mitochondrial membrane 13homolog a (yeast) /FL=gb:AF150090.1 gb:NM_013899.1	5904.9336	1361.7144	4263.733	1337.6084	1.4892417	3.0	0.23318216	0.33930993
gb:BC002008.1 /DB_XREF=gi:12805102 /FEA=FLmRNA /CNT=262 /TID=Mm.741.1 /TIER=FL+Stack /STK=143 /UG=Mm.741 /LL=16592 /UG_GENE=Fabp5 /DEF=Mus musculus, fatty acid binding protein 5, epidermal, clone MGC:5786 IMAGE:3490535, mRNA, complete cds. /PROD=fatty acid binding protein 5, epidermal /FL=gb:NM_010634.1 gb:BC002008.1	4093.6667	1114.5125	3003.1335	609.46136	1.4869765	3.0	0.23373333	0.33902535
gb:AK008824.1 /DB_XREF=gi:12843252 /FEA=mRNA /CNT=3 /TID=Mm.158805.1 /TIER=ConsEnd /STK=1 /UG=Mm.158805 /LL=72279 /UG_GENE=2210404C19Rik /UG_TITLE=RIKEN cDNA 2210404C19 gene /DEF=Mus musculus adult male stomach cDNA, RIKEN full-length enriched library, clone:2210404C19:related to GB|AAF24540.1, full insert sequence.	1792.4332	644.0837	1104.4	297.82657	1.6793883	2.0	0.23508698	0.33990282
gb:NM_025577.1 /DB_XREF=gi:13385019 /GEN=2810428I15Rik /FEA=FLmRNA /CNT=48 /TID=Mm.28242.1 /TIER=FL+Stack /STK=19 /UG=Mm.28242 /LL=66462 /DEF=Mus musculus RIKEN cDNA 2810428I15 gene (2810428I15Rik), mRNA. /PROD=RIKEN cDNA 2810428I15 /FL=gb:NM_025577.1	1729.6333	373.95267	1207.7999	482.65717	1.4803193	3.0	0.23536141	0.3392193
gb:NM_009439.1 /DB_XREF=gi:19705423 /GEN=Psmd3 /FEA=FLmRNA /CNT=195 /TID=Mm.12194.1 /TIER=FL+Stack /STK=67 /UG=Mm.12194 /LL=22123 /DEF=Mus musculus proteasome (prosome, macropain) 26S subunit, non-ATPase, 3 (Psmd3), mRNA. /PROD=proteasome (prosome, macropain) 26S subunit,non-ATPase, 3 /FL=gb:NM_009439.1 gb:BC003197.1	1436.9	464.20163	966.0	151.58029	1.6702513	2.0	0.23682195	0.34024417
gb:BC010345.1 /DB_XREF=gi:16307590 /FEA=FLmRNA /CNT=121 /TID=Mm.12267.2 /TIER=FL+Stack /STK=40 /UG=Mm.12267 /LL=170715 /UG_GENE=MGC6685 /DEF=Mus musculus, clone MGC:11665 IMAGE:3707766, mRNA, complete cds. /PROD=Unknown (protein for MGC:11665) /FL=gb:BC010345.1	4166.533	1018.5175	3069.8	796.75226	1.4689889	3.0	0.23816094	0.34108853
gb:NM_011031.1 /DB_XREF=gi:6754969 /GEN=P4ha2 /FEA=FLmRNA /CNT=86 /TID=Mm.3705.1 /TIER=FL+Stack /STK=55 /UG=Mm.3705 /LL=18452 /DEF=Mus musculus procollagen-proline, 2-oxoglutarate 4-dioxygenase (proline 4-hydroxylase), alpha II polypeptide (P4ha2), mRNA. /PROD=procollagen-proline, 2-oxoglutarate4-dioxygenase (proline 4-hydroxylase), alpha IIpolypeptide /FL=gb:BC018411.1 gb:NM_011031.1 gb:U16163.1	1713.3002	499.787	1194.1666	360.7275	1.4588095	3.0	0.24070708	0.343651
gb:AK020708.1 /DB_XREF=gi:12861376 /FEA=mRNA /CNT=32 /TID=Mm.142511.2 /TIER=Stack /STK=10 /UG=Mm.142511 /LL=106529 /UG_GENE=AI173355 /UG_TITLE=expressed sequence AI173355 /DEF=Mus musculus adult male hypothalamus cDNA, RIKEN full-length enriched library, clone:A230102P12:homolog to SC2=SYNAPTIC GLYCOPROTEIN, full insert sequence.	6540.6997	1708.0126	4378.0005	1922.3267	1.4566965	3.0	0.24123931	0.3433312
gb:BC004732.1 /DB_XREF=gi:13435746 /FEA=FLmRNA /CNT=285 /TID=Mm.30016.2 /TIER=FL+Stack /STK=79 /UG=Mm.30016 /LL=97755 /UG_GENE=C87222 /DEF=Mus musculus, clone MGC:7904 IMAGE:3583026, mRNA, complete cds. /PROD=Unknown (protein for MGC:7904) /FL=gb:BC004732.1	2339.9668	1092.4368	1295.1666	129.02925	1.6450883	2.0	0.24168666	0.34289294
gb:NM_011777.1 /DB_XREF=gi:6756084 /GEN=Zyx /FEA=FLmRNA /CNT=160 /TID=Mm.4895.1 /TIER=FL+Stack /STK=51 /UG=Mm.4895 /LL=22793 /DEF=Mus musculus zyxin (Zyx), mRNA. /PROD=zyxin /FL=gb:NM_011777.1	1814.6	746.12103	1055.8	285.90887	1.6448554	2.0	0.2417323	0.3418893
gb:BC011306.1 /DB_XREF=gi:15030108 /FEA=FLmRNA /CNT=38 /TID=Mm.16771.2 /TIER=FL /STK=2 /UG=Mm.16771 /LL=14972 /UG_GENE=H2-K /DEF=Mus musculus, clone MGC:7052 IMAGE:3156482, mRNA, complete cds. /PROD=Unknown (protein for MGC:7052) /FL=gb:BC011306.1	7775.4336	1879.953	5659.6333	1689.736	1.449787	3.0	0.24298863	0.3425989
gb:NM_009720.1 /DB_XREF=gi:6753135 /GEN=Atox1 /FEA=FLmRNA /CNT=114 /TID=Mm.9703.1 /TIER=FL+Stack /STK=96 /UG=Mm.9703 /LL=11927 /DEF=Mus musculus ATX1 (antioxidant protein 1) homolog 1 (yeast) (Atox1), mRNA. /PROD=ATX1 (antioxidant protein 1, yeast) homolog 1 /FL=gb:AF004591.1 gb:NM_009720.1	1247.1666	382.0489	829.26666	321.92905	1.4488084	3.0	0.24323751	0.341888
gb:AF157628.1 /DB_XREF=gi:8886020 /FEA=mRNA /CNT=12 /TID=Mm.37213.1 /TIER=ConsEnd /STK=0 /UG=Mm.37213 /LL=19263 /UG_GENE=Ptprb /DEF=Mus musculus receptor-type protein tyrosine phosphatase mRNA, partial cds. /PROD=receptor-type protein tyrosine phosphatase	3937.7668	976.8946	2722.5	1080.711	1.4448842	3.0	0.24423829	0.34223515
gb:NM_010580.1 /DB_XREF=gi:6754379 /GEN=Itgb5 /FEA=FLmRNA /CNT=117 /TID=Mm.6424.1 /TIER=FL+Stack /STK=42 /UG=Mm.6424 /LL=16419 /DEF=Mus musculus integrin beta 5 (Itgb5), mRNA. /PROD=integrin beta 5 /FL=gb:NM_010580.1 gb:AF022110.1 gb:AF043257.1 gb:AF043256.1	3718.7666	1939.8148	1818.9335	550.6831	1.6318688	2.0	0.24429424	0.34126025

gb:NM_024243.1 /DB_XREF=gi:18859606 /GEN=0610006A03Rik /FEA=FLmRNA /CNT=204 /TID=Mm.21580.1 /TIER=FL+Stack /STK=126 /UG=Mm.21580 /LL=71665 /DEF=Mus musculus RIKEN cDNA 0610006A03 gene (0610006A03Rik), mRNA. /PROD=RIKEN cDNA 0610006A03 /FL=gb:NM_024243.1 gb:BC003235.1	2050.0999	373.57025	1488.3667	560.70416	1.4440745	3.0	0.24444532	0.34042385
Mus musculus /REF=M12481 /DEF=Mouse cytoplasmic beta-actin mRNA /LEN=1109 (_5, _M, _3 represent transcript regions 5 prime, Middle, and 3 prime respectively)	7098.533	3205.232	3795.8333	1436.3824	1.6286589	2.0	0.24493289	0.34005973
gb:AK006418.1 /DB_XREF=gi:12839509 /FEA=mRNA /CNT=9 /TID=Mm.139700.2 /TIER=ConsEnd /STK=7 /UG=Mm.139700 /LL=76171 /UG_GENE=6330544B05Rik /UG_TITLE=RIKEN cDNA 6330544B05 gene /DEF=Mus musculus adult male testis cDNA, RIKEN full-length enriched library, clone:1700027H16:homolog to ARF-LIKE 2 BINDING PROTEIN BART1 (BINDER OF ARL TWO), full insert sequence.	1334.9333	456.44818	879.5999	167.46419	1.6220952	2.0	0.24624552	0.34083983
gb:C76322 /DB_XREF=gi:2516652 /DB_XREF=C76322 /CLONE=J0008F11 /FEA=FLmRNA /CNT=215 /TID=Mm.22632.1 /TIER=Stack /STK=32 /UG=Mm.22632 /UG_TITLE=Mus musculus, Similar to transgelin 2, clone MGC:6300 IMAGE:2654381, mRNA, complete cds /FL=gb:BC009076.1 gb:AF149291.1	1422.2999	547.77124	888.6667	163.41887	1.6169243	2.0	0.247286	0.34123966
gb:AK012780.1 /DB_XREF=gi:12849748 /FEA=mRNA /CNT=10 /TID=Mm.1980.2 /TIER=ConsEnd /STK=6 /UG=Mm.1980 /LL=69253 /UG_GENE=Hspb2 /UG_TITLE=heat shock 27kD protein 2 /DEF=Mus musculus 10, 11 days embryo whole body cDNA, RIKEN full-length enriched library, clone:2810021G24:homolog to HEAT-SHOCK PROTEIN, BETA-2 (DMPK-BINDING PROTEIN) (MKBP), full insert sequence.	1949.6333	717.2411	1206.8	345.3317	1.6162702	2.0	0.24741803	0.34038723
gb:NM_008183.1 /DB_XREF=gi:6680120 /GEN=Gstm2 /FEA=FLmRNA /CNT=135 /TID=Mm.14601.1 /TIER=FL+Stack /STK=91 /UG=Mm.14601 /LL=14863 /DEF=Mus musculus glutathione S-transferase, mu 2 (Gstm2), mRNA. /PROD=glutathione-S-transferase, mu 2 /FL=gb:J04696.1 gb:NM_008183.1	2698.0	388.65005	1897.5333	767.24585	1.6120245	2.0	0.24827717	0.34053728
gb:NM_010798.1 /DB_XREF=gi:6754695 /GEN=Mif /FEA=FLmRNA /CNT=216 /TID=Mm.2326.1 /TIER=FL+Stack /STK=96 /UG=Mm.2326 /LL=17319 /DEF=Mus musculus macrophage migration inhibitory factor (Mif), mRNA. /PROD=macrophage migration inhibitory factor /FL=gb:NM_010798.1 gb:L10613.1 gb:BC024895.1	5992.4336	1752.3951	4222.6333	1240.7228	1.4276481	3.0	0.24868712	0.34007215
gb:NM_019391.1 /DB_XREF=gi:11225263 /GEN=Lsp1 /FEA=FLmRNA /CNT=62 /TID=Mm.2183.1 /TIER=FL+Stack /STK=37 /UG=Mm.2183 /LL=16985 /DEF=Mus musculus lymphocyte specific 1 (Lsp1), mRNA. /PROD=lymphocyte specific 1 /FL=gb:NM_019391.1 gb:M90316.1 gb:BC003796.1	1213.8667	345.4198	850.1667	274.75565	1.4272609	3.0	0.24878806	0.33918855
gb:NM_007744.1 /DB_XREF=gi:6680981 /GEN=Comt /FEA=FLmRNA /CNT=84 /TID=Mm.35853.1 /TIER=FL+Stack /STK=19 /UG=Mm.35853 /LL=12846 /DEF=Mus musculus catechol-O-methyltransferase (Comt), mRNA. /PROD=catechol-O-methyltransferase /FL=gb:AF076156.1 gb:NM_007744.1 gb:BC010402.1	6855.0996	1962.6735	4987.967	434.9332	1.60871	2.0	0.24895056	0.33839387
gb:NM_138749.1 /DB_XREF=gi:20270280 /GEN=Plxnb2 /FEA=FLmRNA /CNT=197 /TID=Mm.28780.1 /TIER=FL+Stack /STK=74 /UG=Mm.28780 /LL=140570 /DEF=Mus musculus plexin B2 (Plxnb2), mRNA. /PROD=plexin B2 /FL=gb:NM_138749.1 gb:BC007481.1 gb:BC003293.1	1338.4667	664.8858	697.9333	183.63422	1.6083946	2.0	0.24901477	0.33747074
gb:NM_030694.1 /DB_XREF=gi:13507625 /GEN=Ifitm3l /FEA=FLmRNA /CNT=102 /TID=Mm.14747.1 /TIER=FL+Stack /STK=44 /UG=Mm.14747 /LL=80876 /DEF=Mus musculus interferon induced transmembrane protein 3-like (Ifitm3l), mRNA. /PROD=interferon induced transmembrane protein, like /FL=gb:BC002160.1 gb:BC002102.1 gb:AY082486.1 gb:NM_030694.1	7020.6	2221.2522	4749.467	1641.2349	1.4243245	3.0	0.249555	0.33719635
gb:BC025117.1 /DB_XREF=gi:19264026 /FEA=FLmRNA /CNT=85 /TID=Mm.21793.1 /TIER=FL+Stack /STK=13 /UG=Mm.21793 /LL=66363 /UG_GENE=2310005G07Rik /DEF=Mus musculus, RIKEN cDNA 2310005G07 gene, clone MGC:35747 IMAGE:3984181, mRNA, complete cds. /PROD=RIKEN cDNA 2310005G07 gene /FL=gb:NM_025514.1 gb:BC025117.1	2440.0667	653.6071	1782.5668	282.57187	1.5993049	2.0	0.25087416	0.3379729
gb:BC027196.1 /DB_XREF=gi:20071133 /FEA=FLmRNA /CNT=203 /TID=Mm.34411.1 /TIER=FL+Stack /STK=128 /UG=Mm.34411 /DEF=Mus musculus, Similar to pyruvate dehydrogenase kinase, isoenzyme 1, clone MGC:28719 IMAGE:4458562, mRNA, complete cds. /PROD=Similar to pyruvate dehydrogenase kinase,isoenzyme 1 /FL=gb:BC027196.1	1481.2001	424.4901	2639.0334	1182.8103	1.5958194	2.0	0.25159192	0.3379371
gb:BC013496.1 /DB_XREF=gi:15488700 /FEA=mRNA /CNT=2 /TID=Mm.104747.19 /TIER=ConsEnd /STK=0 /UG=Mm.104747 /LL=16114 /UG_GENE=Igk-V28 /DEF=Mus musculus, clone IMAGE:4219618, mRNA, partial cds. /PROD=Unknown (protein for IMAGE:4219618)	2536.7	1346.0999	5748.767	3229.9329	1.5899211	2.0	0.2528125	0.33857486
gb:NM_013670.1 /DB_XREF=gi:7305508 /GEN=Snrpn /FEA=FLmRNA /CNT=253 /TID=Mm.43721.1 /TIER=FL+Stack /STK=178 /UG=Mm.43721 /LL=20646 /DEF=Mus musculus small nuclear ribonucleoprotein N (Snrpn), mRNA. /PROD=small nuclear ribonucleoprotein N /FL=gb:BC024880.1 gb:NM_013670.1 gb:BC019589.1	4073.7668	1357.4027	2778.2334	400.39764	1.5855645	2.0	0.25371894	0.3387894
gb:BC013777.1 /DB_XREF=gi:15489363 /FEA=FLmRNA /CNT=96 /TID=Mm.821.1 /TIER=FL+Stack /STK=47 /UG=Mm.821 /LL=57806 /UG_GENE=Snrpa1 /DEF=Mus musculus, clone MGC:6298 IMAGE:2651704, mRNA, complete cds. /PROD=Unknown (protein for MGC:6298) /FL=gb:AF230356.1 gb:BC013777.1 gb:NM_021336.1	1188.9333	536.97046	1902.2999	693.9675	1.4081479	3.0	0.2538262	0.3379387
gb:NM_008816.1 /DB_XREF=gi:6679272 /GEN=Pecam /FEA=FLmRNA /CNT=15 /TID=Mm.2822.1 /TIER=FL /STK=2 /UG=Mm.2822 /LL=18613 /DEF=Mus musculus plateletendothelial cell adhesion molecule (Pecam), mRNA. /PROD=plateletendothelial cell adhesion molecule /FL=gb:NM_008816.1 gb:L06039.1	1024.2666	261.8756	663.30005	358.61865	1.407957	3.0	0.2538771	0.33701816
gb:BC004096.1 /DB_XREF=gi:13278617 /FEA=FLmRNA /CNT=1 /TID=Mm.16710.2 /TIER=FL /STK=1 /UG=Mm.16710 /LL=16211 /UG_GENE=Impnb /DEF=Mus musculus, clone MGC:8315 IMAGE:3594201, mRNA, complete cds. /PROD=Unknown (protein for MGC:8315) /FL=gb:BC004096.1	2197.7332	580.041	3536.7668	1344.1012	1.5842928	2.0	0.2539843	0.33617747
gb:AF205078.1 /DB_XREF=gi:9828172 /FEA=mRNA /CNT=116 /TID=Mm.29933.1 /TIER=Stack /STK=76 /UG=Mm.29933 /LL=72333 /UG_GENE=2410003B16Rik /DEF=Mus musculus strain Swiss WebsterNIH actin-associated protein palladin mRNA, partial cds. /PROD=actin-associated protein palladin	2120.3	569.42145	1508.4332	496.02515	1.4033719	3.0	0.25510228	0.33667567
gb:NM_026612.1 /DB_XREF=gi:13386095 /GEN=1810011O01Rik /FEA=FLmRNA /CNT=92 /TID=Mm.29415.1 /TIER=FL+Stack /STK=67 /UG=Mm.29415 /LL=68198 /DEF=Mus musculus RIKEN cDNA 1810011O01 gene (1810011O01Rik), mRNA. /PROD=RIKEN cDNA 1810011O01 /FL=gb:BC013510.1 gb:NM_026612.1	15384.167	4013.6558	11335.101	1932.8859	1.5742899	2.0	0.25608414	0.33699188
gb:BC011114.1 /DB_XREF=gi:15029785 /FEA=FLmRNA /CNT=105 /TID=Mm.41926.1 /TIER=FL+Stack /STK=55 /UG=Mm.41926 /LL=17992 /UG_GENE=Ndufa4 /DEF=Mus musculus, NADH dehydrogenase (ubiquinone) 1 alpha subcomplex, 4, clone MGC:7218 IMAGE:3483028, mRNA, complete cds. /PROD=NADH dehydrogenase (ubiquinone) 1 alphasubcomplex, 4 /FL=gb:BC011114.1	20005.533	6725.475	13639.532	1986.9003	1.5722948	2.0	0.2565056	0.33657095
gb:NM_011793.1 /DB_XREF=gi:6753177 /GEN=Bcrp1-pending /FEA=FLmRNA /CNT=16 /TID=Mm.7508.1 /TIER=FL /STK=2 /UG=Mm.7508 /LL=23825 /DEF=Mus musculus breakpoint cluster region protein 1 (Bcrp1-pending), mRNA. /PROD=breakpoint cluster region protein 1 /FL=gb:NM_011793.1 gb:AB025349.1	1859.2999	494.08163	1304.1666	480.25177	1.3954734	3.0	0.25722775	0.33654583
gb:NM_025706.1 /DB_XREF=gi:13385163 /GEN=4432405K22Rik /FEA=FLmRNA /CNT=232 /TID=Mm.22252.1 /TIER=FL+Stack /STK=137 /UG=Mm.22252 /LL=66687 /DEF=Mus musculus RIKEN cDNA 4432405K22 gene (4432405K22Rik), mRNA. /PROD=RIKEN cDNA 4432405K22 /FL=gb:NM_025706.1	2074.0334	925.5119	3127.4668	926.4323	1.3933324	3.0	0.25780717	0.33633462
gb:AK004661.1 /DB_XREF=gi:12835991 /FEA=FLmRNA /CNT=211 /TID=Mm.22179.1 /TIER=Stack /STK=28 /UG=Mm.22179 /LL=67456 /UG_GENE=1200009B18Rik /UG_TITLE=RIKEN cDNA 1200009B18 gene /DEF=Mus musculus adult male lung cDNA, RIKEN full-length enriched library, clone:1200009B18:homolog to CDA14, full insert sequence. /FL=gb:BC006895.1 gb:BC018188.1 gb:BC026558.1	963.0	467.03268	1549.3334	564.1123	1.3867055	3.0	0.25960946	0.33771545
gb:BC023060.1 /DB_XREF=gi:18606201 /FEA=mRNA /CNT=19 /TID=Mm.44176.1 /TIER=Stack /STK=11 /UG=Mm.44176 /DEF=Mus musculus, Similar to EGF-containing fibulin-like extracellular matrix protein 1, clone IMAGE:5357328, mRNA, partial cds. /PROD=Similar to EGF-containing fibulin-likeextracellular matrix protein 1	1247.9333	767.05914	1964.3334	217.40591	1.5563558	2.0	0.25990483	0.3371337
gb:NM_026533.1 /DB_XREF=gi:13386033 /GEN=Rps13 /FEA=FLmRNA /CNT=343 /TID=Mm.14798.1 /TIER=FL+Stack /STK=170 /UG=Mm.14798 /LL=68052 /DEF=Mus musculus ribosomal protein S13 (Rps13), mRNA. /PROD=ribosomal protein S13 /FL=gb:NM_026533.1 gb:BC011192.1	17834.967	5539.1167	12845.399	941.7359	1.538138	2.0	0.26386073	0.34128997
gb:NM_013670.1 /DB_XREF=gi:7305508 /GEN=Snrpn /FEA=FLmRNA /CNT=253 /TID=Mm.43721.1 /TIER=FL+Stack /STK=178 /UG=Mm.43721 /LL=20646 /DEF=Mus musculus small nuclear ribonucleoprotein N (Snrpn), mRNA. /PROD=small nuclear ribonucleoprotein N /FL=gb:BC024880.1 gb:NM_013670.1 gb:BC019589.1	3676.5	1964.3711	1895.9	476.49503	1.5257676	2.0	0.26659068	0.34384137
gb:AK005096.1 /DB_XREF=gi:12836790 /FEA=mRNA /CNT=38 /TID=Mm.44075.1 /TIER=Stack /STK=22 /UG=Mm.44075 /LL=70364 /UG_GENE=1500001L03Rik /UG_TITLE=RIKEN cDNA 1500001L03 gene /DEF=Mus musculus adult male cerebellum cDNA, RIKEN full-length enriched library, clone:1500001L03:homolog to CDNA: FLJ22362 FIS, CLONE HRC06544, full insert sequence.	1331.4	363.54633	911.39996	398.89136	1.34789	3.0	0.2704402	0.34781826
gb:NM_011793.1 /DB_XREF=gi:6753177 /GEN=Bcrp1-pending /FEA=FLmRNA /CNT=16 /TID=Mm.7508.1 /TIER=FL /STK=2 /UG=Mm.7508 /LL=23825 /DEF=Mus musculus breakpoint cluster region protein 1 (Bcrp1-pending), mRNA. /PROD=breakpoint cluster region protein 1 /FL=gb:NM_011793.1 gb:AB025349.1	1313.1333	341.6223	867.7	460.86154	1.3448688	3.0	0.27130315	0.34794244
gb:NM_022318.1 /DB_XREF=gi:11612496 /GEN=Pop2-pending /FEA=FLmRNA /CNT=49 /TID=Mm.23890.1 /TIER=FL+Stack /STK=36 /UG=Mm.23890 /LL=64082 /DEF=Mus musculus popeye 2 (Pop2-pending), mRNA. /PROD=popeye 2 /FL=gb:AF204175.1 gb:NM_022318.1	7395.8667	2724.9272	4811.0	1195.4429	1.504601	2.0	0.2713454	0.3470164
gb:BC026550.1 /DB_XREF=gi:20071652 /FEA=FLmRNA /CNT=121 /TID=Mm.10091.2 /TIER=FL+Stack /STK=74 /UG=Mm.10091 /LL=18769 /UG_GENE=Pkig /DEF=Mus musculus, protein kinase inhibitor, gamma, clone MGC:36052 IMAGE:5006826, mRNA, complete cds. /PROD=protein kinase inhibitor, gamma /FL=gb:BC026550.1	4608.9336	575.2312	3331.2	1355.7172	1.5027447	2.0	0.2717675	0.3465799
gb:NM_031176.1 /DB_XREF=gi:13928671 /GEN=Tnxb /FEA=FLmRNA /CNT=13 /TID=Mm.183266.1 /TIER=FL /STK=2 /UG=Mm.183266 /LL=81877 /DEF=Mus musculus tenascin XB (Tnxb), mRNA. /PROD=tenascin XB /FL=gb:NM_031176.1	1731.1666	907.9988	932.7	486.432	1.342591	3.0	0.27195567	0.34584838
gb:NM_007750.1 /DB_XREF=gi:6680992 /GEN=Cox8a /FEA=FLmRNA /CNT=169 /TID=Mm.14022.1 /TIER=FL+Stack /STK=125 /UG=Mm.14022 /LL=12868 /DEF=Mus musculus cytochrome c oxidase, subunit VIIIa (Cox8a), mRNA. /PROD=cytochrome c oxidase, subunit VIIIa /FL=gb:U37721.1 gb:NM_007750.1	2268.0	424.9446	1662.5	663.0841	1.3316449	3.0	0.2751148	0.3488886
gb:NM_009982.1 /DB_XREF=gi:6753553 /GEN=Ctsc /FEA=FLmRNA /CNT=280 /TID=Mm.684.1 /TIER=FL+Stack /STK=93 /UG=Mm.684 /LL=13032 /DEF=Mus musculus cathepsin C (Ctsc), mRNA. /PROD=cathepsin C /FL=gb:U74683.3 gb:NM_009982.1 gb:U89269.1	4534.1333	1361.0074	3302.5999	532.1105	1.4596839	2.0	0.28179538	0.3563652
gb:BC002090.1 /DB_XREF=gi:12805254 /FEA=FLmRNA /CNT=31 /TID=Mm.27128.1 /TIER=FL /STK=2 /UG=Mm.27128 /LL=66556 /UG_GENE=2310074H19Rik /DEF=Mus musculus, Similar to DR1-associated protein 1 (negative cofactor 2 alpha), clone MGC:6341 IMAGE:3488139, mRNA, complete cds. /PROD=Similar to DR1-associated protein 1 (negativecofactor 2 alpha) /FL=gb:BC002090.1	3833.4668	1725.8673	2366.5999	262.58167	1.4553742	2.0	0.28282443	0.35667303
gb:BC027290.1 /DB_XREF=gi:20070999 /FEA=FLmRNA /CNT=23 /TID=Mm.48232.1 /TIER=FL+Stack /STK=14 /UG=Mm.48232 /LL=78688 /UG_GENE=B430311C09Rik /DEF=Mus musculus, RIKEN cDNA B430311C09 gene, clone MGC:27734 IMAGE:2647023, mRNA, complete cds. /PROD=RIKEN cDNA B430311C09 gene /FL=gb:BC027290.1	1301.5	433.59323	899.60004	206.88048	1.448966	2.0	0.28436327	0.35762027
gb:BC024398.1 /DB_XREF=gi:19353453 /FEA=FLmRNA /CNT=102 /TID=Mm.10586.1 /TIER=FL+Stack /STK=33 /UG=Mm.10586 /LL=71966 /UG_GENE=2410003M04Rik /DEF=Mus musculus, RIKEN cDNA 2410003M04 gene, clone MGC:36234 IMAGE:5003001, mRNA, complete cds. /PROD=RIKEN cDNA 2410003M04 gene /FL=gb:BC013469.1 gb:BC024398.1	1527.3666	475.4801	1109.0333	157.3033	1.4467617	2.0	0.28489503	0.3572993
gb:BC020068.1 /DB_XREF=gi:18043363 /FEA=FLmRNA /CNT=31 /TID=Mm.172352.1 /TIER=FL /STK=6 /UG=Mm.172352 /DEF=Mus musculus, Similar to RIKEN cDNA 9530053H05 gene, clone MGC:28371 IMAGE:4020866, mRNA, complete cds. /PROD=Similar to RIKEN cDNA 9530053H05 gene /FL=gb:BC020068.1	610.4333	222.68494	1039.9	463.5047	1.4465678	2.0	0.28494188	0.35637358
gb:NM_029796.1 /DB_XREF=gi:16418334 /GEN=Lrg-pending /FEA=FLmRNA /CNT=132 /TID=Mm.176946.1 /TIER=FL+Stack /STK=49 /UG=Mm.176946 /LL=76905 /DEF=Mus musculus leucine-rich alpha-2-glycoprotein (Lrg-pending), mRNA. /PROD=leucine-rich alpha-2-glycoprotein /FL=gb:AF403429.1 gb:AB055885.1 gb:NM_029796.1	990.1667	377.34503	612.86664	335.041	1.2950386	3.0	0.2859639	0.35666925
gb:BC010331.1 /DB_XREF=gi:16307568 /FEA=FLmRNA /CNT=140 /TID=Mm.6586.1 /TIER=FL+Stack /STK=66 /UG=Mm.6586 /DEF=Mus musculus, clone MGC:6299 IMAGE:2654341, mRNA, complete cds. /PROD=Unknown (protein for MGC:6299) /FL=gb:BC010331.1 gb:AF071546.1	1688.6666	570.3714	1201.7999	318.48758	1.2908622	3.0	0.28722993	0.3572668
gb:NM_007750.1 /DB_XREF=gi:6680992 /GEN=Cox8a /FEA=FLmRNA /CNT=169 /TID=Mm.14022.1 /TIER=FL+Stack /STK=125 /UG=Mm.14022 /LL=12868 /DEF=Mus musculus cytochrome c oxidase, subunit VIIIa (Cox8a), mRNA. /PROD=cytochrome c oxidase, subunit VIIIa /FL=gb:U37721.1 gb:NM_007750.1	2348.0667	582.6127	1683.4332	680.7008	1.2848178	3.0	0.28907257	0.35857636
gb:AK007959.1 /DB_XREF=gi:12841843 /GEN=Klf3 /FEA=mRNA /CNT=79 /TID=Mm.28787.2 /TIER=Stack /STK=31 /UG=Mm.28787 /LL=16599 /UG_TITLE=Kruppel-like factor 3 (basic) /DEF=Mus musculus 10 day old male pancreas cDNA, RIKEN full-length enriched library, clone:1810073A04:Kruppel-like factor 3 (basic), full insert sequence.	1284.3334	330.9694	894.1333	409.0531	1.2844399	3.0	0.2891882	0.35774237
gb:AK018782.1 /DB_XREF=gi:12858675 /FEA=mRNA /CNT=90 /TID=Mm.45683.1 /TIER=Stack /STK=34 /UG=Mm.45683 /LL=78304 /UG_GENE=1500034E06Rik /UG_TITLE=RIKEN cDNA 1500034E06 gene /DEF=Mus musculus adult male cerebellum cDNA, RIKEN full-length enriched library, clone:1500034E06:Sm protein containing protein, full insert sequence.	6024.7993	1744.9688	4426.1	861.1195	1.4230223	2.0	0.2907013	0.35863695
gb:AK017269.1 /DB_XREF=gi:12856421 /FEA=mRNA /CNT=42 /TID=Mm.23242.1 /TIER=Stack /STK=21 /UG=Mm.23242 /LL=71408 /UG_GENE=5430405D20Rik /UG_TITLE=RIKEN cDNA 5430405D20 gene /DEF=Mus musculus 6 days neonate head cDNA, RIKEN full-length enriched library, clone:5430405D20:unclassifiable transcript, full insert sequence.	851.36664	218.27292	1371.7666	598.14606	1.4156123	2.0	0.29254377	0.35993192
gb:BC005547.1 /DB_XREF=gi:13542679 /FEA=FLmRNA /CNT=515 /TID=Mm.21840.1 /TIER=FL+Stack /STK=279 /UG=Mm.21840 /LL=68222 /UG_GENE=4930542G03Rik /DEF=Mus musculus, clone MGC:6713 IMAGE:3585500, mRNA, complete cds. /PROD=Unknown (protein for MGC:6713) /FL=gb:BC022919.1 gb:BC005547.1	7940.133	2849.038	5353.1	1400.036	1.4115437	2.0	0.29356164	0.36020806
gb:BC027632.1 /DB_XREF=gi:20380326 /FEA=mRNA /CNT=25 /TID=Mm.9703.2 /TIER=ConsEnd /STK=0 /UG=Mm.9703 /LL=11927 /UG_GENE=Atox1 /DEF=Mus musculus, clone MGC:41183 IMAGE:1328907, mRNA, complete cds. /PROD=Unknown (protein for MGC:41183)	2955.2668	838.1132	2112.1667	790.91815	1.2671942	3.0	0.294516	0.36040503

gb:NM_008303.1 /DB_XREF=gi:6680308 /GEN=Hspe1 /FEA=FLmRNA /CNT=221 /TID=Mm.197601.1 /TIER=FL+Stack /STK=32 /UG=Mm.197601 /LL=15528 /DEF=Mus musculus heat shock 10 kDa protein 1 (chaperonin 10) (Hspe1), mRNA. /PROD=heat shock 10 kDa protein 1 (chaperonin 10) /FL=gb:NM_008303.1 gb:U09659.1 gb:BC024385.1	2460.5999	742.7351	1814.6666	298.21667	1.3978438	2.0	0.2970214	0.36249384
gb:NM_010050.1 /DB_XREF=gi:6753637 /GEN=Dio2 /FEA=FLmRNA /CNT=27 /TID=Mm.21389.1 /TIER=FL+Stack /STK=16 /UG=Mm.21389 /LL=13371 /DEF=Mus musculus deiodinase, iodothyronine, type II (Dio2), mRNA. /PROD=deiodinase, iodothyronine, type II /FL=gb:AF177196.1 gb:NM_010050.1 gb:AF093137.1 gb:AF096875.1	400.40002	116.28194	767.9666	442.2855	1.3921314	2.0	0.2984789	0.36329603
gb:NM_010907.1 /DB_XREF=gi:6754839 /GEN=Nfkbia /FEA=FLmRNA /CNT=183 /TID=Mm.8884.1 /TIER=FL+Stack /STK=97 /UG=Mm.8884 /LL=18035 /DEF=Mus musculus nuclear factor of kappa light chain gene enhancer in B-cells inhibitor, alpha (Nfkbia), mRNA. /PROD=nuclear factor of kappa light chain geneenhancer in B-cells inhibitor, alpha /FL=gb:NM_010907.1 gb:U36277.1	2398.0999	1137.4896	1406.9668	523.849	1.3708122	2.0	0.30399716	0.3690233
gb:BC008166.1 /DB_XREF=gi:14198212 /FEA=FLmRNA /CNT=157 /TID=Mm.186.1 /TIER=FL+Stack /STK=47 /UG=Mm.186 /LL=76281 /UG_GENE=1300011C24Rik /DEF=Mus musculus, RIKEN cDNA 1300011C24 gene, clone MGC:7169 IMAGE:3257323, mRNA, complete cds. /PROD=RIKEN cDNA 1300011C24 gene /FL=gb:BC008166.1	2138.5667	693.2226	1549.7666	272.3373	1.3692713	2.0	0.30440086	0.36852798
gb:BC021383.1 /DB_XREF=gi:18204067 /FEA=FLmRNA /CNT=181 /TID=Mm.22682.1 /TIER=FL+Stack /STK=93 /UG=Mm.22682 /LL=117150 /UG_GENE=Pip5k2c /DEF=Mus musculus, phosphatidylinositol-4-phosphate 5-kinase, type II, gamma, clone MGC:29371 IMAGE:5042933, mRNA, complete cds. /PROD=phosphatidylinositol-4-phosphate 5-kinase, typeII, gamma /FL=gb:AB054591.1 gb:BC021383.1 gb:NM_054097.1	1285.8334	300.57645	910.33344	432.14334	1.2355403	3.0	0.30456167	0.36774203
gb:NM_009161.1 /DB_XREF=gi:6677922 /GEN=Sgca /FEA=FLmRNA /CNT=35 /TID=Mm.18709.1 /TIER=FL+Stack /STK=21 /UG=Mm.18709 /LL=20391 /DEF=Mus musculus sarcoglycan, alpha (50kDa dystrophin-associated glycoprotein) (Sgca), mRNA. /PROD=sarcoglycan, alpha (50kDa dystrophin-associatedglycoprotein) /FL=gb:AB024920.1 gb:AF019564.1 gb:NM_009161.1	2397.4001	819.89575	1718.4	267.866	1.3634822	2.0	0.30592343	0.36840647
gb:AF282255.1 /DB_XREF=gi:9652249 /GEN=Dscr1 /FEA=FLmRNA /CNT=119 /TID=Mm.56.1 /TIER=FL /STK=1 /UG=Mm.56 /LL=54720 /DEF=Mus musculus Down syndrome candidate region 1 protein (Dscr1) mRNA, complete cds. /PROD=Down syndrome candidate region 1 protein /FL=gb:AF260717.1 gb:AF282255.1 gb:AF237789.1 gb:NM_019466.1	870.0999	438.59137	487.6	314.48032	1.2275844	3.0	0.30714175	0.3688951
gb:NM_080555.1 /DB_XREF=gi:18017589 /GEN=Ppap2b /FEA=FLmRNA /CNT=81 /TID=Mm.29124.1 /TIER=FL+Stack /STK=32 /UG=Mm.29124 /LL=67916 /DEF=Mus musculus phosphatidic acid phosphatase type 2B (Ppap2b), mRNA. /PROD=phosphatidic acid phosphatase type 2B /FL=gb:NM_080555.1 gb:BC005558.1	2908.0999	967.49896	2102.5999	605.9832	1.2221063	3.0	0.3089313	0.37006545
gb:BC026055.1 /DB_XREF=gi:19913455 /FEA=FLmRNA /CNT=113 /TID=Mm.3379.2 /TIER=FL /STK=4 /UG=Mm.3379 /LL=20425 /UG_GENE=Shmt1 /DEF=Mus musculus, serine hydroxymethyl transferase 1 (soluble), clone MGC:14007 IMAGE:4160579, mRNA, complete cds. /PROD=serine hydroxymethyl transferase 1 (soluble) /FL=gb:AF237702.1 gb:BC026055.1	459.9	176.8502	1045.8334	731.91003	1.3478124	2.0	0.31009194	0.37047827
gb:NM_011122.1 /DB_XREF=gi:6755105 /GEN=Plod1 /FEA=FLmRNA /CNT=198 /TID=Mm.37371.1 /TIER=FL+Stack /STK=101 /UG=Mm.37371 /LL=18822 /DEF=Mus musculus procollagen-lysine, 2-oxoglutarate 5-dioxygenase 1 (Plod1), mRNA. /PROD=procollagen-lysine, 2-oxoglutarate 5-dioxygenase1 /FL=gb:BC010268.1 gb:BC006599.1 gb:AF046782.1 gb:NM_011122.1	1086.6333	485.95712	700.2	121.54659	1.3361669	2.0	0.313235	0.37325117
gb:BC026051.1 /DB_XREF=gi:19913503 /FEA=FLmRNA /CNT=157 /TID=Mm.68887.1 /TIER=FL+Stack /STK=84 /UG=Mm.68887 /LL=16779 /UG_GENE=Lamb2 /DEF=Mus musculus, laminin, beta 2, clone MGC:13735 IMAGE:4036357, mRNA, complete cds. /PROD=laminin, beta 2 /FL=gb:NM_008483.1 gb:BC026051.1	1952.7334	574.9223	1406.2666	536.5442	1.2036058	3.0	0.31505436	0.37443635
gb:BB406585 /DB_XREF=gi:16415601 /DB_XREF=BB406585 /CLONE=C330046M01 /FEA=mRNA /CNT=204 /TID=Mm.193091.1 /TIER=Stack /STK=12 /UG=Mm.193091 /UG_TITLE=Mus musculus, clone IMAGE:3708675, mRNA, partial cds	2067.4333	803.6975	1414.1334	281.79883	1.3286247	2.0	0.3152914	0.3737397
gb:BC014690.1 /DB_XREF=gi:15928416 /FEA=FLmRNA /CNT=145 /TID=Mm.1291.1 /TIER=FL+Stack /STK=44 /UG=Mm.1291 /LL=21809 /UG_GENE=Tgfb3 /DEF=Mus musculus, Transforming growth factor, beta 3, clone MGC:18512 IMAGE:3663150, mRNA, complete cds. /PROD=Transforming growth factor, beta 3 /FL=gb:NM_009368.1 gb:M32745.1 gb:BC014690.1	1086.0333	464.13068	691.8667	225.32773	1.3232585	2.0	0.31676453	0.37450805
gb:BC003436.1 /DB_XREF=gi:13097383 /FEA=FLmRNA /CNT=210 /TID=Mm.42949.1 /TIER=FL+Stack /STK=100 /UG=Mm.42949 /LL=66158 /UG_GENE=1110012O05Rik /DEF=Mus musculus, RIKEN cDNA 1110012O05 gene, clone MGC:6789 IMAGE:2647372, mRNA, complete cds. /PROD=RIKEN cDNA 1110012O05 gene /FL=gb:BC003436.1	1978.5665	616.1953	1424.0332	520.87994	1.1904018	3.0	0.31950006	0.3767611
gb:NM_009780.1 /DB_XREF=gi:6753225 /GEN=C4 /FEA=FLmRNA /CNT=175 /TID=Mm.16106.1 /TIER=FL+Stack /STK=31 /UG=Mm.16106 /LL=12268 /DEF=Mus musculus complement component 4 (within H-2S) (C4), mRNA. /PROD=complement component 4 (within H-2S) /FL=gb:M21576.1 gb:M12384.1 gb:M11729.1 gb:NM_009780.1	2024.6	703.24695	1436.8334	335.65878	1.3064458	2.0	0.32143447	0.37806022
gb:NM_025959.1 /DB_XREF=gi:13385449 /GEN=2300001E01Rik /FEA=FLmRNA /CNT=160 /TID=Mm.18472.1 /TIER=FL+Stack /STK=36 /UG=Mm.18472 /LL=67089 /DEF=Mus musculus RIKEN cDNA 2300001E01 gene (2300001E01Rik), mRNA. /PROD=RIKEN cDNA 2300001E01 /FL=gb:NM_025959.1	2173.4001	478.3655	3294.8665	1436.6143	1.2828438	2.0	0.32813188	0.38494027
gb:NM_011602.1 /DB_XREF=gi:6755808 /GEN=Tln /FEA=FLmRNA /CNT=180 /TID=Mm.4053.1 /TIER=FL+Stack /STK=78 /UG=Mm.4053 /LL=21894 /DEF=Mus musculus talin (Tln), mRNA. /PROD=talin /FL=gb:NM_011602.1	1652.2334	669.64624	1135.4999	212.40259	1.273989	2.0	0.33068797	0.38693902
gb:AK004148.1 /DB_XREF=gi:12835221 /FEA=mRNA /CNT=4 /TID=Mm.75201.1 /TIER=ConsEnd /STK=3 /UG=Mm.75201 /LL=68753 /UG_GENE=1110037P11Rik /UG_TITLE=RIKEN cDNA 1110037P11 gene /DEF=Mus musculus 18 days embryo whole body cDNA, RIKEN full-length enriched library, clone:1110037P11:similar to MYOSIN-BINDING PROTEIN H (MYBP-H) (H-PROTEIN), full insert sequence.	2268.4666	483.2075	1662.0332	768.1415	1.1574541	3.0	0.33087265	0.38615984
gb:AA798895 /DB_XREF=gi:2861850 /DB_XREF=vv94e07.r1 /CLONE=IMAGE:1230084 /FEA=EST /CNT=14 /TID=Mm.220953.2 /TIER=Stack /STK=9 /UG=Mm.220953 /UG_TITLE=Mus musculus, clone IMAGE:4206769, mRNA	840.73334	59.36046	1457.7001	844.5047	1.2622637	2.0	0.33410957	0.3889378
gb:NM_023119.1 /DB_XREF=gi:12963490 /GEN=Eno1 /FEA=FLmRNA /CNT=270 /TID=Mm.90587.1 /TIER=FL+Stack /STK=14 /UG=Mm.90587 /LL=13806 /DEF=Mus musculus enolase 1, alpha non-neuron (Eno1), mRNA. /PROD=enolase 1, alpha non-neuron /FL=gb:BC024644.1 gb:BC003891.1 gb:BC010685.1 gb:NM_023119.1	6004.6	2392.6035	4060.5667	1205.2532	1.256861	2.0	0.33570045	0.3897903
gb:NM_008540.1 /DB_XREF=gi:6678771 /GEN=Madh4 /FEA=FLmRNA /CNT=200 /TID=Mm.100399.1 /TIER=FL+Stack /STK=73 /UG=Mm.100399 /LL=17128 /DEF=Mus musculus MAD homolog 4 (Drosophila) (Madh4), mRNA. /PROD=MAD homolog 4 (Drosophila) /FL=gb:U79748.1 gb:NM_008540.1	1135.6666	320.7318	1741.6333	773.355	1.2536227	2.0	0.33665836	0.38990536
gb:NM_011340.1 /DB_XREF=gi:6755443 /GEN=Serpinf1 /FEA=FLmRNA /CNT=296 /TID=Mm.2044.1 /TIER=FL+Stack /STK=119 /UG=Mm.2044 /LL=20317 /DEF=Mus musculus serine (or cysteine) proteinase inhibitor, clade F (alpha-2 antiplasmin, pigment epithelium derived factor), member 1 (Serpinf1), mRNA. /PROD=serine (or cysteine) proteinase inhibitor, cladeF (alpha-2 antiplasmin, pigment epithelium derivedfactor), member 1 /FL=gb:D50460.1 gb:AF036164.1 gb:D87975.1 gb:NM_011340.1 gb:AF017057.1 gb:BC019852.1	6529.9336	3005.3062	4241.4	1786.3384	1.1337867	3.0	0.3392925	0.3919562
gb:NM_009260.1 /DB_XREF=gi:7106420 /GEN=Spnb2 /FEA=FLmRNA /CNT=171 /TID=Mm.123110.1 /TIER=FL+Stack /STK=11 /UG=Mm.123110 /LL=20742 /DEF=Mus musculus beta-spectrin 2, non-erythrocytic (Spnb2), mRNA. /PROD=beta-spectrin 2, non-erythrocytic /FL=gb:AF017112.1 gb:NM_009260.1	2108.6335	858.3214	1459.7334	285.89236	1.2423456	2.0	0.34001973	0.3917994
gb:BC006026.1 /DB_XREF=gi:13543747 /FEA=FLmRNA /CNT=129 /TID=Mm.1192.1 /TIER=FL+Stack /STK=38 /UG=Mm.1192 /LL=16069 /UG_GENE=Igj /DEF=Mus musculus, Similar to immunoglobulin joining chain, clone MGC:6626 IMAGE:3491646, mRNA, complete cds. /PROD=Similar to immunoglobulin joining chain /FL=gb:M90766.1 gb:BC006026.1	742.43335	501.58264	2092.5	1830.8022	1.2318512	2.0	0.34318385	0.39444423
gb:NM_012043.1 /DB_XREF=gi:6754373 /GEN=Islr /FEA=FLmRNA /CNT=72 /TID=Mm.38426.1 /TIER=FL+Stack /STK=23 /UG=Mm.38426 /LL=26968 /DEF=Mus musculus immunoglobulin superfamily containing leucine-rich repeat (Islr), mRNA. /PROD=immunoglobulin superfamily containingleucine-rich repeat /FL=gb:BC006602.1 gb:NM_012043.1 gb:AB024538.1	2557.5	1151.92	1698.9668	672.7698	1.1147153	3.0	0.34623244	0.39694327
gb:BC004674.1 /DB_XREF=gi:13435593 /FEA=FLmRNA /CNT=195 /TID=Mm.28028.1 /TIER=FL+Stack /STK=130 /UG=Mm.28028 /DEF=Mus musculus, Similar to RNA binding motif protein 10, clone MGC:7826 IMAGE:3500403, mRNA, complete cds. /PROD=Similar to RNA binding motif protein 10 /FL=gb:BC004674.1	659.73334	137.62909	991.30005	453.94376	1.2106925	2.0	0.3496703	0.39987487
gb:BC007159.1 /DB_XREF=gi:13938088 /FEA=FLmRNA /CNT=1 /TID=Mm.212861.1 /TIER=FL /STK=1 /UG=Mm.212861 /DEF=Mus musculus, Similar to L1 repeat, Tf subfamily, member 30, clone MGC:7372 IMAGE:3487559, mRNA, complete cds. /PROD=Similar to L1 repeat, Tf subfamily, member 30 /FL=gb:BC007159.1	744.03326	325.80676	2445.2	2433.6653	1.2000223	2.0	0.35299635	0.40266415
gb:BC018358.1 /DB_XREF=gi:17390834 /FEA=FLmRNA /CNT=217 /TID=Mm.28830.1 /TIER=FL+Stack /STK=53 /UG=Mm.28830 /LL=59027 /UG_GENE=Pbef-pending /DEF=Mus musculus, pre-B-cell colony-enhancing factor, clone MGC:19365 IMAGE:2609271, mRNA, complete cds. /PROD=pre-B-cell colony-enhancing factor /FL=gb:BC004059.1 gb:NM_021524.1 gb:AF234625.1 gb:BC018358.1	3284.8665	1116.2933	2368.5332	936.2697	1.0893528	3.0	0.35567966	0.40470818
gb:NM_020003.1 /DB_XREF=gi:9910457 /GEN=0610031J06Rik /FEA=FLmRNA /CNT=225 /TID=Mm.21976.1 /TIER=FL+Stack /STK=67 /UG=Mm.21976 /LL=56700 /DEF=Mus musculus RIKEN cDNA 0610031J06 gene (0610031J06Rik), mRNA. /PROD=kidney predominant protein NCU-G1 /FL=gb:NM_020003.1 gb:BC021547.1 gb:AB027142.1 gb:AB027141.1	1087.2333	427.39923	757.9666	216.22142	1.190669	2.0	0.35594267	0.40399492
gb:BC007159.1 /DB_XREF=gi:13938088 /FEA=FLmRNA /CNT=1 /TID=Mm.212861.1 /TIER=FL /STK=1 /UG=Mm.212861 /DEF=Mus musculus, Similar to L1 repeat, Tf subfamily, member 30, clone MGC:7372 IMAGE:3487559, mRNA, complete cds. /PROD=Similar to L1 repeat, Tf subfamily, member 30 /FL=gb:BC007159.1	705.5	321.25174	2421.8333	2482.401	1.1876372	2.0	0.35690385	0.40407568
gb:NM_007687.1 /DB_XREF=gi:6680923 /GEN=Cfl1 /FEA=FLmRNA /CNT=216 /TID=Mm.4024.1 /TIER=FL+Stack /STK=87 /UG=Mm.4024 /LL=12631 /DEF=Mus musculus cofilin 1, non-muscle (Cfl1), mRNA. /PROD=cofilin 1, non-muscle /FL=gb:D00472.1 gb:NM_007687.1	1391.6333	631.5833	931.5667	235.12334	1.1824073	2.0	0.35856915	0.4049512
gb:AK002700.1 /DB_XREF=gi:12832875 /GEN=Sult1a1 /FEA=mRNA /CNT=24 /TID=Mm.17339.2 /TIER=ConsEnd /STK=6 /UG=Mm.17339 /LL=20887 /UG_TITLE=sulfotransferase family 1A, phenol-preferring, member 1 /DEF=Mus musculus adult male kidney cDNA, RIKEN full-length enriched library, clone:0610030E04:sulfotransferase family 1A, phenol-preferring, member 1, full insert sequence.	3867.8665	1119.8217	2661.4333	1635.5717	1.0541868	3.0	0.36919752	0.41591978
gb:NM_009841.1 /DB_XREF=gi:6753331 /GEN=Cd14 /FEA=FLmRNA /CNT=91 /TID=Mm.3460.1 /TIER=FL+Stack /STK=50 /UG=Mm.3460 /LL=12475 /DEF=Mus musculus CD14 antigen (Cd14), mRNA. /PROD=CD14 antigen /FL=gb:NM_009841.1	1378.0	433.33588	948.39996	558.794	1.0522685	3.0	0.3699491	0.4157349
gb:AF357006.1 /DB_XREF=gi:15420511 /GEN=Loxl1 /FEA=FLmRNA /CNT=93 /TID=Mm.21049.1 /TIER=FL+Stack /STK=43 /UG=Mm.21049 /LL=16949 /DEF=Mus musculus lysyl oxidase-like 1 (Loxl1) mRNA, complete cds. /PROD=lysyl oxidase-like 1 /FL=gb:AF357006.1	4293.567	1819.6642	3059.7668	947.52747	1.0416377	3.0	0.37414107	0.41940752
gb:AK011360.1 /DB_XREF=gi:12847432 /FEA=mRNA /CNT=64 /TID=Mm.142187.1 /TIER=Stack /STK=24 /UG=Mm.142187 /LL=69202 /UG_GENE=2610009E16Rik /UG_TITLE=RIKEN cDNA 2610009E16 gene /DEF=Mus musculus 10 days embryo whole body cDNA, RIKEN full-length enriched library, clone:2610009E16:homolog to PARATHYMOSIN (ZINC-BINDING 11.5 KDA PROTEIN), full insert sequence.	3434.7334	1605.6941	2261.0	795.5188	1.1344959	2.0	0.37425452	0.41850138
gb:NM_030127.1 /DB_XREF=gi:14994304 /GEN=9530081K03Rik /FEA=FLmRNA /CNT=52 /TID=Mm.41957.1 /TIER=FL+Stack /STK=11 /UG=Mm.41957 /LL=78558 /DEF=Mus musculus RIKEN cDNA 9530081K03 gene (9530081K03Rik), mRNA. /PROD=toll-associated serine protease /FL=gb:AY037300.1 gb:NM_030127.1	4126.9	1624.9299	2965.7334	755.9192	1.1222255	2.0	0.37839863	0.42209575
gb:NM_013640.1 /DB_XREF=gi:7305416 /GEN=Psmb10 /FEA=FLmRNA /CNT=105 /TID=Mm.787.1 /TIER=FL+Stack /STK=51 /UG=Mm.787 /LL=19171 /DEF=Mus musculus proteasome (prosome, macropain) subunit, beta type 10 (Psmb10), mRNA. /PROD=proteasome (prosomome, macropain) subunit, betatype 10 /FL=gb:BC004730.1 gb:D85561.1 gb:NM_013640.1 gb:U77784.1	1687.7666	727.43	1199.1	208.05478	1.1186851	2.0	0.37960413	0.42240262
gb:BC025618.1 /DB_XREF=gi:19343735 /FEA=FLmRNA /CNT=513 /TID=Mm.205791.1 /TIER=FL+Stack /STK=208 /UG=Mm.205791 /DEF=Mus musculus, clone MGC:38134 IMAGE:5320976, mRNA, complete cds. /PROD=Unknown (protein for MGC:38134) /FL=gb:BC025811.1 gb:BC025618.1 gb:BC025627.1 gb:BC021496.1	4273.3335	4025.0786	1660.7999	424.9315	1.117999	2.0	0.3798383	0.4216298
gb:AF375476.1 /DB_XREF=gi:14150812 /FEA=FLmRNA /CNT=2 /TID=Mm.1566.2 /TIER=FL /STK=1 /UG=Mm.1566 /LL=107503 /UG_GENE=Atf5 /DEF=Mus musculus activating transcription factor 5-beta mRNA, complete cds. /PROD=activating transcription factor 5-beta /FL=gb:AF375476.1	1004.0	431.01715	696.56665	202.9533	1.1177157	2.0	0.379935	0.4207085
gb:NM_011693.1 /DB_XREF=gi:6755960 /GEN=Vcam1 /FEA=FLmRNA /CNT=224 /TID=Mm.1021.1 /TIER=FL+Stack /STK=153 /UG=Mm.1021 /LL=22329 /DEF=Mus musculus vascular cell adhesion molecule 1 (Vcam1), mRNA. /PROD=vascular cell adhesion molecule 1 /FL=gb:NM_011693.1 gb:M84487.1	1582.9332	759.63043	1035.5	381.54373	1.1154205	2.0	0.38071966	0.42055163
gb:AI852593 /DB_XREF=gi:5496499 /DB_XREF=UI-M-BH0-aiu-b-12-0-UI.s1 /CLONE=UI-M-BH0-aiu-b-12-0-UI /FEA=mRNA /CNT=61 /TID=Mm.26671.1 /TIER=Stack /STK=10 /UG=Mm.26671 /UG_TITLE=Mus musculus, clone IMAGE:3487914, mRNA	1663.8334	910.53546	1027.2001	404.96207	1.1065224	2.0	0.38377926	0.42290238
gb:BC024505.1 /DB_XREF=gi:19353592 /FEA=FLmRNA /CNT=296 /TID=Mm.28622.1 /TIER=FL /STK=1 /UG=Mm.28622 /DEF=Mus musculus, clone MGC:37456 IMAGE:4983496, mRNA, complete cds. /PROD=Unknown (protein for MGC:37456) /FL=gb:BC016252.1 gb:BC024505.1	1114.8334	543.79114	752.76666	167.1206	1.1023498	2.0	0.38522372	0.42346627
gb:NM_008826.1 /DB_XREF=gi:6679286 /GEN=Pfkl /FEA=FLmRNA /CNT=5 /TID=Mm.1166.1 /TIER=FL /STK=2 /UG=Mm.1166 /LL=18641 /DEF=Mus musculus phosphofructokinase, liver, B-type (Pfkl), mRNA. /PROD=phosphofructokinase, liver, B-type /FL=gb:NM_008826.1 gb:J03928.1	967.0	495.60147	652.26666	33.80921	1.097394	2.0	0.38694733	0.42433354
gb:AK009097.1 /DB_XREF=gi:12843674 /FEA=mRNA /CNT=1 /TID=Mm.158769.2 /TIER=ConsEnd /STK=0 /UG=Mm.158769 /LL=71886 /UG_GENE=2310003M01Rik /UG_TITLE=RIKEN cDNA 2310003M01 gene /DEF=Mus musculus adult male tongue cDNA, RIKEN full-length enriched library, clone:2310002L09:hypothetical protein, full insert sequence.	1547.9	624.05414	1107.8666	430.20523	1.0055269	3.0	0.38872316	0.42525378
gb:NM_008344.1 /DB_XREF=gi:6680386 /GEN=Igfbp6 /FEA=FLmRNA /CNT=59 /TID=Mm.21373.1 /TIER=FL+Stack /STK=33 /UG=Mm.21373 /LL=16012 /DEF=Mus musculus insulin-like growth factor binding protein 6 (Igfbp6), mRNA. /PROD=insulin-like growth factor binding protein 6 /FL=gb:BC012723.1 gb:NM_008344.1	1815.6	908.03644	1229.5	506.16608	0.97650135	3.0	0.40083343	0.437448
gb:NM_022331.1 /DB_XREF=gi:11612514 /GEN=Herpud1 /FEA=FLmRNA /CNT=280 /TID=Mm.29151.1 /TIER=FL+Stack /STK=140 /UG=Mm.29151 /LL=64209 /DEF=Mus musculus homocysteine-inducible, endoplasmic reticulum stress-inducible, ubiquitin-like domain member 1 (Herpud1), mRNA. /PROD=homocysteine-inducible, endoplasmic reticulumstress-inducible, ubiquitin-like domain member 1 /FL=gb:AB034991.1 gb:NM_022331.1 gb:BC013523.1	4177.6333	2074.8477	2893.8	346.59915	1.0570768	2.0	0.4012997	0.4369066

gb:NM_007657.1 /DB_XREF=gi:6680893 /GEN=Cd9 /FEA=FLmRNA /CNT=324 /TID=Mm.2956.1 /TIER=FL+Stack /STK=135 /UG=Mm.2956 /LL=12527 /DEF=Mus musculus CD9 antigen (Cd9), mRNA. /PROD=CD9 antigen /FL=gb:L08115.1 gb:NM_007657.1	2590.3333	1327.7048	1686.2667	684.79297	1.0481877	2.0	0.40454426	0.43938538
gb:NM_009870.1 /DB_XREF=gi:6753379 /GEN=Cdk4 /FEA=FLmRNA /CNT=239 /TID=Mm.6839.1 /TIER=FL+Stack /STK=122 /UG=Mm.6839 /LL=12567 /DEF=Mus musculus cyclin-dependent kinase 4 (Cdk4), mRNA. /PROD=cyclin-dependent kinase 4 /FL=gb:L01640.1 gb:NM_009870.1	993.26666	444.49567	694.5334	217.49274	1.0456059	2.0	0.40549216	0.43936384
gb:AK004342.1 /DB_XREF=gi:12835491 /FEA=mRNA /CNT=37 /TID=Mm.178550.2 /TIER=Stack /STK=19 /UG=Mm.178550 /LL=66478 /UG_GENE=2410026K10Rik /UG_TITLE=RIKEN cDNA 2410026K10 gene /DEF=Mus musculus 18 days embryo whole body cDNA, RIKEN full-length enriched library, clone:1110061M03:homolog to T-CELL SURFACE GLYCOPROTEIN E2 PRECURSOR (E2 ANTIGEN) (CD99) (MIC2 PROTEIN) (12E7), full insert sequence.	849.5334	656.2247	453.93335	76.852875	1.0370659	2.0	0.40864527	0.44172606
gb:BC025654.1 /DB_XREF=gi:19343912 /FEA=FLmRNA /CNT=105 /TID=Mm.25602.1 /TIER=FL+Stack /STK=80 /UG=Mm.25602 /DEF=Mus musculus, Similar to DKFZP586L151 protein, clone MGC:38296 IMAGE:5342879, mRNA, complete cds. /PROD=Similar to DKFZP586L151 protein /FL=gb:BC025654.1	1097.0668	515.759	756.93335	245.942	1.0310311	2.0	0.41088986	0.44309738
gb:NM_019521.1 /DB_XREF=gi:9506714 /GEN=Gas6 /FEA=FLmRNA /CNT=147 /TID=Mm.3982.1 /TIER=FL+Stack /STK=46 /UG=Mm.3982 /LL=14456 /DEF=Mus musculus growth arrest specific 6 (Gas6), mRNA. /PROD=growth arrest specific 6 /FL=gb:BC005444.1 gb:NM_019521.1	2380.3	1071.7491	1663.9	750.3695	0.9484235	3.0	0.41288343	0.4441921
gb:BC010564.1 /DB_XREF=gi:14714826 /FEA=FLmRNA /CNT=83 /TID=Mm.219659.1 /TIER=FL+Stack /STK=24 /UG=Mm.219659 /LL=15267 /UG_GENE=Hist2 /DEF=Mus musculus, H2A histone family, member O, clone MGC:5956 IMAGE:3582122, mRNA, complete cds. /PROD=H2A histone family, member O /FL=gb:NM_013549.1 gb:BC010564.1	1537.0668	912.65375	992.8333	134.19629	1.0218685	2.0	0.41432413	0.44468832
gb:BC015459.1 /DB_XREF=gi:15930030 /FEA=FLmRNA /CNT=83 /TID=Mm.18814.1 /TIER=FL+Stack /STK=10 /UG=Mm.18814 /LL=66898 /UG_GENE=1300006M19Rik /DEF=Mus musculus, RIKEN cDNA 1300006M19 gene, clone MGC:8269 IMAGE:3592632, mRNA, complete cds. /PROD=RIKEN cDNA 1300006M19 gene /FL=gb:BC015459.1	992.26666	1056.4161	371.96667	48.13329	1.015961	2.0	0.41655517	0.4460284
gb:NM_008788.1 /DB_XREF=gi:6679220 /GEN=Pcolce /FEA=FLmRNA /CNT=152 /TID=Mm.18808.1 /TIER=FL+Stack /STK=74 /UG=Mm.18808 /LL=18542 /DEF=Mus musculus procollagen C-proteinase enhancer protein (Pcolce), mRNA. /PROD=procollagen C-proteinase enhancer protein /FL=gb:NM_008788.1	3300.6667	1719.5554	2233.6333	697.6866	0.99593264	2.0	0.42421845	0.4531651
gb:AK007826.1 /DB_XREF=gi:12841627 /FEA=mRNA /CNT=37 /TID=Mm.157783.14 /TIER=Stack /STK=27 /UG=Mm.157783 /LL=104911 /UG_GENE=AI893585 /UG_TITLE=expressed sequence AI893585 /DEF=Mus musculus 10 day old male pancreas cDNA, RIKEN full-length enriched library, clone:1810048H17:immunoglobulin heavy chain, (J558 family), full insert sequence.	536.60004	343.23163	1281.9667	1260.6519	0.98811466	2.0	0.42725167	0.45533392
gb:NM_010890.1 /DB_XREF=gi:13994148 /GEN=Nedd4a /FEA=FLmRNA /CNT=23 /TID=Mm.16553.1 /TIER=FL /STK=1 /UG=Mm.16553 /LL=17999 /DEF=Mus musculus neural precursor cell expressed, developmentally down-regulated gene 4a (Nedd4a), mRNA. /PROD=neural precursor cell expressed, developmentallydown-regulated gene 4a /FL=gb:NM_010890.1	1768.7333	992.5988	1140.6	479.7904	0.98683304	2.0	0.42775115	0.45479864
gb:BC025131.1 /DB_XREF=gi:19264044 /FEA=FLmRNA /CNT=81 /TID=Mm.41931.1 /TIER=FL+Stack /STK=23 /UG=Mm.41931 /LL=50530 /UG_GENE=Mfap5-pending /DEF=Mus musculus, Similar to microfibrillar associated protein 5, clone MGC:35969 IMAGE:3982519, mRNA, complete cds. /PROD=Similar to microfibrillar associated protein 5 /FL=gb:AF180805.1 gb:NM_015776.1 gb:BC025131.1	4995.033	2107.182	3669.4668	1422.718	0.9030253	3.0	0.43307415	0.45938239
gb:NM_013472.1 /DB_XREF=gi:7304890 /GEN=Anxa6 /FEA=FLmRNA /CNT=413 /TID=Mm.22619.1 /TIER=FL+Stack /STK=233 /UG=Mm.22619 /LL=11749 /DEF=Mus musculus annexin A6 (Anxa6), mRNA. /PROD=annexin A6 /FL=gb:NM_013472.1 gb:BC005595.1	2057.4666	881.61163	1507.8334	424.67703	0.97284544	2.0	0.4332443	0.45849165
gb:BC019775.1 /DB_XREF=gi:18043602 /FEA=FLmRNA /CNT=156 /TID=Mm.214514.1 /TIER=FL+Stack /STK=98 /UG=Mm.214514 /LL=116847 /UG_GENE=Prelp /DEF=Mus musculus, proline arginine-rich end leucine-rich repeat, clone MGC:25825 IMAGE:4165772, mRNA, complete cds. /PROD=proline arginine-rich end leucine-rich repeat /FL=gb:BC019775.1 gb:NM_054077.1	3675.5667	1965.7422	2444.2334	1320.7708	0.90055424	3.0	0.4341985	0.45843282
gb:NM_032541.1 /DB_XREF=gi:14211541 /GEN=Hamp /FEA=FLmRNA /CNT=18 /TID=Mm.23995.1 /TIER=FL+Stack /STK=12 /UG=Mm.23995 /LL=84506 /DEF=Mus musculus hepcidin antimicrobial peptide (Hamp), mRNA. /PROD=hepcidin antimicrobial peptide /FL=gb:NM_032541.1 gb:AF297664.1 gb:BC021587.1	4090.6667	2158.8376	2871.2	441.99368	0.95850414	2.0	0.43895587	0.46238044
gb:AK008203.1 /DB_XREF=gi:12842246 /FEA=FLmRNA /CNT=83 /TID=Mm.18814.1 /TIER=Stack /STK=25 /UG=Mm.18814 /LL=66898 /UG_GENE=1300006M19Rik /UG_TITLE=RIKEN cDNA 1300006M19 gene /DEF=Mus musculus adult male small intestine cDNA, RIKEN full-length enriched library, clone:2010012D17:homolog to INSULIN RECEPTOR TYROSINE KINASE SUBSTRATE, full insert sequence. /FL=gb:BC015459.1	2052.7	2641.6304	592.10004	181.3516	0.95542985	2.0	0.4401908	0.46260792
gb:BC007474.1 /DB_XREF=gi:13938634 /FEA=FLmRNA /CNT=521 /TID=Mm.140785.1 /TIER=FL+Stack /STK=156 /UG=Mm.140785 /LL=20249 /UG_GENE=Scd1 /DEF=Mus musculus, stearoyl-Coenzyme A desaturase 1, clone MGC:6427 IMAGE:3599932, mRNA, complete cds. /PROD=stearoyl-Coenzyme A desaturase 1 /FL=gb:BC007474.1 gb:NM_009127.1	6781.533	901.3425	11328.734	8234.607	0.95077056	2.0	0.44206956	0.46350944
gb:BC025161.1 /DB_XREF=gi:19263792 /FEA=FLmRNA /CNT=98 /TID=Mm.29503.1 /TIER=FL+Stack /STK=47 /UG=Mm.29503 /LL=68479 /UG_GENE=1110007B08Rik /DEF=Mus musculus, hypothetical protein MGC1346, clone MGC:36856 IMAGE:4235469, mRNA, complete cds. /PROD=hypothetical protein MGC1346 /FL=gb:BC025161.1	990.4001	521.377	693.3333	156.94771	0.94498926	2.0	0.4444127	0.46489254
gb:NM_013550.1 /DB_XREF=gi:7305140 /GEN=Hist4 /FEA=FLmRNA /CNT=9 /TID=Mm.193557.1 /TIER=FL /STK=1 /UG=Mm.193557 /LL=15269 /DEF=Mus musculus histone 4 protein (Hist4), mRNA. /PROD=histone 4 protein /FL=gb:NM_013550.1	2056.1667	1616.3438	1194.2001	14.616449	0.9236334	2.0	0.45318398	0.4729782
gb:AK012427.1 /DB_XREF=gi:12849162 /FEA=mRNA /CNT=2 /TID=Mm.331.5 /TIER=ConsEnd /STK=0 /UG=Mm.331 /LL=22190 /UG_GENE=Ubc /UG_TITLE=ubiquitin C /DEF=Mus musculus 11 days embryo whole body cDNA, RIKEN full-length enriched library, clone:2700054O04:homolog to POLYUBIQUITIN UBC (SIMILAR TO UBIQUITIN C), full insert sequence.	1015.76666	444.64703	1562.2001	960.1859	0.8944442	2.0	0.46547025	0.4846869
gb:AK011936.1 /DB_XREF=gi:12848366 /FEA=mRNA /CNT=86 /TID=Mm.203875.1 /TIER=Stack /STK=50 /UG=Mm.203875 /LL=72478 /UG_GENE=2610209L21Rik /UG_TITLE=RIKEN cDNA 2610209L21 gene /DEF=Mus musculus 10 days embryo whole body cDNA, RIKEN full-length enriched library, clone:2610209L21:unclassifiable transcript, full insert sequence.	1655.0	803.78375	1204.4667	482.45084	0.8324072	3.0	0.4662473	0.48438507
gb:NM_010664.1 /DB_XREF=gi:6754481 /GEN=Krt1-18 /FEA=FLmRNA /CNT=380 /TID=Mm.22479.1 /TIER=FL+Stack /STK=149 /UG=Mm.22479 /LL=16668 /DEF=Mus musculus keratin complex 1, acidic, gene 18 (Krt1-18), mRNA. /PROD=keratin complex 1, acidic, gene 18 /FL=gb:M36376.1 gb:NM_010664.1 gb:M11686.1	497.56665	5.105219	883.39996	751.11115	0.8897052	2.0	0.46749777	0.48457533
gb:BB204720 /DB_XREF=gi:16351882 /DB_XREF=BB204720 /CLONE=A430059G22 /FEA=FLmRNA /CNT=74 /TID=Mm.182434.2 /TIER=Stack /STK=16 /UG=Mm.182434 /UG_TITLE=Mus musculus, Similar to RIKEN cDNA 1700066C05 gene, clone MGC:28125 IMAGE:3980327, mRNA, complete cds /FL=gb:BC020078.1	416.06668	181.6803	901.2	929.3317	0.88737375	2.0	0.4684986	0.48450652
gb:NM_009156.1 /DB_XREF=gi:6677916 /GEN=Sepw1 /FEA=FLmRNA /CNT=217 /TID=Mm.42829.1 /TIER=FL+Stack /STK=83 /UG=Mm.42829 /LL=20364 /DEF=Mus musculus selenoprotein W, muscle 1 (Sepw1), mRNA. /PROD=selenoprotein W, muscle 1 /FL=gb:U67890.1 gb:AF241527.2 gb:AF015284.1 gb:NM_009156.1	2498.2	1507.5862	1717.6	341.65616	0.8746445	2.0	0.4740024	0.48908427
gb:NM_134051.1 /DB_XREF=gi:19527267 /GEN=AI893585 /FEA=FLmRNA /CNT=3 /TID=Mm.157783.1 /TIER=FL /STK=2 /UG=Mm.157783 /LL=104911 /DEF=Mus musculus expressed sequence AI893585 (AI893585), mRNA. /PROD=expressed sequence AI893585 /FL=gb:NM_134051.1 gb:BC013488.1	1823.8334	1609.4973	5154.1665	6440.4775	0.8689117	2.0	0.47650295	0.49054953
gb:BC019425.1 /DB_XREF=gi:18043870 /FEA=FLmRNA /CNT=5 /TID=Mm.157783.6 /TIER=FL /STK=1 /UG=Mm.157783 /LL=104911 /UG_GENE=AI893585 /DEF=Mus musculus, clone MGC:30346 IMAGE:4459683, mRNA, complete cds. /PROD=Unknown (protein for MGC:30346) /FL=gb:BC019425.1	893.8333	797.18445	2621.0002	3428.4812	0.8498838	2.0	0.48490024	0.49806497
gb:NM_032541.1 /DB_XREF=gi:14211541 /GEN=Hamp /FEA=FLmRNA /CNT=18 /TID=Mm.23995.1 /TIER=FL+Stack /STK=12 /UG=Mm.23995 /LL=84506 /DEF=Mus musculus hepcidin antimicrobial peptide (Hamp), mRNA. /PROD=hepcidin antimicrobial peptide /FL=gb:NM_032541.1 gb:AF297664.1 gb:BC021587.1	3380.7666	2024.4442	2395.367	236.9967	0.8373586	2.0	0.4905101	0.5026898
gb:AK017926.1 /DB_XREF=gi:12857423 /FEA=mRNA /CNT=124 /TID=Mm.21697.1 /TIER=Stack /STK=56 /UG=Mm.21697 /LL=74747 /UG_GENE=5830413E08Rik /UG_TITLE=RIKEN cDNA 5830413E08 gene /DEF=Mus musculus adult male thymus cDNA, RIKEN full-length enriched library, clone:5830413E08:homolog to HYPOTHETICAL 25.4 KDA PROTEIN, full insert sequence.	2388.0667	451.77954	1741.9	1306.4413	0.8096306	2.0	0.50316334	0.51449585
gb:NM_017370.1 /DB_XREF=gi:8850218 /GEN=Hp /FEA=FLmRNA /CNT=154 /TID=Mm.26730.1 /TIER=FL+Stack /STK=33 /UG=Mm.26730 /LL=15439 /DEF=Mus musculus haptoglobin (Hp), mRNA. /PROD=haptoglobin /FL=gb:NM_017370.1 gb:M96827.1	705.19995	506.99698	440.69998	253.31256	0.8083316	2.0	0.5037641	0.51395255
gb:AF127033.1 /DB_XREF=gi:9937096 /FEA=FLmRNA /CNT=402 /TID=Mm.3760.1 /TIER=FL+Stack /STK=97 /UG=Mm.3760 /LL=14104 /UG_GENE=Fasn /DEF=Mus musculus fatty acid synthase mRNA, complete cds. /PROD=fatty acid synthase /FL=gb:AF127033.1	1379.7334	459.9069	2322.4333	1995.7894	0.797231	2.0	0.5089267	0.51805544
gb:NM_010180.1 /DB_XREF=gi:6753821 /GEN=Fbln1 /FEA=FLmRNA /CNT=91 /TID=Mm.219663.1 /TIER=FL+Stack /STK=41 /UG=Mm.219663 /LL=14114 /DEF=Mus musculus fibulin 1 (Fbln1), mRNA. /PROD=fibulin 1 /FL=gb:NM_010180.1	835.6333	728.61206	505.8666	168.10336	0.76385224	2.0	0.52476585	0.53298366
gb:NM_009052.1 /DB_XREF=gi:6677720 /GEN=Rex3 /FEA=FLmRNA /CNT=77 /TID=Mm.14768.1 /TIER=FL+Stack /STK=56 /UG=Mm.14768 /LL=19716 /DEF=Mus musculus reduced expression 3 (Rex3), mRNA. /PROD=reduced expression 3 /FL=gb:AF051347.1 gb:NM_009052.1 gb:AF097438.1	1297.2999	1452.1323	657.06665	84.7658	0.76234937	2.0	0.5254902	0.53252804
gb:AK010307.1 /DB_XREF=gi:12845653 /FEA=mRNA /CNT=161 /TID=Mm.3014.1 /TIER=ConsEnd /STK=0 /UG=Mm.3014 /LL=66386 /UG_GENE=2310039H15Rik /UG_TITLE=RIKEN cDNA 2310039H15 gene /DEF=Mus musculus ES cells cDNA, RIKEN full-length enriched library, clone:2400010C15:homolog to ACYL CARRIER PROTEIN, MITOCHONDRIAL PRECURSOR (ACP) (NADH-UBIQUINONE OXIDOREDUCTASE 9.6 KDA SUBUNIT) (EC 1.6.5.3) (EC 1.6.99.3) (CI-SDAP), full insert sequ...	1906.5	288.7541	2880.9336	2196.1677	0.7619484	2.0	0.5256836	0.53153753
gb:NM_021400.1 /DB_XREF=gi:10946747 /GEN=Prg4 /FEA=FLmRNA /CNT=10 /TID=Mm.212696.1 /TIER=FL /STK=6 /UG=Mm.212696 /LL=65022 /DEF=Mus musculus proteoglycan 4 (megakaryocyte stimulating factor, articular superficial zone protein) (Prg4), mRNA. /PROD=proteoglycan 4 (megakaryocyte stimulatingfactor, articular superficial zone protein) /FL=gb:AB034730.1 gb:NM_021400.1	2187.3333	1512.217	1530.1998	243.50616	0.74308985	2.0	0.5348581	0.5396124
gb:BC021831.1 /DB_XREF=gi:18256103 /FEA=mRNA /CNT=1 /TID=Mm.218360.1 /TIER=ConsEnd /STK=0 /UG=Mm.218360 /DEF=Mus musculus, clone IMAGE:3983821, mRNA, partial cds. /PROD=Unknown (protein for IMAGE:3983821)	828.2	179.45923	1305.7333	1128.0948	0.72408855	2.0	0.54425615	0.5478765
gb:NM_009879.2 /DB_XREF=gi:15638941 /GEN=Cdv1 /FEA=FLmRNA /CNT=46 /TID=Mm.10125.1 /TIER=FL+Stack /STK=11 /UG=Mm.10125 /LL=12589 /DEF=Mus musculus carnitine deficiency-associated gene expressed in ventricle 1 (Cdv1), mRNA. /PROD=carnitine deficiency-associated gene expressedin ventricle 1 /FL=gb:AF354757.1 gb:NM_009879.2	2408.5	1793.4084	1702.0	263.94318	0.6750568	2.0	0.569223	0.57174164
gb:BC025093.1 /DB_XREF=gi:19263816 /FEA=FLmRNA /CNT=3 /TID=Mm.11480.2 /TIER=FL /STK=1 /UG=Mm.11480 /LL=67239 /UG_GENE=2810470K21Rik /DEF=Mus musculus, RIKEN cDNA 2810470K21 gene, clone MGC:35797 IMAGE:4009314, mRNA, complete cds. /PROD=RIKEN cDNA 2810470K21 gene /FL=gb:BC025093.1	1150.7666	101.20684	2018.6	2279.5244	0.6587568	2.0	0.5777513	0.57902664
gb:NM_054037.1 /DB_XREF=gi:16905068 /GEN=Scgb3a1 /FEA=FLmRNA /CNT=13 /TID=Mm.22802.1 /TIER=FL /STK=7 /UG=Mm.22802 /LL=68662 /DEF=Mus musculus secretoglobin, family 3A, member 1 (Scgb3a1), mRNA. /PROD=UGRP2 type A /FL=gb:NM_054037.1 gb:AF313456.1	6362.733	6998.1064	3526.5332	5637.334	0.5466609	3.0	0.62264764	0.62264764
